# Temporal maintenance of an adolescent suicide attempt screening score in national surveys: An observational study

**DOI:** 10.1097/MD.0000000000050513

**Published:** 2026-08-28

**Authors:** Xiaotian Ma, Su Jia, Junjie Zhang

**Affiliations:** a School of Artificial Intelligence, Jiangsu Vocational College of Business, Nantong, Jiangsu, China; b Shanghai Academy of Global Governance & Area Studies, Shanghai International Studies University, Shanghai, China; c School of Economics and Finance, Shanghai International Studies University, Shanghai, China.

**Keywords:** adolescent health, calibration, model updating, suicide attempt, temporal validation

## Abstract

Screening scores may retain risk ranking while their probabilities become inaccurate in later populations. The information needed to repair this temporal drift is rarely quantified for adolescent suicide screening. We analyzed 5 independent national Youth Risk Behavior Survey cross sections from 2015 through 2023. Of 81,401 sampled students, 68,682 had observed past year attempt status. A survey weighted 2015 ridge score based on 14 harmonized predictors was frozen and transported to 4 later surveys. For each later survey, 50 repeated 5-fold evaluations that separated primary sampling units compared no update, intercept recalibration, intercept and slope recalibration, and full refitting of the same specification using 250 to 4000 target labels. Direct suicide items and identity attributes were excluded. The analysis included 6855 reported attempts. Frozen score areas under the receiver operating characteristic curve remained 0.852 to 0.860, but average risk was overestimated in 2017, 2019, and 2023. Predictor distribution and conditional model components opposed each other in 3 surveys. With 1000 labels, intercept recalibration met a prespecified descriptive calibration rule in 76 to 88% of repetitions; rates were 92 to 100% with 2000 labels. At 250 labels, refitting reduced median area under the curve by 0.036 to 0.043 and increased Brier score by 0.0047 to 0.0060. Intercept recalibration outperformed refitting in all 50 paired repetitions. Alternative ridge penalties did not remove this instability. Stable discrimination did not ensure stable absolute risk. Intercept recalibration was more reliable than coefficient refitting with scarce new outcomes. Maintenance requires reserved current data and an update strategy matched to the observed drift.

## 1. Introduction

Risk scores are often carried forward after development as if their probabilities had a permanent meaning. That assumption is unsafe. A score can retain almost the same area under the receiver operating characteristic curve (AUROC) while systematically overestimating or underestimating absolute risk because discrimination and calibration answer different questions.^[1,2]^ Temporal validation in a genuinely later population is therefore more informative than another random split of pooled observations when intended use extends to future survey cycles or service periods.^[3]^

This distinction is important in adolescent suicide research. Recent systematic reviews show rapid growth in regression and machine learning models, but persistent reliance on internal validation and limited evidence about performance in independent populations.^[4,5]^ Studies using national youth surveys have shown that useful risk stratification is possible without direct suicide questions and that some models transport across countries.^[6,7]^ More recent national analyses have continued to compare algorithms and influential predictors.^[8]^ Temporal studies in school and college samples have reported a need for local calibration in 1 setting and limited average discrimination loss in another.^[9,10]^ These studies establish feasibility, but do not determine how model maintenance should change when only a limited number of current outcomes are available.

Large health record studies provide a deliberately mixed picture. Penfold et al analyzed 1.4 million outpatient visits among 256,823 adolescents in 7 health systems and found almost identical discrimination for an existing model, a recalibrated model, and a newly learned model.^[11]^ Walker et al evaluated about 10.8 million visits from 1,799,765 patients in later calendar data and found preserved calibration and attempt discrimination, but a modest decrease in discrimination for suicide death.^[12]^ In contrast, a prospective study of 115,905 predictions among 77,973 patients found early miscalibration that was corrected through logistic recalibration.^[13]^ Simon et al then found stable discrimination and calibration across about 13.1 million later visits, with no consistent advantage from annual redevelopment.^[14]^ Across 3 clinical data sources containing 285,320 adolescents and 3389 attempts, transported models could improve or deteriorate depending on the source, target, and algorithm.^[15]^ Thus, large sample size does not guarantee either drift or stability, and a more recent or more complex model is not automatically better.

Calibration drift may reflect changes in outcome frequency, predictor distributions, predictor outcome relations, or combinations of these processes.^[16]^ Updating only the intercept estimates 1 new parameter and can correct calibration in the large. Estimating an intercept and calibration slope can also correct global overconfidence or underconfidence. Reestimating every coefficient is more flexible but requires more information and may add variance when current outcomes are scarce.^[17]^ Guidance on external validation sample size and learning curves likewise emphasizes events, effective information, and model complexity rather than nominal sample size alone.^[18,19]^ Empirical comparisons of these update options under controlled current outcome budgets remain uncommon in adolescent suicide screening.

We examined 1 frozen screening score across 5 independent United States (US) national Youth Risk Behavior Survey (YRBS) waves. Four research questions were prespecified. Research Question 1 asked whether discrimination and calibration of a 2015 score remained stable through 2023. Research Question 2 asked whether a model from the immediately preceding wave reliably improved transport. Research Question 3 asked how update strategy interacted with the number of newly labeled records from the target survey. Research Question 4 asked whether routine updating added benefit or variability when the static score was already adequately calibrated. The objective was not to create a clinical diagnostic tool or claim future individual prediction. It was to quantify the information and complexity tradeoff involved in maintaining a contemporaneous population screening score.

## 2. Materials and methods

### 2.1. Study design and data source

We conducted a secondary observational analysis of public national YRBS data from 2015, 2017, 2019, 2021, and 2023. Each wave is a separate cross section of students in grades 9 through 12 attending US public and private schools. The national survey uses a stratified, multistage cluster design and supplies weights, strata, and primary sampling units (PSUs).^[20]^ The waves were not linked at the student level. The 2021 field period occurred in the fall rather than the usual spring. The 2023 survey returned to spring fielding, changed from paper questionnaires to tablets, added an American Indian or Alaska Native supplemental sample, and revised parts of the questionnaire.^[20]^ Temporal contrasts therefore combine population, context, field period, sampling, and administration changes.

The national overall response rate was 60.0% in 2015, 60.0% in 2017, 60.3% in 2019, 57.5% in 2021, and 35.4% in 2023.^[20]^ These rates describe participation in the sampled surveys and are distinct from item availability among records in the public files. The Centers for Disease Control and Prevention weights adjust for school and student nonresponse and oversampling, but weighting cannot ensure that respondents and nonrespondents have identical distributions of unmeasured risk. The lower 2023 response rate therefore remains a possible source of selection and temporal comparability bias.

All 81,401 sampled records in the 5 public files were assessed for eligibility. The analysis population comprised students with an observed answer to the item about suicide attempts during the past year. We did not impute the outcome because the prespecified screening estimand concerned observed outcomes. Exclusion of 12,719 records without this answer left 68,682 records. This complete outcome analysis estimates performance among respondents with recorded attempt status; it does not recover performance for students who did not participate or whose attempt response was unavailable. Missing predictor answers remained explicit categories. Study size was determined by all eligible records in the selected surveys. Update budgets were fixed experimental conditions, not samples selected after inspecting performance.

The prespecified protocol and amendment record are available (see Supplemental Digital Content 2, Supplemental Digital Content 1). The wave-level eligibility and event summary is provided in Supplemental Digital Content 13, Supplemental Digital Content 2.

### 2.2. Outcome and predictor harmonization

The outcome was at least 1 suicide attempt reported during the preceding 12 months. Zero attempts were coded 0 and 1 or more attempts were coded 1. The item records recalled behavior rather than behavioral intention, but it was not corroborated by clinical records. The outcome is therefore contemporaneous reported status, not an event observed after predictor measurement.

Fourteen categorical predictor concepts had comparable meaning in every questionnaire (Table 1). Grade retained the 4 numbered grades, an ungraded or other category, and a missing category. The remaining concepts were collapsed to yes, no, and missing. For frequency items, no denoted 0 occurrences, and yes denoted 1 or more, except that no physical activity denoted 0 active days, and short sleep denoted fewer than 8 hours on an average school night. The item mapping was fixed from the original questionnaires before target performance was evaluated. Item numbers shifted between waves, but the retained meanings and coding rules did not. The fall timing in 2021 and tablet administration in 2023 were treated as contextual and measurement shifts to be captured by temporal validation; they were not statistically adjusted away.

**Table 1:** Predictor harmonization.

Predictor	Coding	2015	2017	2019	2021	2023
Grade	9, 10, 11, 12, other or ungraded, missing	Q3	Q3	Q3	Q3	Q3
Safety absence	0 d, at least 1 d, missing	Q16	Q15	Q15	Q14	Q14
Weapon threat	0 times, at least 1 time, missing	Q17	Q16	Q16	Q15	Q15
School fight	0 times, at least 1 time, missing	Q20	Q18	Q18	Q17	Q17
Forced sexual intercourse	No, yes, missing	Q21	Q19	Q19	Q19	Q19
School bullying	No, yes, missing	Q24	Q23	Q23	Q23	Q24
Electronic bullying	No, yes, missing	Q25	Q24	Q24	Q24	Q25
Sad or hopeless	No, yes, missing	Q26	Q25	Q25	Q25	Q26
Current cigarette use	0 days, at least 1 d, missing	Q33	Q32	Q32	Q32	Q33
Current vaping	0 days, at least 1 d, missing	Q40	Q35	Q35	Q35	Q36
Current alcohol use	0 days, at least 1 d, missing	Q43	Q42	Q41	Q41	Q42
Current marijuana use	0 times, at least 1 time, missing	Q49	Q48	Q47	Q47	Q48
No physical activity	0 active d, at least 1 active d, missing	Q80	Q79	Q78	Q77	Q76
Short sleep	< 8 hrs, at least 8 hrs, missing	Q88	Q88	Q88	Q86	Q85

Items are questionnaire item numbers in each national survey. Missing predictor responses were retained as explicit categories. The 2021 fall field period and 2023 tablet administration and supplemental sampling were treated as temporal context and measurement changes, not adjusted away.

The predictor set was selected for temporal harmonization and screening score maintenance, not for estimation of an exposure effect. A retrospective multicenter infection study, for example, used multivariable models to identify factors associated with treatment efficacy and mortality.^[21]^ That is a different estimand. No variable in the present study was designated as an exposure, ridge coefficients represent predictive associations, and the 14 items are not a comprehensive confounder set. Age, education, income, digital literacy, health status, cognitive function, prior technology use, and urban or rural residence were therefore neither assumed nor claimed to have been causally controlled. Some do not apply to this adolescent school survey, and others were unavailable with comparable wording in all 5 waves.

The study did not measure perceived usefulness, perceived ease of use, technology acceptance, or another latent acceptance construct. It therefore did not invoke the Technology Acceptance Model or specify a structural equation model. Its conceptual framework is predictive measurement maintenance: whether the same score retains discrimination and calibration across later populations and how much current outcome information is needed to update it.

Sex, race and ethnicity, and sexual identity were not model inputs. This restriction avoided using sensitive identity as an individual score component and kept categories comparable across waves, but it also removed attributes strongly associated with attempt prevalence.^[22]^ The score therefore cannot support identity-stratified surveillance or subgroup calibration audits, and its practical utility may be lower where such stratification is essential. Suicidal ideation, suicide plan, attempt frequency, and injury or treatment after an attempt were excluded to prevent direct target leakage. No predictor was selected because it performed favorably in a target survey.

The executable harmonization code is available in Supplemental Digital Content 4, Supplemental Digital Content 3, together with the complete cross-wave item ledger in Supplemental Digital Content 12, Supplemental Digital Content 4.

### 2.3. Source score development

The source score was a ridge logistic regression weighted for the survey design, with indicator encoding of the 14 predictor concepts. The inverse regularization parameter was selected from prespecified candidates through 5-fold 2015 cross-validation in which survey design groups defined by stratum and PSU remained intact. The selected value, *C* = 0.1, was frozen. Cross-fitted 2015 predictions estimated source performance and the weighted 90th percentile threshold. A final source model was fitted to all eligible 2015 records and applied to later surveys without target tuning.

Weights were normalized to each fitted sample size so that the penalty retained a comparable scale, while original survey weights were used for performance estimation. As a recency comparison, the same specification was separately fitted in 2017, 2019, and 2021 and applied to the immediately following wave. A sensitivity analysis repeated source development with *C* values of 0.01, 0.1, 1, and 10. Smaller *C* means stronger shrinkage.

The frozen score artifact and its generation code are available in Supplemental Digital Content 5, Supplemental Digital Content 5, and the frozen coefficient and intercept estimates are available in Supplemental Digital Content 14, Supplemental Digital Content 6.

### 2.4. Updating experiment

In each later survey, we compared the frozen score, intercept recalibration, intercept and slope recalibration, and full refitting of the same ridge specification using 250, 500, 1000, 2000, or 4000 target labels in 50 repeated 5-fold evaluations that kept PSUs separate between updating and evaluation.

Intercept recalibration estimated 1 target offset while holding the frozen linear predictor fixed. Intercept and slope recalibration estimated an offset and 1 calibration slope. Refitting reestimated the complete ridge model with the same predictors and *C* = 0.1. No target-specific predictor search, algorithm selection, or hyperparameter tuning was allowed.

Within every target survey and each of 50 fixed random seeds, 5 outer folds were generated by stratified group splitting. A PSU contributed either to an update partition or to its corresponding evaluation fold, never both. From the 4 outer training folds, records were sampled without reference to outcome status and in proportion to the survey strata. Every strategy received the same update records. Predictions from the 5 reserved folds were combined into 1 vector covering the target survey. The attempt count, PSU count, stratum count, and weighted effective sample size of each update sample were recorded. The complete outer training portion was an additional benchmark.

Proportional, outcome-blind acquisition was chosen to emulate routine labeling of a probability sample and preserve an interpretable record budget. An event-enriched design could provide more attempts for coefficient updating but would require outcome ascertainment before selection and inverse probability weighting to recover population calibration. It would answer a different cost question and was not added after the primary results were known.

The executable learning curve implementation is available in Supplemental Digital Content 8, Supplemental Digital Content 7.

### 2.5. Performance measures and statistical analysis

Primary measures were absolute calibration intercept error, absolute calibration slope error, and Brier score weighted for the survey design. Secondary measures were AUROC, area under the precision-recall curve (AUPRC), weighted log loss, the observed-to-expected risk ratio, and sensitivity and positive predictive value when exactly the highest scoring 10% of the weighted population was selected. AUPRC was included because attempts were uncommon.^[23]^ The 10% analysis represents fixed capacity, not a recommended referral policy. Decision curve analysis was not conducted because YRBS supplies neither a clinically justified probability threshold nor a relative cost for classification errors.

For a descriptive secondary summary, a run was called closely calibrated when the absolute intercept was no > 0.10 and the slope was 0.90 to 1.10. This rule was fixed before the update experiment but is not clinically validated. With the slope fixed at 1, an intercept of plus or minus 0.10 would move a predicted probability of .10 to .109 or .091 and a probability of .20 to .216 or .184. Even shifts of this size could alter referrals under a probability threshold, while intercept recalibration would not change ranking under fixed capacity. Continuous calibration measures remained primary.

Frozen estimates for each complete survey received 95% confidence intervals (CIs) from 500 Rao–Wu stratified PSU bootstrap replicates.^[24]^ After outcome restriction, 1 2017 stratum and 3 2019 strata contained 1 PSU. The primary bootstrap treated them as certainty strata with a multiplier of 1. Sensitivity analyses merged each with the nearest stratum having at least 2 PSUs or omitted singleton strata. The 2023 survey had no singleton strata.

The update experiment was summarized by the median and 5th and 95th percentiles across 50 repetitions. These percentiles describe update sample and fold instability, not population CIs. Paired differences compared strategies within the same wave, repetition, and budget. We added bootstrap CIs for the median paired difference using 20,000 draws, direction consistency, and a 2-sided Wilcoxon signed-rank test. These are algorithmic resampling diagnostics and do not treat repetitions as independent participants. Refit penalty sensitivity at 250 and 1000 labels used *C* values of 0.01, 0.1, 1, and 10 on the same splits and update samples.

Supporting tables and figures are provided in Supplemental Digital Content 1, Supplemental Digital Content 8. The bootstrap implementation is available in Supplemental Digital Content 7, Supplemental Digital Content 9, and the paired strategy comparison data are available in Supplemental Digital Content 19, Supplemental Digital Content 10.

### 2.6. Drift decomposition

We used a descriptive Oaxaca-style decomposition on the probability scale. Let πs and πt denote weighted observed prevalence in 2015 and target wave t. Let ms(X) denote the frozen source model and mt(X) a target local ridge model with *C* = 0.1. The total prevalence shift was πt − πs. The predictor distribution component was Et[ms(X)] − Es[ms(X)]. The conditional model component was Et[mt(X)] − Et[ms(X)]. The residual component was {πt − Et[mt(X)]} − {πs − Es[ms(X)]}. The 3 components reconstruct the total by identity. We repeated the decomposition on the log odds scale. It is descriptive rather than causal, and components can oppose each other, so their shares may be negative or > 100%.

The complete probability scale and log odds decomposition output is available in Supplemental Digital Content 16, Supplemental Digital Content 11.

### 2.7. Bias control, reporting, and reproducibility

Optimistic bias was limited by freezing the source specification before temporal validation, excluding direct suicide items, preventing target labels from influencing predictor or hyperparameter selection, separating PSUs between updating and evaluation, and retaining every prespecified wave, budget, repetition, and strategy. Analyses used Python 3 and scikit-learn.^[25]^ Reporting followed the Strengthening the Reporting of Observational Studies in Epidemiology statement and the Transparent Reporting of a multivariable prediction model for Individual Prognosis Or Diagnosis and Artificial Intelligence guidance, where applicable.^[26,27]^ Key eligibility, variable, missing data, sampling, performance, uncertainty, and validation details are reported in the Methods and Results sections.

Code developed with artificial intelligence assistance was reviewed and executed locally by the authors. A separate verification script recomputed eligible counts and events, metric identities, decomposition reconstruction, and paired summaries from structured outputs. The revision analysis also reproduced every primary *C* = 0.1 frozen metric to within 2 × 10 − 10 and exactly reproduced the original *C* = 0.1 update runs at 250 and 1000 labels. The full analysis was not independently rewritten in a second programming language; this limitation is stated to distinguish verification from complete independent reimplementation.

The original national YRBS protocol was approved by institutional review boards at the US Centers for Disease Control and Prevention and ICF International (formerly ICF Macro, Inc.), the survey contractor. Survey procedures protected privacy through anonymous participation. Participation was voluntary and local parental permission procedures were followed.^[20]^ This secondary analysis used only public, deidentified records, involved no participant contact, and therefore required no additional review or consent. Source data, code, the harmonization ledger, the protocol, amendments, and aggregate outputs are described in the Data availability section.

The reusable analysis utilities are available in Supplemental Digital Content 3, Supplemental Digital Content 12. The revision analysis and verification files are available in Supplemental Digital Content 6, Supplemental Digital Content 13, Supplemental Digital Content 9, Supplemental Digital Content 14, Supplemental Digital Content 10, Supplemental Digital Content 15, Supplemental Digital Content 11, Supplemental Digital Content 16, and Supplemental Digital Content 20, Supplemental Digital Content 17.

## 3. Results

### 3.1. Participants

Across 5 surveys, 81,401 students were sampled, and 68,682 had an observed attempt response. The unavailable count decreased from 3057 of 15,624 sampled students in 2015 to 767 of 20,103 in 2023 (Table 2). Among analyzed records, 6855 students reported at least 1 attempt during the past year. Each survey contributed 10,520 to 19,336 analyzed records. Weighted prevalence was lowest in 2017 at 7.4% (95% CI, 6.5–8.2%) and highest in 2021 at 10.2% (95% CI, 9.3–11.0%).

**Table 2:** Temporal performance.

Year	Sampled, n	Outcome unavailable, n	Analyzed, n	Attempts, n	Weighted prevalence, % (95% CI)	AUROC (95% CI)	Brier score (95% CI)	Calibration intercept (95% CI)	Calibration slope (95% CI)
2015	15,624	3057	12,567	1203	8.6 (7.6–9.5)	0.859 (0.843–0.878)	0.064 (0.057–0.070)	−0.033 (−0.140–0.071)	0.967 (0.894–1.048)
2017	14,765	4079	10,686	837	7.4 (6.5–8.2)	0.860 (0.841–0.879)	0.056 (0.050–0.061)	−0.266 (−0.415–−0.143)	0.996 (0.911–1.072)
2019	13,677	3157	10,520	1067	8.9 (8.0–9.9)	0.856 (0.831–0.880)	0.066 (0.059–0.073)	−0.115 (−0.221–−0.015)	0.993 (0.878–1.115)
2021	17,232	1659	15,573	1753	10.2 (9.3––11.0)	0.852 (0.835–0.869)	0.072 (0.066–0.077)	0.030 (−0.090–0.128)	1.013 (0.942–1.098)
2023	20,103	767	19,336	1995	9.5 (8.4–10.5)	0.860 (0.845–0.875)	0.068 (0.061–0.074)	−0.148 (−0.285–−0.019)	1.009 (0.943–1.088)

Intervals are percentile intervals from 500 Rao–Wu stratified primary sampling unit bootstrap replicates.

AUROC = area under the receiver operating characteristic curve, CI = confidence interval.

The independently tabulated wave counts and missing outcome totals are reported in Supplemental Digital Content 13, Supplemental Digital Content 2.

### 3.2. Research Question 1: temporal stability

**Figure 1.**
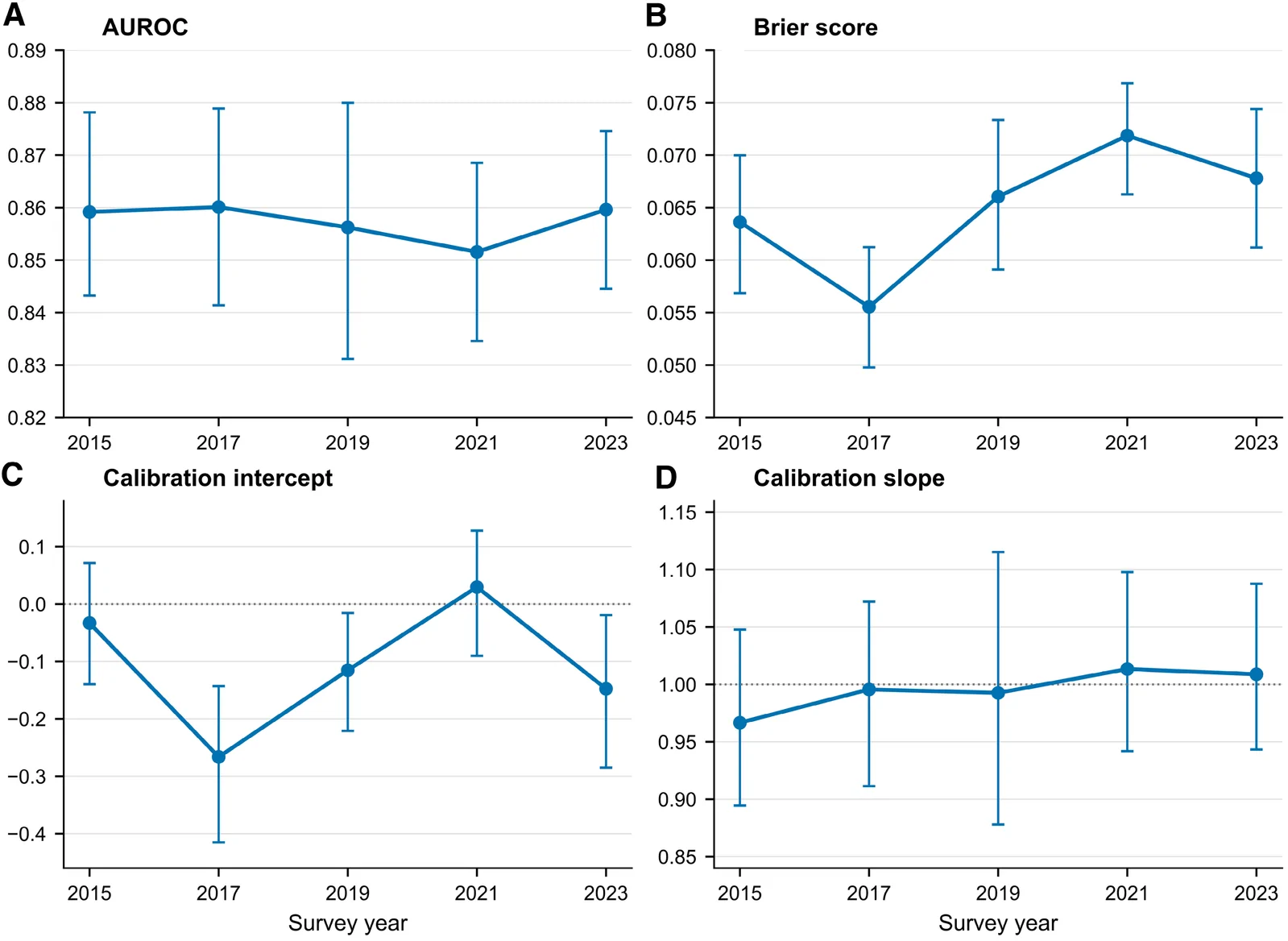
Temporal performance. (A) AUROC, (B) brier score, (C) calibration intercept, and (D) calibration slope of the frozen 2015 score applied to each national survey from 2015 through 2023. Points are survey-weighted estimates, and error bars are 95% CIs from 500 stratified survey bootstrap replicates. Dashed reference lines show the ideal calibration intercept of 0 in panel C and a calibration slope of 1 in panel D. AUROC = area under the receiver operating characteristic curve, CI = confidence interval,

For Research Question 1, discrimination and score spread were stable, but average predicted risk was not. Cross-fitted 2015 predictions yielded an AUROC of 0.859 (95% CI, 0.843–0.878). Transported AUROC remained 0.852 to 0.860 and calibration slopes remained 0.993 to 1.013 (Table 2; Fig. 1). Calibration intercepts were −0.266 (95% CI, −0.415–−0.143) in 2017, −0.115 (95% CI, −0.221–−0.015) in 2019, 0.030 (95% CI, −0.090–0.128) in 2021, and −0.148 (95% CI, −0.285–−0.019) in 2023. Negative values indicate overestimation. Observed-to-expected ratios were 0.825, 0.921, 1.021, and 0.902.

The decomposition showed why prevalence change alone was insufficient as an explanation. Relative to 2015 prevalence of 0.0856, total risk differences were −0.0119, 0.0034, 0.0161, and 0.0090. Changes in predictor distributions increased mean source model risk by 0.0037, 0.0110, 0.0139, and 0.0192, respectively. Conditional model components were −0.0155, −0.0075, 0.0021, and −0.0101. Thus, predictor and conditional components opposed each other in 2017, 2019, and 2023. In 2021 they acted in the same direction, and the predictor component represented 86.8% of the total shift.

Source penalty sensitivity did not change the broad discrimination finding. Across *C* values of 0.01 to 10, target AUROC remained similar, although *C* = 0.01 produced calibration slopes from 1.162 to 1.204, consistent with excessive shrinkage. The selected *C* = 0.1 provided the best balance of discrimination and calibration.

Singleton PSU handling did not explain the primary calibration findings. Under certainty treatment, the 95% CI for the intercept in 2017 was −0.415 to −0.143, identical to the primary bootstrap in Table 2; it was −0.413 to −0.141 after nearest stratum merging and −0.425 to −0.144 after omission. Corresponding 2019 intervals were −0.221 to −0.015 under certainty treatment, −0.219 to −0.018 after merging, and −0.241 to −0.017 after omission. All supported overestimation. There were no singleton strata in 2023, so its interval width arose from other design and outcome information.

The frozen score estimates used for the transported predictions are available in Supplemental Digital Content 14, Supplemental Digital Content 6.

The complete penalty sensitivity output is available in Supplemental Digital Content 17, Supplemental Digital Content 18. The single PSU sensitivity output is available in Supplemental Digital Content 18, Supplemental Digital Content 19.

### 3.3. Research Question 2: model recency

For Research Question 2, replacing the source with the immediately preceding survey did not reliably improve transport. For 2017 to 2019, 2019 to 2021, and 2021 to 2023 transport, AUROCs were 0.854, 0.854, and 0.865; calibration intercepts were 0.044, 0.134, and −0.157; and slopes were 0.975, 1.037, and 1.004. Only the 2017 to 2019 model met the descriptive close calibration rule.

### 3.4. Research Question 3: update strategy and information

**Figure 2.**
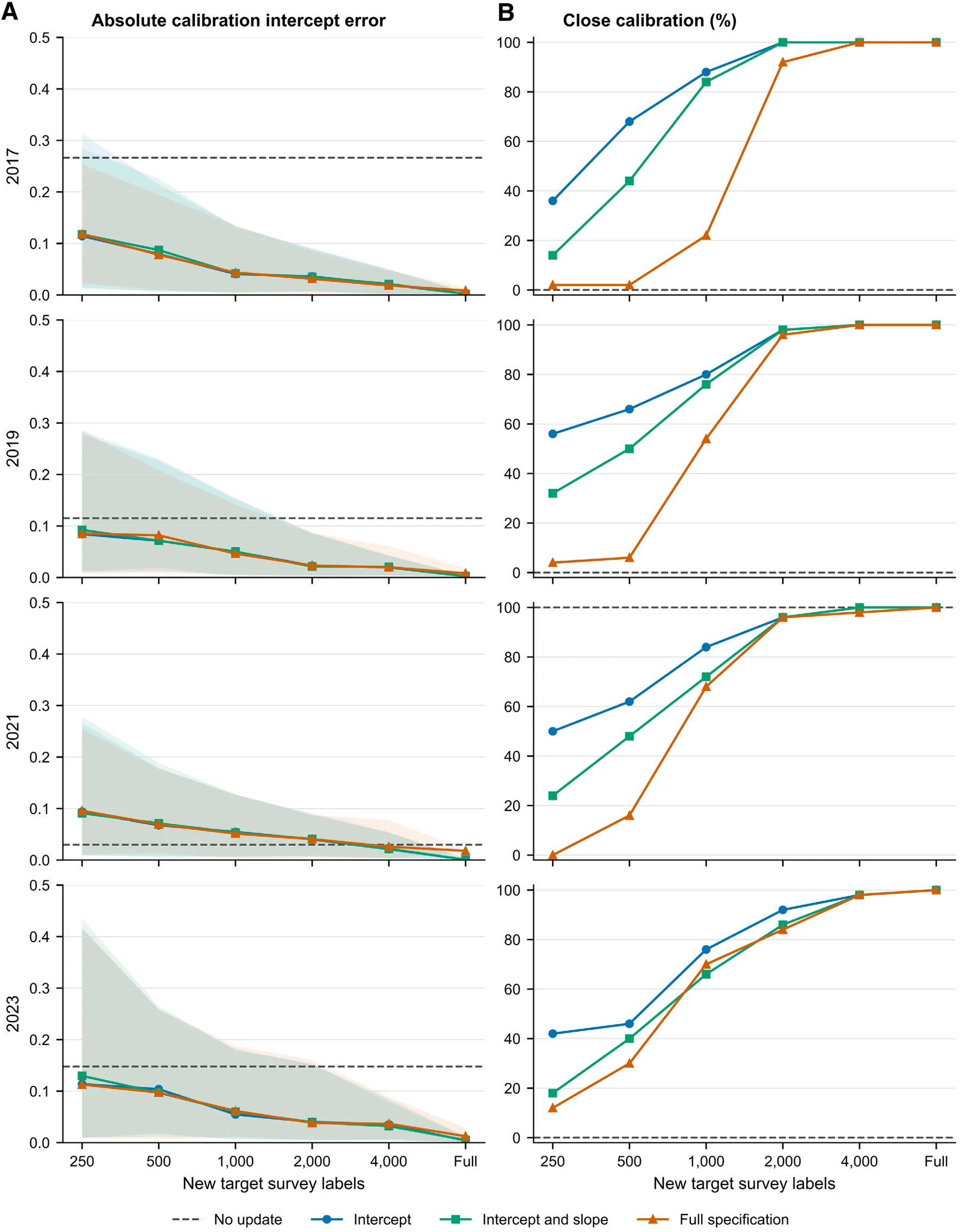
Updating calibration. (A) Absolute calibration intercept error and (B) percentage of evaluations meeting the descriptive close calibration rule (absolute intercept no > 0.10 and slope from 0.90 to 1.10) for the static score, intercept recalibration, intercept and slope recalibration, and full refitting at 250 to 4000 target labels. Each row shows 1 target survey (2017, 2019, 2021, or 2023). Lines show medians, and shaded bands show 5th to 95th percentiles across 50 repeated evaluations that kept primary sampling units separate; dashed lines indicate the static score.

For Research Question 3, update complexity and target information interacted strongly (Fig. 2). At 250 labels, same-specification refitting reduced median AUROC relative to the static score by 0.040 in 2017, 0.043 in 2019, 0.036 in 2021, and 0.041 in 2023. Median Brier score increased by 0.0047, 0.0052, 0.0054, and 0.0060. The median update sample contained only 19 to 27 attempts.

In paired comparisons at 250 labels, intercept recalibration had a lower Brier score and higher AUROC than refitting in all 50 repetitions of every target survey. Median Brier differences for intercept recalibration minus refitting ranged from −0.0050 to −0.0044, and median AUROC differences ranged from 0.0284 to 0.0376. Every bootstrap interval excluded 0, and every signed-rank *P* value was < .001. At 1000 labels, the Brier advantage occurred in 98 to 100% of repetitions and the AUROC advantage in 96 to 100%; median differences ranged from −0.0022 to −0.0015 for Brier score and 0.0072 to 0.0135 for AUROC.

Stronger shrinkage did not rescue refitting with 250 labels. Refit *C* = 0.1 generally produced a lower Brier score and equal or better AUROC than *C* = 0.01, 1, or 10 at 250 and 1000 labels. Weak shrinkage with *C* = 10 yielded nonfinite calibration estimates in 36 to 98% of the 250 label repetitions across target surveys. This sensitivity analysis does not exclude a benefit from a carefully specified Bayesian update, which was not tested.

With 1000 labels, intercept recalibration produced median absolute intercept errors from 0.041 to 0.055 and met the descriptive calibration rule in 88%, 80%, 84%, and 76% of repetitions for 2017, 2019, 2021, and 2023 (Table 3). Rates for intercept and slope recalibration were 84%, 76%, 72%, and 66%; rates for refitting were 22%, 54%, 68%, and 70%. Median AUROC after intercept updating ranged from 0.850 to 0.859, compared with 0.842 to 0.847 after refitting. Update samples contained medians of 79 to 114 attempts, but median weighted effective sample sizes ranged from 383 to 639.

**Table 3:** Updating performance.

Year	Strategy	Median attempts in update sample	Absolute intercept error, median (5th, 95th)	Absolute slope error, median (5th, 95th)	Close calibration, %	Brier score, median (5th, 95th)	AUROC, median (5th, 95th)
2017	Intercept	79	0.041 (0.005, 0.134)	0.012 (0.004, 0.038)	88	0.055 (0.055, 0.056)	0.859 (0.856, 0.861)
2017	Intercept and slope	79	0.042 (0.005, 0.131)	0.033 (0.002, 0.095)	84	0.055 (0.055, 0.056)	0.857 (0.854, 0.860)
2017	Full specification	79	0.043 (0.003, 0.135)	0.117 (0.036, 0.169)	22	0.057 (0.056, 0.058)	0.847 (0.841, 0.854)
2019	Intercept	101	0.050 (0.006, 0.154)	0.018 (0.009, 0.042)	80	0.066 (0.066, 0.067)	0.855 (0.851, 0.857)
2019	Intercept and slope	101	0.050 (0.002, 0.152)	0.035 (0.005, 0.094)	76	0.066 (0.066, 0.067)	0.854 (0.849, 0.857)
2019	Full specification	101	0.046 (0.005, 0.142)	0.080 (0.037, 0.151)	54	0.068 (0.067, 0.069)	0.842 (0.834, 0.849)
2021	Intercept	114	0.055 (0.006, 0.126)	0.008 (0.001, 0.019)	84	0.072 (0.072, 0.073)	0.850 (0.848, 0.852)
2021	Intercept and slope	114	0.053 (0.007, 0.127)	0.049 (0.006, 0.117)	72	0.072 (0.072, 0.073)	0.848 (0.844, 0.851)
2021	Full specification	114	0.051 (0.004, 0.128)	0.053 (0.006, 0.149)	68	0.074 (0.073, 0.075)	0.843 (0.838, 0.848)
2023	Intercept	103	0.055 (0.006, 0.182)	0.011 (0.002, 0.037)	76	0.068 (0.068, 0.069)	0.858 (0.854, 0.860)
2023	Intercept and slope	103	0.060 (0.008, 0.179)	0.048 (0.008, 0.120)	66	0.068 (0.068, 0.069)	0.856 (0.852, 0.859)
2023	Full specification	103	0.062 (0.011, 0.188)	0.026 (0.005, 0.094)	70	0.070 (0.069, 0.071)	0.844 (0.834, 0.851)

Values summarize 50 repeated 5-fold primary sampling unit separated evaluations. Close calibration denotes an absolute intercept no. > 0.10 and a slope from 0.90 to 1.10.

AUROC = area under the receiver operating characteristic curve.

With 2000 labels, intercept recalibration met the descriptive rule in 100%, 98%, 96%, and 92% of repetitions. Median event counts ranged from 157 to 225. With 4000 labels, the corresponding rates were 100%, 100%, 100%, and 98%. These are empirical learning curve results for this score and these surveys, not universal sample size thresholds.

The complete learning curve summary is provided in Supplemental Digital Content 15, Supplemental Digital Content 20.

### 3.5. Research Question 4: updating an adequate score

For Research Question 4, routine updating added variability when there was no established calibration problem. In 2021 the static score already met the descriptive rule, with an intercept of 0.030 and a slope of 1.013. Updating with small samples widened the distribution of calibration error without improving ranking. Intercept-based methods left the highest scoring 10% essentially unchanged, while full refitting altered ranking and was most variable at the smallest budgets.

## 4. Discussion

### 4.1. Principal findings

The 4 research questions produced 4 direct answers. First, the 2015 score retained discrimination through 2023, but its absolute probabilities were not stable in 3 of 4 target surveys. Second, a model from the immediately preceding survey did not reliably improve calibration. Third, intercept recalibration was more stable than full coefficient refitting when current outcomes were limited, and this ordering was consistent across paired repetitions and ridge penalties. Fourth, when the static score was already calibrated in 2021, automatic updating mainly added sampling variability.

The decomposition qualifies a simple prevalence shift narrative. Predictor distributions would have increased expected risk in every later survey, but conditional model components offset that increase in 3 surveys. Calibration slopes near 1 nevertheless indicate that the transported score preserved its global spread. Together, these findings support an intercept as the first repair for the observed global location error, while also showing that the origin of temporal change was not a single causal process. Survey timing, administration, sampling, context, and unmeasured factors remain inseparable.

### 4.2. Relation to previous research

The findings align with large health record studies in 1 respect and differ in another. Penfold et al and Simon et al found little advantage from redevelopment when existing models remained stable, and Walker et al found that large temporal changes in coding and practice did not necessarily destroy attempt model performance.^[11,12,14]^ Walsh et al demonstrated that important miscalibration can occur during prospective use and can respond to a parsimonious calibration update.^[13]^ Zang et al showed that transport may improve or deteriorate across clinical sources, depending on the setting and method.^[15]^ Our survey results likewise reject a universal assumption of either inevitable decay or automatic stability.

This study adds a controlled information dimension. The predictor set, penalty, and source score were fixed; 4 successive national populations were evaluated; older and immediately preceding sources were compared; and every update used a specified number of current records with evaluation in separate PSUs. The main contribution is therefore not a new algorithm or a high AUROC. It is evidence that maintenance performance depends jointly on the type of drift, update complexity, and effective target information. A recent score and a flexible refit can both perform worse when their additional parameters are not supported by sufficient events.

The distinction between a reported measure and a future observed behavior also appears in adjacent digital behavior research. Online review content varies with anonymity and temporal distance, and gaming burnout can coexist with continued play despite a stated intention to quit.^[28,29]^ These studies are not evidence about suicide risk. They instead reinforce why the YRBS endpoint must be named precisely: this study classified a recalled past year attempt, not an intention and not a prospectively observed act.

Adaptation methods are likewise specific to their data and target. Recent work on multimodal sentiment analysis reconstructs information under missing modalities, while unsupervised image domain adaptation aligns source and target representations without target labels.^[30,31]^ Those settings differ from a weighted health survey with structured predictors and observed target outcomes. We therefore compared simple probability recalibration with coefficient refitting rather than importing architectures designed for multimodal or image data. Across these settings, the common methodological lesson is that an update strategy must match the form of shift and the information available in the target population.

### 4.3. Operational guidance

For a surveillance team considering reuse of a fixed score, we suggest an audit at every new biennial YRBS wave and after any change in field timing, instrument, sampling, coding, or administration mode. A reserved current sample should be used to estimate observed-to-expected risk, calibration intercept and slope with CIs, Brier score, AUROC, and AUPRC. The acceptable amount of error should be prespecified from the intended action and referral capacity, not borrowed from our descriptive rule.

If the calibration intercept departs from zero while the slope remains near 1 and discrimination is preserved, an intercept update is the least complex repair. If slope, Brier score, or discrimination also deteriorates, partial coefficient updating or refitting becomes more plausible, but it requires adequate current events and a second reserved evaluation sample. A statistical alarm such as a calibration intercept CI excluding zero can prompt review but should not alone determine deployment because a large sample can detect an operationally trivial error. Conversely, a wide CI is evidence that more validation information is needed.

For this exact score, 2000 target records contained median event counts of 157 to 225 and allowed intercept recalibration to meet the descriptive rule in 92 to 100% of repetitions. This is a useful planning benchmark for a future YRBS audit, not a general minimum. Event count, design effect, effective sample size, degrees of freedom, and intended decisions must be considered.^[18,19]^ Outcome-enriched acquisition could make coefficient updating more efficient, but only if selection probabilities are recorded and population calibration is restored through appropriate weighting. Any such design should compare monetary and operational costs per ascertained event rather than treating all labels as equally costly.

### 4.4. Ethical and practical scope

The score deliberately omits direct suicide questions, but omission does not make indirect screening a sufficient safety procedure. Any operational evaluation would still require confidential, developmentally appropriate questions about current suicidal thoughts, plans, intent, access to means, and prior behavior, followed by a defined safety assessment and referral pathway. Current pediatric guidance treats a positive screen as the beginning of further assessment, not a diagnosis or final disposition.^[32]^ A descriptive rapid response unit study of 24 referred adolescents provides 1 concrete service model in which risk identification was linked to frequent multidisciplinary contact and continued therapeutic engagement, although its size and design do not establish a universal care pathway.^[33]^ A school or health system should not deploy this score unless trained personnel, privacy protection, timely review, crisis response, and referral resources are in place. A concerning direct disclosure or clinical judgment must override an indirect score.

Excluding identity attributes also has a cost. Sex, race and ethnicity, and sexual identity are associated with marked differences in reported attempt prevalence.^[22]^ Their exclusion means that the score cannot estimate identity-specific baseline risk, assess subgroup calibration, or guide stratified surveillance. A future implementation should evaluate performance by relevant groups even if identity is not used to calculate the individual score. The present design makes no algorithmic fairness claim.

### 4.5. Strengths and limitations

Strengths include 5 independent national samples, questionnaire-based harmonization, a frozen source specification, exclusion of direct target proxies, survey-weighted evaluation, PSU separation between updating and evaluation, identical samples for paired strategy comparisons, explicit learning curves, formal drift decomposition, penalty and variance sensitivities, and numerical verification. Every prespecified wave, budget, repetition, and strategy was retained, including findings unfavorable to refitting.

Several limitations remain. First, YRBS consists of repeated cross sections. Predictors and outcome cover overlapping periods, so the score classifies contemporaneous reported status rather than predicting a future individual event, tracing within-person change, or estimating causation. Second, survey participation and outcome availability introduce separate selection concerns. The supplied weights mitigate observed school and student nonresponse but cannot remove all bias from voluntary nonparticipation, and restriction to recorded attempt responses does not recover outcomes for item nonrespondents. Third, all predictors and the attempt outcome were self-reported. Recall error, variation in item interpretation, social desirability, and nondisclosure may affect both prevalence and apparent model performance, and no clinical record was available for corroboration. Fourth, the national sampling frame represents students in grades 9 through 12 enrolled in participating US public and private schools. It does not represent adolescents who are not attending school, students outside those grades, other countries, clinical populations, or a single local school system.

Fifth, the 14 predictors were selected for stable measurement across waves rather than comprehensive causal adjustment. The study did not include a harmonized measure of household income, digital literacy, general health, cognitive function, prior technology experience, or urban or rural residence. Related research among adolescents with depression has linked childhood abuse, peer victimization, and psychological resilience with nonsuicidal self-injury, but nonsuicidal self-injury is distinct from suicide attempt, and those constructs were not all available across the 5 YRBS waves.^[34]^ Unmeasured personal, family, school, clinical, and digital factors may therefore influence both reported risk and temporal drift. Sixth, temporal differences may reflect context, timing, administration, sampling, or measurement and cannot be attributed to the coronavirus disease 2019 pandemic or another single cause.^[20]^ Seventh, the close calibration rule is descriptive rather than a validated decision threshold. Eighth, repetition percentiles and signed-rank results quantify algorithmic sampling stability rather than national population uncertainty. Ninth, proportional outcome-blind sampling is only one acquisition design. Tenth, stronger ridge penalties were studied, but hierarchical Bayesian updating was not. Eleventh, identity attributes were excluded, preventing subgroup performance assessment. Twelfth, no independent clinical or school cohort outside the national YRBS frame was available; transport beyond anonymous school surveys is untested, and clinical benefit has not been established.

The relation to a scientifically separate manuscript using the same public waves is reported in the Related work and data reuse statement. That statement defines the distinct estimands, analysis paths, results, and displays and reports a new narrative overlap audit.

## 5. Conclusions

Stable discrimination did not guarantee stable absolute risk when an adolescent suicide attempt screening score was carried across national survey waves. When error was mainly global and the calibration slope remained stable, intercept recalibration was more reliable than full refitting with limited current outcomes. A recent model was not automatically better, and routine updating could add variability. Model maintenance should begin with a reserved current sample, separate monitoring of calibration and discrimination, and the least complex update that addresses the observed drift. The present findings require validation outside YRBS before any clinical or school deployment.

## Author contributions

**Conceptualization:** Xiaotian Ma, Su Jia.

**Data curation:** Junjie Zhang.

**Formal analysis:** Junjie Zhang.

**Investigation:** Xiaotian Ma, Su Jia.

**Methodology:** Xiaotian Ma, Su Jia.

**Project administration:** Xiaotian Ma.

**Software:** Junjie Zhang.

**Validation:** Su Jia, Junjie Zhang.

**Visualization:** Junjie Zhang.

**Writing – original draft:** Xiaotian Ma, Su Jia.

**Writing – review & editing:** Su Jia, Junjie Zhang.








































