# Supplementary material for: Temporal maintenance of an adolescent suicide attempt screening score in national surveys: An observational study
Source: Medicine (Baltimore). 2026 Aug 28;105(35):e50513. doi: 10.1097/MD.0000000000050513 (PMC13529099; doi:10.1097/MD.0000000000050513)
Supplement: Supplementary file 1 [file medi-105-e50513-s001.docx]

**Supplemental Digital Content 2**

**Reproducibility protocol**

Temporal maintenance of an adolescent suicide attempt screening score in national surveys: An observational study

First author: Xiaotian Ma

This file records the prespecified protocol, amendments, optimization log, reproducibility guide, and software environment. Individual analysis and verification scripts and individual aggregate data files are supplied separately as Supplemental Digital Content 3 through 20.

# Analysis protocol for the independent SCI revision

## Protocol status

This protocol was written on 15 July 2026 after an overlap audit identified a redundant-publication risk between the existing SCI draft and a companion psychology manuscript. It was frozen before running the new update-budget analyses described below. The public YRBS data and earlier frozen-score results had already been inspected; this is therefore a documented post-result redesign, not a prospective preregistration. All claims about the new learning-curve experiment will be described as prespecified within the redesign, not as prospectively confirmatory.

## Provisional title

Data Requirements for Maintaining an Adolescent Suicide Attempt Screening Score Across Five National Surveys

## Scientific question

How do update strategy and the amount of newly observed target-wave data jointly affect the calibration, overall performance, and resource-constrained detection of a frozen adolescent suicide-attempt screening score?

## Independence from the companion manuscript

The companion manuscript estimates group disparities in four suicidal-thought-and-behavior outcomes under incomplete self-report. This SCI revision excludes group contrasts, missing-outcome standardization, bounds, pattern-mixture models, MNAR tipping points, subgroup calibration, and response-process interpretation. The only shared elements are the public survey source, harmonized predictor data, and the recorded suicide-attempt item. The related manuscript and shared-data relationship will be disclosed to the editor.

## Data and analytic population

- National Youth Risk Behavior Survey waves: 2015, 2017, 2019, 2021, and 2023.

- Repeated national cross-sections of students in grades 9 through 12; not a longitudinal cohort.

- SCI analytic population: records with an observed answer to the past-year suicide-attempt item.

- Expected analytic count from the frozen harmonization: 68,682 observed outcomes and 6,855 reported attempts.

- Survey weights are used in model fitting and performance estimation. Primary sampling units are kept intact between update and evaluation folds.

## Outcome and claim boundary

The outcome is at least one self-reported suicide attempt during the preceding 12 months. The study evaluates contemporaneous population risk stratification and transport of a screening score to later survey populations. It does not predict a student's future attempt, establish diagnosis, or estimate causal effects of historical events.

## Frozen score

The amended source score is a survey-weighted ridge logistic model developed among observed 2015 outcomes with 14 harmonized non-suicide predictor concepts. Sex, race and ethnicity, and sexual identity are excluded as direct model inputs because they are the substantive comparison dimensions in the companion manuscript and are not necessary to answer the maintenance question. Direct ideation, plan, attempt-count, and treated-injury items are also excluded. Grade and 13 behavioural or contextual concepts are retained. The penalty is selected only within grouped 2015 cross-validation and is then fixed. No target-wave label is used to retune the source model.

## Research questions

1. How do discrimination and calibration of the frozen 2015 score change in the observed-outcome populations of 2017, 2019, 2021, and 2023?

2. Does a model trained in the immediately preceding survey wave improve later-wave performance relative to retaining the 2015 score?

3. Within a new target wave, how do no update, intercept recalibration, logistic recalibration, and same-specification refitting compare when only 250, 500, 1,000, 2,000, or 4,000 newly observed outcomes are available?

4. How much split-to-split instability accompanies each update strategy, and when does added update complexity cease to provide a stable gain?

## Update strategies

All strategies use the same frozen 2015 score and the same update sample within a run.

1. Static: no update.

2. Intercept: estimate one target-wave intercept offset while retaining the source linear predictor.

3. Logistic: estimate a target-wave intercept and one calibration slope.

4. Refit: refit the same 14-predictor ridge logistic specification with C=0.1.

5. Full-data benchmark: apply each update strategy using the full outer-training portion of the target wave; this is a cross-fitted benchmark, not an untouched external validation.

No algorithm search, new predictor selection, subgroup thresholding, or hyperparameter tuning is allowed in the update experiment. Survey weights are normalized to the fitted sample size in every penalized fit so that the fixed ridge penalty has the same scale across source and target samples; original survey weights are used for performance estimation.

## Cluster-separated repeated evaluation

For each target wave and each of 50 fixed random seeds:

1. create five outer folds using stratified group splitting with the suicide-attempt outcome for balance and the survey primary sampling unit as the indivisible group;

2. hold one outer fold out for evaluation;

3. draw a simple, outcome-blind update sample of the required size from the other four folds, with proportional allocation across survey strata;

4. fit all update strategies on exactly that update sample;

5. predict the held-out primary sampling units;

6. combine the five held-out folds to obtain one complete cross-fitted target-wave prediction vector for each strategy and budget;

7. repeat for all 50 seeds.

This produces 50 full-wave, cluster-separated performance estimates for each target wave, update budget, and strategy. Students from an evaluation primary sampling unit never enter the corresponding update set.

## Update budgets and resource estimate

Budgets are 250, 500, 1,000, 2,000, and 4,000 newly observed outcomes in each outer-training sample. The full-data benchmark uses all eligible records in the outer-training folds. Actual event counts, primary sampling units, survey strata, and weighted effective sample sizes are recorded for every fitted update.

Planned fits:

- 4 target waves x 50 repetitions x 5 outer folds x 5 budgets x 3 fitted update strategies = 15,000 fitted updates;

- plus 3,000 full-data benchmark fits;

- static predictions require no fitting.

The ridge model benchmark indicates approximately 0.05 seconds per 4,000-record refit on the available CPU. Expected wall-clock time is 15 to 35 minutes, with no GPU or paid API use. Estimated result storage is below 100 MB because only metrics and diagnostics, not every prediction vector, will be retained.

## Metrics

Primary metrics:

- absolute calibration intercept error, |intercept|;

- absolute calibration slope error, |slope - 1|;

- survey-weighted Brier score.

Secondary metrics:

- AUROC;

- AUPRC;

- weighted log loss;

- observed-to-expected ratio;

- sensitivity and positive predictive value when exactly the highest predicted-risk 10% of the evaluation population are selected;

- sensitivity, positive predictive value, and referral fraction at the frozen 2015 score threshold.

The fixed-capacity analysis is preferred to decision-curve analysis because no clinically justified probability threshold or false-positive/false-negative utility ratio is available in YRBS. No net-benefit claim will be made without such a decision context.

## Descriptive calibration attainment target

For a transparent descriptive summary, a run is counted as attaining close calibration when both |intercept| <= 0.10 and 0.90 <= slope <= 1.10. This is a stringent reporting convention, not a universal clinical acceptability threshold. Continuous errors and uncertainty distributions remain primary.

## Statistical summaries

- Report the median, 5th percentile, and 95th percentile across the 50 repeated cluster-separated runs.

- Compare strategies within the same wave, repetition, and update budget using paired metric differences.

- Report the proportion of repetitions meeting the descriptive calibration target.

- Do not treat repetitions as independent students or use row-level significance tests.

- Use a design-based 500-replicate Rao-Wu bootstrap for frozen full-wave temporal performance. Analytically restricted strata containing one PSU are treated as certainty strata and reported. The repeated update experiment quantifies update-sample and fold instability rather than a population confidence interval.

- Emphasize effect magnitude and interval width rather than binary P values.

## Stopping and interpretation rules

- Retain all target waves and update budgets regardless of favorability.

- If full refitting performs worse at small budgets, report it as instability rather than remove it.

- If the static score is already well calibrated, do not manufacture a need for updating.

- If no single budget works in every wave, report a wave-specific empirical requirement and reject a universal minimum.

- Do not change the calibration target after seeing results.

- Any code or design change after the first full run must be logged with a scientific or computational reason.

# Analysis amendments

## Amendment 1: substantive redesign after companion-manuscript overlap audit

- Date: 15 July 2026.

- Trigger: a side-by-side audit showed that the existing SCI draft and a companion psychology manuscript both centered missing-outcome assumptions and population-group results.

- Ethical action: supersede the existing PMR package and redesign the SCI study around model maintenance and update-data efficiency.

- Removed from SCI: complete-case versus MAR/MNAR comparisons, outcome-observability analyses, auxiliary-variable ablation, ideation comparator, subgroup calibration, fairness metrics, and all group-disparity interpretation.

- Added to SCI: repeated cluster-separated update-budget learning curves, an immediately preceding-wave baseline, update-strategy instability, and fixed-resource operating characteristics.

- Rationale: scientific separation must occur in the research question, analyses, results, and conclusion; wording changes are insufficient.

- Data-informed status: prior frozen-score results were known, so the redesign is not presented as prospective preregistration. The new update-budget choices were frozen before computing their results.

## Amendment 2: calibration-attainment rate added to the summary export

- Date: 15 July 2026, after the first complete update-budget run.

- Trigger: the frozen protocol required the proportion of repetitions satisfying

the fixed descriptive calibration target, whereas the generic summary

function exported only the median and 5th/95th percentiles of its binary

indicator.

- Change: add the arithmetic mean of the unchanged binary indicator as

close_calibration_rate in the grouped summary.

- Not changed: analytic records, predictors, outcome, folds, random seeds,

update samples, update methods, budgets, continuous metrics, or the fixed

criterion (absolute intercept no greater than 0.10 and slope 0.90 to 1.10).

- Rationale: this is a reporting correction required by the frozen protocol;

no model was refitted and no result-dependent threshold was introduced.

## Amendment 3: protected identity inputs removed and penalized-fit weights standardized

- Date: 15 July 2026, after visual and scientific review of the first complete

maintenance run but before manuscript drafting.

- Trigger 1: sex, race and ethnicity, and sexual identity were the comparison

dimensions in the companion psychology manuscript. Retaining them as direct

inputs to the SCI score weakened substantive separation and would also invite

a fairness analysis that is outside this manuscript's independent question.

- Change 1: remove sex, race_ethnicity, and

sexual_identity_primary from the SCI score. The revised score contains

grade plus 13 behavioural and contextual predictor concepts and continues to

exclude every direct suicide item.

- Supporting audit: in a diagnostic temporal fit, the revised 14 predictor

score retained later wave AUROCs of 0.852 to 0.860, only 0.004 to 0.006 below

the 17 predictor specification. This check was used to reject a scientifically

unnecessary protected attribute input, not to select favorable results.

- Trigger 2: the first update experiment normalized survey weights for the 2015

penalized fit but passed unnormalized weights to target wave ridge refits.

Because sample weight scale changes the effective strength of penalization,

the comparison was not internally consistent.

- Change 2: normalize survey weights to the fitted sample size in every

penalized source, rolling source, partial update, and full refit. Survey

weights remain on their original scale for performance estimation.

- Consequence: the earlier maintenance run and all displays derived from it are

superseded. The complete experiment, verification, displays, and manuscript

must be regenerated from the amended specification.

- Not changed: outcome, observed outcome population, target waves, outer folds,

random seeds, budgets, recalibration methods, evaluation metrics, fixed

calibration criterion, or fixed capacity rule.

## Amendment 4: single PSU strata in the design bootstrap

- Date: 15 July 2026, during the independent SCI uncertainty analysis.

- Trigger: after restriction to records with an observed suicide-attempt item,

the 2017 analytic wave contained one stratum with a single PSU and the 2019

wave contained three. Standard Rao-Wu rescaling requires at least two PSUs

within a stratum.

- Change: retain each single-PSU stratum as a certainty stratum with bootstrap

multiplier 1 rather than combine substantively unrelated strata. All other

strata use Rao-Wu rescaled PSU sampling. A design-diagnostics table records

the number of strata and PSUs in every wave.

- Rationale: the choice is explicit, reproducible, and avoids an arbitrary

pooling rule. It can modestly understate between-PSU uncertainty for the

affected strata and is therefore disclosed as a limitation.

- Not changed: point estimates, frozen model, analytic records, outcome,

predictors, update experiment, metrics, or calibration criterion.

# Optimization attempt log

The scientific objective is not to optimize a favorable result. This file records design or computational changes after the frozen redesign protocol.

| Date | Attempt or change | Reason | Result inspected before change? | Effect on scope | Decision |

|---|---|---|---|---|---|

| 2026-07-15 | Initial independent redesign frozen | Resolve Red companion-manuscript overlap | Existing old-paper results known; new learning curves not run | Replaces missingness/group question with model-maintenance question | Proceed |

| 2026-07-15 | Add calibration-attainment rate to grouped export | Frozen protocol required a proportion; binary median was uninformative | Yes, complete run had finished | Reporting only; no models, data, thresholds, or claims changed | Retain and document as Amendment 2 |

| 2026-07-15 | Remove three protected identity inputs | Strengthen scientific independence and avoid unnecessary use of protected attributes | Yes; a diagnostic comparison was run | Source score changes from 17 to 14 predictors; all SCI results must be regenerated | Retain and document as Amendment 3 |

| 2026-07-15 | Normalize weights within every penalized fit | Correct inconsistent effective ridge penalty across source and target fits | Yes; found during code review | Computational correction; previous refit comparisons are superseded | Retain and document as Amendment 3 |

# Reproducibility archive

This archive reproduces the analysis reported in Temporal maintenance of an adolescent suicide attempt screening score in national surveys: An observational study.

## Contents

- scripts/: data construction, model-maintenance experiment, design-based bootstrap, verification, and display-generation code

- results/sci_maintenance/: aggregate numerical outputs, fitted frozen model, and run metadata; no individual-level predictions are distributed

- results/revision/: penalty sensitivity, drift decomposition, singleton stratum sensitivity, paired nonparametric comparisons, and revision metadata

- derived_data/: variable-harmonization ledger and aggregate wave summary only; individual-level derived records are rebuilt locally

- raw_data/source_checksums.csv: file sizes and SHA-256 hashes for the public CDC source files

- protocol/: frozen redesign protocol, amendments, and the post-run optimization log

- requirements-lock.txt: tested Python package versions

Public raw YRBS files are not redistributed. Download the national ASCII data, SPSS syntax, and user-guide PDF for 2015, 2017, 2019, 2021, and 2023 from the CDC national YRBS data page. The checksum verifier expects 15 source files in total: 2015.dat, 2015.sps, 2015_guide.pdf, ..., 2023.dat, 2023.sps, and 2023_guide.pdf. Place them in raw_data/, or set the YRBS_RAW_DIR environment variable to a directory containing those files.

## Tested environment

- Python 3.12

- Package versions in requirements-lock.txt

- Windows 11; the scripts use portable pathlib paths and no proprietary software

Create and activate a clean virtual environment, then run:

text

python -m pip install -r requirements-lock.txt

python scripts/verify_source_checksums.py

python scripts/build_harmonized_data.py

python scripts/run_sci_maintenance_learning_curves.py

python scripts/run_sci_frozen_bootstrap.py

python scripts/verify_sci_maintenance_learning_curves.py

python scripts/run_revision_analyses.py

python scripts/verify_revision_analyses.py

python scripts/build_sci_maintenance_artifacts.py

The principal verification script must end with:

text

SCI_MAINTENANCE_VERIFICATION_PASS

The revision verification script must end with:

text

MEDICINE_REVISION_ANALYSES_VERIFICATION_PASS

The full learning-curve experiment and 500-replicate complex-survey bootstrap are computationally intensive. The included result files permit result and display inspection without rerunning those stages. Running the pipeline overwrites files under results/sci_maintenance/ and creates publication displays under sci_independent_revision/.

## Design safeguards

- The 2015 score, its ridge penalty, and 14 non-suicide, non-identity predictors are frozen before target-wave evaluation.

- Update and evaluation folds do not share primary sampling units.

- All update strategies within a repetition use the same target-wave sample.

- Bootstrap uncertainty is stratified by survey design. The primary analysis treats analytically restricted single PSU strata as certainty strata, and the revision archive reports merging and omission sensitivities.

- The 5th and 95th percentiles across update repetitions quantify split instability, not national confidence intervals.

## Privacy and data responsibility

The files are public, deidentified surveillance data. This archive intentionally omits the row-level harmonized dataset and row-level out-of-fold predictions. The included learning-curve run files contain only aggregate performance statistics from repeated update experiments. Users remain responsible for CDC terms, ethical use, and any institutional requirements that apply to secondary analyses.

# Software environment

joblib==1.5.3

lxml==6.0.2

matplotlib==3.11.0

numpy==2.3.5

pandas==3.0.1

pillow==12.2.0

pyarrow==24.0.0

pypdf==6.10.0

scikit-learn==1.9.0

scipy==1.18.0

statsmodels==0.14.6

tabulate==0.10.0

xgboost==3.0.2
