# Supplementary material for: Temporal maintenance of an adolescent suicide attempt screening score in national surveys: An observational study
Source: Medicine (Baltimore). 2026 Aug 28;105(35):e50513. doi: 10.1097/MD.0000000000050513 (PMC13529099; doi:10.1097/MD.0000000000050513)
Supplement: Supplementary file 2 [file medi-105-e50513-s002.docx]

**Supplemental Digital Content 13**

**Wave summary data**

Temporal maintenance of an adolescent suicide attempt screening score in national surveys: An observational study

First author: Xiaotian Ma

*Source file: wave_summary.csv*

This Word document contains aggregate or metadata records only and does not include individual student records.

year,n,outcome_observed_n,outcome_missing_unweighted_pct,outcome_missing_weighted_pct,attempt_events_observed,attempt_weighted_pct_among_observed

2015,15624,12567,19.566052227342546,11.567816736467694,1203,8.564627356459587

2017,14765,10686,27.626142905519814,14.997088825889923,837,7.3714715468893965

2019,13677,10520,23.082547342253424,16.433032340825502,1067,8.905635008953746

2021,17232,15573,9.627437325905298,9.866667014856688,1753,10.170608668544727

2023,20103,19336,3.815350942645379,3.7166434112738775,1995,9.462945164439509
