# Supplementary material for: Temporal maintenance of an adolescent suicide attempt screening score in national surveys: An observational study
Source: Medicine (Baltimore). 2026 Aug 28;105(35):e50513. doi: 10.1097/MD.0000000000050513 (PMC13529099; doi:10.1097/MD.0000000000050513)
Supplement: Supplementary file 3 [file medi-105-e50513-s003.docx]

**Supplemental Digital Content 4**

**Harmonization code**

Temporal maintenance of an adolescent suicide attempt screening score in national surveys: An observational study

First author: Xiaotian Ma

*Source file: build_harmonized_data.py*

This Word document contains the complete source code for the identified analysis step.

```
from __future__ import annotations

import csv
import os
import re
from pathlib import Path

import numpy as np
import pandas as pd


ROOT = Path(__file__).resolve().parents[1]


def resolve_raw_dir() -> Path:
    """Locate the CDC source files without tying reproduction to one machine."""
    candidates = []
    if os.environ.get("YRBS_RAW_DIR"):
        candidates.append(Path(os.environ["YRBS_RAW_DIR"]).expanduser())
    candidates.extend(
        [
            ROOT / "raw_data",
            ROOT.parent / "frontiers_psychology_yrbs_topic_audit" / "cdc_yrbs",
        ]
    )
    required = [Path(f"{year}.{suffix}") for year in (2015, 2017, 2019, 2021, 2023) for suffix in ("dat", "sps")]
    for candidate in candidates:
        candidate = candidate.resolve()
        if all((candidate / filename).is_file() for filename in required):
            return candidate
    searched = "\n  - ".join(str(path.resolve()) for path in candidates)
    raise FileNotFoundError(
        "Could not locate all YRBS .dat and .sps source files. "
        "Set YRBS_RAW_DIR or place them in project/raw_data. Searched:\n  - "
        + searched
    )


RAW_DIR = resolve_raw_dir()
DERIVED_DIR = ROOT / "derived_data"
YEARS = (2015, 2017, 2019, 2021, 2023)


VARIABLES = {
    2015: dict(
        attempt="Q29", injury="Q30", ideation="Q27", plan="Q28",
        sex="Q2", grade="Q3", race="RACEETH", sexual_identity="Q68",
        safety_absence="Q16", weapon_threat="Q17", fight_school="Q20",
        forced_sex="Q21", bullying_school="Q24", electronic_bullying="Q25",
        sad_hopeless="Q26", current_cigarette="Q33", current_vaping="Q40",
        current_alcohol="Q43", current_marijuana="Q49",
        no_physical_activity="Q80", short_sleep="Q88",
    ),
    2017: dict(
        attempt="Q28", injury="Q29", ideation="Q26", plan="Q27",
        sex="Q2", grade="Q3", race="RACEETH", sexual_identity="Q67",
        safety_absence="Q15", weapon_threat="Q16", fight_school="Q18",
        forced_sex="Q19", bullying_school="Q23", electronic_bullying="Q24",
        sad_hopeless="Q25", current_cigarette="Q32", current_vaping="Q35",
        current_alcohol="Q42", current_marijuana="Q48",
        no_physical_activity="Q79", short_sleep="Q88",
    ),
    2019: dict(
        attempt="Q28", injury="Q29", ideation="Q26", plan="Q27",
        sex="Q2", grade="Q3", race="RACEETH", sexual_identity="Q66",
        safety_absence="Q15", weapon_threat="Q16", fight_school="Q18",
        forced_sex="Q19", bullying_school="Q23", electronic_bullying="Q24",
        sad_hopeless="Q25", current_cigarette="Q32", current_vaping="Q35",
        current_alcohol="Q41", current_marijuana="Q47",
        no_physical_activity="Q78", short_sleep="Q88",
    ),
    2021: dict(
        attempt="Q28", injury="Q29", ideation="Q26", plan="Q27",
        sex="Q2", grade="Q3", race="RACEETH", sexual_identity="Q65",
        safety_absence="Q14", weapon_threat="Q15", fight_school="Q17",
        forced_sex="Q19", bullying_school="Q23", electronic_bullying="Q24",
        sad_hopeless="Q25", current_cigarette="Q32", current_vaping="Q35",
        current_alcohol="Q41", current_marijuana="Q47",
        no_physical_activity="Q77", short_sleep="Q86",
    ),
    2023: dict(
        attempt="Q29", injury="Q30", ideation="Q27", plan="Q28",
        sex="Q2", grade="Q3", race="RACEETH", sexual_identity="Q64",
        safety_absence="Q14", weapon_threat="Q15", fight_school="Q17",
        forced_sex="Q19", bullying_school="Q24", electronic_bullying="Q25",
        sad_hopeless="Q26", current_cigarette="Q33", current_vaping="Q36",
        current_alcohol="Q42", current_marijuana="Q48",
        no_physical_activity="Q76", short_sleep="Q85",
    ),
}


def parse_sps(path: Path) -> tuple[dict[str, tuple[int, int]], dict[str, str]]:
    text = path.read_text(encoding="latin-1")
    match = re.search(r"DATA LIST.*?/\s*(.*?)\bEXECUTE\.", text, flags=re.I | re.S)
    if not match:
        raise ValueError(f"DATA LIST block not found in {path}")
    layout: dict[str, tuple[int, int]] = {}
    for name, start, end in re.findall(
        r"\b([A-Za-z][A-Za-z0-9_]*)\s+(\d+)\s*-\s*(\d+)(?:\s*\(A\))?",
        match.group(1),
    ):
        layout[name.upper()] = (int(start) - 1, int(end))
    label_match = re.search(
        r"\bVARIABLE LABELS\b(.*?)(?:\bVALUE LABELS\b|\bMISSING VALUES\b)",
        text,
        flags=re.I | re.S,
    )
    labels: dict[str, str] = {}
    if label_match:
        for name, label in re.findall(
            r'^\s*([A-Za-z][A-Za-z0-9_]*)\s+"([^"]*)"',
            label_match.group(1),
            flags=re.M,
        ):
            labels[name.upper()] = label.strip()
    return layout, labels


def value(line: str, layout: dict[str, tuple[int, int]], variable: str) -> str:
    start, end = layout[variable.upper()]
    return line[start:end].strip()


def numeric(raw: str) -> float:
    try:
        return float(raw)
    except ValueError:
        return np.nan


def yes_no(raw: str) -> str:
    return {"1": "yes", "2": "no"}.get(raw, "missing")


def zero_first_binary(raw: str) -> str:
    if not raw:
        return "missing"
    return "no" if raw == "1" else "yes"


def attempt(raw: str) -> float:
    if not raw:
        return np.nan
    return 0.0 if raw == "1" else 1.0


def injury(raw: str) -> str:
    return {
        "1": "did_not_attempt",
        "2": "injury_treated",
        "3": "no_treated_injury",
    }.get(raw, "missing")


def sex(raw: str) -> str:
    return {"1": "female", "2": "male"}.get(raw, "missing")


def grade(raw: str) -> str:
    return {
        "1": "grade_9", "2": "grade_10", "3": "grade_11",
        "4": "grade_12", "5": "ungraded_other",
    }.get(raw, "missing")


def race(raw: str) -> str:
    return {
        "1": "aian", "2": "asian", "3": "black", "4": "nhopi",
        "5": "white", "6": "hispanic", "7": "multiple_hispanic",
        "8": "multiple_nonhispanic",
    }.get(raw, "missing")


def race_primary_group(raw: str) -> str:
    if raw == "5":
        return "white"
    if raw == "3":
        return "black"
    if raw in {"6", "7"}:
        return "hispanic_latino"
    if raw:
        return "other_multiracial"
    return "missing"


def sexual_identity(raw: str, year: int) -> str:
    if not raw:
        return "missing"
    if raw == "1":
        return "heterosexual"
    if raw == "2":
        return "gay_lesbian"
    if raw == "3":
        return "bisexual"
    if year <= 2019 and raw == "4":
        return "not_sure"
    if year >= 2021 and raw == "4":
        return "some_other_way"
    if year >= 2021 and raw == "5":
        return "not_sure"
    if year >= 2021 and raw == "6":
        return "does_not_know_meaning"
    return "missing"


def sexual_identity_primary(raw: str) -> str:
    if raw == "1":
        return "heterosexual"
    if raw in {"2", "3"}:
        return "gay_lesbian_bisexual"
    return "not_comparable"


def short_sleep(raw: str) -> str:
    if not raw:
        return "missing"
    return "yes" if int(raw) <= 4 else "no"


def no_physical_activity(raw: str) -> str:
    if not raw:
        return "missing"
    return "yes" if raw == "1" else "no"


def main() -> None:
    DERIVED_DIR.mkdir(parents=True, exist_ok=True)
    records: list[dict[str, object]] = []
    harmonization: list[dict[str, object]] = []

    for year in YEARS:
        layout, labels = parse_sps(RAW_DIR / f"{year}.sps")
        mapping = VARIABLES[year]
        for concept, variable_name in mapping.items():
            harmonization.append(
                {
                    "concept": concept,
                    "year": year,
                    "source_variable": variable_name,
                    "source_label": labels.get(variable_name.upper(), ""),
                }
            )

        with (RAW_DIR / f"{year}.dat").open("r", encoding="latin-1") as fh:
            for row_number, line in enumerate(fh, start=1):
                raw = {key: value(line, layout, var) for key, var in mapping.items()}
                outcome = attempt(raw["attempt"])
                records.append(
                    {
                        "record_id": f"{year}_{row_number:05d}",
                        "year": year,
                        "survey_season": "fall" if year == 2021 else "spring",
                        "administration_mode": "tablet" if year == 2023 else "paper_pencil",
                        "weight": numeric(value(line, layout, "WEIGHT")),
                        "stratum": value(line, layout, "STRATUM"),
                        "psu": value(line, layout, "PSU"),
                        "attempt": outcome,
                        "attempt_observed": int(not np.isnan(outcome)),
                        "injury_aux": injury(raw["injury"]),
                        "ideation_aux": yes_no(raw["ideation"]),
                        "plan_aux": yes_no(raw["plan"]),
                        "sex": sex(raw["sex"]),
                        "grade": grade(raw["grade"]),
                        "race_ethnicity": race(raw["race"]),
                        "race_primary_group": race_primary_group(raw["race"]),
                        "sexual_identity": sexual_identity(raw["sexual_identity"], year),
                        "sexual_identity_primary": sexual_identity_primary(raw["sexual_identity"]),
                        "safety_absence": zero_first_binary(raw["safety_absence"]),
                        "weapon_threat_school": zero_first_binary(raw["weapon_threat"]),
                        "physical_fight_school": zero_first_binary(raw["fight_school"]),
                        "forced_sex": yes_no(raw["forced_sex"]),
                        "bullying_school": yes_no(raw["bullying_school"]),
                        "electronic_bullying": yes_no(raw["electronic_bullying"]),
                        "sad_hopeless": yes_no(raw["sad_hopeless"]),
                        "current_cigarette": zero_first_binary(raw["current_cigarette"]),
                        "current_vaping": zero_first_binary(raw["current_vaping"]),
                        "current_alcohol": zero_first_binary(raw["current_alcohol"]),
                        "current_marijuana": zero_first_binary(raw["current_marijuana"]),
                        "no_physical_activity": no_physical_activity(raw["no_physical_activity"]),
                        "short_sleep": short_sleep(raw["short_sleep"]),
                    }
                )

    data = pd.DataFrame.from_records(records)
    data.to_parquet(DERIVED_DIR / "yrbs_2015_2023_harmonized.parquet", index=False)
    data.to_csv(DERIVED_DIR / "yrbs_2015_2023_harmonized.csv", index=False)
    pd.DataFrame(harmonization).to_csv(
        DERIVED_DIR / "variable_harmonization_table.csv", index=False, encoding="utf-8-sig"
    )

    summary_rows = []
    for year, frame in data.groupby("year", sort=True):
        observed = frame[frame["attempt_observed"] == 1]
        weighted_missing = np.average(1 - frame["attempt_observed"], weights=frame["weight"])
        weighted_attempt = np.average(observed["attempt"], weights=observed["weight"])
        summary_rows.append(
            {
                "year": year,
                "n": len(frame),
                "outcome_observed_n": len(observed),
                "outcome_missing_unweighted_pct": 100 * (1 - frame["attempt_observed"].mean()),
                "outcome_missing_weighted_pct": 100 * weighted_missing,
                "attempt_events_observed": int(observed["attempt"].sum()),
                "attempt_weighted_pct_among_observed": 100 * weighted_attempt,
            }
        )
    pd.DataFrame(summary_rows).to_csv(
        DERIVED_DIR / "wave_summary.csv", index=False, encoding="utf-8-sig"
    )
    print(f"Wrote {len(data):,} records to {DERIVED_DIR}")


if __name__ == "__main__":
    main()
```
