# Supplementary material for: Temporal maintenance of an adolescent suicide attempt screening score in national surveys: An observational study
Source: Medicine (Baltimore). 2026 Aug 28;105(35):e50513. doi: 10.1097/MD.0000000000050513 (PMC13529099; doi:10.1097/MD.0000000000050513)
Supplement: Supplementary file 4 [file medi-105-e50513-s004.docx]

**Supplemental Digital Content 12**

**Variable harmonization data**

Temporal maintenance of an adolescent suicide attempt screening score in national surveys: An observational study

First author: Xiaotian Ma

*Source file: variable_harmonization_table.csv*

This Word document contains aggregate or metadata records only and does not include individual student records.

concept,year,source_variable,source_label

attempt,2015,Q29,Attempted suicide

injury,2015,Q30,Injurious suicide attempt

ideation,2015,Q27,Considered suicide

plan,2015,Q28,Made a suicide plan

sex,2015,Q2,What is your sex

grade,2015,Q3,In what grade are you

race,2015,RACEETH,Race/Ethnicity

sexual_identity,2015,Q68,Sexual identity

safety_absence,2015,Q16,Safety concerns at school

weapon_threat,2015,Q17,Threatened at school

fight_school,2015,Q20,Physical fighting at school

forced_sex,2015,Q21,Forced sexual intercourse

bullying_school,2015,Q24,Bullying at school

electronic_bullying,2015,Q25,Electronic bullying

sad_hopeless,2015,Q26,Sad or hopeless

current_cigarette,2015,Q33,Current cigarette use

current_vaping,2015,Q40,Current electronic vapor product use

current_alcohol,2015,Q43,Current alcohol use

current_marijuana,2015,Q49,Current marijuana use

no_physical_activity,2015,Q80,Physical activity >= 5 days

short_sleep,2015,Q88,Hours of sleep on school night

attempt,2017,Q28,Attempted suicide

injury,2017,Q29,Injurious suicide attempt

ideation,2017,Q26,Considered suicide

plan,2017,Q27,Made a suicide plan

sex,2017,Q2,What is your sex

grade,2017,Q3,In what grade are you

race,2017,RACEETH,Race/Ethnicity

sexual_identity,2017,Q67,Sexual identity

safety_absence,2017,Q15,Safety concerns at school

weapon_threat,2017,Q16,Threatened at school

fight_school,2017,Q18,Physical fighting at school

forced_sex,2017,Q19,Ever been physically forced sexual intercourse

bullying_school,2017,Q23,Bullying at school

electronic_bullying,2017,Q24,Electronic bullying

sad_hopeless,2017,Q25,Sad or hopeless

current_cigarette,2017,Q32,Current cigarette use

current_vaping,2017,Q35,Current electronic vapor product use

current_alcohol,2017,Q42,Current alcohol use

current_marijuana,2017,Q48,Current marijuana use

no_physical_activity,2017,Q79,Physical activity >= 5 days

short_sleep,2017,Q88,Hours of sleep on school night

attempt,2019,Q28,Attempted suicide

injury,2019,Q29,Injurious suicide attempt

ideation,2019,Q26,Considered suicide

plan,2019,Q27,Made a suicide plan

sex,2019,Q2,What is your sex

grade,2019,Q3,In what grade are you

race,2019,RACEETH,Race/Ethnicity

sexual_identity,2019,Q66,Sexual identity

safety_absence,2019,Q15,Safety concerns at school

weapon_threat,2019,Q16,Threatened at school

fight_school,2019,Q18,Physical fighting at school

forced_sex,2019,Q19,Ever been physically forced sexual intercourse

bullying_school,2019,Q23,Bullying at school

electronic_bullying,2019,Q24,Electronic bullying

sad_hopeless,2019,Q25,Sad or hopeless

current_cigarette,2019,Q32,Current cigarette use

current_vaping,2019,Q35,Current electronic vapor product use

current_alcohol,2019,Q41,Current alcohol use

current_marijuana,2019,Q47,Current marijuana use

no_physical_activity,2019,Q78,Physical activity >= 5 days

short_sleep,2019,Q88,Hours of sleep on school night

attempt,2021,Q28,Actually attempted suicide

injury,2021,Q29,Injurious suicide attempt

ideation,2021,Q26,Considered suicide

plan,2021,Q27,Made a suicide plan past 12 months

sex,2021,Q2,What is your sex

grade,2021,Q3,In what grade are you

race,2021,RACEETH,Race/Ethnicity

sexual_identity,2021,Q65,Sexual identity

safety_absence,2021,Q14,Safety concerns at school

weapon_threat,2021,Q15,Threatened at school

fight_school,2021,Q17,Physical fighting at school

forced_sex,2021,Q19,Ever been physically forced sexual intercourse

bullying_school,2021,Q23,Bullied at school

electronic_bullying,2021,Q24,Electronic bullying

sad_hopeless,2021,Q25,Sad or hopeless

current_cigarette,2021,Q32,Current cigarette use

current_vaping,2021,Q35,Current electronic vapor product use

current_alcohol,2021,Q41,Current alcohol use

current_marijuana,2021,Q47,Current marijuana use

no_physical_activity,2021,Q77,Physical activity >= 5 days

short_sleep,2021,Q86,Hours of sleep on school night

attempt,2023,Q29,Actually attempted suicide

injury,2023,Q30,Injurious suicide attempt

ideation,2023,Q27,Considered suicide

plan,2023,Q28,Made a suicide plan past 12 months

sex,2023,Q2,What is your sex

grade,2023,Q3,In what grade are you

race,2023,RACEETH,Race/Ethnicity

sexual_identity,2023,Q64,Sexual identity

safety_absence,2023,Q14,Safety concerns at school

weapon_threat,2023,Q15,Threatened at school

fight_school,2023,Q17,Physical fighting at school

forced_sex,2023,Q19,Ever been physically forced sexual intercourse

bullying_school,2023,Q24,Bullied at school

electronic_bullying,2023,Q25,Electronic bullying

sad_hopeless,2023,Q26,Sad or hopeless

current_cigarette,2023,Q33,Current cigarette use

current_vaping,2023,Q36,Current electronic vapor product use

current_alcohol,2023,Q42,Current alcohol use

current_marijuana,2023,Q48,Current marijuana use

no_physical_activity,2023,Q76,Physical activity >= 5 days

short_sleep,2023,Q85,Hours of sleep on school night
