# Supplementary material for: Temporal maintenance of an adolescent suicide attempt screening score in national surveys: An observational study
Source: Medicine (Baltimore). 2026 Aug 28;105(35):e50513. doi: 10.1097/MD.0000000000050513 (PMC13529099; doi:10.1097/MD.0000000000050513)
Supplement: Supplementary file 5 [file medi-105-e50513-s005.docx]

**Supplemental Digital Content 5**

**Artifact code**

Temporal maintenance of an adolescent suicide attempt screening score in national surveys: An observational study

First author: Xiaotian Ma

*Source file: build_sci_maintenance_artifacts.py*

This Word document contains the complete source code for the identified analysis step.

```
"""Build publication displays for the independent SCI maintenance study."""

from __future__ import annotations

from pathlib import Path

import joblib
import matplotlib as mpl
import matplotlib.pyplot as plt
import numpy as np
import pandas as pd


ROOT = Path(__file__).resolve().parents[1]
RESULTS = ROOT / "results" / "sci_maintenance"
REVISION = ROOT / "sci_independent_revision"
FIGURES = REVISION / "figures"
TABLES = REVISION / "tables"
FIGURES.mkdir(parents=True, exist_ok=True)
TABLES.mkdir(parents=True, exist_ok=True)

WAVES = [2017, 2019, 2021, 2023]
BUDGETS = [250, 500, 1000, 2000, 4000]
BUDGET_LABELS = ["250", "500", "1,000", "2,000", "4,000", "Full"]
STRATEGIES = ["intercept", "logistic", "refit"]
STRATEGY_LABELS = {
    "intercept": "Intercept",
    "logistic": "Intercept and slope",
    "refit": "Full specification",
}
COLORS = {
    "intercept": "#0072B2",
    "logistic": "#009E73",
    "refit": "#D55E00",
    "static": "#4D4D4D",
    "frozen": "#0072B2",
    "recent": "#D55E00",
}
MARKERS = {"intercept": "o", "logistic": "s", "refit": "^"}


def style() -> None:
    mpl.rcParams.update(
        {
            "font.family": "sans-serif",
            "font.sans-serif": ["Arial", "Liberation Sans", "DejaVu Sans"],
            "font.size": 8.5,
            "axes.titlesize": 9.5,
            "axes.labelsize": 9,
            "xtick.labelsize": 8,
            "ytick.labelsize": 8,
            "legend.fontsize": 8,
            "axes.spines.top": False,
            "axes.spines.right": False,
            "axes.linewidth": 0.7,
            "grid.color": "#D9D9D9",
            "grid.linewidth": 0.5,
            "grid.alpha": 0.8,
            "pdf.fonttype": 42,
            "ps.fonttype": 42,
        }
    )


def save(fig: mpl.figure.Figure, stem: str) -> None:
    fig.savefig(FIGURES / f"{stem}.pdf", bbox_inches="tight")
    fig.savefig(FIGURES / f"{stem}.png", dpi=600, bbox_inches="tight")
    plt.close(fig)


def budget_rows(frame: pd.DataFrame, strategy: str) -> pd.DataFrame:
    selected = frame[
        frame["strategy"].eq(strategy)
        & (frame["budget"].isin(BUDGETS) | frame["budget_label"].eq("full_outer_training"))
    ].copy()
    selected["x"] = selected["budget"].map({value: index for index, value in enumerate(BUDGETS)})
    selected.loc[selected["budget_label"].eq("full_outer_training"), "x"] = len(BUDGETS)
    return selected.sort_values("x")


def figure_temporal_transport(points: pd.DataFrame, intervals: pd.DataFrame) -> None:
    years = [2015, *WAVES]
    point_lookup = points.set_index("year")
    interval_lookup = intervals.set_index(["year", "metric"])
    panels = [
        ("auroc", "(A) AUROC", (0.82, 0.89), None),
        ("brier", "(B) Brier score", (0.045, 0.080), None),
        ("calibration_intercept", "(C) Calibration intercept", (-0.46, 0.18), 0.0),
        ("calibration_slope", "(D) Calibration slope", (0.84, 1.16), 1.0),
    ]
    fig, axes = plt.subplots(2, 2, figsize=(7.2, 5.2), constrained_layout=True)
    x = np.arange(len(years))
    for ax, (metric, title, limits, ideal) in zip(axes.flat, panels, strict=True):
        values = point_lookup.loc[years, metric].to_numpy(dtype=float)
        lower = np.array(
            [interval_lookup.loc[(year, metric), "ci_lower"] for year in years], dtype=float
        )
        upper = np.array(
            [interval_lookup.loc[(year, metric), "ci_upper"] for year in years], dtype=float
        )
        ax.errorbar(
            x,
            values,
            yerr=np.vstack([values - lower, upper - values]),
            color=COLORS["frozen"],
            marker="o",
            linewidth=1.6,
            markersize=4.5,
            capsize=2.5,
            elinewidth=0.9,
        )
        if ideal is not None:
            ax.axhline(ideal, color="#777777", linewidth=0.8, linestyle=":")
        ax.set_title(title, loc="left", fontweight="bold")
        ax.set_xticks(x, years)
        ax.set_ylim(*limits)
        ax.grid(axis="y")
    axes[1, 0].set_xlabel("Survey year")
    axes[1, 1].set_xlabel("Survey year")
    save(fig, "figure_1_frozen_score_transportability")


def figure_maintenance_learning_curves(summary: pd.DataFrame) -> None:
    fig, axes = plt.subplots(4, 2, figsize=(7.2, 9.2), sharex=True, constrained_layout=True)
    x = np.arange(len(BUDGET_LABELS))
    for row, year in enumerate(WAVES):
        wave = summary[summary["target_year"].eq(year)]
        static = wave[wave["strategy"].eq("static")].iloc[0]
        ax_error = axes[row, 0]
        ax_rate = axes[row, 1]
        ax_error.axhline(
            static["absolute_calibration_intercept_error_median"],
            color=COLORS["static"],
            linestyle="--",
            linewidth=1.0,
            label="No update" if row == 0 else None,
        )
        ax_rate.axhline(
            100 * static["close_calibration_rate"],
            color=COLORS["static"],
            linestyle="--",
            linewidth=1.0,
            label="No update" if row == 0 else None,
        )
        for strategy in STRATEGIES:
            values = budget_rows(wave, strategy)
            med = values["absolute_calibration_intercept_error_median"].to_numpy()
            lo = values["absolute_calibration_intercept_error_p05"].to_numpy()
            hi = values["absolute_calibration_intercept_error_p95"].to_numpy()
            ax_error.fill_between(x, lo, hi, color=COLORS[strategy], alpha=0.10, linewidth=0)
            ax_error.plot(
                x,
                med,
                color=COLORS[strategy],
                marker=MARKERS[strategy],
                markersize=3.7,
                linewidth=1.25,
                label=STRATEGY_LABELS[strategy] if row == 0 else None,
            )
            ax_rate.plot(
                x,
                100 * values["close_calibration_rate"].to_numpy(),
                color=COLORS[strategy],
                marker=MARKERS[strategy],
                markersize=3.7,
                linewidth=1.25,
                label=STRATEGY_LABELS[strategy] if row == 0 else None,
            )
        ax_error.set_ylabel(f"{year}")
        ax_error.set_ylim(0, 0.50)
        ax_rate.set_ylim(-2, 102)
        ax_error.grid(axis="y")
        ax_rate.grid(axis="y")
        if row == 0:
            ax_error.set_title("(A) Absolute calibration intercept error", loc="left", fontweight="bold")
            ax_rate.set_title("(B) Close calibration (%)", loc="left", fontweight="bold")
    for ax in axes[-1, :]:
        ax.set_xticks(x, BUDGET_LABELS)
        ax.set_xlabel("New target survey labels")
    handles, labels = axes[0, 0].get_legend_handles_labels()
    # De-duplicate the shared static and strategy labels.
    unique = dict(zip(labels, handles))
    fig.legend(
        unique.values(),
        unique.keys(),
        loc="outside lower center",
        ncol=4,
        frameon=False,
    )
    save(fig, "figure_2_maintenance_learning_curves")


def figure_performance_tradeoffs(paired_summary: pd.DataFrame) -> None:
    fig, axes = plt.subplots(4, 2, figsize=(7.2, 9.2), sharex=True, constrained_layout=True)
    x = np.arange(len(BUDGET_LABELS))
    metrics = [
        ("delta_brier_vs_static", "(A) Change in Brier score"),
        ("delta_auroc_vs_static", "(B) Change in AUROC"),
    ]
    for row, year in enumerate(WAVES):
        wave = paired_summary[paired_summary["target_year"].eq(year)]
        for column, (metric, title) in enumerate(metrics):
            ax = axes[row, column]
            ax.axhline(0, color="#777777", linewidth=0.8, linestyle=":")
            for strategy in STRATEGIES:
                values = budget_rows(wave, strategy)
                med = values[f"{metric}_median"].to_numpy()
                lo = values[f"{metric}_p05"].to_numpy()
                hi = values[f"{metric}_p95"].to_numpy()
                ax.fill_between(x, lo, hi, color=COLORS[strategy], alpha=0.10, linewidth=0)
                ax.plot(
                    x,
                    med,
                    color=COLORS[strategy],
                    marker=MARKERS[strategy],
                    markersize=3.7,
                    linewidth=1.25,
                    label=STRATEGY_LABELS[strategy] if row == 0 else None,
                )
            ax.grid(axis="y")
            if column == 0:
                ax.set_ylabel(f"{year}")
            if row == 0:
                ax.set_title(title, loc="left", fontweight="bold")
    for ax in axes[-1, :]:
        ax.set_xticks(x, BUDGET_LABELS)
        ax.set_xlabel("New target survey labels")
    handles, labels = axes[0, 0].get_legend_handles_labels()
    fig.legend(handles, labels, loc="outside lower center", ncol=3, frameon=False)
    save(fig, "figure_s1_performance_tradeoffs")


def figure_fixed_capacity(summary: pd.DataFrame) -> None:
    fig, axes = plt.subplots(4, 2, figsize=(7.2, 9.2), sharex=True, constrained_layout=True)
    x = np.arange(len(BUDGET_LABELS))
    metrics = [
        ("top10_sensitivity", "(A) Sensitivity at 10% capacity"),
        ("top10_positive_predictive_value", "(B) Positive predictive value at 10% capacity"),
    ]
    for row, year in enumerate(WAVES):
        wave = summary[summary["target_year"].eq(year)]
        static = wave[wave["strategy"].eq("static")].iloc[0]
        for column, (metric, title) in enumerate(metrics):
            ax = axes[row, column]
            ax.axhline(
                static[f"{metric}_median"],
                color=COLORS["static"],
                linestyle="--",
                linewidth=1.0,
                label="No update" if row == 0 else None,
            )
            for strategy in STRATEGIES:
                values = budget_rows(wave, strategy)
                med = values[f"{metric}_median"].to_numpy()
                lo = values[f"{metric}_p05"].to_numpy()
                hi = values[f"{metric}_p95"].to_numpy()
                ax.fill_between(x, lo, hi, color=COLORS[strategy], alpha=0.10, linewidth=0)
                ax.plot(
                    x,
                    med,
                    color=COLORS[strategy],
                    marker=MARKERS[strategy],
                    markersize=3.7,
                    linewidth=1.25,
                    label=STRATEGY_LABELS[strategy] if row == 0 else None,
                )
            ax.set_ylim(0.30, 0.56)
            ax.grid(axis="y")
            if column == 0:
                ax.set_ylabel(f"{year}")
            if row == 0:
                ax.set_title(title, loc="left", fontweight="bold")
    for ax in axes[-1, :]:
        ax.set_xticks(x, BUDGET_LABELS)
        ax.set_xlabel("New target survey labels")
    handles, labels = axes[0, 0].get_legend_handles_labels()
    unique = dict(zip(labels, handles))
    fig.legend(unique.values(), unique.keys(), loc="outside lower center", ncol=4, frameon=False)
    save(fig, "figure_s2_fixed_capacity")


def figure_update_samples(diagnostics: pd.DataFrame) -> None:
    fig, axes = plt.subplots(1, 2, figsize=(7.2, 3.0), constrained_layout=True)
    for year, color, marker in zip(
        WAVES,
        ["#0072B2", "#009E73", "#D55E00", "#CC79A7"],
        ["o", "s", "^", "D"],
        strict=True,
    ):
        wave = diagnostics[diagnostics["target_year"].eq(year)].sort_values("budget")
        axes[0].plot(
            wave["budget"],
            wave["update_events_median"],
            color=color,
            marker=marker,
            linewidth=1.3,
            markersize=4,
            label=str(year),
        )
        axes[1].plot(
            wave["budget"],
            wave["update_effective_n_median"],
            color=color,
            marker=marker,
            linewidth=1.3,
            markersize=4,
            label=str(year),
        )
    axes[0].set_title("(A) Reported attempts", loc="left", fontweight="bold")
    axes[1].set_title("(B) Survey weighted effective sample size", loc="left", fontweight="bold")
    for ax in axes:
        ax.set_xlabel("New target survey labels")
        ax.grid(axis="y")
        ax.set_xscale("log", base=2)
        ax.set_xticks(BUDGETS, ["250", "500", "1,000", "2,000", "4,000"])
    axes[0].set_ylabel("Median per update sample")
    axes[1].set_ylabel("Median per update sample")
    handles, labels = axes[0].get_legend_handles_labels()
    fig.legend(handles, labels, loc="outside lower center", ncol=4, frameon=False)
    save(fig, "figure_s3_update_samples")


def interval_text(value: float, lower: float, upper: float, digits: int = 3) -> str:
    return f"{value:.{digits}f} ({lower:.{digits}f}, {upper:.{digits}f})"


def build_table_1(data: pd.DataFrame) -> pd.DataFrame:
    performance = pd.read_csv(RESULTS / "frozen_point_estimates.csv")
    intervals = pd.read_csv(RESULTS / "frozen_bootstrap_summary.csv")
    interval_lookup = intervals.set_index(["year", "metric"])
    rows: list[dict[str, object]] = []
    for year in [2015, *WAVES]:
        point = performance[performance["year"].eq(year)].iloc[0]
        wave = data[(data["year"].eq(year)) & data["attempt_observed"].eq(1)]
        row: dict[str, object] = {
            "Year": year,
            "N": f"{len(wave):,}",
            "Events": f"{int(wave['attempt'].sum()):,}",
        }
        for metric, label, digits, scale in [
            ("prevalence", "Prevalence, %", 1, 100),
            ("auroc", "AUROC", 3, 1),
            ("auprc", "AUPRC", 3, 1),
            ("calibration_intercept", "Calibration intercept", 3, 1),
            ("calibration_slope", "Calibration slope", 3, 1),
            ("brier", "Brier score", 3, 1),
        ]:
            ci = interval_lookup.loc[(year, metric)]
            if scale == 100:
                row[label] = interval_text(
                    100 * point[metric], 100 * ci["ci_lower"], 100 * ci["ci_upper"], digits
                )
            else:
                row[label] = interval_text(point[metric], ci["ci_lower"], ci["ci_upper"], digits)
        rows.append(row)
    table = pd.DataFrame(rows)
    table.to_csv(TABLES / "table_1_temporal_performance.csv", index=False)
    return table


def build_table_2(summary: pd.DataFrame, diagnostics: pd.DataFrame) -> pd.DataFrame:
    diagnostic_lookup = diagnostics.set_index(["target_year", "budget"])
    rows: list[dict[str, object]] = []
    for year in WAVES:
        budget = 1000
        for strategy in STRATEGIES:
            value = summary[
                summary["target_year"].eq(year)
                & summary["strategy"].eq(strategy)
                & summary["budget"].eq(budget)
            ].iloc[0]
            diagnostic = diagnostic_lookup.loc[(year, budget)]
            rows.append(
                {
                    "Year": year,
                    "Strategy": STRATEGY_LABELS[strategy],
                    "Events in update sample": f"{diagnostic['update_events_median']:.0f}",
                    "Absolute intercept error, median (5th, 95th)": interval_text(
                        value["absolute_calibration_intercept_error_median"],
                        value["absolute_calibration_intercept_error_p05"],
                        value["absolute_calibration_intercept_error_p95"],
                    ),
                    "Absolute slope error, median (5th, 95th)": interval_text(
                        value["absolute_calibration_slope_error_median"],
                        value["absolute_calibration_slope_error_p05"],
                        value["absolute_calibration_slope_error_p95"],
                    ),
                    "Close calibration, %": f"{100 * value['close_calibration_rate']:.0f}",
                    "Brier score, median (5th, 95th)": interval_text(
                        value["brier_median"], value["brier_p05"], value["brier_p95"]
                    ),
                    "AUROC, median (5th, 95th)": interval_text(
                        value["auroc_median"], value["auroc_p05"], value["auroc_p95"]
                    ),
                }
            )
    table = pd.DataFrame(rows)
    table.to_csv(TABLES / "table_2_update_performance_1000_labels.csv", index=False)
    return table


def write_latex_table(table: pd.DataFrame, path: Path, label: str, caption: str, tiny: bool = True) -> None:
    replacements = {
        "\\": r"\textbackslash{}",
        "&": r"\&",
        "%": r"\%",
        "$": r"\$",
        "#": r"\#",
        "_": r"\_",
        "{": r"\{",
        "}": r"\}",
        "~": r"\textasciitilde{}",
        "^": r"\textasciicircum{}",
    }

    def escape(value: object) -> str:
        output = str(value)
        for source, target in replacements.items():
            output = output.replace(source, target)
        return output

    alignment = "l" * len(table.columns)
    body_lines = [
        f"\\begin{{tabular}}{{{alignment}}}",
        "\\toprule",
        " & ".join(escape(column) for column in table.columns) + r" \\",
        "\\midrule",
    ]
    for row in table.itertuples(index=False, name=None):
        body_lines.append(" & ".join(escape(value) for value in row) + r" \\")
    body_lines.extend(["\\bottomrule", "\\end{tabular}"])
    body = "\n".join(body_lines)
    size = "\\scriptsize" if tiny else "\\small"
    output = "\n".join(
        [
            "\\begin{table*}[!ht]",
            "\\centering",
            f"\\caption{{{caption}}}",
            f"\\label{{{label}}}",
            size,
            "\\resizebox{\\textwidth}{!}{%",
            body.rstrip(),
            "}%",
            "\\end{table*}",
            "",
        ]
    )
    path.write_text(output, encoding="utf-8")


def main() -> None:
    style()
    summary = pd.read_csv(RESULTS / "maintenance_learning_curve_summary.csv")
    paired_summary = pd.read_csv(RESULTS / "maintenance_paired_differences_summary.csv")
    diagnostics = pd.read_csv(RESULTS / "maintenance_update_sample_diagnostics_summary.csv")
    rolling = pd.read_csv(RESULTS / "rolling_source_validation.csv")
    frozen_points = pd.read_csv(RESULTS / "frozen_point_estimates.csv")
    frozen_intervals = pd.read_csv(RESULTS / "frozen_bootstrap_summary.csv")
    data = pd.read_parquet(ROOT / "derived_data" / "yrbs_2015_2023_harmonized.parquet")

    figure_temporal_transport(frozen_points, frozen_intervals)
    figure_maintenance_learning_curves(summary)
    figure_performance_tradeoffs(paired_summary)
    figure_fixed_capacity(summary)
    figure_update_samples(diagnostics)

    table_1 = build_table_1(data)
    table_2 = build_table_2(summary, diagnostics)
    write_latex_table(
        table_1,
        TABLES / "table_1_temporal_performance.tex",
        "tab:main1",
        "Temporal Performance of the 2015 Screening Score",
    )
    write_latex_table(
        table_2,
        TABLES / "table_2_update_performance_1000_labels.tex",
        "tab:main2",
        "Update Performance With 1,000 New Labels",
    )

    harmonization = pd.read_csv(ROOT / "derived_data" / "variable_harmonization_table.csv")
    sci_concepts = [
        "attempt",
        "grade",
        "safety_absence",
        "weapon_threat",
        "fight_school",
        "forced_sex",
        "bullying_school",
        "electronic_bullying",
        "sad_hopeless",
        "current_cigarette",
        "current_vaping",
        "current_alcohol",
        "current_marijuana",
        "no_physical_activity",
        "short_sleep",
    ]
    harmonization[harmonization["concept"].isin(sci_concepts)].to_csv(
        TABLES / "table_s1_predictor_harmonization.csv", index=False
    )

    frozen_model = joblib.load(RESULTS / "frozen_source_model.joblib")
    feature_names = frozen_model.named_steps["preprocess"].get_feature_names_out()
    coefficients = frozen_model.named_steps["model"].coef_[0]
    pd.DataFrame(
        {
            "encoded_term": [name.replace("categorical__", "") for name in feature_names],
            "ridge_coefficient": coefficients,
            "odds_ratio_scale": np.exp(coefficients),
        }
    ).to_csv(TABLES / "table_s2_frozen_model_terms.csv", index=False)

    # Machine-readable supplementary tables contain every prespecified budget
    # and strategy; the manuscript supplement renders selected columns.
    rolling.to_csv(TABLES / "table_s3_rolling_source_validation.csv", index=False)
    summary.to_csv(TABLES / "table_s4_learning_curve_summary.csv", index=False)
    paired_summary.to_csv(TABLES / "table_s5_paired_differences_summary.csv", index=False)
    diagnostics.to_csv(TABLES / "table_s6_update_sample_diagnostics.csv", index=False)
    pd.read_csv(RESULTS / "frozen_bootstrap_design_diagnostics.csv").to_csv(
        TABLES / "table_s7_bootstrap_design_diagnostics.csv", index=False
    )
    pd.read_csv(RESULTS / "frozen_bootstrap_summary.csv").to_csv(
        TABLES / "table_s8_frozen_bootstrap_summary.csv", index=False
    )

    print(f"Built {len(list(FIGURES.glob('*.pdf')))} PDF figures and main tables in {REVISION}")


if __name__ == "__main__":
    main()
```
