# Supplementary material for: Temporal maintenance of an adolescent suicide attempt screening score in national surveys: An observational study
Source: Medicine (Baltimore). 2026 Aug 28;105(35):e50513. doi: 10.1097/MD.0000000000050513 (PMC13529099; doi:10.1097/MD.0000000000050513)
Supplement: Supplementary file 6 [file medi-105-e50513-s006.docx]

**Supplemental Digital Content 14**

**Frozen score estimates**

Temporal maintenance of an adolescent suicide attempt screening score in national surveys: An observational study

First author: Xiaotian Ma

*Source file: frozen_point_estimates.csv*

This Word document contains aggregate or metadata records only and does not include individual student records.

year,score_origin,n,effective_n,expected_events,prevalence,auroc,auprc,brier,scaled_brier,calibration_intercept,calibration_slope,observed_expected_ratio,sensitivity,false_negative_rate,positive_predictive_value,referral_fraction,weighted_log_loss,absolute_calibration_intercept_error,absolute_calibration_slope_error,capacity_threshold,top10_sensitivity,top10_positive_predictive_value,top10_referral_fraction,close_calibration

2015,2015 cross-fitted,12567.0,5842.121655132331,1183.3445,0.08564627356459587,0.8591870643533408,0.389724580488319,0.06362473417496849,0.18753760268761233,-0.03305081367247834,0.9666431130334396,0.976907096496851,0.45925865206624106,0.5407413479337589,0.39333894638287975,0.09999973944478144,0.2182616761669894,0.03305081367247834,0.03335688696656036,0.24854573288567855,0.45925865206624106,0.39333894638287975,0.09999973944478144,1.0

2017,2015 frozen model,10686.0,6834.4116150131185,925.1701,0.07371471546889397,0.8601100889825326,0.3887092377679062,0.05554088835712368,0.18658184073209216,-0.2662781858554113,0.9956287943903915,0.8253491280078259,0.4938448616097731,0.506155138390227,0.34952867728606185,0.10415063433992919,0.19621229881790847,0.2662781858554113,0.004371205609608464,0.2589873375886714,0.47844672023015006,0.35265953467494576,0.1000074019870152,0.0

2019,2015 frozen model,10520.0,6240.351246070618,1017.866,0.08905635008953747,0.856241231794382,0.38783791718232474,0.06605088378196856,0.1858166285001669,-0.11529779595047002,0.9926402389184689,0.9210979291291886,0.4643839169399508,0.5356160830600492,0.38040845811395774,0.10871560766024285,0.22512481971956658,0.11529779595047002,0.007359761081531135,0.2621843106632928,0.4491809334431055,0.39978692301194746,0.10005933701102819,0.0

2021,2015 frozen model,15573.0,7465.749225286986,1579.6734000000001,0.10170608668544727,0.8515625000389302,0.44067224828804763,0.07186769785506224,0.2133739365563434,0.02965953518780984,1.0134040898681458,1.0211846163865483,0.4706577954658222,0.5293422045341778,0.421406265896917,0.11359290646271007,0.2448441581407855,0.02965953518780984,0.013404089868145785,0.27126045287083456,0.4314394988229845,0.436868966905357,0.10044206934094847,1.0

2023,2015 frozen model,19336.0,7383.468311248124,1831.6335,0.09462945164439508,0.8596242232474842,0.4287899260930158,0.06780345405296319,0.20859437626883315,-0.14762652942101953,1.008825598136005,0.9024759280882273,0.5071069075773074,0.49289309242269264,0.38144260930047724,0.12580463592446775,0.230451655791709,0.14762652942101953,0.00882559813600503,0.29107427049338286,0.44704167072724976,0.42301680641662437,0.10000384741557959,0.0
