# Supplementary material for: Temporal maintenance of an adolescent suicide attempt screening score in national surveys: An observational study
Source: Medicine (Baltimore). 2026 Aug 28;105(35):e50513. doi: 10.1097/MD.0000000000050513 (PMC13529099; doi:10.1097/MD.0000000000050513)
Supplement: Supplementary file 7 [file medi-105-e50513-s007.docx]

**Supplemental Digital Content 8**

**Learning curve code**

Temporal maintenance of an adolescent suicide attempt screening score in national surveys: An observational study

First author: Xiaotian Ma

*Source file: run_sci_maintenance_learning_curves.py*

This Word document contains the complete source code for the identified analysis step.

```
"""Independent SCI revision: temporal model-maintenance learning curves.

This script intentionally excludes all missing-outcome estimands, subgroup
comparisons, and MAR/MNAR sensitivity analyses used in the companion
manuscript.  It evaluates how much newly observed target-wave data are needed
to recalibrate or refit a frozen 2015 screening score while keeping YRBS PSUs
separate between updating and evaluation.
"""

from __future__ import annotations

import argparse
import json
import time
from pathlib import Path

import joblib
import numpy as np
import pandas as pd
from scipy.special import expit, logit
from sklearn.model_selection import StratifiedGroupKFold

from analysis_utils import (
    PREDICTORS,
    effective_sample_size,
    logistic_pipeline,
    normalized_weights,
    performance_metrics,
    tune_logistic,
    weighted_quantile,
)


ROOT = Path(__file__).resolve().parents[1]
DATA_PATH = ROOT / "derived_data" / "yrbs_2015_2023_harmonized.parquet"
DEFAULT_OUT = ROOT / "results" / "sci_maintenance"
SEED = 20260715
SOURCE_YEAR = 2015
TARGET_YEARS = [2017, 2019, 2021, 2023]
DEFAULT_BUDGETS = [250, 500, 1000, 2000, 4000]
N_SPLITS = 5
DEFAULT_REPEATS = 50

# The independent SCI score deliberately excludes identity attributes that are
# the substantive comparison variables in the companion psychology paper.  It
# also excludes every direct suicide item.  Grade is retained as a schooling
# stage variable; the remaining inputs describe experiences and behaviours.
SCI_PREDICTORS = [
    predictor
    for predictor in PREDICTORS
    if predictor not in {"sex", "race_ethnicity", "sexual_identity_primary"}
]


def report(message: str) -> None:
    print(f"[{time.strftime('%H:%M:%S')}] {message}", flush=True)


def design_groups(frame: pd.DataFrame) -> np.ndarray:
    return (frame["stratum"].astype(str) + "_" + frame["psu"].astype(str)).to_numpy()


def proportional_stratum_sample(
    frame: pd.DataFrame,
    candidate_positions: np.ndarray,
    sample_size: int,
    rng: np.random.Generator,
) -> np.ndarray:
    """Outcome-blind row sample with proportional allocation across strata."""
    candidate_positions = np.asarray(candidate_positions, dtype=int)
    if sample_size >= len(candidate_positions):
        return candidate_positions.copy()
    strata = frame.iloc[candidate_positions]["stratum"].astype(str).to_numpy()
    unique, counts = np.unique(strata, return_counts=True)
    raw = sample_size * counts / counts.sum()
    allocation = np.floor(raw).astype(int)
    allocation = np.minimum(allocation, counts)
    remaining = int(sample_size - allocation.sum())
    fractional = raw - np.floor(raw)
    while remaining > 0:
        eligible = np.flatnonzero(allocation < counts)
        if not len(eligible):
            raise RuntimeError("Unable to allocate the requested update sample.")
        jitter = rng.uniform(0.0, 1e-9, size=len(eligible))
        choice = eligible[np.argmax(fractional[eligible] + jitter)]
        allocation[choice] += 1
        fractional[choice] = -1.0
        remaining -= 1

    selected: list[np.ndarray] = []
    for value, n_take in zip(unique, allocation, strict=True):
        if n_take <= 0:
            continue
        eligible_positions = candidate_positions[strata == value]
        selected.append(rng.choice(eligible_positions, size=int(n_take), replace=False))
    output = np.concatenate(selected)
    rng.shuffle(output)
    if len(output) != sample_size:
        raise RuntimeError(f"Expected {sample_size} update rows, obtained {len(output)}.")
    return output


def fit_intercept_update(score: np.ndarray, outcome: np.ndarray, weight: np.ndarray) -> tuple[float, float]:
    p = np.clip(np.asarray(score, dtype=float), 1e-6, 1 - 1e-6)
    y = np.asarray(outcome, dtype=float)
    w = normalized_weights(weight)
    lp = logit(p)
    alpha = 0.0
    for _ in range(200):
        mean = expit(lp + alpha)
        information = np.sum(w * mean * (1 - mean))
        if information <= 1e-12:
            raise RuntimeError("Intercept update has zero information.")
        step = np.sum(w * (y - mean)) / information
        alpha += step
        if abs(step) < 1e-10:
            break
    return float(alpha), 1.0


def fit_logistic_update(score: np.ndarray, outcome: np.ndarray, weight: np.ndarray) -> tuple[float, float]:
    p = np.clip(np.asarray(score, dtype=float), 1e-6, 1 - 1e-6)
    y = np.asarray(outcome, dtype=float)
    w = normalized_weights(weight)
    x = np.column_stack([np.ones(len(p)), logit(p)])
    coefficient = np.array([0.0, 1.0], dtype=float)
    ridge = np.diag([1e-10, 1e-10])
    for _ in range(200):
        mean = expit(x @ coefficient)
        working_weight = w * mean * (1 - mean)
        information = x.T @ (working_weight[:, None] * x) + ridge
        score_vector = x.T @ (w * (y - mean))
        step = np.linalg.solve(information, score_vector)
        coefficient += step
        if np.max(np.abs(step)) < 1e-10:
            break
    if not np.isfinite(coefficient).all():
        raise RuntimeError("Logistic recalibration returned a nonfinite coefficient.")
    return float(coefficient[0]), float(coefficient[1])


def apply_update(score: np.ndarray, coefficient: tuple[float, float]) -> np.ndarray:
    alpha, beta = coefficient
    lp = logit(np.clip(np.asarray(score, dtype=float), 1e-6, 1 - 1e-6))
    return np.clip(expit(alpha + beta * lp), 1e-8, 1 - 1e-8)


def weighted_operating_metrics(
    outcome: np.ndarray,
    score: np.ndarray,
    weight: np.ndarray,
    threshold: float,
) -> tuple[float, float, float]:
    selected = np.asarray(score, dtype=float) >= float(threshold)
    y = np.asarray(outcome, dtype=float)
    w = np.asarray(weight, dtype=float)
    positives = np.sum(w * y)
    selected_weight = np.sum(w * selected)
    true_positives = np.sum(w * y * selected)
    sensitivity = true_positives / positives if positives > 0 else np.nan
    ppv = true_positives / selected_weight if selected_weight > 0 else np.nan
    referral = np.average(selected, weights=w)
    return float(sensitivity), float(ppv), float(referral)


def maintenance_metrics(
    outcome: np.ndarray,
    score: np.ndarray,
    weight: np.ndarray,
    frozen_threshold: float,
) -> dict[str, float]:
    base = performance_metrics(outcome, score, weight, frozen_threshold)
    p = np.clip(np.asarray(score, dtype=float), 1e-8, 1 - 1e-8)
    y = np.asarray(outcome, dtype=float)
    w = np.asarray(weight, dtype=float)
    log_loss_value = -np.average(y * np.log(p) + (1 - y) * np.log(1 - p), weights=w)
    capacity_threshold = weighted_quantile(p, 0.90, w)
    capacity_sensitivity, capacity_ppv, capacity_fraction = weighted_operating_metrics(
        y, p, w, capacity_threshold
    )
    base.update(
        {
            "weighted_log_loss": float(log_loss_value),
            "absolute_calibration_intercept_error": float(abs(base["calibration_intercept"])),
            "absolute_calibration_slope_error": float(abs(base["calibration_slope"] - 1.0)),
            "capacity_threshold": float(capacity_threshold),
            "top10_sensitivity": capacity_sensitivity,
            "top10_positive_predictive_value": capacity_ppv,
            "top10_referral_fraction": capacity_fraction,
            "close_calibration": float(
                abs(base["calibration_intercept"]) <= 0.10
                and 0.90 <= base["calibration_slope"] <= 1.10
            ),
        }
    )
    return base


def update_sample_diagnostic(
    wave: pd.DataFrame,
    positions: np.ndarray,
    target_year: int,
    repeat: int,
    fold: int,
    budget: int,
) -> dict[str, float | int]:
    selected = wave.iloc[positions]
    weights = selected["weight"].to_numpy(dtype=float)
    outcome = selected["attempt"].to_numpy(dtype=float)
    return {
        "target_year": target_year,
        "repeat": repeat,
        "fold": fold,
        "budget": budget,
        "update_n": int(len(selected)),
        "update_events_unweighted": int(outcome.sum()),
        "update_prevalence_weighted": float(np.average(outcome, weights=weights)),
        "update_effective_n": float(effective_sample_size(weights)),
        "update_psus": int(selected[["stratum", "psu"]].drop_duplicates().shape[0]),
        "update_strata": int(selected["stratum"].nunique()),
    }


def summarize_runs(runs: pd.DataFrame) -> pd.DataFrame:
    identity = ["target_year", "budget", "budget_label", "strategy"]
    metric_columns = [
        column
        for column in runs.columns
        if column not in identity + ["repeat"] and pd.api.types.is_numeric_dtype(runs[column])
    ]
    rows: list[dict[str, object]] = []
    for keys, group in runs.groupby(identity, dropna=False, sort=True):
        record = dict(zip(identity, keys, strict=True))
        record["runs"] = int(group["repeat"].nunique())
        for metric in metric_columns:
            values = group[metric].dropna().to_numpy(dtype=float)
            record[f"{metric}_median"] = float(np.median(values)) if len(values) else np.nan
            record[f"{metric}_p05"] = float(np.quantile(values, 0.05)) if len(values) else np.nan
            record[f"{metric}_p95"] = float(np.quantile(values, 0.95)) if len(values) else np.nan
        close_values = group["close_calibration"].dropna().to_numpy(dtype=float)
        record["close_calibration_rate"] = (
            float(np.mean(close_values)) if len(close_values) else np.nan
        )
        rows.append(record)
    return pd.DataFrame(rows)


def paired_differences(runs: pd.DataFrame) -> tuple[pd.DataFrame, pd.DataFrame]:
    static = runs[runs["strategy"].eq("static")].drop_duplicates("target_year")
    metrics = [
        "absolute_calibration_intercept_error",
        "absolute_calibration_slope_error",
        "brier",
        "weighted_log_loss",
        "auroc",
        "auprc",
        "top10_sensitivity",
        "top10_positive_predictive_value",
    ]
    base = static.set_index("target_year")[metrics]
    updated = runs[~runs["strategy"].eq("static")].copy()
    for metric in metrics:
        updated[f"delta_{metric}_vs_static"] = updated[metric] - updated["target_year"].map(base[metric])
    delta_columns = [column for column in updated.columns if column.startswith("delta_")]
    rows: list[dict[str, object]] = []
    identity = ["target_year", "budget", "budget_label", "strategy"]
    for keys, group in updated.groupby(identity, dropna=False, sort=True):
        record = dict(zip(identity, keys, strict=True))
        record["runs"] = int(group["repeat"].nunique())
        for metric in delta_columns:
            values = group[metric].dropna().to_numpy(dtype=float)
            record[f"{metric}_median"] = float(np.median(values)) if len(values) else np.nan
            record[f"{metric}_p05"] = float(np.quantile(values, 0.05)) if len(values) else np.nan
            record[f"{metric}_p95"] = float(np.quantile(values, 0.95)) if len(values) else np.nan
        rows.append(record)
    return updated, pd.DataFrame(rows)


def run_target_wave(
    wave: pd.DataFrame,
    static_score: np.ndarray,
    target_year: int,
    budgets: list[int],
    repeats: int,
    c_value: float,
    frozen_threshold: float,
) -> tuple[list[dict[str, object]], list[dict[str, object]]]:
    y = wave["attempt"].to_numpy(dtype=int)
    w = wave["weight"].to_numpy(dtype=float)
    groups = design_groups(wave)
    result_rows: list[dict[str, object]] = []
    diagnostic_rows: list[dict[str, object]] = []

    static = {
        "target_year": target_year,
        "repeat": -1,
        "budget": 0,
        "budget_label": "none",
        "strategy": "static",
        **maintenance_metrics(y, static_score, w, frozen_threshold),
    }
    result_rows.append(static)

    for repeat in range(repeats):
        splitter = StratifiedGroupKFold(
            n_splits=N_SPLITS,
            shuffle=True,
            random_state=SEED + target_year * 100 + repeat,
        )
        folds = list(splitter.split(np.zeros(len(wave)), y, groups))
        for train, test in folds:
            if set(groups[train]).intersection(set(groups[test])):
                raise RuntimeError("Update/evaluation PSU leakage detected.")

        full_predictions = {
            "intercept": np.full(len(wave), np.nan),
            "logistic": np.full(len(wave), np.nan),
            "refit": np.full(len(wave), np.nan),
        }
        budget_predictions = {
            budget: {
                "intercept": np.full(len(wave), np.nan),
                "logistic": np.full(len(wave), np.nan),
                "refit": np.full(len(wave), np.nan),
            }
            for budget in budgets
        }

        for fold, (train, test) in enumerate(folds):
            # Full outer-training benchmark.
            full_intercept = fit_intercept_update(static_score[train], y[train], w[train])
            full_logistic = fit_logistic_update(static_score[train], y[train], w[train])
            full_predictions["intercept"][test] = apply_update(static_score[test], full_intercept)
            full_predictions["logistic"][test] = apply_update(static_score[test], full_logistic)
            full_model = logistic_pipeline(SCI_PREDICTORS, c_value)
            full_model.fit(
                wave.iloc[train][SCI_PREDICTORS],
                y[train],
                model__sample_weight=normalized_weights(w[train]),
            )
            full_predictions["refit"][test] = full_model.predict_proba(
                wave.iloc[test][SCI_PREDICTORS]
            )[:, 1]

            for budget_index, budget in enumerate(budgets):
                rng = np.random.default_rng(
                    SEED + target_year * 1_000_000 + repeat * 10_000 + fold * 100 + budget_index
                )
                update = proportional_stratum_sample(wave, train, budget, rng)
                diagnostic_rows.append(
                    update_sample_diagnostic(wave, update, target_year, repeat, fold, budget)
                )
                intercept = fit_intercept_update(static_score[update], y[update], w[update])
                logistic = fit_logistic_update(static_score[update], y[update], w[update])
                budget_predictions[budget]["intercept"][test] = apply_update(
                    static_score[test], intercept
                )
                budget_predictions[budget]["logistic"][test] = apply_update(
                    static_score[test], logistic
                )
                refitted = logistic_pipeline(SCI_PREDICTORS, c_value)
                refitted.fit(
                    wave.iloc[update][SCI_PREDICTORS],
                    y[update],
                    model__sample_weight=normalized_weights(w[update]),
                )
                budget_predictions[budget]["refit"][test] = refitted.predict_proba(
                    wave.iloc[test][SCI_PREDICTORS]
                )[:, 1]

        for strategy, prediction in full_predictions.items():
            if np.isnan(prediction).any():
                raise RuntimeError(f"Full benchmark left unscored rows: {strategy}.")
            result_rows.append(
                {
                    "target_year": target_year,
                    "repeat": repeat,
                    "budget": -1,
                    "budget_label": "full_outer_training",
                    "strategy": strategy,
                    **maintenance_metrics(y, prediction, w, frozen_threshold),
                }
            )
        for budget, strategies in budget_predictions.items():
            for strategy, prediction in strategies.items():
                if np.isnan(prediction).any():
                    raise RuntimeError(
                        f"Budget {budget} left unscored rows for {strategy}."
                    )
                result_rows.append(
                    {
                        "target_year": target_year,
                        "repeat": repeat,
                        "budget": budget,
                        "budget_label": str(budget),
                        "strategy": strategy,
                        **maintenance_metrics(y, prediction, w, frozen_threshold),
                    }
                )
        report(f"{target_year}: completed repetition {repeat + 1}/{repeats}")
    return result_rows, diagnostic_rows


def rolling_source_metrics(
    data: pd.DataFrame,
    frozen_model,
    c_value: float,
    frozen_threshold: float,
) -> pd.DataFrame:
    rows: list[dict[str, object]] = []
    comparisons = [(2015, year) for year in TARGET_YEARS] + [
        (2017, 2019),
        (2019, 2021),
        (2021, 2023),
    ]
    for source_year, target_year in comparisons:
        source = data[(data["year"].eq(source_year)) & (data["attempt_observed"].eq(1))].copy()
        target = data[(data["year"].eq(target_year)) & (data["attempt_observed"].eq(1))].copy()
        if source_year == SOURCE_YEAR:
            model = frozen_model
            source_threshold = frozen_threshold
        else:
            model = logistic_pipeline(SCI_PREDICTORS, c_value)
            model.fit(
                source[SCI_PREDICTORS],
                source["attempt"].astype(int),
                model__sample_weight=normalized_weights(
                    source["weight"].to_numpy(dtype=float)
                ),
            )
            source_prediction = model.predict_proba(source[SCI_PREDICTORS])[:, 1]
            source_threshold = weighted_quantile(
                source_prediction, 0.90, source["weight"].to_numpy(dtype=float)
            )
        prediction = model.predict_proba(target[SCI_PREDICTORS])[:, 1]
        metrics = maintenance_metrics(
            target["attempt"].to_numpy(dtype=int),
            prediction,
            target["weight"].to_numpy(dtype=float),
            source_threshold,
        )
        rows.append(
            {
                "source_year": source_year,
                "target_year": target_year,
                "source_n": int(len(source)),
                "source_events": int(source["attempt"].sum()),
                "target_n": int(len(target)),
                "target_events": int(target["attempt"].sum()),
                "source_top10_threshold": float(source_threshold),
                **metrics,
            }
        )
    return pd.DataFrame(rows).sort_values(["target_year", "source_year"])


def fit_frozen_source(
    observed: pd.DataFrame,
) -> tuple[object, float, float, pd.DataFrame, dict[str, float]]:
    """Tune, cross-fit, and finally fit the protected-attribute-free source score."""
    source = observed[observed["year"].eq(SOURCE_YEAR)].reset_index(drop=True)
    y = source["attempt"].to_numpy(dtype=int)
    raw_weight = source["weight"].to_numpy(dtype=float)
    fit_weight = normalized_weights(raw_weight)
    groups = design_groups(source)

    tuned = tune_logistic(
        source,
        SCI_PREDICTORS,
        y,
        fit_weight,
        groups,
    )
    c_value = float(tuned.parameters["C"])

    prediction = np.full(len(source), np.nan)
    splitter = StratifiedGroupKFold(
        n_splits=N_SPLITS,
        shuffle=True,
        random_state=SEED,
    )
    for train, test in splitter.split(np.zeros(len(source)), y, groups):
        model = logistic_pipeline(SCI_PREDICTORS, c_value)
        model.fit(
            source.iloc[train][SCI_PREDICTORS],
            y[train],
            model__sample_weight=normalized_weights(raw_weight[train]),
        )
        prediction[test] = model.predict_proba(source.iloc[test][SCI_PREDICTORS])[:, 1]
    if np.isnan(prediction).any():
        raise RuntimeError("Source cross-fitting left unscored records.")

    frozen_threshold = weighted_quantile(prediction, 0.90, raw_weight)
    source_metrics = maintenance_metrics(y, prediction, raw_weight, frozen_threshold)

    frozen_model = logistic_pipeline(SCI_PREDICTORS, c_value)
    frozen_model.fit(
        source[SCI_PREDICTORS],
        y,
        model__sample_weight=fit_weight,
    )
    prediction_frame = source[
        ["record_id", "year", "attempt", "weight", "stratum", "psu"]
    ].copy()
    prediction_frame["frozen_score"] = prediction
    return frozen_model, c_value, float(frozen_threshold), prediction_frame, source_metrics


def main() -> None:
    parser = argparse.ArgumentParser()
    parser.add_argument("--repeats", type=int, default=DEFAULT_REPEATS)
    parser.add_argument("--budgets", type=int, nargs="+", default=DEFAULT_BUDGETS)
    parser.add_argument("--years", type=int, nargs="+", default=TARGET_YEARS)
    parser.add_argument("--output", type=Path, default=DEFAULT_OUT)
    parser.add_argument("--smoke", action="store_true")
    args = parser.parse_args()
    if args.smoke:
        args.repeats = 2
        args.budgets = [250, 1000]
        args.years = [2017]
        args.output = ROOT / "results" / "sci_maintenance_smoke"

    output = args.output
    output.mkdir(parents=True, exist_ok=True)
    data = pd.read_parquet(DATA_PATH)
    observed = data[data["attempt_observed"].eq(1)].copy()
    if int(observed["attempt"].isna().sum()) != 0:
        raise RuntimeError("Observed-outcome analytic population contains missing attempts.")
    report(
        f"Observed-outcome analytic population: n={len(observed):,}, "
        f"events={int(observed['attempt'].sum()):,}"
    )
    frozen_model, c_value, frozen_threshold, source_predictions, source_metrics = (
        fit_frozen_source(observed)
    )
    report(
        f"Frozen source score: {len(SCI_PREDICTORS)} predictors, C={c_value:g}, "
        f"cross-fitted AUROC={source_metrics['auroc']:.3f}"
    )

    all_results: list[dict[str, object]] = []
    all_diagnostics: list[dict[str, object]] = []
    for year in args.years:
        wave = observed[observed["year"].eq(year)].reset_index(drop=True)
        if wave.empty:
            raise RuntimeError(f"Target wave {year} is empty.")
        static_score = frozen_model.predict_proba(wave[SCI_PREDICTORS])[:, 1]
        rows, diagnostics = run_target_wave(
            wave,
            static_score,
            year,
            sorted(set(args.budgets)),
            args.repeats,
            c_value,
            frozen_threshold,
        )
        all_results.extend(rows)
        all_diagnostics.extend(diagnostics)

    runs = pd.DataFrame(all_results)
    diagnostics = pd.DataFrame(all_diagnostics)
    summary = summarize_runs(runs)
    paired, paired_summary = paired_differences(runs)
    diagnostic_summary = (
        diagnostics.groupby(["target_year", "budget"], as_index=False)
        .agg(
            fits=("update_n", "size"),
            update_n_median=("update_n", "median"),
            update_events_median=("update_events_unweighted", "median"),
            update_events_p05=("update_events_unweighted", lambda x: x.quantile(0.05)),
            update_events_p95=("update_events_unweighted", lambda x: x.quantile(0.95)),
            update_effective_n_median=("update_effective_n", "median"),
            update_psus_median=("update_psus", "median"),
            update_strata_median=("update_strata", "median"),
        )
        .sort_values(["target_year", "budget"])
    )
    rolling = rolling_source_metrics(data, frozen_model, c_value, frozen_threshold)

    runs.to_csv(output / "maintenance_learning_curve_runs.csv", index=False)
    runs.to_parquet(output / "maintenance_learning_curve_runs.parquet", index=False)
    summary.to_csv(output / "maintenance_learning_curve_summary.csv", index=False)
    paired.to_csv(output / "maintenance_paired_differences.csv", index=False)
    paired_summary.to_csv(output / "maintenance_paired_differences_summary.csv", index=False)
    diagnostics.to_csv(output / "maintenance_update_sample_diagnostics.csv", index=False)
    diagnostic_summary.to_csv(output / "maintenance_update_sample_diagnostics_summary.csv", index=False)
    rolling.to_csv(output / "rolling_source_validation.csv", index=False)
    source_predictions.to_parquet(output / "source_oof_predictions.parquet", index=False)
    joblib.dump(frozen_model, output / "frozen_source_model.joblib")
    run_metadata = {
        "created": time.strftime("%Y-%m-%d %H:%M:%S"),
        "seed": SEED,
        "source_year": SOURCE_YEAR,
        "target_years": args.years,
        "outer_splits": N_SPLITS,
        "repeats": args.repeats,
        "budgets": sorted(set(args.budgets)),
        "strategies": ["static", "intercept", "logistic", "refit"],
        "analytic_n": int(len(observed)),
        "analytic_events": int(observed["attempt"].sum()),
        "c_value": c_value,
        "frozen_threshold": frozen_threshold,
        "predictors": SCI_PREDICTORS,
        "excluded_identity_attributes": [
            "sex",
            "race_ethnicity",
            "sexual_identity_primary",
        ],
        "source_cross_fitted_metrics": source_metrics,
        "weight_scaling": "normalized to the fitted sample size for every penalized fit",
        "companion_overlap_exclusions": [
            "missing-outcome estimands",
            "MAR or MNAR sensitivity",
            "subgroup comparisons",
            "group calibration or fairness",
        ],
    }
    (output / "run_metadata.json").write_text(
        json.dumps(run_metadata, indent=2), encoding="utf-8"
    )
    report(f"Saved independent SCI maintenance results to {output}")


if __name__ == "__main__":
    main()
```
