# Supplementary material for: Temporal maintenance of an adolescent suicide attempt screening score in national surveys: An observational study
Source: Medicine (Baltimore). 2026 Aug 28;105(35):e50513. doi: 10.1097/MD.0000000000050513 (PMC13529099; doi:10.1097/MD.0000000000050513)
Supplement: Supplementary file 8 [file medi-105-e50513-s008.docx]

Supplemental Digital Content 1

**Supporting tables and figures**

**Temporal maintenance of an adolescent suicide attempt screening score in national surveys: An observational study**

First author: Xiaotian Ma

**Table S1. Survey items**

| **Concept** | **Year** | **Source item** | **Source label** |
| --- | --- | --- | --- |
| grade | 2015 | Q3 | In what grade are you |
| safety_absence | 2015 | Q16 | Safety concerns at school |
| weapon_threat | 2015 | Q17 | Threatened at school |
| fight_school | 2015 | Q20 | Physical fighting at school |
| forced_sex | 2015 | Q21 | Forced sexual intercourse |
| bullying_school | 2015 | Q24 | Bullying at school |
| electronic_bullying | 2015 | Q25 | Electronic bullying |
| sad_hopeless | 2015 | Q26 | Sad or hopeless |
| current_cigarette | 2015 | Q33 | Current cigarette use |
| current_vaping | 2015 | Q40 | Current electronic vapor product use |
| current_alcohol | 2015 | Q43 | Current alcohol use |
| current_marijuana | 2015 | Q49 | Current marijuana use |
| no_physical_activity | 2015 | Q80 | Physical activity >= 5 days |
| short_sleep | 2015 | Q88 | Hours of sleep on school night |
| grade | 2017 | Q3 | In what grade are you |
| safety_absence | 2017 | Q15 | Safety concerns at school |
| weapon_threat | 2017 | Q16 | Threatened at school |
| fight_school | 2017 | Q18 | Physical fighting at school |
| forced_sex | 2017 | Q19 | Ever been physically forced sexual intercourse |
| bullying_school | 2017 | Q23 | Bullying at school |
| electronic_bullying | 2017 | Q24 | Electronic bullying |
| sad_hopeless | 2017 | Q25 | Sad or hopeless |
| current_cigarette | 2017 | Q32 | Current cigarette use |
| current_vaping | 2017 | Q35 | Current electronic vapor product use |
| current_alcohol | 2017 | Q42 | Current alcohol use |
| current_marijuana | 2017 | Q48 | Current marijuana use |
| no_physical_activity | 2017 | Q79 | Physical activity >= 5 days |
| short_sleep | 2017 | Q88 | Hours of sleep on school night |
| grade | 2019 | Q3 | In what grade are you |
| safety_absence | 2019 | Q15 | Safety concerns at school |
| weapon_threat | 2019 | Q16 | Threatened at school |
| fight_school | 2019 | Q18 | Physical fighting at school |
| forced_sex | 2019 | Q19 | Ever been physically forced sexual intercourse |
| bullying_school | 2019 | Q23 | Bullying at school |
| electronic_bullying | 2019 | Q24 | Electronic bullying |
| sad_hopeless | 2019 | Q25 | Sad or hopeless |
| current_cigarette | 2019 | Q32 | Current cigarette use |
| current_vaping | 2019 | Q35 | Current electronic vapor product use |
| current_alcohol | 2019 | Q41 | Current alcohol use |
| current_marijuana | 2019 | Q47 | Current marijuana use |
| no_physical_activity | 2019 | Q78 | Physical activity >= 5 days |
| short_sleep | 2019 | Q88 | Hours of sleep on school night |
| grade | 2021 | Q3 | In what grade are you |
| safety_absence | 2021 | Q14 | Safety concerns at school |
| weapon_threat | 2021 | Q15 | Threatened at school |
| fight_school | 2021 | Q17 | Physical fighting at school |
| forced_sex | 2021 | Q19 | Ever been physically forced sexual intercourse |
| bullying_school | 2021 | Q23 | Bullied at school |
| electronic_bullying | 2021 | Q24 | Electronic bullying |
| sad_hopeless | 2021 | Q25 | Sad or hopeless |
| current_cigarette | 2021 | Q32 | Current cigarette use |
| current_vaping | 2021 | Q35 | Current electronic vapor product use |
| current_alcohol | 2021 | Q41 | Current alcohol use |
| current_marijuana | 2021 | Q47 | Current marijuana use |
| no_physical_activity | 2021 | Q77 | Physical activity >= 5 days |
| short_sleep | 2021 | Q86 | Hours of sleep on school night |
| grade | 2023 | Q3 | In what grade are you |
| safety_absence | 2023 | Q14 | Safety concerns at school |
| weapon_threat | 2023 | Q15 | Threatened at school |
| fight_school | 2023 | Q17 | Physical fighting at school |
| forced_sex | 2023 | Q19 | Ever been physically forced sexual intercourse |
| bullying_school | 2023 | Q24 | Bullied at school |
| electronic_bullying | 2023 | Q25 | Electronic bullying |
| sad_hopeless | 2023 | Q26 | Sad or hopeless |
| current_cigarette | 2023 | Q33 | Current cigarette use |
| current_vaping | 2023 | Q36 | Current electronic vapor product use |
| current_alcohol | 2023 | Q42 | Current alcohol use |
| current_marijuana | 2023 | Q48 | Current marijuana use |
| no_physical_activity | 2023 | Q76 | Physical activity >= 5 days |
| short_sleep | 2023 | Q85 | Hours of sleep on school night |

Source item gives the question number in the corresponding national survey.

**Table S2. Score terms**

| **Model term** | **Coefficient** | **Exponentiated coefficient** |
| --- | --- | --- |
| grade_grade_10 | 0.103 | 1.109 |
| grade_grade_11 | −0.160 | 0.852 |
| grade_grade_12 | −0.426 | 0.653 |
| grade_grade_9 | 0.139 | 1.150 |
| grade_missing | 0.228 | 1.256 |
| grade_ungraded_other | 0.058 | 1.059 |
| safety_absence_missing | −0.200 | 0.819 |
| safety_absence_no | −0.104 | 0.901 |
| safety_absence_yes | 0.245 | 1.278 |
| weapon_threat_school_missing | 0.191 | 1.211 |
| weapon_threat_school_no | −0.346 | 0.707 |
| weapon_threat_school_yes | 0.097 | 1.102 |
| physical_fight_school_missing | 0.192 | 1.212 |
| physical_fight_school_no | −0.377 | 0.686 |
| physical_fight_school_yes | 0.126 | 1.135 |
| forced_sex_missing | −0.092 | 0.912 |
| forced_sex_no | −0.406 | 0.666 |
| forced_sex_yes | 0.440 | 1.552 |
| bullying_school_missing | 0.025 | 1.025 |
| bullying_school_no | −0.301 | 0.740 |
| bullying_school_yes | 0.219 | 1.244 |
| electronic_bullying_missing | 0.341 | 1.406 |
| electronic_bullying_no | −0.458 | 0.633 |
| electronic_bullying_yes | 0.059 | 1.061 |
| sad_hopeless_missing | 0.186 | 1.205 |
| sad_hopeless_no | −1.085 | 0.338 |
| sad_hopeless_yes | 0.841 | 2.319 |
| current_cigarette_missing | 0.252 | 1.287 |
| current_cigarette_no | −0.383 | 0.682 |
| current_cigarette_yes | 0.073 | 1.075 |
| current_vaping_missing | 0.007 | 1.007 |
| current_vaping_no | −0.002 | 0.998 |
| current_vaping_yes | −0.064 | 0.938 |
| current_alcohol_missing | 0.194 | 1.214 |
| current_alcohol_no | −0.345 | 0.709 |
| current_alcohol_yes | 0.093 | 1.098 |
| current_marijuana_missing | 0.160 | 1.174 |
| current_marijuana_no | −0.286 | 0.751 |
| current_marijuana_yes | 0.068 | 1.070 |
| no_physical_activity_missing | −0.122 | 0.885 |
| no_physical_activity_no | −0.099 | 0.905 |
| no_physical_activity_yes | 0.163 | 1.177 |
| short_sleep_missing | −0.038 | 0.962 |
| short_sleep_no | 0.002 | 1.002 |
| short_sleep_yes | −0.021 | 0.979 |

Coefficients are penalized predictive model terms and should not be interpreted as causal effect estimates.

**Table S3. Rolling validation**

| **Source year** | **Target year** | **Source n** | **Source attempts** | **Target n** | **Target attempts** | **AUROC** | **Brier** | **Calibration intercept** | **Calibration slope** | **Observed/expected** | **Close calibration** |
| --- | --- | --- | --- | --- | --- | --- | --- | --- | --- | --- | --- |
| 2015 | 2017 | 12,567 | 1,203 | 10,686 | 837 | 0.860 | 0.056 | −0.266 | 0.996 | 0.825 | No |
| 2015 | 2019 | 12,567 | 1,203 | 10,520 | 1,067 | 0.856 | 0.066 | −0.115 | 0.993 | 0.921 | No |
| 2017 | 2019 | 10,686 | 837 | 10,520 | 1,067 | 0.854 | 0.066 | 0.044 | 0.975 | 1.032 | Yes |
| 2015 | 2021 | 12,567 | 1,203 | 15,573 | 1,753 | 0.852 | 0.072 | 0.030 | 1.013 | 1.021 | Yes |
| 2019 | 2021 | 10,520 | 1,067 | 15,573 | 1,753 | 0.854 | 0.072 | 0.134 | 1.037 | 1.102 | No |
| 2015 | 2023 | 12,567 | 1,203 | 19,336 | 1,995 | 0.860 | 0.068 | −0.148 | 1.009 | 0.902 | No |
| 2021 | 2023 | 15,573 | 1,753 | 19,336 | 1,995 | 0.865 | 0.067 | −0.157 | 1.004 | 0.899 | No |

AUROC = area under the receiver operating characteristic curve. Observed/expected compares weighted observed and predicted risk.

**Table S4. Update samples**

| **Target year** | **Budget** | **Fits** | **Median n** | **Median attempts** | **Attempts 5th percentile** | **Attempts 95th percentile** | **Median effective n** | **Median PSUs** | **Median strata** |
| --- | --- | --- | --- | --- | --- | --- | --- | --- | --- |
| 2017 | 250 | 250 | 250 | 19 | 12 | 26 | 160.121 | 40 | 16 |
| 2017 | 500 | 250 | 500 | 39 | 30 | 51 | 320.610 | 41 | 16 |
| 2017 | 1,000 | 250 | 1,000 | 79 | 66 | 93 | 639.136 | 41 | 16 |
| 2017 | 2,000 | 250 | 2,000 | 156 | 140 | 176 | 1277.501 | 41 | 16 |
| 2017 | 4,000 | 250 | 4,000 | 314 | 293 | 335 | 2550.862 | 41 | 16 |
| 2019 | 250 | 250 | 250 | 25 | 18 | 34 | 148.948 | 39 | 15 |
| 2019 | 500 | 250 | 500 | 50 | 38 | 60 | 296.675 | 40 | 15 |
| 2019 | 1,000 | 250 | 1,000 | 101 | 87 | 120 | 593.790 | 40 | 15 |
| 2019 | 2,000 | 250 | 2,000 | 205 | 183 | 225 | 1191.971 | 40 | 15 |
| 2019 | 4,000 | 250 | 4,000 | 410 | 362 | 437 | 2378.017 | 40 | 15 |
| 2021 | 250 | 250 | 250 | 27 | 20 | 38 | 121.593 | 43 | 16 |
| 2021 | 500 | 250 | 500 | 56 | 43 | 69 | 244.032 | 45 | 16 |
| 2021 | 1,000 | 250 | 1,000 | 114 | 98 | 131 | 490.216 | 45 | 16 |
| 2021 | 2,000 | 250 | 2,000 | 225 | 200 | 247 | 980.698 | 45 | 16 |
| 2021 | 4,000 | 250 | 4,000 | 451 | 408 | 483 | 1968.236 | 45 | 16 |
| 2023 | 250 | 250 | 250 | 26 | 18 | 34 | 96.962 | 62 | 16 |
| 2023 | 500 | 250 | 500 | 50 | 39 | 63 | 192.046 | 68 | 16 |
| 2023 | 1,000 | 250 | 1,000 | 103 | 88 | 118 | 382.620 | 70 | 16 |
| 2023 | 2,000 | 250 | 2,000 | 205 | 183 | 228 | 759.231 | 70 | 16 |
| 2023 | 4,000 | 250 | 4,000 | 411 | 383 | 439 | 1527.802 | 70 | 16 |

PSU = primary sampling unit. Effective sample size is the Kish weighted effective sample size.

**Table S5. Learning curves**

| **Target year** | **Label budget** | **Strategy** | **AUROC** | **Brier** | **Absolute intercept error** | **Absolute slope error** | **Top 10% sensitivity** | **Top 10% PPV** | **Close calibration rate** |
| --- | --- | --- | --- | --- | --- | --- | --- | --- | --- |
| 2017 | full_outer_training | intercept | 0.860 | 0.055 | 0.002 | 0.007 | 0.480 | 0.354 | 1.000 |
| 2017 | full_outer_training | logistic | 0.860 | 0.055 | 0.002 | 0.005 | 0.482 | 0.355 | 1.000 |
| 2017 | full_outer_training | refit | 0.864 | 0.055 | 0.008 | 0.008 | 0.494 | 0.364 | 1.000 |
| 2017 | none | static | 0.860 | 0.056 | 0.266 | 0.004 | 0.478 | 0.353 | 0.000 |
| 2017 | 250 | intercept | 0.855 | 0.056 | 0.114 | 0.045 | 0.478 | 0.352 | 0.360 |
| 2017 | 250 | logistic | 0.850 | 0.056 | 0.117 | 0.114 | 0.476 | 0.351 | 0.140 |
| 2017 | 250 | refit | 0.820 | 0.060 | 0.118 | 0.332 | 0.407 | 0.300 | 0.020 |
| 2017 | 500 | intercept | 0.858 | 0.055 | 0.079 | 0.023 | 0.480 | 0.354 | 0.680 |
| 2017 | 500 | logistic | 0.855 | 0.056 | 0.087 | 0.063 | 0.479 | 0.353 | 0.440 |
| 2017 | 500 | refit | 0.840 | 0.058 | 0.078 | 0.206 | 0.446 | 0.328 | 0.020 |
| 2017 | 1000 | intercept | 0.859 | 0.055 | 0.041 | 0.012 | 0.480 | 0.353 | 0.880 |
| 2017 | 1000 | logistic | 0.857 | 0.055 | 0.042 | 0.033 | 0.482 | 0.355 | 0.840 |
| 2017 | 1000 | refit | 0.847 | 0.057 | 0.043 | 0.117 | 0.457 | 0.337 | 0.220 |
| 2017 | 2000 | intercept | 0.860 | 0.055 | 0.036 | 0.011 | 0.482 | 0.356 | 1.000 |
| 2017 | 2000 | logistic | 0.859 | 0.055 | 0.035 | 0.033 | 0.482 | 0.355 | 1.000 |
| 2017 | 2000 | refit | 0.854 | 0.056 | 0.031 | 0.042 | 0.473 | 0.348 | 0.920 |
| 2017 | 4000 | intercept | 0.860 | 0.055 | 0.020 | 0.008 | 0.481 | 0.355 | 1.000 |
| 2017 | 4000 | logistic | 0.860 | 0.055 | 0.021 | 0.013 | 0.482 | 0.355 | 1.000 |
| 2017 | 4000 | refit | 0.859 | 0.056 | 0.018 | 0.023 | 0.486 | 0.358 | 1.000 |
| 2019 | full_outer_training | intercept | 0.856 | 0.066 | 0.002 | 0.010 | 0.445 | 0.395 | 1.000 |
| 2019 | full_outer_training | logistic | 0.855 | 0.066 | 0.003 | 0.005 | 0.443 | 0.395 | 1.000 |
| 2019 | full_outer_training | refit | 0.853 | 0.066 | 0.008 | 0.017 | 0.437 | 0.389 | 1.000 |
| 2019 | none | static | 0.856 | 0.066 | 0.115 | 0.007 | 0.449 | 0.400 | 0.000 |
| 2019 | 250 | intercept | 0.852 | 0.067 | 0.084 | 0.042 | 0.436 | 0.388 | 0.560 |
| 2019 | 250 | logistic | 0.848 | 0.068 | 0.092 | 0.093 | 0.434 | 0.387 | 0.320 |
| 2019 | 250 | refit | 0.813 | 0.071 | 0.085 | 0.359 | 0.376 | 0.335 | 0.040 |
| 2019 | 500 | intercept | 0.853 | 0.066 | 0.071 | 0.030 | 0.441 | 0.392 | 0.660 |
| 2019 | 500 | logistic | 0.850 | 0.067 | 0.071 | 0.063 | 0.438 | 0.390 | 0.500 |
| 2019 | 500 | refit | 0.831 | 0.069 | 0.082 | 0.203 | 0.398 | 0.354 | 0.060 |
| 2019 | 1000 | intercept | 0.855 | 0.066 | 0.050 | 0.018 | 0.441 | 0.393 | 0.800 |
| 2019 | 1000 | logistic | 0.854 | 0.066 | 0.050 | 0.035 | 0.440 | 0.391 | 0.760 |
| 2019 | 1000 | refit | 0.842 | 0.068 | 0.046 | 0.080 | 0.416 | 0.370 | 0.540 |
| 2019 | 2000 | intercept | 0.855 | 0.066 | 0.023 | 0.014 | 0.443 | 0.394 | 0.980 |
| 2019 | 2000 | logistic | 0.854 | 0.066 | 0.021 | 0.026 | 0.443 | 0.395 | 0.980 |
| 2019 | 2000 | refit | 0.846 | 0.067 | 0.023 | 0.031 | 0.428 | 0.381 | 0.960 |
| 2019 | 4000 | intercept | 0.855 | 0.066 | 0.020 | 0.010 | 0.445 | 0.396 | 1.000 |
| 2019 | 4000 | logistic | 0.855 | 0.066 | 0.021 | 0.014 | 0.444 | 0.395 | 1.000 |
| 2019 | 4000 | refit | 0.851 | 0.067 | 0.020 | 0.015 | 0.434 | 0.386 | 1.000 |
| 2021 | full_outer_training | intercept | 0.851 | 0.072 | 0.001 | 0.012 | 0.429 | 0.437 | 1.000 |
| 2021 | full_outer_training | logistic | 0.851 | 0.072 | 0.001 | 0.002 | 0.430 | 0.436 | 1.000 |
| 2021 | full_outer_training | refit | 0.856 | 0.071 | 0.018 | 0.015 | 0.445 | 0.452 | 1.000 |
| 2021 | none | static | 0.852 | 0.072 | 0.030 | 0.013 | 0.431 | 0.437 | 1.000 |
| 2021 | 250 | intercept | 0.847 | 0.073 | 0.094 | 0.015 | 0.429 | 0.437 | 0.500 |
| 2021 | 250 | logistic | 0.842 | 0.074 | 0.091 | 0.110 | 0.426 | 0.433 | 0.240 |
| 2021 | 250 | refit | 0.816 | 0.077 | 0.096 | 0.259 | 0.396 | 0.403 | 0.000 |
| 2021 | 500 | intercept | 0.849 | 0.072 | 0.068 | 0.007 | 0.431 | 0.438 | 0.620 |
| 2021 | 500 | logistic | 0.847 | 0.073 | 0.071 | 0.067 | 0.431 | 0.439 | 0.480 |
| 2021 | 500 | refit | 0.835 | 0.075 | 0.069 | 0.126 | 0.412 | 0.419 | 0.160 |
| 2021 | 1000 | intercept | 0.850 | 0.072 | 0.055 | 0.008 | 0.432 | 0.439 | 0.840 |
| 2021 | 1000 | logistic | 0.848 | 0.072 | 0.053 | 0.049 | 0.431 | 0.438 | 0.720 |
| 2021 | 1000 | refit | 0.843 | 0.074 | 0.051 | 0.053 | 0.424 | 0.431 | 0.680 |
| 2021 | 2000 | intercept | 0.851 | 0.072 | 0.040 | 0.007 | 0.429 | 0.436 | 0.960 |
| 2021 | 2000 | logistic | 0.850 | 0.072 | 0.041 | 0.027 | 0.429 | 0.436 | 0.960 |
| 2021 | 2000 | refit | 0.848 | 0.073 | 0.041 | 0.019 | 0.432 | 0.440 | 0.960 |
| 2021 | 4000 | intercept | 0.851 | 0.072 | 0.021 | 0.011 | 0.429 | 0.437 | 1.000 |
| 2021 | 4000 | logistic | 0.850 | 0.072 | 0.022 | 0.018 | 0.430 | 0.437 | 1.000 |
| 2021 | 4000 | refit | 0.853 | 0.072 | 0.026 | 0.022 | 0.441 | 0.449 | 0.980 |
| 2023 | full_outer_training | intercept | 0.859 | 0.068 | 0.004 | 0.005 | 0.447 | 0.422 | 1.000 |
| 2023 | full_outer_training | logistic | 0.859 | 0.068 | 0.005 | 0.005 | 0.446 | 0.422 | 1.000 |
| 2023 | full_outer_training | refit | 0.864 | 0.067 | 0.012 | 0.016 | 0.468 | 0.442 | 1.000 |
| 2023 | none | static | 0.860 | 0.068 | 0.148 | 0.009 | 0.447 | 0.423 | 0.000 |
| 2023 | 250 | intercept | 0.854 | 0.069 | 0.114 | 0.043 | 0.437 | 0.414 | 0.420 |
| 2023 | 250 | logistic | 0.848 | 0.070 | 0.130 | 0.141 | 0.436 | 0.412 | 0.180 |
| 2023 | 250 | refit | 0.818 | 0.074 | 0.113 | 0.169 | 0.385 | 0.364 | 0.120 |
| 2023 | 500 | intercept | 0.857 | 0.068 | 0.104 | 0.014 | 0.442 | 0.418 | 0.460 |
| 2023 | 500 | logistic | 0.855 | 0.069 | 0.097 | 0.056 | 0.443 | 0.419 | 0.400 |
| 2023 | 500 | refit | 0.835 | 0.072 | 0.097 | 0.099 | 0.408 | 0.386 | 0.300 |
| 2023 | 1000 | intercept | 0.858 | 0.068 | 0.055 | 0.011 | 0.443 | 0.419 | 0.760 |
| 2023 | 1000 | logistic | 0.856 | 0.068 | 0.060 | 0.048 | 0.444 | 0.420 | 0.660 |
| 2023 | 1000 | refit | 0.844 | 0.070 | 0.062 | 0.026 | 0.422 | 0.400 | 0.700 |
| 2023 | 2000 | intercept | 0.859 | 0.068 | 0.040 | 0.005 | 0.447 | 0.423 | 0.920 |
| 2023 | 2000 | logistic | 0.858 | 0.068 | 0.040 | 0.035 | 0.447 | 0.423 | 0.860 |
| 2023 | 2000 | refit | 0.853 | 0.069 | 0.038 | 0.041 | 0.437 | 0.413 | 0.840 |
| 2023 | 4000 | intercept | 0.859 | 0.068 | 0.035 | 0.005 | 0.447 | 0.423 | 0.980 |
| 2023 | 4000 | logistic | 0.858 | 0.068 | 0.032 | 0.023 | 0.446 | 0.422 | 0.980 |
| 2023 | 4000 | refit | 0.858 | 0.068 | 0.037 | 0.031 | 0.451 | 0.427 | 0.980 |

AUROC = area under the receiver operating characteristic curve; PPV = positive predictive value. Values are medians across 50 repetitions.

**Table S6. Paired differences**

| **Target year** | **Label budget** | **Strategy** | **Δ absolute intercept error** | **Δ absolute slope error** | **Δ Brier** | **Δ AUROC** | **Δ top 10% sensitivity** | **Δ top 10% PPV** |
| --- | --- | --- | --- | --- | --- | --- | --- | --- |
| 2017 | full_outer_training | intercept | −0.264 | 0.002 | −0.001 | −0.000 | 0.002 | 0.001 |
| 2017 | full_outer_training | logistic | −0.264 | 0.001 | −0.000 | −0.000 | 0.004 | 0.003 |
| 2017 | full_outer_training | refit | −0.258 | 0.004 | −0.000 | 0.004 | 0.015 | 0.011 |
| 2017 | 250 | intercept | −0.152 | 0.041 | 0.000 | −0.005 | −0.000 | −0.000 |
| 2017 | 250 | logistic | −0.149 | 0.110 | 0.001 | −0.010 | −0.002 | −0.002 |
| 2017 | 250 | refit | −0.148 | 0.327 | 0.005 | −0.040 | −0.071 | −0.053 |
| 2017 | 500 | intercept | −0.187 | 0.019 | −0.000 | −0.002 | 0.002 | 0.001 |
| 2017 | 500 | logistic | −0.179 | 0.059 | 0.000 | −0.005 | 0.001 | 0.001 |
| 2017 | 500 | refit | −0.189 | 0.202 | 0.002 | −0.020 | −0.033 | −0.024 |
| 2017 | 1000 | intercept | −0.226 | 0.008 | −0.000 | −0.001 | 0.001 | 0.001 |
| 2017 | 1000 | logistic | −0.225 | 0.029 | −0.000 | −0.003 | 0.003 | 0.002 |
| 2017 | 1000 | refit | −0.223 | 0.113 | 0.001 | −0.013 | −0.021 | −0.016 |
| 2017 | 2000 | intercept | −0.230 | 0.007 | −0.000 | −0.001 | 0.004 | 0.003 |
| 2017 | 2000 | logistic | −0.231 | 0.029 | −0.000 | −0.001 | 0.003 | 0.002 |
| 2017 | 2000 | refit | −0.235 | 0.038 | 0.000 | −0.006 | −0.005 | −0.004 |
| 2017 | 4000 | intercept | −0.246 | 0.003 | −0.001 | −0.000 | 0.002 | 0.002 |
| 2017 | 4000 | logistic | −0.245 | 0.009 | −0.000 | −0.001 | 0.003 | 0.003 |
| 2017 | 4000 | refit | −0.248 | 0.019 | −0.000 | −0.001 | 0.008 | 0.005 |
| 2019 | full_outer_training | intercept | −0.113 | 0.003 | −0.000 | −0.001 | −0.004 | −0.004 |
| 2019 | full_outer_training | logistic | −0.113 | −0.002 | 0.000 | −0.001 | −0.006 | −0.005 |
| 2019 | full_outer_training | refit | −0.107 | 0.009 | 0.000 | −0.003 | −0.012 | −0.010 |
| 2019 | 250 | intercept | −0.031 | 0.035 | 0.001 | −0.004 | −0.013 | −0.012 |
| 2019 | 250 | logistic | −0.023 | 0.085 | 0.002 | −0.009 | −0.015 | −0.013 |
| 2019 | 250 | refit | −0.030 | 0.351 | 0.005 | −0.043 | −0.073 | −0.065 |
| 2019 | 500 | intercept | −0.044 | 0.022 | 0.000 | −0.003 | −0.008 | −0.008 |
| 2019 | 500 | logistic | −0.044 | 0.055 | 0.001 | −0.006 | −0.012 | −0.010 |
| 2019 | 500 | refit | −0.033 | 0.195 | 0.003 | −0.025 | −0.052 | −0.046 |
| 2019 | 1000 | intercept | −0.065 | 0.011 | 0.000 | −0.001 | −0.008 | −0.007 |
| 2019 | 1000 | logistic | −0.065 | 0.028 | 0.000 | −0.003 | −0.010 | −0.008 |
| 2019 | 1000 | refit | −0.069 | 0.073 | 0.002 | −0.015 | −0.033 | −0.030 |
| 2019 | 2000 | intercept | −0.092 | 0.006 | 0.000 | −0.001 | −0.006 | −0.005 |
| 2019 | 2000 | logistic | −0.094 | 0.018 | 0.000 | −0.002 | −0.006 | −0.005 |
| 2019 | 2000 | refit | −0.092 | 0.024 | 0.001 | −0.010 | −0.021 | −0.019 |
| 2019 | 4000 | intercept | −0.096 | 0.003 | −0.000 | −0.001 | −0.004 | −0.004 |
| 2019 | 4000 | logistic | −0.095 | 0.006 | 0.000 | −0.002 | −0.005 | −0.005 |
| 2019 | 4000 | refit | −0.095 | 0.007 | 0.001 | −0.005 | −0.015 | −0.014 |
| 2021 | full_outer_training | intercept | −0.029 | −0.002 | 0.000 | −0.000 | −0.002 | −0.000 |
| 2021 | full_outer_training | logistic | −0.029 | −0.011 | 0.000 | −0.001 | −0.001 | −0.001 |
| 2021 | full_outer_training | refit | −0.012 | 0.002 | −0.001 | 0.004 | 0.013 | 0.015 |
| 2021 | 250 | intercept | 0.064 | 0.002 | 0.001 | −0.004 | −0.002 | −0.000 |
| 2021 | 250 | logistic | 0.061 | 0.096 | 0.002 | −0.009 | −0.005 | −0.004 |
| 2021 | 250 | refit | 0.066 | 0.245 | 0.005 | −0.036 | −0.035 | −0.034 |
| 2021 | 500 | intercept | 0.038 | −0.006 | 0.000 | −0.002 | −0.001 | 0.001 |
| 2021 | 500 | logistic | 0.042 | 0.054 | 0.001 | −0.005 | −0.000 | 0.002 |
| 2021 | 500 | refit | 0.040 | 0.113 | 0.003 | −0.017 | −0.020 | −0.018 |
| 2021 | 1000 | intercept | 0.025 | −0.006 | 0.000 | −0.001 | 0.000 | 0.002 |
| 2021 | 1000 | logistic | 0.024 | 0.035 | 0.000 | −0.003 | −0.001 | 0.001 |
| 2021 | 1000 | refit | 0.022 | 0.040 | 0.002 | −0.008 | −0.007 | −0.006 |
| 2021 | 2000 | intercept | 0.010 | −0.006 | 0.000 | −0.001 | −0.002 | −0.000 |
| 2021 | 2000 | logistic | 0.011 | 0.014 | 0.000 | −0.002 | −0.003 | −0.001 |
| 2021 | 2000 | refit | 0.011 | 0.006 | 0.001 | −0.003 | 0.001 | 0.003 |
| 2021 | 4000 | intercept | −0.008 | −0.002 | 0.000 | −0.000 | −0.002 | −0.000 |
| 2021 | 4000 | logistic | −0.008 | 0.005 | 0.000 | −0.001 | −0.001 | 0.000 |
| 2021 | 4000 | refit | −0.004 | 0.009 | 0.000 | 0.001 | 0.010 | 0.012 |
| 2023 | full_outer_training | intercept | −0.144 | −0.004 | −0.000 | −0.000 | −0.000 | −0.001 |
| 2023 | full_outer_training | logistic | −0.143 | −0.004 | −0.000 | −0.001 | −0.001 | −0.001 |
| 2023 | full_outer_training | refit | −0.135 | 0.007 | −0.001 | 0.005 | 0.021 | 0.019 |
| 2023 | 250 | intercept | −0.034 | 0.034 | 0.001 | −0.006 | −0.010 | −0.009 |
| 2023 | 250 | logistic | −0.018 | 0.133 | 0.002 | −0.012 | −0.011 | −0.011 |
| 2023 | 250 | refit | −0.035 | 0.160 | 0.006 | −0.041 | −0.062 | −0.059 |
| 2023 | 500 | intercept | −0.044 | 0.006 | 0.001 | −0.002 | −0.005 | −0.005 |
| 2023 | 500 | logistic | −0.051 | 0.047 | 0.001 | −0.004 | −0.004 | −0.004 |
| 2023 | 500 | refit | −0.050 | 0.090 | 0.004 | −0.025 | −0.039 | −0.037 |
| 2023 | 1000 | intercept | −0.093 | 0.003 | 0.000 | −0.002 | −0.004 | −0.004 |
| 2023 | 1000 | logistic | −0.088 | 0.039 | 0.001 | −0.003 | −0.003 | −0.003 |
| 2023 | 1000 | refit | −0.086 | 0.017 | 0.002 | −0.015 | −0.025 | −0.023 |
| 2023 | 2000 | intercept | −0.107 | −0.004 | −0.000 | −0.001 | −0.000 | −0.000 |
| 2023 | 2000 | logistic | −0.108 | 0.026 | 0.000 | −0.002 | −0.000 | −0.000 |
| 2023 | 2000 | refit | −0.109 | 0.032 | 0.001 | −0.007 | −0.010 | −0.010 |
| 2023 | 4000 | intercept | −0.113 | −0.004 | −0.000 | −0.001 | −0.000 | −0.000 |
| 2023 | 4000 | logistic | −0.116 | 0.014 | 0.000 | −0.001 | −0.001 | −0.001 |
| 2023 | 4000 | refit | −0.111 | 0.022 | 0.000 | −0.002 | 0.004 | 0.004 |

AUROC = area under the receiver operating characteristic curve; PPV = positive predictive value. Delta values compare each strategy with the static score within identical repetitions.

**Table S7. Survey design diagnostics**

| **Year** | **Strata** | **PSUs** | **Single PSU certainty strata** | **Minimum PSUs per stratum** | **Maximum PSUs per stratum** |
| --- | --- | --- | --- | --- | --- |
| 2015 | 13 | 53 | 0 | 2 | 9 |
| 2017 | 16 | 51 | 1 | 1 | 7 |
| 2019 | 16 | 50 | 3 | 1 | 8 |
| 2021 | 16 | 57 | 0 | 2 | 10 |
| 2023 | 16 | 88 | 0 | 2 | 40 |

PSU = primary sampling unit. Singleton strata were treated as certainty strata in the primary bootstrap.

**Table S8. Source penalty sensitivity**

| **C** | **Year** | **Origin** | **AUROC** | **Brier** | **Calibration intercept** | **Calibration slope** | **Observed/expected** | **Close calibration** |
| --- | --- | --- | --- | --- | --- | --- | --- | --- |
| 0.010 | 2015 | cross_fitted_source | 0.862 | 0.063 | −0.009 | 1.202 | 0.993 | No |
| 0.010 | 2017 | frozen_source | 0.860 | 0.055 | −0.233 | 1.162 | 0.836 | No |
| 0.010 | 2019 | frozen_source | 0.856 | 0.066 | −0.097 | 1.165 | 0.929 | No |
| 0.010 | 2021 | frozen_source | 0.854 | 0.072 | 0.069 | 1.204 | 1.053 | No |
| 0.010 | 2023 | frozen_source | 0.862 | 0.067 | −0.079 | 1.202 | 0.943 | No |
| 0.100 | 2015 | cross_fitted_source | 0.859 | 0.064 | −0.033 | 0.967 | 0.977 | Yes |
| 0.100 | 2017 | frozen_source | 0.860 | 0.056 | −0.266 | 0.996 | 0.825 | No |
| 0.100 | 2019 | frozen_source | 0.856 | 0.066 | −0.115 | 0.993 | 0.921 | No |
| 0.100 | 2021 | frozen_source | 0.852 | 0.072 | 0.030 | 1.013 | 1.021 | Yes |
| 0.100 | 2023 | frozen_source | 0.860 | 0.068 | −0.148 | 1.009 | 0.902 | No |
| 1.000 | 2015 | cross_fitted_source | 0.856 | 0.064 | −0.052 | 0.915 | 0.965 | Yes |
| 1.000 | 2017 | frozen_source | 0.859 | 0.056 | −0.270 | 0.967 | 0.825 | No |
| 1.000 | 2019 | frozen_source | 0.856 | 0.066 | −0.122 | 0.957 | 0.918 | No |
| 1.000 | 2021 | frozen_source | 0.847 | 0.072 | 0.026 | 0.968 | 1.019 | Yes |
| 1.000 | 2023 | frozen_source | 0.858 | 0.068 | −0.170 | 0.961 | 0.890 | No |
| 10.000 | 2015 | cross_fitted_source | 0.855 | 0.065 | −0.057 | 0.903 | 0.962 | Yes |
| 10.000 | 2017 | frozen_source | 0.859 | 0.056 | −0.275 | 0.960 | 0.822 | No |
| 10.000 | 2019 | frozen_source | 0.855 | 0.066 | −0.122 | 0.948 | 0.918 | No |
| 10.000 | 2021 | frozen_source | 0.846 | 0.072 | 0.041 | 0.961 | 1.029 | Yes |
| 10.000 | 2023 | frozen_source | 0.856 | 0.069 | −0.173 | 0.945 | 0.889 | No |

AUROC = area under the receiver operating characteristic curve. C is the inverse ridge penalty; smaller values indicate stronger shrinkage.

**Table S9. Refit penalty sensitivity**

| **Year** | **Budget** | **C** | **Valid calibration runs** | **Calibration failure rate** | **Brier** | **AUROC** | **Absolute intercept error** | **Absolute slope error** | **Delta Brier vs intercept** | **Delta AUROC vs intercept** |
| --- | --- | --- | --- | --- | --- | --- | --- | --- | --- | --- |
| 2017 | 250 | 0.010 | 50.000 | 0.000 | 0.066 | 0.695 | 0.113 | 1.222 | 0.011 | −0.161 |
| 2017 | 250 | 0.100 | 50.000 | 0.000 | 0.060 | 0.820 | 0.118 | 0.332 | 0.005 | −0.034 |
| 2017 | 250 | 1.000 | 50.000 | 0.000 | 0.063 | 0.799 | 0.146 | 0.351 | 0.007 | −0.054 |
| 2017 | 250 | 10.000 | 26.000 | 0.480 | 0.074 | 0.761 | 0.207 | 0.626 | 0.018 | −0.093 |
| 2017 | 1,000 | 0.010 | 50.000 | 0.000 | 0.062 | 0.842 | 0.034 | 1.523 | 0.007 | −0.018 |
| 2017 | 1,000 | 0.100 | 50.000 | 0.000 | 0.057 | 0.847 | 0.043 | 0.117 | 0.002 | −0.012 |
| 2017 | 1,000 | 1.000 | 50.000 | 0.000 | 0.059 | 0.838 | 0.047 | 0.212 | 0.004 | −0.021 |
| 2017 | 1,000 | 10.000 | 50.000 | 0.000 | 0.061 | 0.831 | 0.058 | 0.316 | 0.005 | −0.028 |
| 2019 | 250 | 0.010 | 50.000 | 0.000 | 0.078 | 0.717 | 0.076 | 1.899 | 0.012 | −0.133 |
| 2019 | 250 | 0.100 | 50.000 | 0.000 | 0.071 | 0.813 | 0.085 | 0.359 | 0.004 | −0.038 |
| 2019 | 250 | 1.000 | 50.000 | 0.000 | 0.075 | 0.797 | 0.119 | 0.373 | 0.009 | −0.053 |
| 2019 | 250 | 10.000 | 32.000 | 0.360 | 0.087 | 0.763 | 0.206 | 0.628 | 0.021 | −0.088 |
| 2019 | 1,000 | 0.010 | 50.000 | 0.000 | 0.073 | 0.836 | 0.038 | 1.379 | 0.006 | −0.019 |
| 2019 | 1,000 | 0.100 | 50.000 | 0.000 | 0.068 | 0.842 | 0.046 | 0.080 | 0.002 | −0.013 |
| 2019 | 1,000 | 1.000 | 50.000 | 0.000 | 0.071 | 0.831 | 0.052 | 0.235 | 0.005 | −0.024 |
| 2019 | 1,000 | 10.000 | 50.000 | 0.000 | 0.073 | 0.822 | 0.067 | 0.332 | 0.007 | −0.033 |
| 2021 | 250 | 0.010 | 50.000 | 0.000 | 0.087 | 0.744 | 0.086 | 1.718 | 0.014 | −0.102 |
| 2021 | 250 | 0.100 | 50.000 | 0.000 | 0.077 | 0.816 | 0.096 | 0.259 | 0.004 | −0.028 |
| 2021 | 250 | 1.000 | 50.000 | 0.000 | 0.084 | 0.793 | 0.123 | 0.409 | 0.011 | −0.051 |
| 2021 | 250 | 10.000 | 15.000 | 0.700 | 0.099 | 0.751 | 0.301 | 0.658 | 0.027 | −0.092 |
| 2021 | 1,000 | 0.010 | 50.000 | 0.000 | 0.079 | 0.843 | 0.049 | 1.190 | 0.007 | −0.008 |
| 2021 | 1,000 | 0.100 | 50.000 | 0.000 | 0.074 | 0.843 | 0.051 | 0.053 | 0.002 | −0.007 |
| 2021 | 1,000 | 1.000 | 50.000 | 0.000 | 0.077 | 0.829 | 0.068 | 0.260 | 0.005 | −0.021 |
| 2021 | 1,000 | 10.000 | 50.000 | 0.000 | 0.080 | 0.817 | 0.098 | 0.373 | 0.008 | −0.033 |
| 2023 | 250 | 0.010 | 50.000 | 0.000 | 0.082 | 0.716 | 0.103 | 0.997 | 0.013 | −0.138 |
| 2023 | 250 | 0.100 | 50.000 | 0.000 | 0.074 | 0.818 | 0.113 | 0.169 | 0.005 | −0.034 |
| 2023 | 250 | 1.000 | 50.000 | 0.000 | 0.081 | 0.795 | 0.188 | 0.455 | 0.012 | −0.058 |
| 2023 | 250 | 10.000 | 1.000 | 0.980 | 0.097 | 0.758 | 0.397 | 0.649 | 0.028 | −0.096 |
| 2023 | 1,000 | 0.010 | 50.000 | 0.000 | 0.074 | 0.845 | 0.044 | 1.142 | 0.006 | −0.012 |
| 2023 | 1,000 | 0.100 | 50.000 | 0.000 | 0.070 | 0.844 | 0.062 | 0.026 | 0.002 | −0.013 |
| 2023 | 1,000 | 1.000 | 50.000 | 0.000 | 0.075 | 0.826 | 0.093 | 0.335 | 0.007 | −0.030 |
| 2023 | 1,000 | 10.000 | 50.000 | 0.000 | 0.080 | 0.812 | 0.113 | 0.465 | 0.012 | −0.046 |

AUROC = area under the receiver operating characteristic curve. Values are medians across valid repetitions. Delta values compare refitting with intercept recalibration.

**Table S10. Drift decomposition**

| **Year** | **Total shift** | **Predictor component** | **Conditional component** | **Residual** | **Reconstruction error** | **Predictor share** | **Conditional share** |
| --- | --- | --- | --- | --- | --- | --- | --- |
| 2017 | −0.012 | 0.004 | −0.016 | −0.000 | 0.000 | −0.307 | 1.302 |
| 2019 | 0.003 | 0.011 | −0.008 | −0.000 | 0.000 | 3.235 | −2.212 |
| 2021 | 0.016 | 0.014 | 0.002 | −0.000 | 0.000 | 0.868 | 0.134 |
| 2023 | 0.009 | 0.019 | −0.010 | −0.000 | 0.000 | 2.137 | −1.128 |

Components are descriptive risk differences relative to 2015. Shares may be negative or exceed one when components oppose each other.

**Table S11. Single PSU sensitivity**

| **Year** | **Scheme** | **Metric** | **Analyzed n** | **Singleton strata** | **Estimate** | **CI lower** | **CI upper** | **Bootstrap SE** |
| --- | --- | --- | --- | --- | --- | --- | --- | --- |
| 2017 | certainty | calibration_intercept | 10,686 | 1 | −0.266 | −0.415 | −0.143 | 0.069 |
| 2017 | certainty | calibration_slope | 10,686 | 1 | 0.996 | 0.911 | 1.072 | 0.041 |
| 2017 | certainty | auroc | 10,686 | 1 | 0.860 | 0.841 | 0.879 | 0.010 |
| 2017 | nearest_stratum_collapse | calibration_intercept | 10,686 | 1 | −0.266 | −0.413 | −0.141 | 0.070 |
| 2017 | nearest_stratum_collapse | calibration_slope | 10,686 | 1 | 0.996 | 0.916 | 1.080 | 0.041 |
| 2017 | nearest_stratum_collapse | auroc | 10,686 | 1 | 0.860 | 0.841 | 0.879 | 0.010 |
| 2017 | omit_singleton_strata | calibration_intercept | 10,524 | 1 | −0.264 | −0.425 | −0.144 | 0.070 |
| 2017 | omit_singleton_strata | calibration_slope | 10,524 | 1 | 0.997 | 0.909 | 1.074 | 0.043 |
| 2017 | omit_singleton_strata | auroc | 10,524 | 1 | 0.861 | 0.838 | 0.880 | 0.010 |
| 2019 | certainty | calibration_intercept | 10,520 | 3 | −0.115 | −0.221 | −0.015 | 0.055 |
| 2019 | certainty | calibration_slope | 10,520 | 3 | 0.993 | 0.878 | 1.115 | 0.060 |
| 2019 | certainty | auroc | 10,520 | 3 | 0.856 | 0.831 | 0.880 | 0.012 |
| 2019 | nearest_stratum_collapse | calibration_intercept | 10,520 | 3 | −0.115 | −0.219 | −0.018 | 0.055 |
| 2019 | nearest_stratum_collapse | calibration_slope | 10,520 | 3 | 0.993 | 0.876 | 1.129 | 0.065 |
| 2019 | nearest_stratum_collapse | auroc | 10,520 | 3 | 0.856 | 0.833 | 0.879 | 0.013 |
| 2019 | omit_singleton_strata | calibration_intercept | 9,977 | 3 | −0.134 | −0.241 | −0.017 | 0.060 |
| 2019 | omit_singleton_strata | calibration_slope | 9,977 | 3 | 0.977 | 0.871 | 1.111 | 0.064 |
| 2019 | omit_singleton_strata | auroc | 9,977 | 3 | 0.854 | 0.831 | 0.877 | 0.013 |
| 2023 | certainty | calibration_intercept | 19,336 | 0 | −0.148 | −0.285 | −0.019 | 0.067 |
| 2023 | certainty | calibration_slope | 19,336 | 0 | 1.009 | 0.943 | 1.088 | 0.036 |
| 2023 | certainty | auroc | 19,336 | 0 | 0.860 | 0.845 | 0.875 | 0.008 |

CI = confidence interval; PSU = primary sampling unit; SE = standard error. Each interval used 500 survey bootstrap replicates.

**Table S12. Singleton strata**

| **Year** | **Singleton stratum** | **Merged stratum** |
| --- | --- | --- |
| 2017 | 114 | 113 |
| 2019 | 102 | 101 |
| 2019 | 112 | 111 |
| 2019 | 114 | 113 |

Each singleton stratum was merged with the numerically nearest stratum containing at least two primary sampling units.

**Table S13. Paired comparisons**

| **Year** | **Budget** | **Metric** | **Repetitions** | **Median difference** | **Bootstrap CI lower** | **Bootstrap CI upper** | **Intercept favorable rate** | **Signed rank P** |
| --- | --- | --- | --- | --- | --- | --- | --- | --- |
| 2017 | 250 | brier | 50 | −0.005 | −0.005 | −0.004 | 1.000 | 0.000 |
| 2017 | 250 | auroc | 50 | 0.034 | 0.030 | 0.040 | 1.000 | 0.000 |
| 2017 | 1,000 | brier | 50 | −0.002 | −0.002 | −0.002 | 1.000 | 0.000 |
| 2017 | 1,000 | auroc | 50 | 0.012 | 0.011 | 0.013 | 1.000 | 0.000 |
| 2019 | 250 | brier | 50 | −0.004 | −0.005 | −0.004 | 1.000 | 0.000 |
| 2019 | 250 | auroc | 50 | 0.038 | 0.032 | 0.041 | 1.000 | 0.000 |
| 2019 | 1,000 | brier | 50 | −0.002 | −0.002 | −0.002 | 1.000 | 0.000 |
| 2019 | 1,000 | auroc | 50 | 0.013 | 0.012 | 0.015 | 1.000 | 0.000 |
| 2021 | 250 | brier | 50 | −0.004 | −0.005 | −0.004 | 1.000 | 0.000 |
| 2021 | 250 | auroc | 50 | 0.028 | 0.024 | 0.032 | 1.000 | 0.000 |
| 2021 | 1,000 | brier | 50 | −0.002 | −0.002 | −0.001 | 0.980 | 0.000 |
| 2021 | 1,000 | auroc | 50 | 0.007 | 0.006 | 0.008 | 0.960 | 0.000 |
| 2023 | 250 | brier | 50 | −0.005 | −0.005 | −0.004 | 1.000 | 0.000 |
| 2023 | 250 | auroc | 50 | 0.034 | 0.032 | 0.040 | 1.000 | 0.000 |
| 2023 | 1,000 | brier | 50 | −0.002 | −0.003 | −0.002 | 1.000 | 0.000 |
| 2023 | 1,000 | auroc | 50 | 0.013 | 0.011 | 0.014 | 1.000 | 0.000 |

CI = confidence interval. Differences equal intercept recalibration minus refitting. Lower Brier score and higher AUROC favor intercept recalibration. Tests describe algorithmic resampling stability, not population inference.

**Figure S1. Performance tradeoffs**

**Figure S2. Capacity performance**

**Figure S3. Update information**
