# Supplementary material for: Temporal maintenance of an adolescent suicide attempt screening score in national surveys: An observational study
Source: Medicine (Baltimore). 2026 Aug 28;105(35):e50513. doi: 10.1097/MD.0000000000050513 (PMC13529099; doi:10.1097/MD.0000000000050513)
Supplement: Supplementary file 9 [file medi-105-e50513-s009.docx]

**Supplemental Digital Content 7**

**Bootstrap code**

Temporal maintenance of an adolescent suicide attempt screening score in national surveys: An observational study

First author: Xiaotian Ma

*Source file: run_sci_frozen_bootstrap.py*

This Word document contains the complete source code for the identified analysis step.

```
"""Survey-design bootstrap uncertainty for the independent SCI screening score.

The 2015 point estimates use cross-fitted source predictions. Later waves use
the frozen model fitted on all observed-outcome 2015 records. Uncertainty is
estimated with a Rao-Wu rescaled bootstrap that resamples PSUs within survey
strata. The model is not refitted inside the bootstrap because the estimand is
the performance of the already developed score in each survey wave.

This script intentionally contains no missing-outcome, subgroup, identity, or
fairness analysis from the companion psychology manuscript.
"""

from __future__ import annotations

import json
import time
from pathlib import Path

import joblib
import numpy as np
import pandas as pd

from run_sci_maintenance_learning_curves import SCI_PREDICTORS, maintenance_metrics


ROOT = Path(__file__).resolve().parents[1]
DATA_PATH = ROOT / "derived_data" / "yrbs_2015_2023_harmonized.parquet"
RESULTS = ROOT / "results" / "sci_maintenance"
MODEL_PATH = RESULTS / "frozen_source_model.joblib"
SOURCE_PREDICTIONS = RESULTS / "source_oof_predictions.parquet"
METADATA_PATH = RESULTS / "run_metadata.json"
SEED = 20260716
BOOTSTRAP_REPLICATES = 500
YEARS = [2015, 2017, 2019, 2021, 2023]


def report(message: str) -> None:
    print(f"[{time.strftime('%H:%M:%S')}] {message}", flush=True)


def rao_wu_multiplier(frame: pd.DataFrame, rng: np.random.Generator) -> np.ndarray:
    """Return Rao-Wu rescaling multipliers for one reset-index survey wave."""
    multiplier = np.zeros(len(frame), dtype=float)
    for _, indices in frame.groupby("stratum", observed=False).groups.items():
        positions = np.asarray(list(indices), dtype=int)
        psu = frame.iloc[positions]["psu"].astype(str).to_numpy()
        unique_psu = np.unique(psu)
        number = len(unique_psu)
        if number < 2:
            # An analytically restricted wave can contain a single sampled PSU
            # in a stratum. Treat it as a certainty unit rather than silently
            # combining substantively unrelated strata.
            multiplier[positions] = 1.0
            continue
        sampled = rng.choice(unique_psu, size=number - 1, replace=True)
        values, counts = np.unique(sampled, return_counts=True)
        factor = number / (number - 1)
        for value, count in zip(values, counts, strict=True):
            multiplier[positions[psu == value]] = factor * count
    return multiplier


def summarize_bootstrap(runs: pd.DataFrame, points: pd.DataFrame) -> pd.DataFrame:
    identifiers = {
        "year",
        "replicate",
        "score_origin",
        "capacity_threshold",
        "absolute_calibration_intercept_error",
        "absolute_calibration_slope_error",
        "close_calibration",
    }
    metric_columns = [
        column
        for column in runs.columns
        if column not in identifiers and pd.api.types.is_numeric_dtype(runs[column])
    ]
    rows: list[dict[str, object]] = []
    for year, group in runs.groupby("year", sort=True):
        point = points.loc[points["year"].eq(year)].iloc[0]
        for metric in metric_columns:
            values = group[metric].dropna().to_numpy(dtype=float)
            rows.append(
                {
                    "year": int(year),
                    "score_origin": point["score_origin"],
                    "metric": metric,
                    "point_estimate": float(point[metric]),
                    "bootstrap_replicates": int(len(values)),
                    "bootstrap_standard_error": float(np.std(values, ddof=1)),
                    "ci_lower": float(np.quantile(values, 0.025)),
                    "ci_upper": float(np.quantile(values, 0.975)),
                }
            )
    return pd.DataFrame(rows)


def main() -> None:
    metadata = json.loads(METADATA_PATH.read_text(encoding="utf-8"))
    threshold = float(metadata["frozen_threshold"])
    if metadata["predictors"] != SCI_PREDICTORS:
        raise RuntimeError("Bootstrap predictors do not match the frozen-score metadata.")

    data = pd.read_parquet(DATA_PATH)
    observed = data[data["attempt_observed"].eq(1)].copy()
    if observed["attempt"].isna().any():
        raise RuntimeError("Observed-outcome data contain missing attempt outcomes.")

    source = pd.read_parquet(SOURCE_PREDICTIONS)[["record_id", "frozen_score"]]
    observed = observed.merge(source, on="record_id", how="left", validate="one_to_one")
    model = joblib.load(MODEL_PATH)

    for year in YEARS:
        mask = observed["year"].eq(year)
        if year == 2015:
            if observed.loc[mask, "frozen_score"].isna().any():
                raise RuntimeError("Source cross-fitted predictions are incomplete.")
        else:
            observed.loc[mask, "frozen_score"] = model.predict_proba(
                observed.loc[mask, SCI_PREDICTORS]
            )[:, 1]
    if observed["frozen_score"].isna().any():
        raise RuntimeError("Frozen scores are incomplete after target-wave scoring.")

    rng = np.random.default_rng(SEED)
    point_rows: list[dict[str, object]] = []
    bootstrap_rows: list[dict[str, object]] = []
    design_rows: list[dict[str, object]] = []
    for year in YEARS:
        wave = observed[observed["year"].eq(year)].reset_index(drop=True)
        outcome = wave["attempt"].to_numpy(dtype=int)
        score = wave["frozen_score"].to_numpy(dtype=float)
        base_weight = wave["weight"].to_numpy(dtype=float)
        origin = "2015 cross-fitted" if year == 2015 else "2015 frozen model"
        point_rows.append(
            {
                "year": year,
                "score_origin": origin,
                **maintenance_metrics(outcome, score, base_weight, threshold),
            }
        )
        psus_per_stratum = wave.groupby("stratum", observed=False)["psu"].nunique()
        design_rows.append(
            {
                "year": year,
                "strata": int(len(psus_per_stratum)),
                "psus": int(psus_per_stratum.sum()),
                "single_psu_strata_treated_as_certainty": int((psus_per_stratum == 1).sum()),
                "minimum_psus_per_stratum": int(psus_per_stratum.min()),
                "maximum_psus_per_stratum": int(psus_per_stratum.max()),
            }
        )
        report(f"Rao-Wu bootstrap for {year}: n={len(wave):,}")
        for replicate in range(BOOTSTRAP_REPLICATES):
            replicate_weight = base_weight * rao_wu_multiplier(wave, rng)
            metrics = maintenance_metrics(outcome, score, replicate_weight, threshold)
            bootstrap_rows.append(
                {
                    "year": year,
                    "replicate": replicate,
                    "score_origin": origin,
                    **metrics,
                }
            )

    points = pd.DataFrame(point_rows)
    runs = pd.DataFrame(bootstrap_rows)
    summary = summarize_bootstrap(runs, points)
    points.to_csv(RESULTS / "frozen_point_estimates.csv", index=False)
    runs.to_csv(RESULTS / "frozen_bootstrap_runs.csv", index=False)
    summary.to_csv(RESULTS / "frozen_bootstrap_summary.csv", index=False)
    pd.DataFrame(design_rows).to_csv(
        RESULTS / "frozen_bootstrap_design_diagnostics.csv", index=False
    )
    report(
        f"Saved {len(runs):,} bootstrap evaluations and {len(summary):,} confidence intervals"
    )


if __name__ == "__main__":
    main()
```
