# Supplementary material for: Temporal maintenance of an adolescent suicide attempt screening score in national surveys: An observational study
Source: Medicine (Baltimore). 2026 Aug 28;105(35):e50513. doi: 10.1097/MD.0000000000050513 (PMC13529099; doi:10.1097/MD.0000000000050513)
Supplement: Supplementary file 10 [file medi-105-e50513-s010.docx]

**Supplemental Digital Content 19**

**Paired comparison data**

Temporal maintenance of an adolescent suicide attempt screening score in national surveys: An observational study

First author: Xiaotian Ma

*Source file: paired_nonparametric_summary.csv*

This Word document contains aggregate or metadata records only and does not include individual student records.

target_year,budget,metric,preferred_direction,strategy_a,strategy_b,repetitions,median_difference_a_minus_b,paired_bootstrap_median_ci_lower,paired_bootstrap_median_ci_upper,a_favorable_rate,tie_rate,wilcoxon_statistic,wilcoxon_two_sided_p,interpretation_scope

2017,250,absolute_calibration_intercept_error,lower,intercept,static,50,-0.15197905238267,-0.1680892488731284,-0.1069530617691242,0.86,0.0,50.0,5.610267805877811e-11,"algorithmic resampling stability, not population inference"

2017,250,absolute_calibration_intercept_error,lower,logistic,static,50,-0.14882438898215308,-0.16654628724317921,-0.10137504559453155,0.88,0.0,41.0,1.7687185049908294e-11,"algorithmic resampling stability, not population inference"

2017,250,absolute_calibration_intercept_error,lower,refit,static,50,-0.14801624400545016,-0.1682671868905254,-0.1279572750857091,0.96,0.0,28.0,2.6343371928305714e-12,"algorithmic resampling stability, not population inference"

2017,250,absolute_calibration_intercept_error,lower,intercept,logistic,50,0.0008070551847964014,-0.0027297618457871042,0.007171609660644437,0.48,0.0,591.0,0.6596977023333945,"algorithmic resampling stability, not population inference"

2017,250,absolute_calibration_intercept_error,lower,intercept,refit,50,0.020743313731959014,-0.0183251688354249,0.034185417661711195,0.46,0.0,581.0,0.5918179227766114,"algorithmic resampling stability, not population inference"

2017,250,absolute_calibration_intercept_error,lower,logistic,refit,50,0.008628434044244362,-0.017869473391376012,0.022985609956477693,0.44,0.0,572.0,0.5335711137818162,"algorithmic resampling stability, not population inference"

2017,250,absolute_calibration_slope_error,lower,intercept,static,50,0.0406593682116079,0.0299533793453469,0.0555620788426526,0.0,0.0,0.0,1.7763568394002505e-15,"algorithmic resampling stability, not population inference"

2017,250,absolute_calibration_slope_error,lower,logistic,static,50,0.11008602942508225,0.0771109571400005,0.14477856818816498,0.0,0.0,0.0,1.7763568394002505e-15,"algorithmic resampling stability, not population inference"

2017,250,absolute_calibration_slope_error,lower,refit,static,50,0.32715569497282615,0.2931645186786188,0.36436770585347644,0.0,0.0,0.0,1.7763568394002505e-15,"algorithmic resampling stability, not population inference"

2017,250,absolute_calibration_slope_error,lower,intercept,logistic,50,-0.07912699001757964,-0.10075937013723316,-0.04675788543658785,0.82,0.0,113.0,2.6378053519238165e-08,"algorithmic resampling stability, not population inference"

2017,250,absolute_calibration_slope_error,lower,intercept,refit,50,-0.2735908568291846,-0.3267379692135184,-0.240737808383539,0.98,0.0,9.0,5.861977570020827e-14,"algorithmic resampling stability, not population inference"

2017,250,absolute_calibration_slope_error,lower,logistic,refit,50,-0.2190180817080459,-0.2571951926876821,-0.17480407205429044,0.84,0.0,65.0,3.134559278805682e-10,"algorithmic resampling stability, not population inference"

2017,250,brier,lower,intercept,static,50,0.00030245702197520194,4.132556179680183e-05,0.000519805818323199,0.34,0.0,270.0,0.000251529064962952,"algorithmic resampling stability, not population inference"

2017,250,brier,lower,logistic,static,50,0.0009062971999088017,0.000620811295968051,0.0014819180328080536,0.1,0.0,33.0,5.707434524993005e-12,"algorithmic resampling stability, not population inference"

2017,250,brier,lower,refit,static,50,0.004693691833851751,0.004280526293073201,0.0051839308782605995,0.0,0.0,0.0,1.7763568394002505e-15,"algorithmic resampling stability, not population inference"

2017,250,brier,lower,intercept,logistic,50,-0.0005790829360558976,-0.0011145578008524004,-0.000408491021724397,0.9,0.0,60.0,1.8094681308866711e-10,"algorithmic resampling stability, not population inference"

2017,250,brier,lower,intercept,refit,50,-0.004522455873466152,-0.004706142947182298,-0.004094711948790596,1.0,0.0,0.0,1.7763568394002505e-15,"algorithmic resampling stability, not population inference"

2017,250,brier,lower,logistic,refit,50,-0.003841576749411199,-0.004224332035198203,-0.003299418200354301,0.98,0.0,2.0,5.329070518200751e-15,"algorithmic resampling stability, not population inference"

2017,250,auroc,higher,intercept,static,50,-0.004867304508847536,-0.005981144894345092,-0.0035135461304979243,0.04,0.0,5.0,1.7763568394002505e-14,"algorithmic resampling stability, not population inference"

2017,250,auroc,higher,logistic,static,50,-0.010456067600086383,-0.012531696879221788,-0.00783673966736187,0.0,0.0,0.0,1.7763568394002505e-15,"algorithmic resampling stability, not population inference"

2017,250,auroc,higher,refit,static,50,-0.04019568979111393,-0.04621674585392255,-0.03393677975195031,0.0,0.0,0.0,1.7763568394002505e-15,"algorithmic resampling stability, not population inference"

2017,250,auroc,higher,intercept,logistic,50,0.004318858075279208,0.00278377678856756,0.006159123172976955,0.92,0.0,27.0,2.239985974483716e-12,"algorithmic resampling stability, not population inference"

2017,250,auroc,higher,intercept,refit,50,0.03401177892990093,0.030208542544765182,0.04030855705433112,1.0,0.0,0.0,1.7763568394002505e-15,"algorithmic resampling stability, not population inference"

2017,250,auroc,higher,logistic,refit,50,0.03014651848826161,0.02432458584042918,0.03380821027894859,1.0,0.0,0.0,1.7763568394002505e-15,"algorithmic resampling stability, not population inference"

2017,500,absolute_calibration_intercept_error,lower,intercept,static,50,-0.1869407162301432,-0.20286817015286995,-0.1677366993344416,1.0,0.0,0.0,1.7763568394002505e-15,"algorithmic resampling stability, not population inference"

2017,500,absolute_calibration_intercept_error,lower,logistic,static,50,-0.1792152279828808,-0.21119096235058785,-0.1663117544658119,1.0,0.0,0.0,1.7763568394002505e-15,"algorithmic resampling stability, not population inference"

2017,500,absolute_calibration_intercept_error,lower,refit,static,50,-0.1886636940018239,-0.2132698099048933,-0.16987851058913445,1.0,0.0,0.0,1.7763568394002505e-15,"algorithmic resampling stability, not population inference"

2017,500,absolute_calibration_intercept_error,lower,intercept,logistic,50,-0.0017863321263454496,-0.006065036617404346,0.0012362586650361998,0.58,0.0,503.0,0.1976349682926415,"algorithmic resampling stability, not population inference"

2017,500,absolute_calibration_intercept_error,lower,intercept,refit,50,0.004481952756466097,-0.0031607738303131537,0.0105787521050046,0.4,0.0,505.0,0.20447619481721802,"algorithmic resampling stability, not population inference"

2017,500,absolute_calibration_intercept_error,lower,logistic,refit,50,0.0072994697518333485,-0.0061320208590821025,0.017246261746695996,0.38,0.0,470.0,0.10737600575610173,"algorithmic resampling stability, not population inference"

2017,500,absolute_calibration_slope_error,lower,intercept,static,50,0.0187468042959126,0.0125018443434,0.02431034121361675,0.0,0.0,0.0,1.7763568394002505e-15,"algorithmic resampling stability, not population inference"

2017,500,absolute_calibration_slope_error,lower,logistic,static,50,0.05853277763562575,0.0312242838490105,0.0863125671079107,0.0,0.0,0.0,1.7763568394002505e-15,"algorithmic resampling stability, not population inference"

2017,500,absolute_calibration_slope_error,lower,refit,static,50,0.20151511173575676,0.1829739976534158,0.23566708126002167,0.0,0.0,0.0,1.7763568394002505e-15,"algorithmic resampling stability, not population inference"

2017,500,absolute_calibration_slope_error,lower,intercept,logistic,50,-0.03140814355351955,-0.049378111338374095,-0.0162376484307133,0.78,0.0,116.0,3.348859145546612e-08,"algorithmic resampling stability, not population inference"

2017,500,absolute_calibration_slope_error,lower,intercept,refit,50,-0.19131589181948516,-0.2189696764311048,-0.1559334542410123,1.0,0.0,0.0,1.7763568394002505e-15,"algorithmic resampling stability, not population inference"

2017,500,absolute_calibration_slope_error,lower,logistic,refit,50,-0.13799547336168094,-0.18057742662974213,-0.11304280823172114,0.9,0.0,53.0,8.060396794462577e-11,"algorithmic resampling stability, not population inference"

2017,500,brier,lower,intercept,static,50,-0.00018812395933644893,-0.00031299276124159905,-8.676777744259789e-05,0.7,0.0,346.0,0.004316904154682888,"algorithmic resampling stability, not population inference"

2017,500,brier,lower,logistic,static,50,0.00020860587765460237,7.495177847330337e-05,0.00040129718142049853,0.28,0.0,303.0,0.0009504084140701252,"algorithmic resampling stability, not population inference"

2017,500,brier,lower,refit,static,50,0.002353176335909604,0.0020554815471139527,0.0027170228574487504,0.0,0.0,0.0,1.7763568394002505e-15,"algorithmic resampling stability, not population inference"

2017,500,brier,lower,intercept,logistic,50,-0.00032813001588364796,-0.0005887808082580062,-0.00023278190669650395,0.82,0.0,82.0,1.7568968502246207e-09,"algorithmic resampling stability, not population inference"

2017,500,brier,lower,intercept,refit,50,-0.0025399146395710982,-0.002907279064283501,-0.0023543305727087988,1.0,0.0,0.0,1.7763568394002505e-15,"algorithmic resampling stability, not population inference"

2017,500,brier,lower,logistic,refit,50,-0.002075712112491652,-0.0023621539554895023,-0.001846065608062348,1.0,0.0,0.0,1.7763568394002505e-15,"algorithmic resampling stability, not population inference"

2017,500,auroc,higher,intercept,static,50,-0.001860905040064309,-0.003108693728499645,-0.0012785238494630313,0.12,0.0,43.0,2.3080204414327454e-11,"algorithmic resampling stability, not population inference"

2017,500,auroc,higher,logistic,static,50,-0.0046462817638667775,-0.00555288711240165,-0.003860310401229766,0.04,0.0,5.0,1.7763568394002505e-14,"algorithmic resampling stability, not population inference"

2017,500,auroc,higher,refit,static,50,-0.019763990290142863,-0.023435329286720208,-0.018021871084248964,0.0,0.0,0.0,1.7763568394002505e-15,"algorithmic resampling stability, not population inference"

2017,500,auroc,higher,intercept,logistic,50,0.001847417550673136,0.0012315095006396604,0.0023942675117055168,0.88,0.0,71.0,5.897913268881894e-10,"algorithmic resampling stability, not population inference"

2017,500,auroc,higher,intercept,refit,50,0.01756668622799401,0.01538283469605839,0.020820315501611675,1.0,0.0,0.0,1.7763568394002505e-15,"algorithmic resampling stability, not population inference"

2017,500,auroc,higher,logistic,refit,50,0.015970593401938515,0.013884142194128146,0.01714685788975523,1.0,0.0,0.0,1.7763568394002505e-15,"algorithmic resampling stability, not population inference"

2017,1000,absolute_calibration_intercept_error,lower,intercept,static,50,-0.22571026550214945,-0.2379707808335518,-0.20079363972380798,1.0,0.0,0.0,1.7763568394002505e-15,"algorithmic resampling stability, not population inference"

2017,1000,absolute_calibration_intercept_error,lower,logistic,static,50,-0.22469469246863275,-0.23790517164909308,-0.2053292974550355,1.0,0.0,0.0,1.7763568394002505e-15,"algorithmic resampling stability, not population inference"

2017,1000,absolute_calibration_intercept_error,lower,refit,static,50,-0.22281849443995144,-0.2438203305893585,-0.2066947571666709,1.0,0.0,0.0,1.7763568394002505e-15,"algorithmic resampling stability, not population inference"

2017,1000,absolute_calibration_intercept_error,lower,intercept,logistic,50,0.00011667596096995,-0.0015983680920487985,0.000808866661225642,0.48,0.0,600.0,0.7232586770768918,"algorithmic resampling stability, not population inference"

2017,1000,absolute_calibration_intercept_error,lower,intercept,refit,50,-7.301273326039976e-05,-0.007542834480736202,0.009439334808204003,0.5,0.0,624.0,0.9010179923007229,"algorithmic resampling stability, not population inference"

2017,1000,absolute_calibration_intercept_error,lower,logistic,refit,50,0.0013323053784094498,-0.0118461180001126,0.009634666207618646,0.48,0.0,613.0,0.81837027658068,"algorithmic resampling stability, not population inference"

2017,1000,absolute_calibration_slope_error,lower,intercept,static,50,0.007811066394583,0.0065538854926506995,0.012151519945528851,0.08,0.0,22.0,9.521272659185342e-13,"algorithmic resampling stability, not population inference"

2017,1000,absolute_calibration_slope_error,lower,logistic,static,50,0.028577740721868,0.014322860014427001,0.0396028356517456,0.12,0.0,46.0,3.40367733997482e-11,"algorithmic resampling stability, not population inference"

2017,1000,absolute_calibration_slope_error,lower,refit,static,50,0.11296194877972646,0.1005767795254812,0.1227960381234814,0.0,0.0,0.0,1.7763568394002505e-15,"algorithmic resampling stability, not population inference"

2017,1000,absolute_calibration_slope_error,lower,intercept,logistic,50,-0.0215798279620367,-0.0317805852953052,-0.0039780911309324,0.7,0.0,247.0,9.040639391599825e-05,"algorithmic resampling stability, not population inference"

2017,1000,absolute_calibration_slope_error,lower,intercept,refit,50,-0.10548176765540315,-0.1133328295271073,-0.0874883304119967,0.98,0.0,1.0,3.552713678800501e-15,"algorithmic resampling stability, not population inference"

2017,1000,absolute_calibration_slope_error,lower,logistic,refit,50,-0.08801236427482545,-0.100170744069513,-0.07596428031265451,0.9,0.0,49.0,4.9604764740251994e-11,"algorithmic resampling stability, not population inference"

2017,1000,brier,lower,intercept,static,50,-0.0003862890607227998,-0.00045087986719220124,-0.00030026277390934886,0.86,0.0,58.0,1.4432899320127035e-10,"algorithmic resampling stability, not population inference"

2017,1000,brier,lower,logistic,static,50,-0.00014275995734599686,-0.00024759208769310065,-5.989496536129915e-05,0.72,0.0,383.0,0.01330480151673541,"algorithmic resampling stability, not population inference"

2017,1000,brier,lower,refit,static,50,0.0014621997479678031,0.00129524602914885,0.0015682190604181523,0.0,0.0,0.0,1.7763568394002505e-15,"algorithmic resampling stability, not population inference"

2017,1000,brier,lower,intercept,logistic,50,-0.00018896440832480432,-0.0002875860985851464,-0.00011998403422810305,0.82,0.0,142.0,2.305757291765076e-07,"algorithmic resampling stability, not population inference"

2017,1000,brier,lower,intercept,refit,50,-0.001772658355865396,-0.0018378797114266023,-0.0015614605965038053,1.0,0.0,0.0,1.7763568394002505e-15,"algorithmic resampling stability, not population inference"

2017,1000,brier,lower,logistic,refit,50,-0.0015623902110926506,-0.0017341455962858049,-0.0013752468889932992,1.0,0.0,0.0,1.7763568394002505e-15,"algorithmic resampling stability, not population inference"

2017,1000,auroc,higher,intercept,static,50,-0.0007216462776882815,-0.00142853012658406,-0.0003839860895014091,0.26,0.0,178.0,2.36936675790389e-06,"algorithmic resampling stability, not population inference"

2017,1000,auroc,higher,logistic,static,50,-0.0026414762832317473,-0.003012308505748018,-0.002095218247433972,0.12,0.0,34.0,6.616929226765933e-12,"algorithmic resampling stability, not population inference"

2017,1000,auroc,higher,refit,static,50,-0.012985873742911769,-0.014766413469470496,-0.01182174800702751,0.0,0.0,0.0,1.7763568394002505e-15,"algorithmic resampling stability, not population inference"

2017,1000,auroc,higher,intercept,logistic,50,0.0010103931530904275,0.0007544046611859256,0.0016855369596331382,0.92,0.0,38.0,1.1727507853720454e-11,"algorithmic resampling stability, not population inference"

2017,1000,auroc,higher,intercept,refit,50,0.011703922059632721,0.010720984141504408,0.013026183578735306,1.0,0.0,0.0,1.7763568394002505e-15,"algorithmic resampling stability, not population inference"

2017,1000,auroc,higher,logistic,refit,50,0.010324265212182382,0.008885241734729987,0.01162536446726048,1.0,0.0,0.0,1.7763568394002505e-15,"algorithmic resampling stability, not population inference"

2017,2000,absolute_calibration_intercept_error,lower,intercept,static,50,-0.23032044697114507,-0.2375755873011154,-0.2221913532789315,1.0,0.0,0.0,1.7763568394002505e-15,"algorithmic resampling stability, not population inference"

2017,2000,absolute_calibration_intercept_error,lower,logistic,static,50,-0.23123633552175765,-0.23893583591692208,-0.2210389645907782,1.0,0.0,0.0,1.7763568394002505e-15,"algorithmic resampling stability, not population inference"

2017,2000,absolute_calibration_intercept_error,lower,refit,static,50,-0.23499935319326865,-0.2440275598791382,-0.22172835427281823,1.0,0.0,0.0,1.7763568394002505e-15,"algorithmic resampling stability, not population inference"

2017,2000,absolute_calibration_intercept_error,lower,intercept,logistic,50,-0.00020323285449000417,-0.0009163233149636482,0.0005645078605094542,0.54,0.0,637.0,1.0,"algorithmic resampling stability, not population inference"

2017,2000,absolute_calibration_intercept_error,lower,intercept,refit,50,0.0026298033560306494,-0.00027367936254624973,0.007150695511625301,0.42,0.0,431.0,0.04610038786489312,"algorithmic resampling stability, not population inference"

2017,2000,absolute_calibration_intercept_error,lower,logistic,refit,50,0.0023007545895017997,-0.0006318377619992988,0.006895310180012399,0.4,0.0,437.0,0.052989788310034314,"algorithmic resampling stability, not population inference"

2017,2000,absolute_calibration_slope_error,lower,intercept,static,50,0.00667639680788915,0.00529074595131245,0.008592969325499,0.1,0.0,24.0,1.3535839116229909e-12,"algorithmic resampling stability, not population inference"

2017,2000,absolute_calibration_slope_error,lower,logistic,static,50,0.028873859926623946,0.016860337596187,0.036819475467743945,0.06,0.0,9.0,5.861977570020827e-14,"algorithmic resampling stability, not population inference"

2017,2000,absolute_calibration_slope_error,lower,refit,static,50,0.037880289496979094,0.02996410016606081,0.0559282891969096,0.0,0.0,0.0,1.7763568394002505e-15,"algorithmic resampling stability, not population inference"

2017,2000,absolute_calibration_slope_error,lower,intercept,logistic,50,-0.0208488363641155,-0.0330113867786279,-0.0102453888998937,0.84,0.0,98.0,7.546860913976161e-09,"algorithmic resampling stability, not population inference"

2017,2000,absolute_calibration_slope_error,lower,intercept,refit,50,-0.03186396813053265,-0.050868806757126704,-0.023686177876043553,0.92,0.0,27.0,2.239985974483716e-12,"algorithmic resampling stability, not population inference"

2017,2000,absolute_calibration_slope_error,lower,logistic,refit,50,-0.01951485784108455,-0.03833353997374725,-0.001108534666611198,0.66,0.0,375.0,0.010566648384996213,"algorithmic resampling stability, not population inference"

2017,2000,brier,lower,intercept,static,50,-0.0004451989509610989,-0.0005115654732308003,-0.0004025862121652471,0.98,0.0,3.0,8.881784197001252e-15,"algorithmic resampling stability, not population inference"

2017,2000,brier,lower,logistic,static,50,-0.00034356978600319846,-0.0004125512487653492,-0.00024076458174610016,0.8,0.0,170.0,1.4543591948523726e-06,"algorithmic resampling stability, not population inference"

2017,2000,brier,lower,refit,static,50,0.0004382246805152007,0.00034622622249540214,0.0006529115641464509,0.02,0.0,5.0,1.7763568394002505e-14,"algorithmic resampling stability, not population inference"

2017,2000,brier,lower,intercept,logistic,50,-0.00011927179288214948,-0.0001635267662144016,-8.827144381440277e-05,0.86,0.0,73.0,7.237197507947712e-10,"algorithmic resampling stability, not population inference"

2017,2000,brier,lower,intercept,refit,50,-0.0009324237635336477,-0.0010572989241821491,-0.0008333065833438028,1.0,0.0,0.0,1.7763568394002505e-15,"algorithmic resampling stability, not population inference"

2017,2000,brier,lower,logistic,refit,50,-0.0008045876268785029,-0.0009196886826966996,-0.0006979558819123974,1.0,0.0,0.0,1.7763568394002505e-15,"algorithmic resampling stability, not population inference"

2017,2000,auroc,higher,intercept,static,50,-0.0005308599121797153,-0.0006672714578498939,-0.00040587679663256493,0.22,0.0,168.0,1.2841054282120012e-06,"algorithmic resampling stability, not population inference"

2017,2000,auroc,higher,logistic,static,50,-0.0012329584614507727,-0.001716250530712879,-0.0008672853296698424,0.1,0.0,25.0,1.6058265828178264e-12,"algorithmic resampling stability, not population inference"

2017,2000,auroc,higher,refit,static,50,-0.005806916249793015,-0.006455022302513602,-0.004679837709717916,0.04,0.0,8.0,4.440892098500626e-14,"algorithmic resampling stability, not population inference"

2017,2000,auroc,higher,intercept,logistic,50,0.000660024400727488,0.0004307837097921041,0.0009352834200495191,0.82,0.0,135.0,1.4032918471684752e-07,"algorithmic resampling stability, not population inference"

2017,2000,auroc,higher,intercept,refit,50,0.0050377345871288615,0.003989072390526149,0.005788428429198855,0.94,0.0,14.0,1.9539925233402755e-13,"algorithmic resampling stability, not population inference"

2017,2000,auroc,higher,logistic,refit,50,0.004343666629522958,0.003132605366973795,0.005150531379948586,0.92,0.0,22.0,9.521272659185342e-13,"algorithmic resampling stability, not population inference"

2017,4000,absolute_calibration_intercept_error,lower,intercept,static,50,-0.24627633945664362,-0.2516672738032989,-0.24113623301320025,1.0,0.0,0.0,1.7763568394002505e-15,"algorithmic resampling stability, not population inference"

2017,4000,absolute_calibration_intercept_error,lower,logistic,static,50,-0.24504900054876294,-0.2526032104455253,-0.241741623148704,1.0,0.0,0.0,1.7763568394002505e-15,"algorithmic resampling stability, not population inference"

2017,4000,absolute_calibration_intercept_error,lower,refit,static,50,-0.24793604456082968,-0.2516872420533917,-0.2433688962445835,1.0,0.0,0.0,1.7763568394002505e-15,"algorithmic resampling stability, not population inference"

2017,4000,absolute_calibration_intercept_error,lower,intercept,logistic,50,0.00044331338849250106,-0.0006947136897124014,0.0012479724176846507,0.46,0.0,520.0,0.26120880915335754,"algorithmic resampling stability, not population inference"

2017,4000,absolute_calibration_intercept_error,lower,intercept,refit,50,-0.00012474479013555018,-0.0035667539184582,0.002466122945717051,0.5,0.0,616.0,0.8407540567937097,"algorithmic resampling stability, not population inference"

2017,4000,absolute_calibration_intercept_error,lower,logistic,refit,50,0.0004745602587095995,-0.0034491466996416998,0.0030626685151073506,0.48,0.0,597.0,0.7018365959451138,"algorithmic resampling stability, not population inference"

2017,4000,absolute_calibration_slope_error,lower,intercept,static,50,0.0034527906746344506,0.0017938460695484,0.004605713528987451,0.16,0.0,78.0,1.192391962945294e-09,"algorithmic resampling stability, not population inference"

2017,4000,absolute_calibration_slope_error,lower,logistic,static,50,0.008812318782552751,0.0043182688655936,0.014258468704818202,0.18,0.0,77.0,1.080529443697742e-09,"algorithmic resampling stability, not population inference"

2017,4000,absolute_calibration_slope_error,lower,refit,static,50,0.0185274398249585,0.014989793769298501,0.023242971337933,0.12,0.0,36.0,8.842704346534447e-12,"algorithmic resampling stability, not population inference"

2017,4000,absolute_calibration_slope_error,lower,intercept,logistic,50,-0.004288438841439049,-0.0109383658190454,-0.00128154871570035,0.7,0.0,246.0,8.630484972549368e-05,"algorithmic resampling stability, not population inference"

2017,4000,absolute_calibration_slope_error,lower,intercept,refit,50,-0.0149596103848968,-0.0204261895454943,-0.0103910197455124,0.8,0.0,103.0,1.158324636207908e-08,"algorithmic resampling stability, not population inference"

2017,4000,absolute_calibration_slope_error,lower,logistic,refit,50,-0.010917932724660252,-0.01811085228150265,-0.0012169868084587997,0.68,0.0,330.0,0.002525686043762576,"algorithmic resampling stability, not population inference"

2017,4000,brier,lower,intercept,static,50,-0.0005228004690644474,-0.0005467379150378496,-0.00048442850459389647,1.0,0.0,0.0,1.7763568394002505e-15,"algorithmic resampling stability, not population inference"

2017,4000,brier,lower,logistic,static,50,-0.000456751916022749,-0.0004916329371054962,-0.0004131791227224013,1.0,0.0,0.0,1.7763568394002505e-15,"algorithmic resampling stability, not population inference"

2017,4000,brier,lower,refit,static,50,-2.189685745598857e-06,-0.00010894083615024808,9.748786913810203e-05,0.5,0.0,620.0,0.8707939059443142,"algorithmic resampling stability, not population inference"

2017,4000,brier,lower,intercept,logistic,50,-5.963696920134945e-05,-8.777718918894992e-05,-3.364835990379844e-05,0.86,0.0,73.0,7.237197507947712e-10,"algorithmic resampling stability, not population inference"

2017,4000,brier,lower,intercept,refit,50,-0.0004990957615025014,-0.0005932808257667968,-0.00043606940166939986,0.94,0.0,10.0,7.638334409421077e-14,"algorithmic resampling stability, not population inference"

2017,4000,brier,lower,logistic,refit,50,-0.0004435735450407999,-0.0005169307853845964,-0.0003677237686839016,0.94,0.0,33.0,5.707434524993005e-12,"algorithmic resampling stability, not population inference"

2017,4000,auroc,higher,intercept,static,50,-0.0001988479744013949,-0.00039570467567884293,-3.464867443281339e-05,0.34,0.0,388.0,0.01531204751384152,"algorithmic resampling stability, not population inference"

2017,4000,auroc,higher,logistic,static,50,-0.0005340499302437518,-0.0006982763014970472,-0.0003361743895248237,0.18,0.0,97.0,6.917133532624575e-09,"algorithmic resampling stability, not population inference"

2017,4000,auroc,higher,refit,static,50,-0.0007277063628443603,-0.0011720401642960065,0.0003837857397538391,0.42,0.0,483.0,0.13807290852462017,"algorithmic resampling stability, not population inference"

2017,4000,auroc,higher,intercept,logistic,50,0.00045045400815207914,0.00010242429449158763,0.0006159726521292597,0.66,0.0,241.0,6.825250486741652e-05,"algorithmic resampling stability, not population inference"

2017,4000,auroc,higher,intercept,refit,50,0.0005580276858787037,-0.0003574794520956326,0.0009140162305035782,0.56,0.0,544.0,0.3724861917482851,"algorithmic resampling stability, not population inference"

2017,4000,auroc,higher,logistic,refit,50,-0.0001338509091536877,-0.0007556170402884255,0.00043939119840108454,0.48,0.0,571.0,0.5272794190915597,"algorithmic resampling stability, not population inference"

2019,250,absolute_calibration_intercept_error,lower,intercept,static,50,-0.0310980498999395,-0.06193542722921645,0.004720641415779797,0.62,0.0,503.0,0.1976349682926415,"algorithmic resampling stability, not population inference"

2019,250,absolute_calibration_intercept_error,lower,logistic,static,50,-0.0230705193310217,-0.050977092547485406,0.008855785600406944,0.6,0.0,570.0,0.5210251168620434,"algorithmic resampling stability, not population inference"

2019,250,absolute_calibration_intercept_error,lower,refit,static,50,-0.03019134946798291,-0.055911056947289504,0.009873380082774746,0.62,0.0,529.0,0.2999546071877397,"algorithmic resampling stability, not population inference"

2019,250,absolute_calibration_intercept_error,lower,intercept,logistic,50,0.00016460330832484757,-0.0077048499030432545,0.003118961172885744,0.48,0.0,476.0,0.12080975020940343,"algorithmic resampling stability, not population inference"

2019,250,absolute_calibration_intercept_error,lower,intercept,refit,50,0.0025311489043543,-0.023638553276179603,0.027480864233391383,0.5,0.0,628.0,0.9313837099567692,"algorithmic resampling stability, not population inference"

2019,250,absolute_calibration_intercept_error,lower,logistic,refit,50,0.0030584904958125686,-0.0240511406940927,0.03132617662810909,0.48,0.0,583.0,0.6051405400941228,"algorithmic resampling stability, not population inference"

2019,250,absolute_calibration_slope_error,lower,intercept,static,50,0.03508267366228665,0.026543031933119448,0.0443964723696575,0.04,0.0,8.0,4.440892098500626e-14,"algorithmic resampling stability, not population inference"

2019,250,absolute_calibration_slope_error,lower,logistic,static,50,0.08530263123041382,0.07057215979723774,0.1149233664612783,0.04,0.0,4.0,1.2434497875801753e-14,"algorithmic resampling stability, not population inference"

2019,250,absolute_calibration_slope_error,lower,refit,static,50,0.3514605493681552,0.2739271691033399,0.37488694208393625,0.0,0.0,0.0,1.7763568394002505e-15,"algorithmic resampling stability, not population inference"

2019,250,absolute_calibration_slope_error,lower,intercept,logistic,50,-0.0426776326626798,-0.0693649105693324,-0.0144860398409173,0.74,0.0,200.0,8.398093875428003e-06,"algorithmic resampling stability, not population inference"

2019,250,absolute_calibration_slope_error,lower,intercept,refit,50,-0.30028704711750975,-0.3436532476947236,-0.246027168130308,0.96,0.0,13.0,1.5631940186722204e-13,"algorithmic resampling stability, not population inference"

2019,250,absolute_calibration_slope_error,lower,logistic,refit,50,-0.24075113809866233,-0.3044721599109141,-0.16989749434376258,0.92,0.0,37.0,1.0192735544478637e-11,"algorithmic resampling stability, not population inference"

2019,250,brier,lower,intercept,static,50,0.0006911943332916087,0.0005990180680380667,0.0009157415118132561,0.1,0.0,34.0,6.616929226765933e-12,"algorithmic resampling stability, not population inference"

2019,250,brier,lower,logistic,static,50,0.0015612141858512077,0.001173976004599911,0.001927394692881751,0.04,0.0,7.0,3.375077994860476e-14,"algorithmic resampling stability, not population inference"

2019,250,brier,lower,refit,static,50,0.005185529583031208,0.004808129831755652,0.005597059856874004,0.0,0.0,0.0,1.7763568394002505e-15,"algorithmic resampling stability, not population inference"

2019,250,brier,lower,intercept,logistic,50,-0.0006577875478662037,-0.000875757202339747,-0.00035970115044319656,0.88,0.0,76.0,9.785132704109856e-10,"algorithmic resampling stability, not population inference"

2019,250,brier,lower,intercept,refit,50,-0.004390025799488752,-0.005032082468219595,-0.004033051566508697,1.0,0.0,0.0,1.7763568394002505e-15,"algorithmic resampling stability, not population inference"

2019,250,brier,lower,logistic,refit,50,-0.0039882438420899,-0.004304607846841849,-0.0036012382836941997,1.0,0.0,0.0,1.7763568394002505e-15,"algorithmic resampling stability, not population inference"

2019,250,auroc,higher,intercept,static,50,-0.004259802527047107,-0.00598800126560306,-0.00298257315404421,0.04,0.0,13.0,1.5631940186722204e-13,"algorithmic resampling stability, not population inference"

2019,250,auroc,higher,logistic,static,50,-0.008500195832941038,-0.01046633974114719,-0.0068764055060796525,0.0,0.0,0.0,1.7763568394002505e-15,"algorithmic resampling stability, not population inference"

2019,250,auroc,higher,refit,static,50,-0.043192270924622356,-0.046418810102547614,-0.03639818839064285,0.0,0.0,0.0,1.7763568394002505e-15,"algorithmic resampling stability, not population inference"

2019,250,auroc,higher,intercept,logistic,50,0.003360235996904548,0.0024199660061856587,0.004446428626238452,0.9,0.0,81.0,1.5961560961841315e-09,"algorithmic resampling stability, not population inference"

2019,250,auroc,higher,intercept,refit,50,0.03760853541171866,0.03249802666973611,0.040984158589960185,1.0,0.0,0.0,1.7763568394002505e-15,"algorithmic resampling stability, not population inference"

2019,250,auroc,higher,logistic,refit,50,0.0341908888973399,0.028934497589590602,0.03785202746336391,1.0,0.0,0.0,1.7763568394002505e-15,"algorithmic resampling stability, not population inference"

2019,500,absolute_calibration_intercept_error,lower,intercept,static,50,-0.043963836864101706,-0.06760628192671285,-0.022426794980687705,0.76,0.0,315.0,0.0014846473444478647,"algorithmic resampling stability, not population inference"

2019,500,absolute_calibration_intercept_error,lower,logistic,static,50,-0.0438122432925236,-0.06606086553484015,-0.027761392070987205,0.72,0.0,321.0,0.0018425334424101436,"algorithmic resampling stability, not population inference"

2019,500,absolute_calibration_intercept_error,lower,refit,static,50,-0.03336356768596535,-0.0596574818431783,-0.018301684337251503,0.76,0.0,290.0,0.0005734773907004609,"algorithmic resampling stability, not population inference"

2019,500,absolute_calibration_intercept_error,lower,intercept,logistic,50,0.0006944638551117984,-0.002851474139078297,0.0023511698161974987,0.48,0.0,599.0,0.7160933233749613,"algorithmic resampling stability, not population inference"

2019,500,absolute_calibration_intercept_error,lower,intercept,refit,50,0.002391674870451048,-0.012649541307126002,0.012281770338091497,0.5,0.0,629.0,0.9389922173111831,"algorithmic resampling stability, not population inference"

2019,500,absolute_calibration_intercept_error,lower,logistic,refit,50,0.008948276937961849,-0.0168559103909853,0.013699264372417298,0.44,0.0,637.0,1.0,"algorithmic resampling stability, not population inference"

2019,500,absolute_calibration_slope_error,lower,intercept,static,50,0.0222828898372827,0.0174765109619568,0.026880454877421696,0.02,0.0,3.0,8.881784197001252e-15,"algorithmic resampling stability, not population inference"

2019,500,absolute_calibration_slope_error,lower,logistic,static,50,0.0551716671669545,0.04215962501468705,0.07271622694489596,0.06,0.0,15.0,2.433608869978343e-13,"algorithmic resampling stability, not population inference"

2019,500,absolute_calibration_slope_error,lower,refit,static,50,0.19535538847375633,0.17122231174182048,0.20835679808142823,0.0,0.0,0.0,1.7763568394002505e-15,"algorithmic resampling stability, not population inference"

2019,500,absolute_calibration_slope_error,lower,intercept,logistic,50,-0.0354521910262932,-0.049454738181701395,-0.01704891750535375,0.78,0.0,155.0,5.572207957271758e-07,"algorithmic resampling stability, not population inference"

2019,500,absolute_calibration_slope_error,lower,intercept,refit,50,-0.16910383084923852,-0.19090533958149808,-0.1405429011429735,1.0,0.0,0.0,1.7763568394002505e-15,"algorithmic resampling stability, not population inference"

2019,500,absolute_calibration_slope_error,lower,logistic,refit,50,-0.1386217330532269,-0.1719443599803167,-0.1115410089025871,0.88,0.0,72.0,6.535714192068554e-10,"algorithmic resampling stability, not population inference"

2019,500,brier,lower,intercept,static,50,0.0003272351272853094,0.00015558239166010845,0.00048765792589565427,0.14,0.0,95.0,5.802245794939154e-09,"algorithmic resampling stability, not population inference"

2019,500,brier,lower,logistic,static,50,0.0007275764062829038,0.0006048177633071056,0.0009583829091596024,0.06,0.0,13.0,1.5631940186722204e-13,"algorithmic resampling stability, not population inference"

2019,500,brier,lower,refit,static,50,0.003432550454201551,0.003199197615216802,0.0036675112009613986,0.0,0.0,0.0,1.7763568394002505e-15,"algorithmic resampling stability, not population inference"

2019,500,brier,lower,intercept,logistic,50,-0.00030101661280070025,-0.0005984495469986972,-0.00022225522673580134,0.9,0.0,43.0,2.3080204414327454e-11,"algorithmic resampling stability, not population inference"

2019,500,brier,lower,intercept,refit,50,-0.003195113375453848,-0.003342914327109099,-0.0028270176610960956,1.0,0.0,0.0,1.7763568394002505e-15,"algorithmic resampling stability, not population inference"

2019,500,brier,lower,logistic,refit,50,-0.0027320901224348465,-0.002991205127168245,-0.002342210985760103,1.0,0.0,0.0,1.7763568394002505e-15,"algorithmic resampling stability, not population inference"

2019,500,auroc,higher,intercept,static,50,-0.003231608529211172,-0.00418678426313962,-0.0024160331270515756,0.04,0.0,5.0,1.7763568394002505e-14,"algorithmic resampling stability, not population inference"

2019,500,auroc,higher,logistic,static,50,-0.005861046780359924,-0.007152663835860218,-0.004337951931995099,0.02,0.0,1.0,3.552713678800501e-15,"algorithmic resampling stability, not population inference"

2019,500,auroc,higher,refit,static,50,-0.024788686503580293,-0.025821613015450773,-0.02277717430547399,0.0,0.0,0.0,1.7763568394002505e-15,"algorithmic resampling stability, not population inference"

2019,500,auroc,higher,intercept,logistic,50,0.0016705576926537291,0.0011560962804394448,0.0030625050952444166,0.8,0.0,132.0,1.1287300338835848e-07,"algorithmic resampling stability, not population inference"

2019,500,auroc,higher,intercept,refit,50,0.02090573862850964,0.01927524741648512,0.02245841215710792,1.0,0.0,0.0,1.7763568394002505e-15,"algorithmic resampling stability, not population inference"

2019,500,auroc,higher,logistic,refit,50,0.018232776153918495,0.01682675868307182,0.020208410327563486,1.0,0.0,0.0,1.7763568394002505e-15,"algorithmic resampling stability, not population inference"

2019,1000,absolute_calibration_intercept_error,lower,intercept,static,50,-0.0653029497626013,-0.08564020056911206,-0.05197086142462436,0.82,0.0,84.0,2.1246773229677274e-09,"algorithmic resampling stability, not population inference"

2019,1000,absolute_calibration_intercept_error,lower,logistic,static,50,-0.06492590169942726,-0.086809486010156,-0.049549154508581106,0.82,0.0,84.0,2.1246773229677274e-09,"algorithmic resampling stability, not population inference"

2019,1000,absolute_calibration_intercept_error,lower,refit,static,50,-0.06907358520456741,-0.08249848842947399,-0.054301760069186904,0.86,0.0,67.0,3.8819791825517314e-10,"algorithmic resampling stability, not population inference"

2019,1000,absolute_calibration_intercept_error,lower,intercept,logistic,50,0.00022675946103899896,-0.0007579589163986995,0.001349754108373999,0.42,0.0,582.0,0.5984627102635613,"algorithmic resampling stability, not population inference"

2019,1000,absolute_calibration_intercept_error,lower,intercept,refit,50,0.0017030093207762503,-0.0008436466401409498,0.0093768167515512,0.4,0.0,541.0,0.35718707191412413,"algorithmic resampling stability, not population inference"

2019,1000,absolute_calibration_intercept_error,lower,logistic,refit,50,0.00215878744010585,-0.003803159957630452,0.0075982569978768,0.44,0.0,579.0,0.5786292137562761,"algorithmic resampling stability, not population inference"

2019,1000,absolute_calibration_slope_error,lower,intercept,static,50,0.0110485387080645,0.0062676366805823,0.0135871157396531,0.04,0.0,3.0,8.881784197001252e-15,"algorithmic resampling stability, not population inference"

2019,1000,absolute_calibration_slope_error,lower,logistic,static,50,0.0280324590503639,0.02210996111319255,0.039248838992605596,0.1,0.0,25.0,1.6058265828178264e-12,"algorithmic resampling stability, not population inference"

2019,1000,absolute_calibration_slope_error,lower,refit,static,50,0.07261215945260815,0.057055395064565896,0.09671541185546771,0.0,0.0,0.0,1.7763568394002505e-15,"algorithmic resampling stability, not population inference"

2019,1000,absolute_calibration_slope_error,lower,intercept,logistic,50,-0.01777794368511045,-0.02729558715545495,-0.004801863682969851,0.7,0.0,216.0,1.9760169596594324e-05,"algorithmic resampling stability, not population inference"

2019,1000,absolute_calibration_slope_error,lower,intercept,refit,50,-0.06291938081734486,-0.08652649447850351,-0.0478564569179887,0.96,0.0,11.0,9.769962616701378e-14,"algorithmic resampling stability, not population inference"

2019,1000,absolute_calibration_slope_error,lower,logistic,refit,50,-0.048692278300556946,-0.0666934497647152,-0.032750148495928395,0.84,0.0,123.0,5.7649630846867694e-08,"algorithmic resampling stability, not population inference"

2019,1000,brier,lower,intercept,static,50,0.00012459572984620942,5.197521259355279e-05,0.00022337558623179867,0.26,0.0,213.0,1.6896629077223224e-05,"algorithmic resampling stability, not population inference"

2019,1000,brier,lower,logistic,static,50,0.00034569457542864973,0.00025624791486665577,0.000490272990844455,0.14,0.0,59.0,1.616786704516926e-10,"algorithmic resampling stability, not population inference"

2019,1000,brier,lower,refit,static,50,0.0021355106300851023,0.0019036075346243048,0.0022353177729190557,0.0,0.0,0.0,1.7763568394002505e-15,"algorithmic resampling stability, not population inference"

2019,1000,brier,lower,intercept,logistic,50,-0.00024510400997589804,-0.0003276471720539964,-9.055211807739577e-05,0.82,0.0,153.0,4.880519277605799e-07,"algorithmic resampling stability, not population inference"

2019,1000,brier,lower,intercept,refit,50,-0.001925703137529003,-0.002018076417431,-0.0017999756433501046,1.0,0.0,0.0,1.7763568394002505e-15,"algorithmic resampling stability, not population inference"

2019,1000,brier,lower,logistic,refit,50,-0.0016599192920565484,-0.0019033430604356028,-0.0014471689084218572,1.0,0.0,0.0,1.7763568394002505e-15,"algorithmic resampling stability, not population inference"

2019,1000,auroc,higher,intercept,static,50,-0.0012620266257951651,-0.0018401471782358847,-0.0008615367189346856,0.12,0.0,77.0,1.080529443697742e-09,"algorithmic resampling stability, not population inference"

2019,1000,auroc,higher,logistic,static,50,-0.002726274349963309,-0.003286532330431191,-0.0015800665705559824,0.12,0.0,30.0,3.6148861681795097e-12,"algorithmic resampling stability, not population inference"

2019,1000,auroc,higher,refit,static,50,-0.014607651507290376,-0.016303156817691122,-0.013472855075120305,0.0,0.0,0.0,1.7763568394002505e-15,"algorithmic resampling stability, not population inference"

2019,1000,auroc,higher,intercept,logistic,50,0.0011479206653920992,0.0006240758186096818,0.0017451178553702928,0.78,0.0,137.0,1.6198182883897516e-07,"algorithmic resampling stability, not population inference"

2019,1000,auroc,higher,intercept,refit,50,0.013498706203116606,0.012413380146363617,0.014653681828861065,1.0,0.0,0.0,1.7763568394002505e-15,"algorithmic resampling stability, not population inference"

2019,1000,auroc,higher,logistic,refit,50,0.012048932138338853,0.011689376157626608,0.0132844310257767,1.0,0.0,0.0,1.7763568394002505e-15,"algorithmic resampling stability, not population inference"

2019,2000,absolute_calibration_intercept_error,lower,intercept,static,50,-0.09238367000925825,-0.09562196601149206,-0.0853258977775268,1.0,0.0,0.0,1.7763568394002505e-15,"algorithmic resampling stability, not population inference"

2019,2000,absolute_calibration_intercept_error,lower,logistic,static,50,-0.0940902656308516,-0.0986796342726958,-0.08105606760837256,1.0,0.0,0.0,1.7763568394002505e-15,"algorithmic resampling stability, not population inference"

2019,2000,absolute_calibration_intercept_error,lower,refit,static,50,-0.09192732525955191,-0.0992238231091319,-0.0798493235208405,1.0,0.0,0.0,1.7763568394002505e-15,"algorithmic resampling stability, not population inference"

2019,2000,absolute_calibration_intercept_error,lower,intercept,logistic,50,0.0005176255643074006,-0.0004360314999996484,0.001129183323170341,0.4,0.0,566.0,0.4963885938865378,"algorithmic resampling stability, not population inference"

2019,2000,absolute_calibration_intercept_error,lower,intercept,refit,50,-0.0016429268818118504,-0.0103562194724904,0.0010873527680209493,0.64,0.0,526.0,0.2866420365460076,"algorithmic resampling stability, not population inference"

2019,2000,absolute_calibration_intercept_error,lower,logistic,refit,50,-0.0026372117121429006,-0.009344031359486946,0.002059927183948251,0.56,0.0,531.0,0.30905100446984335,"algorithmic resampling stability, not population inference"

2019,2000,absolute_calibration_slope_error,lower,intercept,static,50,0.0061884196816677,0.00491031725184475,0.0078651513229324,0.06,0.0,13.0,1.5631940186722204e-13,"algorithmic resampling stability, not population inference"

2019,2000,absolute_calibration_slope_error,lower,logistic,static,50,0.01836438575935795,0.0126687989004578,0.025839513637848498,0.22,0.0,79.0,1.3149694666481082e-09,"algorithmic resampling stability, not population inference"

2019,2000,absolute_calibration_slope_error,lower,refit,static,50,0.0235834115179052,0.014958866262768,0.0304335933309523,0.1,0.0,25.0,1.6058265828178264e-12,"algorithmic resampling stability, not population inference"

2019,2000,absolute_calibration_slope_error,lower,intercept,logistic,50,-0.01332528593939625,-0.0209153013710048,-0.0075633175649161,0.7,0.0,227.0,3.457260659445183e-05,"algorithmic resampling stability, not population inference"

2019,2000,absolute_calibration_slope_error,lower,intercept,refit,50,-0.014766696996775798,-0.022095773435256004,-0.007098960368678701,0.82,0.0,148.0,3.486415511844143e-07,"algorithmic resampling stability, not population inference"

2019,2000,absolute_calibration_slope_error,lower,logistic,refit,50,-0.0078451710264362,-0.0229449335889143,0.008042416576041598,0.56,0.0,565.0,0.49032624846387485,"algorithmic resampling stability, not population inference"

2019,2000,brier,lower,intercept,static,50,2.552040033999836e-06,-3.917288740259983e-05,7.68767674041021e-05,0.48,0.0,500.0,0.18768390417341507,"algorithmic resampling stability, not population inference"

2019,2000,brier,lower,logistic,static,50,0.00012907243377880545,7.619082835361168e-05,0.00022118409931095456,0.22,0.0,186.0,3.801371960321376e-06,"algorithmic resampling stability, not population inference"

2019,2000,brier,lower,refit,static,50,0.0011438581280500057,0.0010096729123026532,0.0013247696934593003,0.0,0.0,0.0,1.7763568394002505e-15,"algorithmic resampling stability, not population inference"

2019,2000,brier,lower,intercept,logistic,50,-0.00013715441047625065,-0.00018614298402780394,-6.563639271920524e-05,0.78,0.0,139.0,1.8673260271384606e-07,"algorithmic resampling stability, not population inference"

2019,2000,brier,lower,intercept,refit,50,-0.001146568555618649,-0.0012684623147085489,-0.0009692866263198985,1.0,0.0,0.0,1.7763568394002505e-15,"algorithmic resampling stability, not population inference"

2019,2000,brier,lower,logistic,refit,50,-0.0010392852337640021,-0.001185450463159099,-0.0008915889914061018,1.0,0.0,0.0,1.7763568394002505e-15,"algorithmic resampling stability, not population inference"

2019,2000,auroc,higher,intercept,static,50,-0.0009249366916701529,-0.001349823886339252,-0.0006903747822658013,0.12,0.0,39.0,1.34718902700115e-11,"algorithmic resampling stability, not population inference"

2019,2000,auroc,higher,logistic,static,50,-0.0019779654999476093,-0.0022315068288581985,-0.0016528621901005236,0.0,0.0,0.0,1.7763568394002505e-15,"algorithmic resampling stability, not population inference"

2019,2000,auroc,higher,refit,static,50,-0.009879228231489956,-0.011060726286111655,-0.008359761494480034,0.02,0.0,1.0,3.552713678800501e-15,"algorithmic resampling stability, not population inference"

2019,2000,auroc,higher,intercept,logistic,50,0.0010611430981475323,0.0006307415630848467,0.0014648965238421852,0.86,0.0,88.0,3.0854181432005134e-09,"algorithmic resampling stability, not population inference"

2019,2000,auroc,higher,intercept,refit,50,0.008297819619903002,0.006769485266443387,0.009401390127990683,0.98,0.0,1.0,3.552713678800501e-15,"algorithmic resampling stability, not population inference"

2019,2000,auroc,higher,logistic,refit,50,0.006776462585519727,0.006168392987892568,0.008134377866189313,0.98,0.0,2.0,5.329070518200751e-15,"algorithmic resampling stability, not population inference"

2019,4000,absolute_calibration_intercept_error,lower,intercept,static,50,-0.09557695431589305,-0.1026897389438519,-0.0906972978937932,1.0,0.0,0.0,1.7763568394002505e-15,"algorithmic resampling stability, not population inference"

2019,4000,absolute_calibration_intercept_error,lower,logistic,static,50,-0.0945365303251395,-0.1001650533549699,-0.09074303655306705,1.0,0.0,0.0,1.7763568394002505e-15,"algorithmic resampling stability, not population inference"

2019,4000,absolute_calibration_intercept_error,lower,refit,static,50,-0.09547253307749931,-0.09993625610539256,-0.0892803076060346,1.0,0.0,0.0,1.7763568394002505e-15,"algorithmic resampling stability, not population inference"

2019,4000,absolute_calibration_intercept_error,lower,intercept,logistic,50,-0.00012914626149820039,-0.0005414076318744996,0.0006828301394652021,0.5,0.0,596.0,0.6947463342363385,"algorithmic resampling stability, not population inference"

2019,4000,absolute_calibration_intercept_error,lower,intercept,refit,50,-0.0029017989459676017,-0.006916267496173099,0.0028461477846738518,0.58,0.0,482.0,0.13549845162767582,"algorithmic resampling stability, not population inference"

2019,4000,absolute_calibration_intercept_error,lower,logistic,refit,50,-0.0019193183444895477,-0.00739583237263455,0.00384575636169135,0.56,0.0,517.0,0.24908489906916564,"algorithmic resampling stability, not population inference"

2019,4000,absolute_calibration_slope_error,lower,intercept,static,50,0.0027964648557146504,0.002220179716338801,0.0046064074997675996,0.08,0.0,28.0,2.6343371928305714e-12,"algorithmic resampling stability, not population inference"

2019,4000,absolute_calibration_slope_error,lower,logistic,static,50,0.00627899317945475,0.004339253217742401,0.00887841684968565,0.22,0.0,143.0,2.4721383162784605e-07,"algorithmic resampling stability, not population inference"

2019,4000,absolute_calibration_slope_error,lower,refit,static,50,0.00746266332225725,0.0046999607132997495,0.0117900868408219,0.22,0.0,122.0,5.3406212785489515e-08,"algorithmic resampling stability, not population inference"

2019,4000,absolute_calibration_slope_error,lower,intercept,logistic,50,-0.0028780438971125494,-0.005953763098382799,2.544556584369896e-05,0.62,0.0,347.0,0.004459422606641894,"algorithmic resampling stability, not population inference"

2019,4000,absolute_calibration_slope_error,lower,intercept,refit,50,-0.0045473248962045005,-0.006687978906915201,-0.0003531125435846504,0.64,0.0,328.0,0.002356807144249018,"algorithmic resampling stability, not population inference"

2019,4000,absolute_calibration_slope_error,lower,logistic,refit,50,-0.0001731113590910001,-0.005596535540990899,0.0070648789309164005,0.5,0.0,604.0,0.7521534307154649,"algorithmic resampling stability, not population inference"

2019,4000,brier,lower,intercept,static,50,-3.8139633465592016e-05,-6.0696537821300356e-05,-1.3807482458445819e-05,0.68,0.0,387.0,0.014890897756602683,"algorithmic resampling stability, not population inference"

2019,4000,brier,lower,logistic,static,50,0.00011170419900185385,7.092370147990246e-05,0.00015120907178880344,0.18,0.0,169.0,1.3667570399888973e-06,"algorithmic resampling stability, not population inference"

2019,4000,brier,lower,refit,static,50,0.0007524758935507567,0.0006642956632401595,0.0008456363638657052,0.04,0.0,7.0,3.375077994860476e-14,"algorithmic resampling stability, not population inference"

2019,4000,brier,lower,intercept,logistic,50,-0.00011438513734279843,-0.000173493484409995,-8.363537487664913e-05,0.9,0.0,35.0,7.65609797781508e-12,"algorithmic resampling stability, not population inference"

2019,4000,brier,lower,intercept,refit,50,-0.0008052452840518545,-0.000859443846324151,-0.0006521181453092023,0.96,0.0,5.0,1.7763568394002505e-14,"algorithmic resampling stability, not population inference"

2019,4000,brier,lower,logistic,refit,50,-0.0006199517777119995,-0.0007180316335847016,-0.0005027885751119066,0.96,0.0,9.0,5.861977570020827e-14,"algorithmic resampling stability, not population inference"

2019,4000,auroc,higher,intercept,static,50,-0.0007776365965494469,-0.0010146133482655806,-0.0005553295532410574,0.16,0.0,94.0,5.310079487230723e-09,"algorithmic resampling stability, not population inference"

2019,4000,auroc,higher,logistic,static,50,-0.0017232240387297093,-0.0020050411560318393,-0.0012467098203987298,0.04,0.0,7.0,3.375077994860476e-14,"algorithmic resampling stability, not population inference"

2019,4000,auroc,higher,refit,static,50,-0.00548404686861903,-0.005930203338884943,-0.004441384204746934,0.0,0.0,0.0,1.7763568394002505e-15,"algorithmic resampling stability, not population inference"

2019,4000,auroc,higher,intercept,logistic,50,0.000843751397571646,0.0006169274478174946,0.0010982637403122095,0.86,0.0,40.0,1.5448975432263978e-11,"algorithmic resampling stability, not population inference"

2019,4000,auroc,higher,intercept,refit,50,0.004688582257741936,0.003993187283325206,0.005336900771264863,1.0,0.0,0.0,1.7763568394002505e-15,"algorithmic resampling stability, not population inference"

2019,4000,auroc,higher,logistic,refit,50,0.0037389810280183955,0.003069618054630463,0.004175636382281411,0.98,0.0,4.0,1.2434497875801753e-14,"algorithmic resampling stability, not population inference"

2021,250,absolute_calibration_intercept_error,lower,intercept,static,50,0.0643253411954631,0.027783831344288,0.10624573787291329,0.24,0.0,112.0,2.434094703573919e-08,"algorithmic resampling stability, not population inference"

2021,250,absolute_calibration_intercept_error,lower,logistic,static,50,0.061261744755808804,0.034652614079332504,0.10828967202704541,0.26,0.0,112.0,2.434094703573919e-08,"algorithmic resampling stability, not population inference"

2021,250,absolute_calibration_intercept_error,lower,refit,static,50,0.06606633592120739,0.0268770381738208,0.12022092736612494,0.22,0.0,89.0,3.3822118439275073e-09,"algorithmic resampling stability, not population inference"

2021,250,absolute_calibration_intercept_error,lower,intercept,logistic,50,-0.0010501552034775513,-0.00874205530868145,0.004228553175840848,0.5,0.0,484.0,0.1406841997467243,"algorithmic resampling stability, not population inference"

2021,250,absolute_calibration_intercept_error,lower,intercept,refit,50,-0.0080864139052829,-0.0238342607860626,0.0048655340790354044,0.58,0.0,533.0,0.31832456588976044,"algorithmic resampling stability, not population inference"

2021,250,absolute_calibration_intercept_error,lower,logistic,refit,50,-0.0047510836047732034,-0.020503805205329198,0.014143133979041654,0.6,0.0,583.0,0.6051405400941228,"algorithmic resampling stability, not population inference"

2021,250,absolute_calibration_slope_error,lower,intercept,static,50,0.0017920906485873493,-0.0014986785330763008,0.007403793331750799,0.4,0.0,422.0,0.037166605079788084,"algorithmic resampling stability, not population inference"

2021,250,absolute_calibration_slope_error,lower,logistic,static,50,0.09640880281793984,0.0623313673930562,0.11088438747261159,0.08,0.0,16.0,3.0020430585864233e-13,"algorithmic resampling stability, not population inference"

2021,250,absolute_calibration_slope_error,lower,refit,static,50,0.2451719615010913,0.2183160962770932,0.27432371466940764,0.0,0.0,0.0,1.7763568394002505e-15,"algorithmic resampling stability, not population inference"

2021,250,absolute_calibration_slope_error,lower,intercept,logistic,50,-0.06685626645702214,-0.107148838361276,-0.0456961616085414,0.86,0.0,69.0,4.792291008470784e-10,"algorithmic resampling stability, not population inference"

2021,250,absolute_calibration_slope_error,lower,intercept,refit,50,-0.234172673723106,-0.2835459468449082,-0.2048349377945553,0.98,0.0,1.0,3.552713678800501e-15,"algorithmic resampling stability, not population inference"

2021,250,absolute_calibration_slope_error,lower,logistic,refit,50,-0.1365691390848003,-0.2115558630151505,-0.10509542379994528,0.94,0.0,42.0,2.022026990289305e-11,"algorithmic resampling stability, not population inference"

2021,250,brier,lower,intercept,static,50,0.0007169041802820506,0.0005548394141610055,0.001001806019666103,0.04,0.0,3.0,8.881784197001252e-15,"algorithmic resampling stability, not population inference"

2021,250,brier,lower,logistic,static,50,0.0016342733708606533,0.001323649346304398,0.0022383046527661016,0.0,0.0,0.0,1.7763568394002505e-15,"algorithmic resampling stability, not population inference"

2021,250,brier,lower,refit,static,50,0.0053515094569589056,0.005061358991211906,0.00602044174105925,0.0,0.0,0.0,1.7763568394002505e-15,"algorithmic resampling stability, not population inference"

2021,250,brier,lower,intercept,logistic,50,-0.0007724618282708984,-0.0009803514971696015,-0.0005175402913975002,0.94,0.0,13.0,1.5631940186722204e-13,"algorithmic resampling stability, not population inference"

2021,250,brier,lower,intercept,refit,50,-0.004455883104365101,-0.004822103134724004,-0.004218738484700051,1.0,0.0,0.0,1.7763568394002505e-15,"algorithmic resampling stability, not population inference"

2021,250,brier,lower,logistic,refit,50,-0.0040452154810702995,-0.00422493943308825,-0.0034302763251770896,0.98,0.0,1.0,3.552713678800501e-15,"algorithmic resampling stability, not population inference"

2021,250,auroc,higher,intercept,static,50,-0.004468156731158468,-0.005884388286603814,-0.0038960460494436022,0.0,0.0,0.0,1.7763568394002505e-15,"algorithmic resampling stability, not population inference"

2021,250,auroc,higher,logistic,static,50,-0.009231843858704125,-0.010664237119573872,-0.007392093362765528,0.0,0.0,0.0,1.7763568394002505e-15,"algorithmic resampling stability, not population inference"

2021,250,auroc,higher,refit,static,50,-0.0355047796313398,-0.03823686298767026,-0.02708888720995617,0.0,0.0,0.0,1.7763568394002505e-15,"algorithmic resampling stability, not population inference"

2021,250,auroc,higher,intercept,logistic,50,0.004387597819554445,0.0024289492416391667,0.005849522155584985,0.88,0.0,49.0,4.9604764740251994e-11,"algorithmic resampling stability, not population inference"

2021,250,auroc,higher,intercept,refit,50,0.02840992497235345,0.023965569275038116,0.03197228224255555,1.0,0.0,0.0,1.7763568394002505e-15,"algorithmic resampling stability, not population inference"

2021,250,auroc,higher,logistic,refit,50,0.02356007745800881,0.020919803171939644,0.02677471849393326,1.0,0.0,0.0,1.7763568394002505e-15,"algorithmic resampling stability, not population inference"

2021,500,absolute_calibration_intercept_error,lower,intercept,static,50,0.037906607072078304,0.0187249249580181,0.0739563382172308,0.26,0.0,138.0,1.7394549622906652e-07,"algorithmic resampling stability, not population inference"

2021,500,absolute_calibration_intercept_error,lower,logistic,static,50,0.04160170456358315,0.02632018073963375,0.061810674467332405,0.24,0.0,121.0,4.945666454148068e-08,"algorithmic resampling stability, not population inference"

2021,500,absolute_calibration_intercept_error,lower,refit,static,50,0.039655396782113404,0.025724854800699897,0.06036436409840685,0.16,0.0,70.0,5.318447904301138e-10,"algorithmic resampling stability, not population inference"

2021,500,absolute_calibration_intercept_error,lower,intercept,logistic,50,-0.001477580792334253,-0.0038081165757256983,0.0022251477863282007,0.58,0.0,573.0,0.5398998476772991,"algorithmic resampling stability, not population inference"

2021,500,absolute_calibration_intercept_error,lower,intercept,refit,50,-0.0009844452867136082,-0.014589655420309652,0.004754635715135848,0.54,0.0,586.0,0.6253677971440439,"algorithmic resampling stability, not population inference"

2021,500,absolute_calibration_intercept_error,lower,logistic,refit,50,-0.007961415303731345,-0.018249474363946954,0.011252737929610901,0.56,0.0,605.0,0.7594325529338448,"algorithmic resampling stability, not population inference"

2021,500,absolute_calibration_slope_error,lower,intercept,static,50,-0.006344185248303701,-0.00746557019441055,-0.0039291411894096,0.8,0.0,237.0,5.639617199193481e-05,"algorithmic resampling stability, not population inference"

2021,500,absolute_calibration_slope_error,lower,logistic,static,50,0.053715700858121745,0.031679619079117996,0.06256206279326554,0.12,0.0,31.0,4.218847493575595e-12,"algorithmic resampling stability, not population inference"

2021,500,absolute_calibration_slope_error,lower,refit,static,50,0.11272294303544131,0.1062343872617553,0.1384653157697761,0.0,0.0,0.0,1.7763568394002505e-15,"algorithmic resampling stability, not population inference"

2021,500,absolute_calibration_slope_error,lower,intercept,logistic,50,-0.05254073343974885,-0.0715056479864819,-0.0371929670525961,0.92,0.0,15.0,2.433608869978343e-13,"algorithmic resampling stability, not population inference"

2021,500,absolute_calibration_slope_error,lower,intercept,refit,50,-0.12350548626177645,-0.1471937883348458,-0.1078804584515229,0.98,0.0,6.0,2.4868995751603507e-14,"algorithmic resampling stability, not population inference"

2021,500,absolute_calibration_slope_error,lower,logistic,refit,50,-0.0807018881811451,-0.1034127952505564,-0.035402703779718506,0.8,0.0,184.0,3.38235121510877e-06,"algorithmic resampling stability, not population inference"

2021,500,brier,lower,intercept,static,50,0.0003330796572540498,0.00020548696711992085,0.0004965392199629548,0.22,0.0,76.0,9.785132704109856e-10,"algorithmic resampling stability, not population inference"

2021,500,brier,lower,logistic,static,50,0.0008338185023565037,0.0006619275442609485,0.001052850502913899,0.0,0.0,0.0,1.7763568394002505e-15,"algorithmic resampling stability, not population inference"

2021,500,brier,lower,refit,static,50,0.0029369331937023,0.0028096709327410513,0.0033449307268130987,0.0,0.0,0.0,1.7763568394002505e-15,"algorithmic resampling stability, not population inference"

2021,500,brier,lower,intercept,logistic,50,-0.0004202036681576482,-0.0005752475972826479,-0.00034732613549345176,0.98,0.0,7.0,3.375077994860476e-14,"algorithmic resampling stability, not population inference"

2021,500,brier,lower,intercept,refit,50,-0.002671588695603154,-0.0028974206205799047,-0.0024944825253037026,1.0,0.0,0.0,1.7763568394002505e-15,"algorithmic resampling stability, not population inference"

2021,500,brier,lower,logistic,refit,50,-0.0023069410350520445,-0.0025166617045252956,-0.001976579658356195,0.96,0.0,5.0,1.7763568394002505e-14,"algorithmic resampling stability, not population inference"

2021,500,auroc,higher,intercept,static,50,-0.002076173061531572,-0.002464038857254436,-0.0015197368038067927,0.06,0.0,8.0,4.440892098500626e-14,"algorithmic resampling stability, not population inference"

2021,500,auroc,higher,logistic,static,50,-0.004766362528501711,-0.005777936877249756,-0.0038889093176810077,0.0,0.0,0.0,1.7763568394002505e-15,"algorithmic resampling stability, not population inference"

2021,500,auroc,higher,refit,static,50,-0.01651057128380229,-0.017399372014133607,-0.014140895977540258,0.0,0.0,0.0,1.7763568394002505e-15,"algorithmic resampling stability, not population inference"

2021,500,auroc,higher,intercept,logistic,50,0.0023132094951774373,0.0015469904790446076,0.0035357443494561935,0.78,0.0,136.0,1.507920117660433e-07,"algorithmic resampling stability, not population inference"

2021,500,auroc,higher,intercept,refit,50,0.013937782070601812,0.011672097817213123,0.015795413947935155,1.0,0.0,0.0,1.7763568394002505e-15,"algorithmic resampling stability, not population inference"

2021,500,auroc,higher,logistic,refit,50,0.012629370028771791,0.010516044295325624,0.01428410470974023,1.0,0.0,0.0,1.7763568394002505e-15,"algorithmic resampling stability, not population inference"

2021,1000,absolute_calibration_intercept_error,lower,intercept,static,50,0.025034675896600002,0.009377873900505002,0.039652841858904955,0.3,0.0,213.0,1.6896629077223224e-05,"algorithmic resampling stability, not population inference"

2021,1000,absolute_calibration_intercept_error,lower,logistic,static,50,0.023565576159506,0.0097073662090594,0.0413921326331768,0.3,0.0,214.0,1.7805245978053108e-05,"algorithmic resampling stability, not population inference"

2021,1000,absolute_calibration_intercept_error,lower,refit,static,50,0.0217216326541045,0.009470823083633447,0.0405885592982737,0.28,0.0,225.0,3.128163219123792e-05,"algorithmic resampling stability, not population inference"

2021,1000,absolute_calibration_intercept_error,lower,intercept,logistic,50,-0.0012660348280728005,-0.002482226473455501,-1.8906412809700877e-05,0.64,0.0,378.0,0.011529687649083797,"algorithmic resampling stability, not population inference"

2021,1000,absolute_calibration_intercept_error,lower,intercept,refit,50,0.0036069392952506486,-0.005875481192633549,0.009492746054423094,0.42,0.0,583.0,0.6051405400941228,"algorithmic resampling stability, not population inference"

2021,1000,absolute_calibration_intercept_error,lower,logistic,refit,50,0.003909691381099898,-0.0055949870411324,0.014356590238350946,0.42,0.0,545.0,0.3776732059941086,"algorithmic resampling stability, not population inference"

2021,1000,absolute_calibration_slope_error,lower,intercept,static,50,-0.005797535555747451,-0.0084746895959913,-0.002963295438895801,0.84,0.0,165.0,1.0633089786438177e-06,"algorithmic resampling stability, not population inference"

2021,1000,absolute_calibration_slope_error,lower,logistic,static,50,0.035145525686197804,0.02198120140251485,0.04913137729235739,0.14,0.0,50.0,5.610267805877811e-11,"algorithmic resampling stability, not population inference"

2021,1000,absolute_calibration_slope_error,lower,refit,static,50,0.03958972358160455,0.02201919053144295,0.057370230784772394,0.18,0.0,68.0,4.3148595807451784e-10,"algorithmic resampling stability, not population inference"

2021,1000,absolute_calibration_slope_error,lower,intercept,logistic,50,-0.04175912545538285,-0.0552429314212211,-0.02892164441233455,0.9,0.0,28.0,2.6343371928305714e-12,"algorithmic resampling stability, not population inference"

2021,1000,absolute_calibration_slope_error,lower,intercept,refit,50,-0.0492817220605435,-0.06348278242005286,-0.023085104771212596,0.88,0.0,42.0,2.022026990289305e-11,"algorithmic resampling stability, not population inference"

2021,1000,absolute_calibration_slope_error,lower,logistic,refit,50,-0.0052165620755434475,-0.052366995563981805,0.018739562943437902,0.56,0.0,594.0,0.6806447467771797,"algorithmic resampling stability, not population inference"

2021,1000,brier,lower,intercept,static,50,0.0002032072160353532,7.230182307239874e-05,0.00031698390064485715,0.2,0.0,113.0,2.6378053519238165e-08,"algorithmic resampling stability, not population inference"

2021,1000,brier,lower,logistic,static,50,0.0004075213042451023,0.0002884845004119535,0.0006559382925506035,0.08,0.0,14.0,1.9539925233402755e-13,"algorithmic resampling stability, not population inference"

2021,1000,brier,lower,refit,static,50,0.001720645697378051,0.0015342360054609039,0.0018723899677849098,0.02,0.0,1.0,3.552713678800501e-15,"algorithmic resampling stability, not population inference"

2021,1000,brier,lower,intercept,logistic,50,-0.00021654592442630488,-0.0003182530480766027,-0.0001235811325760519,0.88,0.0,33.0,5.707434524993005e-12,"algorithmic resampling stability, not population inference"

2021,1000,brier,lower,intercept,refit,50,-0.0015188197731909464,-0.0016750303636296482,-0.001348759264621198,0.98,0.0,1.0,3.552713678800501e-15,"algorithmic resampling stability, not population inference"

2021,1000,brier,lower,logistic,refit,50,-0.0012912556071514036,-0.001400570571783194,-0.001070889708206145,0.98,0.0,2.0,5.329070518200751e-15,"algorithmic resampling stability, not population inference"

2021,1000,auroc,higher,intercept,static,50,-0.0012511626220807481,-0.0016785715221838693,-0.000719238205321937,0.14,0.0,52.0,7.151434999741468e-11,"algorithmic resampling stability, not population inference"

2021,1000,auroc,higher,logistic,static,50,-0.0031717193851044656,-0.00382765313078004,-0.0024486902913181674,0.04,0.0,3.0,8.881784197001252e-15,"algorithmic resampling stability, not population inference"

2021,1000,auroc,higher,refit,static,50,-0.008411748170670141,-0.009961757005778504,-0.007530614582734851,0.04,0.0,3.0,8.881784197001252e-15,"algorithmic resampling stability, not population inference"

2021,1000,auroc,higher,intercept,logistic,50,0.0019086545488538609,0.0008024701820402713,0.002609800645831384,0.82,0.0,102.0,1.0642100534141719e-08,"algorithmic resampling stability, not population inference"

2021,1000,auroc,higher,intercept,refit,50,0.007183904169089483,0.006476212821233451,0.008403012191449566,0.96,0.0,5.0,1.7763568394002505e-14,"algorithmic resampling stability, not population inference"

2021,1000,auroc,higher,logistic,refit,50,0.005455185311645516,0.004386589664655882,0.006137265352193166,0.9,0.0,29.0,3.0890845437170356e-12,"algorithmic resampling stability, not population inference"

2021,2000,absolute_calibration_intercept_error,lower,intercept,static,50,0.010474483053191298,0.0032354414645472986,0.018348523746324997,0.34,0.0,321.0,0.0018425334424101436,"algorithmic resampling stability, not population inference"

2021,2000,absolute_calibration_intercept_error,lower,logistic,static,50,0.011329355144240653,0.0011019471529482988,0.01986329818455015,0.36,0.0,302.0,0.0009149367393188612,"algorithmic resampling stability, not population inference"

2021,2000,absolute_calibration_intercept_error,lower,refit,static,50,0.011067555136064802,0.001505729168187999,0.022327450547792352,0.34,0.0,311.0,0.001282241835095732,"algorithmic resampling stability, not population inference"

2021,2000,absolute_calibration_intercept_error,lower,intercept,logistic,50,-0.0016044749175095456,-0.0019014119488501013,-0.0007583173656354504,0.68,0.0,394.0,0.01806109651468013,"algorithmic resampling stability, not population inference"

2021,2000,absolute_calibration_intercept_error,lower,intercept,refit,50,-0.002647799072258549,-0.012237891096886895,0.005943220318943201,0.54,0.0,555.0,0.43191110415215306,"algorithmic resampling stability, not population inference"

2021,2000,absolute_calibration_intercept_error,lower,logistic,refit,50,0.0009164946757703497,-0.011716446396723496,0.006911746326775194,0.46,0.0,587.0,0.6321732707310517,"algorithmic resampling stability, not population inference"

2021,2000,absolute_calibration_slope_error,lower,intercept,static,50,-0.00600099608817245,-0.007270244310773601,-0.004824801350454,0.98,0.0,8.0,4.440892098500626e-14,"algorithmic resampling stability, not population inference"

2021,2000,absolute_calibration_slope_error,lower,logistic,static,50,0.0135671844948177,0.0079139284878035,0.025561791179102997,0.18,0.0,83.0,1.9326371614170057e-09,"algorithmic resampling stability, not population inference"

2021,2000,absolute_calibration_slope_error,lower,refit,static,50,0.0056368519746504,-0.0005086652325835012,0.009830233174455867,0.42,0.0,377.0,0.011200519477185367,"algorithmic resampling stability, not population inference"

2021,2000,absolute_calibration_slope_error,lower,intercept,logistic,50,-0.0184867788890324,-0.030260723211457702,-0.01303312969361425,0.94,0.0,16.0,3.0020430585864233e-13,"algorithmic resampling stability, not population inference"

2021,2000,absolute_calibration_slope_error,lower,intercept,refit,50,-0.01010377450493995,-0.014279601027302401,-0.0048758900173940505,0.82,0.0,119.0,4.2364032282193875e-08,"algorithmic resampling stability, not population inference"

2021,2000,absolute_calibration_slope_error,lower,logistic,refit,50,0.006215660105726599,0.0039167182568765006,0.0107533015040285,0.26,0.0,282.0,0.0004153236253312542,"algorithmic resampling stability, not population inference"

2021,2000,brier,lower,intercept,static,50,0.00017841759383375316,0.00015280406706630334,0.00021965010025210008,0.1,0.0,25.0,1.6058265828178264e-12,"algorithmic resampling stability, not population inference"

2021,2000,brier,lower,logistic,static,50,0.00033238497050105953,0.0002850825159176548,0.0004493925807599053,0.04,0.0,7.0,3.375077994860476e-14,"algorithmic resampling stability, not population inference"

2021,2000,brier,lower,refit,static,50,0.0008246260400780539,0.0007020993511355558,0.0009821199315821066,0.06,0.0,13.0,1.5631940186722204e-13,"algorithmic resampling stability, not population inference"

2021,2000,brier,lower,intercept,logistic,50,-0.0001419845506123521,-0.00018623678185498982,-9.901560401374965e-05,0.9,0.0,51.0,6.337863567296154e-11,"algorithmic resampling stability, not population inference"

2021,2000,brier,lower,intercept,refit,50,-0.0005764044390239517,-0.0008417719209427993,-0.0004306688430463085,0.88,0.0,43.0,2.3080204414327454e-11,"algorithmic resampling stability, not population inference"

2021,2000,brier,lower,logistic,refit,50,-0.0004694217145041965,-0.0006677757969041909,-0.00026909738250530113,0.74,0.0,166.0,1.1326234101716182e-06,"algorithmic resampling stability, not population inference"

2021,2000,auroc,higher,intercept,static,50,-0.0009473258981415045,-0.0011940773774572477,-0.0007541952082945613,0.1,0.0,26.0,1.8989254613188677e-12,"algorithmic resampling stability, not population inference"

2021,2000,auroc,higher,logistic,static,50,-0.0020526151914331825,-0.002606671137238392,-0.0018843027830601766,0.02,0.0,2.0,5.329070518200751e-15,"algorithmic resampling stability, not population inference"

2021,2000,auroc,higher,refit,static,50,-0.003162637886511832,-0.004047385396045988,-0.002431586266426533,0.12,0.0,37.0,1.0192735544478637e-11,"algorithmic resampling stability, not population inference"

2021,2000,auroc,higher,intercept,logistic,50,0.001135843792488045,0.0005929316803199303,0.0017546962331084215,0.84,0.0,88.0,3.0854181432005134e-09,"algorithmic resampling stability, not population inference"

2021,2000,auroc,higher,intercept,refit,50,0.002155787282668409,0.0012674624919974953,0.0028938591944787095,0.82,0.0,137.0,1.6198182883897516e-07,"algorithmic resampling stability, not population inference"

2021,2000,auroc,higher,logistic,refit,50,0.0012484596725582109,-0.00010578999869748085,0.002079226562105241,0.62,0.0,373.0,0.009964281765361349,"algorithmic resampling stability, not population inference"

2021,4000,absolute_calibration_intercept_error,lower,intercept,static,50,-0.0084277560544213,-0.01577425450789955,0.004556731709635099,0.58,0.0,453.0,0.07556206690378708,"algorithmic resampling stability, not population inference"

2021,4000,absolute_calibration_intercept_error,lower,logistic,static,50,-0.00776990052258995,-0.0153540825589475,0.004826950247467401,0.58,0.0,470.0,0.10737600575610173,"algorithmic resampling stability, not population inference"

2021,4000,absolute_calibration_intercept_error,lower,refit,static,50,-0.0037841012970387007,-0.0111998359528766,0.005769222417057903,0.6,0.0,592.0,0.6666519727454681,"algorithmic resampling stability, not population inference"

2021,4000,absolute_calibration_intercept_error,lower,intercept,logistic,50,-0.00037666145702175066,-0.0009092633099824013,0.00010140034207520039,0.6,0.0,388.0,0.01531204751384152,"algorithmic resampling stability, not population inference"

2021,4000,absolute_calibration_intercept_error,lower,intercept,refit,50,-0.005480239934996951,-0.012700416546520904,0.0019768511542716,0.6,0.0,437.0,0.052989788310034314,"algorithmic resampling stability, not population inference"

2021,4000,absolute_calibration_intercept_error,lower,logistic,refit,50,-0.0044082838129284,-0.010546793806011501,0.0026961640325137,0.58,0.0,455.0,0.07885790741956278,"algorithmic resampling stability, not population inference"

2021,4000,absolute_calibration_slope_error,lower,intercept,static,50,-0.002361713519982901,-0.003134322654473401,-0.0017041159594197502,0.88,0.0,48.0,4.3806736016449577e-11,"algorithmic resampling stability, not population inference"

2021,4000,absolute_calibration_slope_error,lower,logistic,static,50,0.0050630001302178,0.00014533008272489979,0.0113128428791685,0.36,0.0,341.0,0.0036632546526647047,"algorithmic resampling stability, not population inference"

2021,4000,absolute_calibration_slope_error,lower,refit,static,50,0.00867352370006375,-0.0010033464747258,0.0138021473633491,0.4,0.0,291.0,0.0005966978973450665,"algorithmic resampling stability, not population inference"

2021,4000,absolute_calibration_slope_error,lower,intercept,logistic,50,-0.007794584597576002,-0.012907460101331002,-0.0039202343950908,0.7,0.0,222.0,2.6885878247284722e-05,"algorithmic resampling stability, not population inference"

2021,4000,absolute_calibration_slope_error,lower,intercept,refit,50,-0.0107860194042389,-0.0158409921946503,-0.0014725632715413004,0.66,0.0,208.0,1.2965142769161275e-05,"algorithmic resampling stability, not population inference"

2021,4000,absolute_calibration_slope_error,lower,logistic,refit,50,0.0015758619450790996,-0.0034071132947279016,0.006955852133477103,0.44,0.0,600.0,0.7232586770768918,"algorithmic resampling stability, not population inference"

2021,4000,brier,lower,intercept,static,50,7.54829845813973e-05,5.313681957255095e-05,0.00011970174731920635,0.18,0.0,159.0,7.239255257474042e-07,"algorithmic resampling stability, not population inference"

2021,4000,brier,lower,logistic,static,50,0.0001899528248558574,0.00013610514018440323,0.00023108936540990055,0.12,0.0,29.0,3.0890845437170356e-12,"algorithmic resampling stability, not population inference"

2021,4000,brier,lower,refit,static,50,6.034088892144934e-05,-2.573207294229163e-05,0.00019000489225190487,0.4,0.0,400.0,0.02122367163684835,"algorithmic resampling stability, not population inference"

2021,4000,brier,lower,intercept,logistic,50,-8.823109542560603e-05,-0.00013601590912229455,-6.43496906801988e-05,0.92,0.0,39.0,1.34718902700115e-11,"algorithmic resampling stability, not population inference"

2021,4000,brier,lower,intercept,refit,50,2.3846478238248592e-05,-7.046076705934795e-05,0.00010173718938959186,0.46,0.0,615.0,0.8332778036429911,"algorithmic resampling stability, not population inference"

2021,4000,brier,lower,logistic,refit,50,0.00014000278080189554,2.196737120589709e-05,0.0002415599182523931,0.36,0.0,484.0,0.1406841997467243,"algorithmic resampling stability, not population inference"

2021,4000,auroc,higher,intercept,static,50,-0.0004436325395674179,-0.000662952305058112,-0.0002979129949484127,0.16,0.0,69.0,4.792291008470784e-10,"algorithmic resampling stability, not population inference"

2021,4000,auroc,higher,logistic,static,50,-0.001240783227607789,-0.001674957545995559,-0.0010319139604236804,0.04,0.0,7.0,3.375077994860476e-14,"algorithmic resampling stability, not population inference"

2021,4000,auroc,higher,refit,static,50,0.001013903269474814,0.00038820338305867175,0.0017595871362853233,0.76,0.0,259.0,0.00015581408102782746,"algorithmic resampling stability, not population inference"

2021,4000,auroc,higher,intercept,logistic,50,0.0007389826105564379,0.0004621244750055986,0.001236378313479225,0.9,0.0,37.0,1.0192735544478637e-11,"algorithmic resampling stability, not population inference"

2021,4000,auroc,higher,intercept,refit,50,-0.001629780064062003,-0.002162440273925763,-0.0012327323833911663,0.18,0.0,141.0,2.1499046276574063e-07,"algorithmic resampling stability, not population inference"

2021,4000,auroc,higher,logistic,refit,50,-0.002610410961436882,-0.0033113125890396056,-0.0019837918168092994,0.1,0.0,33.0,5.707434524993005e-12,"algorithmic resampling stability, not population inference"

2023,250,absolute_calibration_intercept_error,lower,intercept,static,50,-0.034023911243495554,-0.0705510345421181,0.009196218115215787,0.62,0.0,631.0,0.9542249738357711,"algorithmic resampling stability, not population inference"

2023,250,absolute_calibration_intercept_error,lower,logistic,static,50,-0.018087984008321553,-0.07437318620480911,0.027896077295071042,0.54,0.0,602.0,0.7376605378038441,"algorithmic resampling stability, not population inference"

2023,250,absolute_calibration_intercept_error,lower,refit,static,50,-0.0348765482851303,-0.07664680686507411,-0.0008510526863177575,0.64,0.0,553.0,0.42072286282100535,"algorithmic resampling stability, not population inference"

2023,250,absolute_calibration_intercept_error,lower,intercept,logistic,50,-0.0006295597919941422,-0.0112866263092707,0.0074500148974024,0.5,0.0,552.0,0.4151920088524754,"algorithmic resampling stability, not population inference"

2023,250,absolute_calibration_intercept_error,lower,intercept,refit,50,0.0224511607025317,-0.00272981837546267,0.0514707444583382,0.36,0.0,509.0,0.21866240175859453,"algorithmic resampling stability, not population inference"

2023,250,absolute_calibration_intercept_error,lower,logistic,refit,50,0.020254022950505095,-0.0013935456547376004,0.040898711242331194,0.38,0.0,475.0,0.11848533640686121,"algorithmic resampling stability, not population inference"

2023,250,absolute_calibration_slope_error,lower,intercept,static,50,0.0340752020424094,0.0127716666021592,0.0504916807605985,0.16,0.0,84.0,2.1246773229677274e-09,"algorithmic resampling stability, not population inference"

2023,250,absolute_calibration_slope_error,lower,logistic,static,50,0.13266983967203866,0.1047668458675045,0.15945593917557943,0.02,0.0,2.0,5.329070518200751e-15,"algorithmic resampling stability, not population inference"

2023,250,absolute_calibration_slope_error,lower,refit,static,50,0.15987317640617094,0.11911824257926934,0.1925704428998924,0.0,0.0,0.0,1.7763568394002505e-15,"algorithmic resampling stability, not population inference"

2023,250,absolute_calibration_slope_error,lower,intercept,logistic,50,-0.09250764072740744,-0.12260685315622838,-0.059669634895454994,0.86,0.0,67.0,3.8819791825517314e-10,"algorithmic resampling stability, not population inference"

2023,250,absolute_calibration_slope_error,lower,intercept,refit,50,-0.128062401290493,-0.1633091510109601,-0.0657441668926347,0.8,0.0,105.0,1.370373858833318e-08,"algorithmic resampling stability, not population inference"

2023,250,absolute_calibration_slope_error,lower,logistic,refit,50,-0.0017448873927322014,-0.09064962057507775,0.04524975606687451,0.52,0.0,545.0,0.3776732059941086,"algorithmic resampling stability, not population inference"

2023,250,brier,lower,intercept,static,50,0.0011899565037361975,0.0005575925746966887,0.0016089540042469944,0.08,0.0,36.0,8.842704346534447e-12,"algorithmic resampling stability, not population inference"

2023,250,brier,lower,logistic,static,50,0.0024823599864333967,0.0018982794862790475,0.002981630020960295,0.0,0.0,0.0,1.7763568394002505e-15,"algorithmic resampling stability, not population inference"

2023,250,brier,lower,refit,static,50,0.0060208139400532434,0.005593955894367349,0.006476217874092999,0.0,0.0,0.0,1.7763568394002505e-15,"algorithmic resampling stability, not population inference"

2023,250,brier,lower,intercept,logistic,50,-0.0009729775234541965,-0.0014180330171493505,-0.0006998659044408981,0.92,0.0,55.0,1.0206946399193839e-10,"algorithmic resampling stability, not population inference"

2023,250,brier,lower,intercept,refit,50,-0.005014743353942752,-0.0052694126884147985,-0.004456518545652793,1.0,0.0,0.0,1.7763568394002505e-15,"algorithmic resampling stability, not population inference"

2023,250,brier,lower,logistic,refit,50,-0.003641970411938797,-0.004515395915291903,-0.003131947101273999,0.92,0.0,13.0,1.5631940186722204e-13,"algorithmic resampling stability, not population inference"

2023,250,auroc,higher,intercept,static,50,-0.005617679558287181,-0.008861888568511667,-0.0037148680448739757,0.02,0.0,2.0,5.329070518200751e-15,"algorithmic resampling stability, not population inference"

2023,250,auroc,higher,logistic,static,50,-0.011837535977217262,-0.01476404584241131,-0.007687180758164658,0.0,0.0,0.0,1.7763568394002505e-15,"algorithmic resampling stability, not population inference"

2023,250,auroc,higher,refit,static,50,-0.0413011453222103,-0.049516609273113465,-0.036778918479626665,0.0,0.0,0.0,1.7763568394002505e-15,"algorithmic resampling stability, not population inference"

2023,250,auroc,higher,intercept,logistic,50,0.004205916590431569,0.003555395737157574,0.005644947814981427,0.88,0.0,88.0,3.0854181432005134e-09,"algorithmic resampling stability, not population inference"

2023,250,auroc,higher,intercept,refit,50,0.0340443930606571,0.031534560142955415,0.04003789739130059,1.0,0.0,0.0,1.7763568394002505e-15,"algorithmic resampling stability, not population inference"

2023,250,auroc,higher,logistic,refit,50,0.029137412015638797,0.026644170969794656,0.03442694958755166,1.0,0.0,0.0,1.7763568394002505e-15,"algorithmic resampling stability, not population inference"

2023,500,absolute_calibration_intercept_error,lower,intercept,static,50,-0.04379663441511206,-0.0742937891360099,-0.024044791113515707,0.78,0.0,267.0,0.0002211422351869885,"algorithmic resampling stability, not population inference"

2023,500,absolute_calibration_intercept_error,lower,logistic,static,50,-0.05069848875342285,-0.07668550123838123,-0.02818151922502181,0.72,0.0,273.0,0.0002857006608838475,"algorithmic resampling stability, not population inference"

2023,500,absolute_calibration_intercept_error,lower,refit,static,50,-0.0504395178186157,-0.08687215355806906,-0.008238557421390305,0.66,0.0,343.0,0.003913313547160513,"algorithmic resampling stability, not population inference"

2023,500,absolute_calibration_intercept_error,lower,intercept,logistic,50,0.0014109514394568118,-0.003622173032005599,0.007352387386626538,0.46,0.0,542.0,0.3622430237473182,"algorithmic resampling stability, not population inference"

2023,500,absolute_calibration_intercept_error,lower,intercept,refit,50,0.0026710113289739514,-0.022783228666533817,0.013881106859741948,0.48,0.0,592.0,0.6666519727454681,"algorithmic resampling stability, not population inference"

2023,500,absolute_calibration_intercept_error,lower,logistic,refit,50,-0.011465947078760558,-0.02080684394403719,0.013800444198707403,0.54,0.0,564.0,0.4843032319103049,"algorithmic resampling stability, not population inference"

2023,500,absolute_calibration_slope_error,lower,intercept,static,50,0.005629745828987001,0.0012747972477685004,0.0146037483374581,0.32,0.0,244.0,7.861200783665367e-05,"algorithmic resampling stability, not population inference"

2023,500,absolute_calibration_slope_error,lower,logistic,static,50,0.047293163369837146,0.030852093108455002,0.0738369566813324,0.1,0.0,34.0,6.616929226765933e-12,"algorithmic resampling stability, not population inference"

2023,500,absolute_calibration_slope_error,lower,refit,static,50,0.0899113534175646,0.0587309130346345,0.11104888482997011,0.08,0.0,11.0,9.769962616701378e-14,"algorithmic resampling stability, not population inference"

2023,500,absolute_calibration_slope_error,lower,intercept,logistic,50,-0.0416582706003233,-0.0598569786531786,-0.0209766061444443,0.86,0.0,86.0,2.5633326572460646e-09,"algorithmic resampling stability, not population inference"

2023,500,absolute_calibration_slope_error,lower,intercept,refit,50,-0.06277726612031478,-0.10621441984597346,-0.04136735583342045,0.8,0.0,97.0,6.917133532624575e-09,"algorithmic resampling stability, not population inference"

2023,500,absolute_calibration_slope_error,lower,logistic,refit,50,-0.03510596250946525,-0.0699084312021846,-0.006972962354153744,0.66,0.0,421.0,0.0362699414949752,"algorithmic resampling stability, not population inference"

2023,500,brier,lower,intercept,static,50,0.0005145482461942907,0.0002402347530379989,0.0006503204266682938,0.08,0.0,32.0,4.9116266609416925e-12,"algorithmic resampling stability, not population inference"

2023,500,brier,lower,logistic,static,50,0.0008520122129437452,0.0006203053540840484,0.0011390673531229967,0.04,0.0,4.0,1.2434497875801753e-14,"algorithmic resampling stability, not population inference"

2023,500,brier,lower,refit,static,50,0.0040621276499777925,0.0037366107466016013,0.004562115151302698,0.0,0.0,0.0,1.7763568394002505e-15,"algorithmic resampling stability, not population inference"

2023,500,brier,lower,intercept,logistic,50,-0.0004022774837564094,-0.0005059649297916025,-0.00033823141416375324,0.9,0.0,43.0,2.3080204414327454e-11,"algorithmic resampling stability, not population inference"

2023,500,brier,lower,intercept,refit,50,-0.0036053533679156965,-0.003847276463706098,-0.0030674438798955067,1.0,0.0,0.0,1.7763568394002505e-15,"algorithmic resampling stability, not population inference"

2023,500,brier,lower,logistic,refit,50,-0.0030497137797442023,-0.00340465725926499,-0.0026456011370976085,0.98,0.0,2.0,5.329070518200751e-15,"algorithmic resampling stability, not population inference"

2023,500,auroc,higher,intercept,static,50,-0.002361796334611921,-0.003502884015810115,-0.0018408707718169937,0.02,0.0,2.0,5.329070518200751e-15,"algorithmic resampling stability, not population inference"

2023,500,auroc,higher,logistic,static,50,-0.004266227310624537,-0.006308259829066909,-0.0029272101953601304,0.0,0.0,0.0,1.7763568394002505e-15,"algorithmic resampling stability, not population inference"

2023,500,auroc,higher,refit,static,50,-0.024588287802402575,-0.02749918242008287,-0.02316632435049737,0.0,0.0,0.0,1.7763568394002505e-15,"algorithmic resampling stability, not population inference"

2023,500,auroc,higher,intercept,logistic,50,0.0013332631580708076,0.0008457547957735345,0.0021223433924222324,0.84,0.0,115.0,3.094002387626915e-08,"algorithmic resampling stability, not population inference"

2023,500,auroc,higher,intercept,refit,50,0.021956394036593108,0.019799878337796784,0.023337351422680785,1.0,0.0,0.0,1.7763568394002505e-15,"algorithmic resampling stability, not population inference"

2023,500,auroc,higher,logistic,refit,50,0.020420224213411664,0.017368384572771456,0.02233928973087751,1.0,0.0,0.0,1.7763568394002505e-15,"algorithmic resampling stability, not population inference"

2023,1000,absolute_calibration_intercept_error,lower,intercept,static,50,-0.09293205331225035,-0.10312342724953574,-0.06410522153585546,0.86,0.0,58.0,1.4432899320127035e-10,"algorithmic resampling stability, not population inference"

2023,1000,absolute_calibration_intercept_error,lower,logistic,static,50,-0.08771597989490656,-0.09837539696463594,-0.0620631148836661,0.84,0.0,59.0,1.616786704516926e-10,"algorithmic resampling stability, not population inference"

2023,1000,absolute_calibration_intercept_error,lower,refit,static,50,-0.0860378588228783,-0.0979552276805597,-0.0638924276049241,0.88,0.0,89.0,3.3822118439275073e-09,"algorithmic resampling stability, not population inference"

2023,1000,absolute_calibration_intercept_error,lower,intercept,logistic,50,-0.0013820857871918524,-0.0037051947150087555,0.002390769851335201,0.54,0.0,538.0,0.3422827758111566,"algorithmic resampling stability, not population inference"

2023,1000,absolute_calibration_intercept_error,lower,intercept,refit,50,-0.012806919245476707,-0.027164393694287754,0.00562147462074415,0.58,0.0,517.0,0.24908489906916564,"algorithmic resampling stability, not population inference"

2023,1000,absolute_calibration_intercept_error,lower,logistic,refit,50,-0.011014531010997603,-0.018393112960572153,0.005213084926641243,0.58,0.0,517.0,0.24908489906916564,"algorithmic resampling stability, not population inference"

2023,1000,absolute_calibration_slope_error,lower,intercept,static,50,0.00263143872867125,-0.0006803884312384986,0.0072961317142714,0.38,0.0,350.0,0.004912360514200742,"algorithmic resampling stability, not population inference"

2023,1000,absolute_calibration_slope_error,lower,logistic,static,50,0.039383916436727305,0.023790178718320103,0.058014674794499746,0.06,0.0,12.0,1.2434497875801753e-13,"algorithmic resampling stability, not population inference"

2023,1000,absolute_calibration_slope_error,lower,refit,static,50,0.017280430671578653,0.0114551356206558,0.03571288422812496,0.16,0.0,58.0,1.4432899320127035e-10,"algorithmic resampling stability, not population inference"

2023,1000,absolute_calibration_slope_error,lower,intercept,logistic,50,-0.030551502035087053,-0.0465455678869967,-0.019297060223096803,0.88,0.0,40.0,1.5448975432263978e-11,"algorithmic resampling stability, not population inference"

2023,1000,absolute_calibration_slope_error,lower,intercept,refit,50,-0.019367481631649253,-0.023617540278908496,-0.0070242147667833,0.74,0.0,219.0,2.3068974657647345e-05,"algorithmic resampling stability, not population inference"

2023,1000,absolute_calibration_slope_error,lower,logistic,refit,50,0.017395997789496798,0.004011533982083204,0.0367464318727877,0.34,0.0,375.0,0.010566648384996213,"algorithmic resampling stability, not population inference"

2023,1000,brier,lower,intercept,static,50,0.00022960111528678995,8.060313008494135e-05,0.0003494795933585912,0.26,0.0,208.0,1.2965142769161275e-05,"algorithmic resampling stability, not population inference"

2023,1000,brier,lower,logistic,static,50,0.0005419735457600425,0.00035618160996299386,0.000738106482821993,0.12,0.0,40.0,1.5448975432263978e-11,"algorithmic resampling stability, not population inference"

2023,1000,brier,lower,refit,static,50,0.0024380956378966465,0.0022421759263447985,0.002848839689282595,0.0,0.0,0.0,1.7763568394002505e-15,"algorithmic resampling stability, not population inference"

2023,1000,brier,lower,intercept,logistic,50,-0.0002751196161965494,-0.0003403667479182984,-0.0001682186293785054,0.86,0.0,87.0,2.81308842886574e-09,"algorithmic resampling stability, not population inference"

2023,1000,brier,lower,intercept,refit,50,-0.0021868444239445534,-0.002611972065861605,-0.0019008107972607463,1.0,0.0,0.0,1.7763568394002505e-15,"algorithmic resampling stability, not population inference"

2023,1000,brier,lower,logistic,refit,50,-0.001900723611296791,-0.0022542732527091533,-0.001675464179147601,1.0,0.0,0.0,1.7763568394002505e-15,"algorithmic resampling stability, not population inference"

2023,1000,auroc,higher,intercept,static,50,-0.0017063662004668245,-0.0026023122818027433,-0.0012544938673160755,0.12,0.0,36.0,8.842704346534447e-12,"algorithmic resampling stability, not population inference"

2023,1000,auroc,higher,logistic,static,50,-0.0032509437459868895,-0.0038988942483326183,-0.0025655009952040286,0.02,0.0,1.0,3.552713678800501e-15,"algorithmic resampling stability, not population inference"

2023,1000,auroc,higher,refit,static,50,-0.015312204100212223,-0.016808937005345248,-0.014061356277495551,0.0,0.0,0.0,1.7763568394002505e-15,"algorithmic resampling stability, not population inference"

2023,1000,auroc,higher,intercept,logistic,50,0.0012881907165574469,0.0008203835097040924,0.00176067538055652,0.8,0.0,103.0,1.158324636207908e-08,"algorithmic resampling stability, not population inference"

2023,1000,auroc,higher,intercept,refit,50,0.012983694048848249,0.011253719360852787,0.014488530191165083,1.0,0.0,0.0,1.7763568394002505e-15,"algorithmic resampling stability, not population inference"

2023,1000,auroc,higher,logistic,refit,50,0.012195519597386573,0.010126783789696114,0.013765711216009557,1.0,0.0,0.0,1.7763568394002505e-15,"algorithmic resampling stability, not population inference"

2023,2000,absolute_calibration_intercept_error,lower,intercept,static,50,-0.10740910576087226,-0.119228321118096,-0.09618225671834005,0.94,0.0,9.0,5.861977570020827e-14,"algorithmic resampling stability, not population inference"

2023,2000,absolute_calibration_intercept_error,lower,logistic,static,50,-0.1079433830468868,-0.11892919039082188,-0.0966682839636599,0.94,0.0,9.0,5.861977570020827e-14,"algorithmic resampling stability, not population inference"

2023,2000,absolute_calibration_intercept_error,lower,refit,static,50,-0.1093759311144643,-0.11690489705866611,-0.0902117678582971,0.92,0.0,20.0,6.590283874174929e-13,"algorithmic resampling stability, not population inference"

2023,2000,absolute_calibration_intercept_error,lower,intercept,logistic,50,-0.0008591299724837534,-0.002088757379336801,0.0007337251788349444,0.6,0.0,530.0,0.3044806608698192,"algorithmic resampling stability, not population inference"

2023,2000,absolute_calibration_intercept_error,lower,intercept,refit,50,-0.004165381890739953,-0.0145079356322951,0.005579951648173849,0.6,0.0,505.0,0.20447619481721802,"algorithmic resampling stability, not population inference"

2023,2000,absolute_calibration_intercept_error,lower,logistic,refit,50,-0.002267189820104147,-0.012985517985159246,0.005632805706443399,0.56,0.0,529.0,0.2999546071877397,"algorithmic resampling stability, not population inference"

2023,2000,absolute_calibration_slope_error,lower,intercept,static,50,-0.0036036848396159495,-0.004183285838536399,-0.0024822655232258997,0.86,0.0,220.0,2.4281497463363166e-05,"algorithmic resampling stability, not population inference"

2023,2000,absolute_calibration_slope_error,lower,logistic,static,50,0.02602195684872095,0.01175257088273425,0.04050029019845875,0.2,0.0,82.0,1.7568968502246207e-09,"algorithmic resampling stability, not population inference"

2023,2000,absolute_calibration_slope_error,lower,refit,static,50,0.0319612101680869,0.024401557977179203,0.039866097697960595,0.08,0.0,25.0,1.6058265828178264e-12,"algorithmic resampling stability, not population inference"

2023,2000,absolute_calibration_slope_error,lower,intercept,logistic,50,-0.028026371660391354,-0.043555567370649896,-0.013356617544209502,0.88,0.0,41.0,1.7687185049908294e-11,"algorithmic resampling stability, not population inference"

2023,2000,absolute_calibration_slope_error,lower,intercept,refit,50,-0.0363630799278487,-0.043838673066909445,-0.025180417829955504,0.94,0.0,10.0,7.638334409421077e-14,"algorithmic resampling stability, not population inference"

2023,2000,absolute_calibration_slope_error,lower,logistic,refit,50,-3.094960598494989e-05,-0.010489251592162303,0.013214977971869095,0.5,0.0,629.0,0.9389922173111831,"algorithmic resampling stability, not population inference"

2023,2000,brier,lower,intercept,static,50,-3.25759131332079e-05,-0.00010284373414860826,2.4670974680195135e-05,0.56,0.0,569.0,0.5148085516941183,"algorithmic resampling stability, not population inference"

2023,2000,brier,lower,logistic,static,50,6.770803156644356e-05,2.2402381330499654e-05,0.00017972813948019528,0.3,0.0,304.0,0.0009871220775430345,"algorithmic resampling stability, not population inference"

2023,2000,brier,lower,refit,static,50,0.0013115272216795912,0.0011573953567391992,0.001534801621240492,0.02,0.0,3.0,8.881784197001252e-15,"algorithmic resampling stability, not population inference"

2023,2000,brier,lower,intercept,logistic,50,-0.00014681976619085324,-0.00022718768841330228,-3.6011239622504854e-05,0.76,0.0,178.0,2.36936675790389e-06,"algorithmic resampling stability, not population inference"

2023,2000,brier,lower,intercept,refit,50,-0.001308326593191446,-0.0015625514402949556,-0.0010343566023812978,0.98,0.0,1.0,3.552713678800501e-15,"algorithmic resampling stability, not population inference"

2023,2000,brier,lower,logistic,refit,50,-0.0010458027985821503,-0.0012947789085157521,-0.0009821309980587029,0.92,0.0,14.0,1.9539925233402755e-13,"algorithmic resampling stability, not population inference"

2023,2000,auroc,higher,intercept,static,50,-0.0008994529120686656,-0.001212717126462426,-0.0005599486762347672,0.16,0.0,72.0,6.535714192068554e-10,"algorithmic resampling stability, not population inference"

2023,2000,auroc,higher,logistic,static,50,-0.0017518915896440612,-0.0020900004953408047,-0.0010126469751200462,0.06,0.0,9.0,5.861977570020827e-14,"algorithmic resampling stability, not population inference"

2023,2000,auroc,higher,refit,static,50,-0.006604434934542158,-0.007966555301150202,-0.0047340959422824125,0.02,0.0,1.0,3.552713678800501e-15,"algorithmic resampling stability, not population inference"

2023,2000,auroc,higher,intercept,logistic,50,0.0006275517733158598,0.00037462812903543696,0.0008088685965035869,0.86,0.0,63.0,2.5226754019058717e-10,"algorithmic resampling stability, not population inference"

2023,2000,auroc,higher,intercept,refit,50,0.005330886240338006,0.003609718436204501,0.006511352286427852,0.92,0.0,10.0,7.638334409421077e-14,"algorithmic resampling stability, not population inference"

2023,2000,auroc,higher,logistic,refit,50,0.004792939737682189,0.003086337853787424,0.005695538047151727,0.9,0.0,42.0,2.022026990289305e-11,"algorithmic resampling stability, not population inference"

2023,4000,absolute_calibration_intercept_error,lower,intercept,static,50,-0.112517458216613,-0.1192953095214794,-0.105891993641729,1.0,0.0,0.0,1.7763568394002505e-15,"algorithmic resampling stability, not population inference"

2023,4000,absolute_calibration_intercept_error,lower,logistic,static,50,-0.11559310090282204,-0.1204088438057612,-0.1067263364843926,1.0,0.0,0.0,1.7763568394002505e-15,"algorithmic resampling stability, not population inference"

2023,4000,absolute_calibration_intercept_error,lower,refit,static,50,-0.11091841400500779,-0.1275034539573412,-0.1025842420077123,1.0,0.0,0.0,1.7763568394002505e-15,"algorithmic resampling stability, not population inference"

2023,4000,absolute_calibration_intercept_error,lower,intercept,logistic,50,0.0008087677386951018,-0.0013705685063967517,0.0016571159128323035,0.42,0.0,579.0,0.5786292137562761,"algorithmic resampling stability, not population inference"

2023,4000,absolute_calibration_intercept_error,lower,intercept,refit,50,-0.0005526962636805011,-0.009445078172071297,0.0070626126851926,0.5,0.0,612.0,0.8109402812359416,"algorithmic resampling stability, not population inference"

2023,4000,absolute_calibration_intercept_error,lower,logistic,refit,50,0.0003374890238635993,-0.0083740451387187,0.007082296988466999,0.5,0.0,603.0,0.7448958960780061,"algorithmic resampling stability, not population inference"

2023,4000,absolute_calibration_slope_error,lower,intercept,static,50,-0.00416187674800895,-0.0058587168198005,-0.003040606337496499,0.84,0.0,65.0,3.134559278805682e-10,"algorithmic resampling stability, not population inference"

2023,4000,absolute_calibration_slope_error,lower,logistic,static,50,0.01381211062562405,0.006659273170025001,0.0193087204551634,0.16,0.0,86.0,2.5633326572460646e-09,"algorithmic resampling stability, not population inference"

2023,4000,absolute_calibration_slope_error,lower,refit,static,50,0.021927719464732348,0.011664171516608101,0.02951538495759725,0.08,0.0,14.0,1.9539925233402755e-13,"algorithmic resampling stability, not population inference"

2023,4000,absolute_calibration_slope_error,lower,intercept,logistic,50,-0.018925204850216402,-0.02436487631854145,-0.01099466999604,0.9,0.0,32.0,4.9116266609416925e-12,"algorithmic resampling stability, not population inference"

2023,4000,absolute_calibration_slope_error,lower,intercept,refit,50,-0.02454271593828055,-0.03211797716910755,-0.0157895503541265,0.98,0.0,2.0,5.329070518200751e-15,"algorithmic resampling stability, not population inference"

2023,4000,absolute_calibration_slope_error,lower,logistic,refit,50,-0.0058938505684601,-0.012761192632381402,-0.0019339265703026474,0.7,0.0,321.0,0.0018425334424101436,"algorithmic resampling stability, not population inference"

2023,4000,brier,lower,intercept,static,50,-7.613974689185676e-05,-0.0001305183381106051,-1.735461128499205e-06,0.64,0.0,304.0,0.0009871220775430345,"algorithmic resampling stability, not population inference"

2023,4000,brier,lower,logistic,static,50,1.7746291348590126e-05,-3.7878031940608015e-05,0.00010781287448780041,0.42,0.0,468.0,0.10316599771973678,"algorithmic resampling stability, not population inference"

2023,4000,brier,lower,refit,static,50,0.0004162535595785971,0.0002508472049205443,0.0005924448364973967,0.18,0.0,118.0,3.918623114884667e-08,"algorithmic resampling stability, not population inference"

2023,4000,brier,lower,intercept,logistic,50,-9.777163427629465e-05,-0.0001531589177795889,-5.998036567349957e-05,0.86,0.0,83.0,1.9326371614170057e-09,"algorithmic resampling stability, not population inference"

2023,4000,brier,lower,intercept,refit,50,-0.00047952132625899563,-0.0006057270960008049,-0.0002776470408206999,0.9,0.0,74.0,8.008242957657785e-10,"algorithmic resampling stability, not population inference"

2023,4000,brier,lower,logistic,refit,50,-0.0003778969522311021,-0.0004940190392464516,-0.00015970654088490188,0.84,0.0,167.0,1.206143563692308e-06,"algorithmic resampling stability, not population inference"

2023,4000,auroc,higher,intercept,static,50,-0.0007498489899458449,-0.0009822393797537243,-0.0004957144136957803,0.12,0.0,47.0,3.863753761379485e-11,"algorithmic resampling stability, not population inference"

2023,4000,auroc,higher,logistic,static,50,-0.0012020548904572714,-0.0015785509850768098,-0.0009073528983524204,0.02,0.0,13.0,1.5631940186722204e-13,"algorithmic resampling stability, not population inference"

2023,4000,auroc,higher,refit,static,50,-0.0016327297699675336,-0.002391100969615201,-0.001031371125985614,0.16,0.0,224.0,2.9747345218211763e-05,"algorithmic resampling stability, not population inference"

2023,4000,auroc,higher,intercept,logistic,50,0.00035982956896463136,0.00025042576644057624,0.0005696270646529111,0.84,0.0,122.0,5.3406212785489515e-08,"algorithmic resampling stability, not population inference"

2023,4000,auroc,higher,intercept,refit,50,0.0008862229349338446,0.0004934569649265863,0.0018533416489645571,0.7,0.0,348.0,0.004606102327862516,"algorithmic resampling stability, not population inference"

2023,4000,auroc,higher,logistic,refit,50,0.00044311841451433187,5.459668821583552e-05,0.001077968681488295,0.66,0.0,458.0,0.08401577354329604,"algorithmic resampling stability, not population inference"
