# Supplementary material for: Temporal maintenance of an adolescent suicide attempt screening score in national surveys: An observational study
Source: Medicine (Baltimore). 2026 Aug 28;105(35):e50513. doi: 10.1097/MD.0000000000050513 (PMC13529099; doi:10.1097/MD.0000000000050513)
Supplement: Supplementary file 11 [file medi-105-e50513-s011.docx]

**Supplemental Digital Content 16**

**Drift decomposition data**

Temporal maintenance of an adolescent suicide attempt screening score in national surveys: An observational study

First author: Xiaotian Ma

*Source file: drift_decomposition.csv*

This Word document contains aggregate or metadata records only and does not include individual student records.

source_year,target_year,source_observed_prevalence,source_model_mean_in_source,target_observed_prevalence,source_model_mean_in_target,target_model_mean_in_target,total_prevalence_shift_risk_difference,covariate_shift_component_risk_difference,coefficient_shift_component_risk_difference,model_residual_component_risk_difference,risk_difference_reconstruction_error,total_prevalence_shift_logodds,covariate_shift_component_logodds,coefficient_shift_component_logodds,model_residual_component_logodds,logodds_reconstruction_error,covariate_share_of_total_risk_difference,coefficient_share_of_total_risk_difference

2015,2017,0.08564627356459587,0.08565474476208892,0.07371471546889397,0.08931337414364483,0.07377642092215678,-0.0119315580957019,0.0036586293815559084,-0.015536953221488056,-5.3234255769751826e-05,0.0,-0.16298803373045967,0.045836014960601634,-0.20802886494878692,-0.0007951837422743857,0.0,-0.3066346701923075,1.3021730353125403

2015,2019,0.08564627356459587,0.08565474476208892,0.08905635008953747,0.0966849965385677,0.08914101991569577,0.003410076524941602,0.011030251776478775,-0.0075439766228719285,-7.61986286652444e-05,0.0,0.04278006600962847,0.13327052281997886,-0.08955538133489815,-0.0009350754754522406,0.0,3.2346053514642663,-2.2122602140141474

2015,2021,0.08564627356459587,0.08565474476208892,0.10170608668544727,0.09959617982234521,0.10174144225756976,0.016059813120851404,0.013941435060256288,0.002145262435224546,-2.6884374629429497e-05,0.0,0.18958162794972067,0.16616407762510077,0.023696305255116012,-0.00027875493049611677,0.0,0.8680944762772674,0.13357953913169918

2015,2023,0.08564627356459587,0.08565474476208892,0.09462945164439508,0.10485537475204987,0.09472224923764075,0.008983178079799217,0.019200629989960943,-0.01013312551440912,-8.432639575260692e-05,0.0,0.10961623987477553,0.22348034045056497,-0.11288960620854294,-0.0009744943672465034,0.0,2.1373983482680883,-1.1280112032061158
