# Supplementary material for: Temporal maintenance of an adolescent suicide attempt screening score in national surveys: An observational study
Source: Medicine (Baltimore). 2026 Aug 28;105(35):e50513. doi: 10.1097/MD.0000000000050513 (PMC13529099; doi:10.1097/MD.0000000000050513)
Supplement: Supplementary file 12 [file medi-105-e50513-s012.docx]

**Supplemental Digital Content 3**

**Analysis utilities**

Temporal maintenance of an adolescent suicide attempt screening score in national surveys: An observational study

First author: Xiaotian Ma

*Source file: analysis_utils.py*

This Word document contains the complete source code for the identified analysis step.

```
from __future__ import annotations

from dataclasses import dataclass
from typing import Iterable, Literal

import numpy as np
import pandas as pd
import statsmodels.api as sm
from scipy.special import expit, logit
from sklearn.compose import ColumnTransformer
from sklearn.metrics import log_loss
from sklearn.model_selection import GroupKFold
from sklearn.pipeline import Pipeline
from sklearn.preprocessing import OneHotEncoder
from sklearn.linear_model import LogisticRegression
try:
    from xgboost import XGBClassifier
except ImportError:  # The Medicine revision uses only logistic models.
    XGBClassifier = None


PREDICTORS = [
    "sex",
    "grade",
    "race_ethnicity",
    "sexual_identity_primary",
    "safety_absence",
    "weapon_threat_school",
    "physical_fight_school",
    "forced_sex",
    "bullying_school",
    "electronic_bullying",
    "sad_hopeless",
    "current_cigarette",
    "current_vaping",
    "current_alcohol",
    "current_marijuana",
    "no_physical_activity",
    "short_sleep",
]

AUXILIARIES = ["ideation_aux", "plan_aux", "injury_aux"]
# Survey stratum is used only in nuisance models for outcome observability. It is
# deliberately excluded from the deployable psychological risk score.
RESPONSE_FEATURES = PREDICTORS + AUXILIARIES + ["stratum"]


def design_groups(frame: pd.DataFrame) -> pd.Series:
    return frame["stratum"].astype(str) + "_" + frame["psu"].astype(str)


def normalized_weights(weights: Iterable[float]) -> np.ndarray:
    w = np.asarray(weights, dtype=float)
    return w * (len(w) / w.sum())


def weighted_quantile(values: Iterable[float], quantile: float, weights: Iterable[float]) -> float:
    values = np.asarray(values, dtype=float)
    weights = np.asarray(weights, dtype=float)
    order = np.argsort(values)
    values = values[order]
    weights = weights[order]
    cumulative = np.cumsum(weights) - 0.5 * weights
    cumulative /= weights.sum()
    return float(np.interp(quantile, cumulative, values))


def weighted_sensitivity_threshold(
    outcome: Iterable[float], score: Iterable[float], weights: Iterable[float], target: float = 0.80
) -> float:
    """Return the highest-risk cutoff reaching a target weighted sensitivity."""
    y = np.asarray(outcome, dtype=float)
    p = np.asarray(score, dtype=float)
    w = np.asarray(weights, dtype=float)
    order = np.argsort(-p)
    y, p, w = y[order], p[order], w[order]
    positive = w * y
    if positive.sum() <= 0:
        return np.nan
    crossing = int(np.searchsorted(np.cumsum(positive) / positive.sum(), target, side="left"))
    return float(p[min(crossing, len(p) - 1)])


def effective_sample_size(weights: Iterable[float]) -> float:
    w = np.asarray(weights, dtype=float)
    return float(w.sum() ** 2 / np.square(w).sum())


def fractional_auc(y_probability: Iterable[float], score: Iterable[float], weights: Iterable[float]) -> float:
    q = np.asarray(y_probability, dtype=float)
    p = np.asarray(score, dtype=float)
    w = np.asarray(weights, dtype=float)
    pos = w * q
    neg = w * (1 - q)
    total_pos = pos.sum()
    total_neg = neg.sum()
    if total_pos <= 0 or total_neg <= 0:
        return np.nan
    order = np.argsort(p)
    p, pos, neg = p[order], pos[order], neg[order]
    starts = np.r_[0, np.flatnonzero(np.diff(p)) + 1]
    pos_group = np.add.reduceat(pos, starts)
    neg_group = np.add.reduceat(neg, starts)
    cumulative_neg_before = np.cumsum(neg_group) - neg_group
    numerator = np.sum(pos_group * (cumulative_neg_before + 0.5 * neg_group))
    return float(numerator / (total_pos * total_neg))


def fractional_average_precision(
    y_probability: Iterable[float], score: Iterable[float], weights: Iterable[float]
) -> float:
    q = np.asarray(y_probability, dtype=float)
    p = np.asarray(score, dtype=float)
    w = np.asarray(weights, dtype=float)
    pos = w * q
    neg = w * (1 - q)
    total_pos = pos.sum()
    if total_pos <= 0:
        return np.nan
    order = np.argsort(-p)
    p, pos, neg = p[order], pos[order], neg[order]
    starts = np.r_[0, np.flatnonzero(np.diff(p)) + 1]
    pos_group = np.add.reduceat(pos, starts)
    total_group = pos_group + np.add.reduceat(neg, starts)
    cumulative_pos = np.cumsum(pos_group)
    cumulative_total = np.cumsum(total_group)
    precision = np.divide(
        cumulative_pos,
        cumulative_total,
        out=np.zeros_like(cumulative_pos, dtype=float),
        where=cumulative_total > 0,
    )
    average_precision = np.sum(precision * (pos_group / total_pos))
    return float(average_precision)


def calibration_coefficients(
    y_probability: Iterable[float], score: Iterable[float], weights: Iterable[float]
) -> tuple[float, float]:
    q = np.asarray(y_probability, dtype=float)
    p = np.clip(np.asarray(score, dtype=float), 1e-6, 1 - 1e-6)
    w = normalized_weights(weights)
    lp = logit(p)
    try:
        alpha = 0.0
        for _ in range(100):
            mean = expit(lp + alpha)
            information = np.sum(w * mean * (1 - mean))
            step = np.sum(w * (q - mean)) / information
            alpha += step
            if abs(step) < 1e-10:
                break

        design = np.column_stack([np.ones(len(lp)), lp])
        coefficient = np.array([0.0, 1.0])
        for _ in range(100):
            mean = expit(design @ coefficient)
            working_weight = w * mean * (1 - mean)
            information = design.T @ (working_weight[:, None] * design)
            score_vector = design.T @ (w * (q - mean))
            step = np.linalg.solve(information, score_vector)
            coefficient += step
            if np.max(np.abs(step)) < 1e-10:
                break
        return float(alpha), float(coefficient[1])
    except Exception:
        return np.nan, np.nan


def performance_metrics(
    y_probability: Iterable[float],
    score: Iterable[float],
    weights: Iterable[float],
    threshold: float,
) -> dict[str, float]:
    q = np.asarray(y_probability, dtype=float)
    p = np.clip(np.asarray(score, dtype=float), 1e-8, 1 - 1e-8)
    w = np.asarray(weights, dtype=float)
    prevalence = np.average(q, weights=w)
    brier = np.average(q * np.square(1 - p) + (1 - q) * np.square(p), weights=w)
    null_brier = prevalence * (1 - prevalence)
    calibration_intercept, calibration_slope = calibration_coefficients(q, p, w)
    selected = p >= threshold
    positive_weight = np.sum(w * q)
    predicted_positive_weight = np.sum(w * selected)
    true_positive_weight = np.sum(w * q * selected)
    sensitivity = true_positive_weight / positive_weight if positive_weight > 0 else np.nan
    ppv = true_positive_weight / predicted_positive_weight if predicted_positive_weight > 0 else np.nan
    return {
        "n": float(len(q)),
        "effective_n": effective_sample_size(w),
        "expected_events": float(positive_weight),
        "prevalence": float(prevalence),
        "auroc": fractional_auc(q, p, w),
        "auprc": fractional_average_precision(q, p, w),
        "brier": float(brier),
        "scaled_brier": float(1 - brier / null_brier) if null_brier > 0 else np.nan,
        "calibration_intercept": calibration_intercept,
        "calibration_slope": calibration_slope,
        "observed_expected_ratio": float(np.sum(w * q) / np.sum(w * p)),
        "sensitivity": float(sensitivity),
        "false_negative_rate": float(1 - sensitivity),
        "positive_predictive_value": float(ppv),
        "referral_fraction": float(np.average(selected, weights=w)),
    }


def calibration_curve_fractional(
    y_probability: Iterable[float],
    score: Iterable[float],
    weights: Iterable[float],
    bins: int = 10,
) -> pd.DataFrame:
    q = np.asarray(y_probability, dtype=float)
    p = np.asarray(score, dtype=float)
    w = np.asarray(weights, dtype=float)
    cuts = np.unique([weighted_quantile(p, x, w) for x in np.linspace(0, 1, bins + 1)])
    if len(cuts) < 3:
        return pd.DataFrame()
    group = np.clip(np.digitize(p, cuts[1:-1], right=True), 0, len(cuts) - 2)
    rows = []
    for b in np.unique(group):
        mask = group == b
        rows.append(
            {
                "bin": int(b) + 1,
                "n": int(mask.sum()),
                "weight": float(w[mask].sum()),
                "mean_predicted": float(np.average(p[mask], weights=w[mask])),
                "mean_outcome": float(np.average(q[mask], weights=w[mask])),
                "min_predicted": float(p[mask].min()),
                "max_predicted": float(p[mask].max()),
            }
        )
    return pd.DataFrame(rows)


def categorical_preprocessor(columns: list[str]) -> ColumnTransformer:
    return ColumnTransformer(
        [("categorical", OneHotEncoder(handle_unknown="ignore", sparse_output=True), columns)],
        remainder="drop",
    )


def logistic_pipeline(columns: list[str], c_value: float = 1.0) -> Pipeline:
    return Pipeline(
        [
            ("preprocess", categorical_preprocessor(columns)),
            (
                "model",
                LogisticRegression(
                    C=c_value,
                    l1_ratio=0.0,
                    solver="lbfgs",
                    max_iter=3000,
                    random_state=20260713,
                ),
            ),
        ]
    )


def xgboost_pipeline(columns: list[str], params: dict[str, object]) -> Pipeline:
    if XGBClassifier is None:
        raise ImportError("xgboost is required only for the optional XGBoost pipeline.")
    defaults: dict[str, object] = {
        "objective": "binary:logistic",
        "eval_metric": "logloss",
        "tree_method": "hist",
        "n_jobs": 4,
        "random_state": 20260713,
        "subsample": 0.85,
        "colsample_bytree": 0.85,
        "reg_lambda": 5.0,
        "min_child_weight": 10.0,
        "verbosity": 0,
    }
    defaults.update(params)
    return Pipeline(
        [
            ("preprocess", categorical_preprocessor(columns)),
            ("model", XGBClassifier(**defaults)),
        ]
    )


@dataclass
class TunedModel:
    pipeline: Pipeline
    parameters: dict[str, object]
    cross_validated_log_loss: float


def tune_logistic(
    frame: pd.DataFrame,
    columns: list[str],
    outcome: np.ndarray,
    weights: np.ndarray,
    groups: np.ndarray,
    candidates: tuple[float, ...] = (0.01, 0.1, 1.0, 10.0),
) -> TunedModel:
    cv = GroupKFold(n_splits=5)
    rows = []
    for c_value in candidates:
        losses = []
        fold_weights = []
        for train, test in cv.split(frame, outcome, groups):
            model = logistic_pipeline(columns, c_value)
            model.fit(frame.iloc[train][columns], outcome[train], model__sample_weight=weights[train])
            prediction = model.predict_proba(frame.iloc[test][columns])[:, 1]
            losses.append(log_loss(outcome[test], prediction, sample_weight=weights[test], labels=[0, 1]))
            fold_weights.append(weights[test].sum())
        rows.append((float(np.average(losses, weights=fold_weights)), c_value))
    best_loss, best_c = min(rows)
    fitted = logistic_pipeline(columns, best_c)
    fitted.fit(frame[columns], outcome, model__sample_weight=weights)
    return TunedModel(fitted, {"C": best_c}, best_loss)


def tune_xgboost(
    frame: pd.DataFrame,
    columns: list[str],
    outcome: np.ndarray,
    weights: np.ndarray,
    groups: np.ndarray,
) -> TunedModel:
    candidates = [
        {"n_estimators": 250, "max_depth": 2, "learning_rate": 0.04},
        {"n_estimators": 400, "max_depth": 2, "learning_rate": 0.03},
        {"n_estimators": 250, "max_depth": 3, "learning_rate": 0.04},
        {"n_estimators": 400, "max_depth": 3, "learning_rate": 0.03},
    ]
    cv = GroupKFold(n_splits=5)
    rows = []
    for params in candidates:
        losses = []
        fold_weights = []
        for train, test in cv.split(frame, outcome, groups):
            model = xgboost_pipeline(columns, params)
            model.fit(frame.iloc[train][columns], outcome[train], model__sample_weight=weights[train])
            prediction = model.predict_proba(frame.iloc[test][columns])[:, 1]
            losses.append(log_loss(outcome[test], prediction, sample_weight=weights[test], labels=[0, 1]))
            fold_weights.append(weights[test].sum())
        rows.append((float(np.average(losses, weights=fold_weights)), params))
    best_loss, best_params = min(rows, key=lambda item: item[0])
    fitted = xgboost_pipeline(columns, best_params)
    fitted.fit(frame[columns], outcome, model__sample_weight=weights)
    return TunedModel(fitted, best_params, best_loss)


def grouped_oof_predictions(
    frame: pd.DataFrame,
    columns: list[str],
    observed_mask: np.ndarray,
    outcome: np.ndarray,
    weights: np.ndarray,
    groups: np.ndarray,
    model_kind: Literal["logistic", "xgboost"],
    parameters: dict[str, object],
    n_splits: int = 5,
) -> np.ndarray:
    """Predict every record out of fold while fitting only records with observed outcomes."""
    prediction = np.full(len(frame), np.nan, dtype=float)
    splitter = GroupKFold(n_splits=n_splits)
    placeholder = np.zeros(len(frame), dtype=int)
    for train, test in splitter.split(frame, placeholder, groups):
        fit_rows = train[observed_mask[train]]
        if model_kind == "logistic":
            model = logistic_pipeline(columns, float(parameters["C"]))
        else:
            model = xgboost_pipeline(columns, parameters)
        model.fit(
            frame.iloc[fit_rows][columns],
            outcome[fit_rows].astype(int),
            model__sample_weight=weights[fit_rows],
        )
        prediction[test] = model.predict_proba(frame.iloc[test][columns])[:, 1]
    if np.isnan(prediction).any():
        raise RuntimeError("Grouped out-of-fold prediction left unscored records.")
    return prediction


def cross_fitted_observation_propensity(frame: pd.DataFrame) -> tuple[np.ndarray, dict[str, float]]:
    outcome = frame["attempt_observed"].to_numpy(dtype=int)
    weights = frame["weight"].to_numpy(dtype=float)
    groups = design_groups(frame).to_numpy()
    predictions = np.zeros(len(frame), dtype=float)
    cv = GroupKFold(n_splits=5)
    for train, test in cv.split(frame, outcome, groups):
        model = logistic_pipeline(RESPONSE_FEATURES, c_value=0.1)
        model.fit(
            frame.iloc[train][RESPONSE_FEATURES],
            outcome[train],
            model__sample_weight=weights[train],
        )
        predictions[test] = model.predict_proba(frame.iloc[test][RESPONSE_FEATURES])[:, 1]
    lower = max(0.02, float(np.quantile(predictions, 0.01)))
    upper = min(0.995, float(np.quantile(predictions, 0.99)))
    clipped = np.clip(predictions, lower, upper)
    observed_weights = weights[outcome == 1] / clipped[outcome == 1]
    diagnostics = {
        "propensity_min": float(predictions.min()),
        "propensity_p01": float(np.quantile(predictions, 0.01)),
        "propensity_median": float(np.median(predictions)),
        "propensity_p99": float(np.quantile(predictions, 0.99)),
        "propensity_max": float(predictions.max()),
        "clip_lower": lower,
        "clip_upper": upper,
        "ipw_effective_n": effective_sample_size(observed_weights),
        "ipw_max": float((1 / clipped[outcome == 1]).max()),
    }
    return clipped, diagnostics


def missing_outcome_probabilities(frame: pd.DataFrame) -> tuple[np.ndarray, dict[str, object]]:
    observed_mask = frame["attempt_observed"].to_numpy(dtype=bool)
    observed = frame.loc[observed_mask].copy()
    y = observed["attempt"].to_numpy(dtype=int)
    w = observed["weight"].to_numpy(dtype=float)
    groups = design_groups(observed).to_numpy()
    tuned = tune_logistic(observed, RESPONSE_FEATURES, y, w, groups, candidates=(0.01, 0.1, 1.0))
    probability = np.empty(len(frame), dtype=float)
    probability[observed_mask] = frame.loc[observed_mask, "attempt"].to_numpy(dtype=float)
    probability[~observed_mask] = tuned.pipeline.predict_proba(
        frame.loc[~observed_mask, RESPONSE_FEATURES]
    )[:, 1]
    diagnostics: dict[str, object] = {
        "imputation_C": tuned.parameters["C"],
        "imputation_cv_log_loss": tuned.cross_validated_log_loss,
        "missing_predicted_attempt_mean_weighted": float(
            np.average(probability[~observed_mask], weights=frame.loc[~observed_mask, "weight"])
        ),
        "missing_predicted_attempt_min": float(probability[~observed_mask].min()),
        "missing_predicted_attempt_max": float(probability[~observed_mask].max()),
    }
    return probability, diagnostics


def apply_delta_to_missing(
    base_probability: np.ndarray,
    observed_mask: np.ndarray,
    odds_multiplier: float,
) -> np.ndarray:
    result = base_probability.copy()
    missing = ~observed_mask
    result[missing] = expit(logit(np.clip(result[missing], 1e-6, 1 - 1e-6)) + np.log(odds_multiplier))
    return result
```
