# Supplementary material for: Temporal maintenance of an adolescent suicide attempt screening score in national surveys: An observational study
Source: Medicine (Baltimore). 2026 Aug 28;105(35):e50513. doi: 10.1097/MD.0000000000050513 (PMC13529099; doi:10.1097/MD.0000000000050513)
Supplement: Supplementary file 13 [file medi-105-e50513-s013.docx]

**Supplemental Digital Content 6**

**Revision analysis code**

Temporal maintenance of an adolescent suicide attempt screening score in national surveys: An observational study

First author: Xiaotian Ma

*Source file: run_revision_analyses.py*

This Word document contains the complete source code for the identified analysis step.

```
"""Analyses requested in the Medicine major revision.

The script adds four bounded sensitivity analyses without changing the frozen
primary analysis: ridge penalty sensitivity, stronger regularization for small
target samples, an Oaxaca style drift decomposition, variance sensitivity for
single PSU strata, and paired nonparametric summaries of the original repeated
updating experiment.
"""

from __future__ import annotations

import json
import time
from pathlib import Path

import joblib
import numpy as np
import pandas as pd
from scipy.special import logit
from scipy.stats import wilcoxon
from sklearn.model_selection import StratifiedGroupKFold

from analysis_utils import (
    logistic_pipeline,
    normalized_weights,
    weighted_quantile,
)
from run_sci_maintenance_learning_curves import (
    N_SPLITS,
    SCI_PREDICTORS,
    SEED,
    design_groups,
    maintenance_metrics,
    proportional_stratum_sample,
)


ROOT = Path(__file__).resolve().parents[1]
DATA_PATH = ROOT / "derived_data" / "yrbs_2015_2023_harmonized.csv"
PRIMARY_RESULTS = ROOT / "results" / "sci_maintenance"
OUT = ROOT / "results" / "revision"
PENALTY_GRID = [0.01, 0.1, 1.0, 10.0]
PENALTY_BUDGETS = [250, 1000]
ORIGINAL_BUDGET_INDEX = {250: 0, 1000: 2}
TARGET_YEARS = [2017, 2019, 2021, 2023]
REPEATS = 50
BOOTSTRAP_REPLICATES = 500


def report(message: str) -> None:
    print(f"[{time.strftime('%H:%M:%S')}] {message}", flush=True)


def load_analysis_data() -> tuple[pd.DataFrame, object, float]:
    data = pd.read_csv(DATA_PATH, low_memory=False)
    observed = data[data["attempt_observed"].eq(1)].copy()
    if len(observed) != 68682 or int(observed["attempt"].sum()) != 6855:
        raise RuntimeError("The observed outcome population does not match the frozen ledger.")
    model = joblib.load(PRIMARY_RESULTS / "frozen_source_model.joblib")
    metadata = json.loads((PRIMARY_RESULTS / "run_metadata.json").read_text(encoding="utf-8"))
    threshold = float(metadata["frozen_threshold"])
    return observed, model, threshold


def add_frozen_scores(observed: pd.DataFrame, model) -> pd.DataFrame:
    scored = observed.copy()
    source_score = pd.read_parquet(PRIMARY_RESULTS / "source_oof_predictions.parquet")[[
        "record_id",
        "frozen_score",
    ]]
    scored = scored.merge(source_score, on="record_id", how="left", validate="one_to_one")
    for year in TARGET_YEARS:
        mask = scored["year"].eq(year)
        scored.loc[mask, "frozen_score"] = model.predict_proba(
            scored.loc[mask, SCI_PREDICTORS]
        )[:, 1]
    if scored["frozen_score"].isna().any():
        raise RuntimeError("Frozen scores are incomplete.")
    return scored


def source_penalty_sensitivity(observed: pd.DataFrame) -> pd.DataFrame:
    source = observed[observed["year"].eq(2015)].reset_index(drop=True)
    y_source = source["attempt"].to_numpy(dtype=int)
    w_source = source["weight"].to_numpy(dtype=float)
    groups = design_groups(source)
    rows: list[dict[str, object]] = []
    for c_value in PENALTY_GRID:
        oof = np.full(len(source), np.nan)
        splitter = StratifiedGroupKFold(
            n_splits=N_SPLITS,
            shuffle=True,
            random_state=SEED,
        )
        for train, test in splitter.split(np.zeros(len(source)), y_source, groups):
            model = logistic_pipeline(SCI_PREDICTORS, c_value)
            model.fit(
                source.iloc[train][SCI_PREDICTORS],
                y_source[train],
                model__sample_weight=normalized_weights(w_source[train]),
            )
            oof[test] = model.predict_proba(source.iloc[test][SCI_PREDICTORS])[:, 1]
        if np.isnan(oof).any():
            raise RuntimeError(f"Source cross fitting failed for C={c_value}.")
        threshold = weighted_quantile(oof, 0.90, w_source)
        rows.append(
            {
                "c_value": c_value,
                "year": 2015,
                "score_origin": "cross_fitted_source",
                **maintenance_metrics(y_source, oof, w_source, threshold),
            }
        )
        full_model = logistic_pipeline(SCI_PREDICTORS, c_value)
        full_model.fit(
            source[SCI_PREDICTORS],
            y_source,
            model__sample_weight=normalized_weights(w_source),
        )
        for year in TARGET_YEARS:
            wave = observed[observed["year"].eq(year)]
            prediction = full_model.predict_proba(wave[SCI_PREDICTORS])[:, 1]
            rows.append(
                {
                    "c_value": c_value,
                    "year": year,
                    "score_origin": "frozen_source",
                    **maintenance_metrics(
                        wave["attempt"].to_numpy(dtype=int),
                        prediction,
                        wave["weight"].to_numpy(dtype=float),
                        threshold,
                    ),
                }
            )
        report(f"Completed frozen source sensitivity for C={c_value:g}")
    return pd.DataFrame(rows)


def refit_penalty_sensitivity(
    observed: pd.DataFrame,
    frozen_model,
    frozen_threshold: float,
) -> pd.DataFrame:
    rows: list[dict[str, object]] = []
    for year in TARGET_YEARS:
        wave = observed[observed["year"].eq(year)].reset_index(drop=True)
        y = wave["attempt"].to_numpy(dtype=int)
        w = wave["weight"].to_numpy(dtype=float)
        groups = design_groups(wave)
        static_score = frozen_model.predict_proba(wave[SCI_PREDICTORS])[:, 1]
        for repeat in range(REPEATS):
            splitter = StratifiedGroupKFold(
                n_splits=N_SPLITS,
                shuffle=True,
                random_state=SEED + year * 100 + repeat,
            )
            folds = list(splitter.split(np.zeros(len(wave)), y, groups))
            for budget in PENALTY_BUDGETS:
                predictions = {
                    c_value: np.full(len(wave), np.nan) for c_value in PENALTY_GRID
                }
                for fold, (train, test) in enumerate(folds):
                    budget_index = ORIGINAL_BUDGET_INDEX[budget]
                    rng = np.random.default_rng(
                        SEED + year * 1_000_000 + repeat * 10_000 + fold * 100 + budget_index
                    )
                    update = proportional_stratum_sample(wave, train, budget, rng)
                    for c_value in PENALTY_GRID:
                        refitted = logistic_pipeline(SCI_PREDICTORS, c_value)
                        refitted.fit(
                            wave.iloc[update][SCI_PREDICTORS],
                            y[update],
                            model__sample_weight=normalized_weights(w[update]),
                        )
                        predictions[c_value][test] = refitted.predict_proba(
                            wave.iloc[test][SCI_PREDICTORS]
                        )[:, 1]
                for c_value, prediction in predictions.items():
                    if np.isnan(prediction).any():
                        raise RuntimeError(
                            f"Penalty sensitivity left missing predictions: {year}, "
                            f"repeat {repeat}, n={budget}, C={c_value}."
                        )
                    rows.append(
                        {
                            "target_year": year,
                            "repeat": repeat,
                            "budget": budget,
                            "refit_c_value": c_value,
                            **maintenance_metrics(y, prediction, w, frozen_threshold),
                        }
                    )
        report(f"Completed refit penalty sensitivity for {year}")
    return pd.DataFrame(rows)


def summarize_refit_penalties(runs: pd.DataFrame, original_runs: pd.DataFrame) -> pd.DataFrame:
    static = (
        original_runs[original_runs["strategy"].eq("static")]
        .drop_duplicates("target_year")
        .set_index("target_year")
    )
    intercept = original_runs[original_runs["strategy"].eq("intercept")][
        ["target_year", "repeat", "budget", "brier", "auroc", "absolute_calibration_intercept_error"]
    ].rename(
        columns={
            "brier": "intercept_brier",
            "auroc": "intercept_auroc",
            "absolute_calibration_intercept_error": "intercept_abs_calibration_intercept",
        }
    )
    merged = runs.merge(intercept, on=["target_year", "repeat", "budget"], how="left")
    for metric in ["brier", "auroc", "absolute_calibration_intercept_error"]:
        merged[f"delta_{metric}_vs_static"] = merged[metric] - merged["target_year"].map(
            static[metric]
        )
    merged["delta_brier_vs_intercept"] = merged["brier"] - merged["intercept_brier"]
    merged["delta_auroc_vs_intercept"] = merged["auroc"] - merged["intercept_auroc"]
    merged["delta_abs_calibration_intercept_vs_intercept"] = (
        merged["absolute_calibration_intercept_error"]
        - merged["intercept_abs_calibration_intercept"]
    )
    metrics = [
        "brier",
        "auroc",
        "absolute_calibration_intercept_error",
        "absolute_calibration_slope_error",
        "delta_brier_vs_static",
        "delta_auroc_vs_static",
        "delta_absolute_calibration_intercept_error_vs_static",
        "delta_brier_vs_intercept",
        "delta_auroc_vs_intercept",
        "delta_abs_calibration_intercept_vs_intercept",
    ]
    records: list[dict[str, object]] = []
    for keys, group in merged.groupby(["target_year", "budget", "refit_c_value"], sort=True):
        record = dict(zip(["target_year", "budget", "refit_c_value"], keys, strict=True))
        record["repetitions"] = int(group["repeat"].nunique())
        calibration_valid = np.isfinite(group["calibration_intercept"]) & np.isfinite(
            group["calibration_slope"]
        )
        record["calibration_valid_runs"] = int(calibration_valid.sum())
        record["calibration_failure_rate"] = float(1.0 - calibration_valid.mean())
        for metric in metrics:
            values = group[metric].dropna().to_numpy(dtype=float)
            record[f"{metric}_valid_runs"] = int(len(values))
            if len(values) == 0:
                record[f"{metric}_median"] = np.nan
                record[f"{metric}_p05"] = np.nan
                record[f"{metric}_p95"] = np.nan
            else:
                record[f"{metric}_median"] = float(np.median(values))
                record[f"{metric}_p05"] = float(np.quantile(values, 0.05))
                record[f"{metric}_p95"] = float(np.quantile(values, 0.95))
        records.append(record)
    return pd.DataFrame(records)


def weighted_mean(values: np.ndarray, weights: np.ndarray) -> float:
    return float(np.average(np.asarray(values, dtype=float), weights=np.asarray(weights, dtype=float)))


def logit_mean(value: float) -> float:
    return float(logit(np.clip(value, 1e-8, 1 - 1e-8)))


def drift_decomposition(observed: pd.DataFrame, frozen_model) -> pd.DataFrame:
    source = observed[observed["year"].eq(2015)].copy()
    source_y = source["attempt"].to_numpy(dtype=float)
    source_w = source["weight"].to_numpy(dtype=float)
    source_pred = frozen_model.predict_proba(source[SCI_PREDICTORS])[:, 1]
    source_observed = weighted_mean(source_y, source_w)
    source_expected = weighted_mean(source_pred, source_w)
    rows: list[dict[str, object]] = []
    for year in TARGET_YEARS:
        target = observed[observed["year"].eq(year)].copy()
        target_y = target["attempt"].to_numpy(dtype=float)
        target_w = target["weight"].to_numpy(dtype=float)
        source_on_target = frozen_model.predict_proba(target[SCI_PREDICTORS])[:, 1]
        target_model = logistic_pipeline(SCI_PREDICTORS, 0.1)
        target_model.fit(
            target[SCI_PREDICTORS],
            target_y.astype(int),
            model__sample_weight=normalized_weights(target_w),
        )
        target_on_target = target_model.predict_proba(target[SCI_PREDICTORS])[:, 1]
        target_observed = weighted_mean(target_y, target_w)
        source_model_target_mean = weighted_mean(source_on_target, target_w)
        target_model_target_mean = weighted_mean(target_on_target, target_w)

        total_rd = target_observed - source_observed
        covariate_rd = source_model_target_mean - source_expected
        coefficient_rd = target_model_target_mean - source_model_target_mean
        residual_rd = (
            target_observed
            - target_model_target_mean
            - (source_observed - source_expected)
        )

        total_logodds = logit_mean(target_observed) - logit_mean(source_observed)
        covariate_logodds = logit_mean(source_model_target_mean) - logit_mean(source_expected)
        coefficient_logodds = logit_mean(target_model_target_mean) - logit_mean(
            source_model_target_mean
        )
        residual_logodds = (
            logit_mean(target_observed)
            - logit_mean(target_model_target_mean)
            - (logit_mean(source_observed) - logit_mean(source_expected))
        )
        rows.append(
            {
                "source_year": 2015,
                "target_year": year,
                "source_observed_prevalence": source_observed,
                "source_model_mean_in_source": source_expected,
                "target_observed_prevalence": target_observed,
                "source_model_mean_in_target": source_model_target_mean,
                "target_model_mean_in_target": target_model_target_mean,
                "total_prevalence_shift_risk_difference": total_rd,
                "covariate_shift_component_risk_difference": covariate_rd,
                "coefficient_shift_component_risk_difference": coefficient_rd,
                "model_residual_component_risk_difference": residual_rd,
                "risk_difference_reconstruction_error": total_rd
                - (covariate_rd + coefficient_rd + residual_rd),
                "total_prevalence_shift_logodds": total_logodds,
                "covariate_shift_component_logodds": covariate_logodds,
                "coefficient_shift_component_logodds": coefficient_logodds,
                "model_residual_component_logodds": residual_logodds,
                "logodds_reconstruction_error": total_logodds
                - (covariate_logodds + coefficient_logodds + residual_logodds),
                "covariate_share_of_total_risk_difference": (
                    covariate_rd / total_rd if abs(total_rd) > 1e-12 else np.nan
                ),
                "coefficient_share_of_total_risk_difference": (
                    coefficient_rd / total_rd if abs(total_rd) > 1e-12 else np.nan
                ),
            }
        )
    return pd.DataFrame(rows)


def nearest_stratum_collapse(wave: pd.DataFrame) -> tuple[pd.Series, dict[int, int]]:
    counts = wave.groupby("stratum", observed=False)["psu"].nunique()
    singletons = counts[counts.eq(1)].index.astype(int).tolist()
    eligible = counts[counts.ge(2)].index.astype(int).tolist()
    mapping: dict[int, int] = {}
    for stratum in singletons:
        block = stratum // 100
        candidates = [candidate for candidate in eligible if candidate // 100 == block]
        if not candidates:
            candidates = eligible
        mapping[stratum] = min(candidates, key=lambda candidate: (abs(candidate - stratum), candidate))
    collapsed = wave["stratum"].astype(int).replace(mapping)
    return collapsed, mapping


def rao_wu_multiplier(
    frame: pd.DataFrame,
    stratum_column: str,
    rng: np.random.Generator,
) -> np.ndarray:
    multiplier = np.zeros(len(frame), dtype=float)
    for _, indices in frame.groupby(stratum_column, observed=False).groups.items():
        positions = np.asarray(list(indices), dtype=int)
        psu = frame.iloc[positions]["psu"].astype(str).to_numpy()
        unique_psu = np.unique(psu)
        number = len(unique_psu)
        if number < 2:
            multiplier[positions] = 1.0
            continue
        sampled = rng.choice(unique_psu, size=number - 1, replace=True)
        values, counts = np.unique(sampled, return_counts=True)
        factor = number / (number - 1)
        for value, count in zip(values, counts, strict=True):
            multiplier[positions[psu == value]] = factor * count
    return multiplier


def single_psu_sensitivity(
    scored: pd.DataFrame,
    threshold: float,
    frozen_points: pd.DataFrame,
    frozen_summary: pd.DataFrame,
) -> tuple[pd.DataFrame, pd.DataFrame]:
    """Variance sensitivity for singleton PSU strata.

    The certainty treatment is the frozen primary bootstrap, which already
    treated analytically restricted singleton strata as certainty units.
    Its intervals are reported directly from the frozen summary so that the
    reference condition reproduces the primary results in Table 2 exactly.
    Merging and omission are genuinely new bootstrap runs with their own
    fixed seeds.
    """
    summary_rows: list[dict[str, object]] = []
    mapping_rows: list[dict[str, object]] = []
    metrics_to_report = ["calibration_intercept", "calibration_slope", "auroc"]
    frozen_ci = frozen_summary[frozen_summary["metric"].isin(metrics_to_report)].copy()
    for year in [2017, 2019, 2023]:
        full_wave = scored[scored["year"].eq(year)].reset_index(drop=True)
        psu_counts = full_wave.groupby("stratum", observed=False)["psu"].nunique()
        singleton_strata = psu_counts[psu_counts.eq(1)].index.astype(int).tolist()
        point_row = frozen_points[frozen_points["year"].eq(year)].iloc[0]
        schemes: list[tuple[str, pd.DataFrame | None, str | None]] = [
            ("certainty", None, None)
        ]
        if singleton_strata:
            collapsed = full_wave.copy()
            collapsed["bootstrap_stratum"], mapping = nearest_stratum_collapse(collapsed)
            schemes.append(("nearest_stratum_collapse", collapsed, "bootstrap_stratum"))
            for source, target in mapping.items():
                mapping_rows.append(
                    {
                        "year": year,
                        "singleton_stratum": source,
                        "collapsed_with_stratum": target,
                    }
                )
            omitted = full_wave[~full_wave["stratum"].isin(singleton_strata)].reset_index(drop=True)
            omitted["bootstrap_stratum"] = omitted["stratum"].astype(int)
            schemes.append(("omit_singleton_strata", omitted, "bootstrap_stratum"))
        for scheme_index, (scheme, wave, column) in enumerate(schemes):
            if scheme == "certainty":
                for metric in metrics_to_report:
                    frozen = frozen_ci[
                        (frozen_ci["year"].eq(year)) & (frozen_ci["metric"].eq(metric))
                    ].iloc[0]
                    summary_rows.append(
                        {
                            "year": year,
                            "scheme": scheme,
                            "metric": metric,
                            "analytic_n": int(len(full_wave)),
                            "singleton_strata_in_original": int(len(singleton_strata)),
                            "bootstrap_replicates": int(frozen["bootstrap_replicates"]),
                            "point_estimate": float(point_row[metric]),
                            "ci_lower": float(frozen["ci_lower"]),
                            "ci_upper": float(frozen["ci_upper"]),
                            "bootstrap_standard_error": float(
                                frozen["bootstrap_standard_error"]
                            ),
                        }
                    )
                continue
            y = wave["attempt"].to_numpy(dtype=int)
            score = wave["frozen_score"].to_numpy(dtype=float)
            base_weight = wave["weight"].to_numpy(dtype=float)
            point = maintenance_metrics(y, score, base_weight, threshold)
            rng = np.random.default_rng(20260804 + year * 10 + scheme_index)
            replicate_values = {metric: [] for metric in metrics_to_report}
            for _ in range(BOOTSTRAP_REPLICATES):
                weights = base_weight * rao_wu_multiplier(wave, column, rng)
                values = maintenance_metrics(y, score, weights, threshold)
                for metric in metrics_to_report:
                    replicate_values[metric].append(values[metric])
            for metric in metrics_to_report:
                values = np.asarray(replicate_values[metric], dtype=float)
                summary_rows.append(
                    {
                        "year": year,
                        "scheme": scheme,
                        "metric": metric,
                        "analytic_n": int(len(wave)),
                        "singleton_strata_in_original": int(len(singleton_strata)),
                        "bootstrap_replicates": BOOTSTRAP_REPLICATES,
                        "point_estimate": float(point[metric]),
                        "ci_lower": float(np.quantile(values, 0.025)),
                        "ci_upper": float(np.quantile(values, 0.975)),
                        "bootstrap_standard_error": float(np.std(values, ddof=1)),
                    }
                )
        report(f"Completed single PSU sensitivity for {year}")
    return pd.DataFrame(summary_rows), pd.DataFrame(mapping_rows)


def bootstrap_median_interval(values: np.ndarray, rng: np.random.Generator) -> tuple[float, float]:
    draws = rng.choice(values, size=(20000, len(values)), replace=True)
    medians = np.median(draws, axis=1)
    return float(np.quantile(medians, 0.025)), float(np.quantile(medians, 0.975))


def paired_nonparametric_summary(runs: pd.DataFrame) -> pd.DataFrame:
    metric_direction = {
        "absolute_calibration_intercept_error": "lower",
        "absolute_calibration_slope_error": "lower",
        "brier": "lower",
        "auroc": "higher",
    }
    comparisons = [
        ("intercept", "static"),
        ("logistic", "static"),
        ("refit", "static"),
        ("intercept", "logistic"),
        ("intercept", "refit"),
        ("logistic", "refit"),
    ]
    static = runs[runs["strategy"].eq("static")].drop_duplicates("target_year").set_index(
        "target_year"
    )
    updated = runs[(runs["budget"].gt(0)) & (~runs["strategy"].eq("static"))].copy()
    rows: list[dict[str, object]] = []
    rng = np.random.default_rng(2026080413)
    for (year, budget), group in updated.groupby(["target_year", "budget"], sort=True):
        wide = group.pivot(index="repeat", columns="strategy")
        for metric, direction in metric_direction.items():
            for strategy_a, strategy_b in comparisons:
                if strategy_a == "static":
                    values_a = np.repeat(float(static.loc[year, metric]), len(wide))
                else:
                    values_a = wide[(metric, strategy_a)].to_numpy(dtype=float)
                if strategy_b == "static":
                    values_b = np.repeat(float(static.loc[year, metric]), len(wide))
                else:
                    values_b = wide[(metric, strategy_b)].to_numpy(dtype=float)
                difference = values_a - values_b
                if np.allclose(difference, 0.0, atol=1e-15, rtol=0.0):
                    statistic = 0.0
                    p_value = 1.0
                else:
                    test = wilcoxon(
                        difference,
                        zero_method="wilcox",
                        alternative="two-sided",
                        method="auto",
                    )
                    statistic = float(test.statistic)
                    p_value = float(test.pvalue)
                lower, upper = bootstrap_median_interval(difference, rng)
                favorable = difference < 0 if direction == "lower" else difference > 0
                ties = np.isclose(difference, 0.0, atol=1e-12, rtol=0.0)
                rows.append(
                    {
                        "target_year": int(year),
                        "budget": int(budget),
                        "metric": metric,
                        "preferred_direction": direction,
                        "strategy_a": strategy_a,
                        "strategy_b": strategy_b,
                        "repetitions": int(len(difference)),
                        "median_difference_a_minus_b": float(np.median(difference)),
                        "paired_bootstrap_median_ci_lower": lower,
                        "paired_bootstrap_median_ci_upper": upper,
                        "a_favorable_rate": float(np.mean(favorable)),
                        "tie_rate": float(np.mean(ties)),
                        "wilcoxon_statistic": statistic,
                        "wilcoxon_two_sided_p": p_value,
                        "interpretation_scope": "algorithmic resampling stability, not population inference",
                    }
                )
    return pd.DataFrame(rows)


def main() -> None:
    OUT.mkdir(parents=True, exist_ok=True)
    observed, frozen_model, threshold = load_analysis_data()
    scored = add_frozen_scores(observed, frozen_model)
    original_runs = pd.read_csv(PRIMARY_RESULTS / "maintenance_learning_curve_runs.csv")

    source_penalties = source_penalty_sensitivity(observed)
    source_penalties.to_csv(OUT / "source_penalty_sensitivity.csv", index=False)

    refit_runs = refit_penalty_sensitivity(observed, frozen_model, threshold)
    refit_runs.to_csv(OUT / "refit_penalty_runs.csv", index=False)
    refit_summary = summarize_refit_penalties(refit_runs, original_runs)
    refit_summary.to_csv(OUT / "refit_penalty_summary.csv", index=False)

    decomposition = drift_decomposition(observed, frozen_model)
    decomposition.to_csv(OUT / "drift_decomposition.csv", index=False)

    frozen_points = pd.read_csv(PRIMARY_RESULTS / "frozen_point_estimates.csv")
    frozen_summary = pd.read_csv(PRIMARY_RESULTS / "frozen_bootstrap_summary.csv")
    single_psu, mapping = single_psu_sensitivity(
        scored, threshold, frozen_points, frozen_summary
    )
    single_psu.to_csv(OUT / "single_psu_variance_sensitivity.csv", index=False)
    mapping.to_csv(OUT / "single_psu_collapse_map.csv", index=False)

    paired = paired_nonparametric_summary(original_runs)
    paired.to_csv(OUT / "paired_nonparametric_summary.csv", index=False)

    metadata = {
        "created": time.strftime("%Y-%m-%d %H:%M:%S"),
        "data_path": str(DATA_PATH),
        "analytic_n": int(len(observed)),
        "analytic_events": int(observed["attempt"].sum()),
        "penalty_grid": PENALTY_GRID,
        "penalty_budgets": PENALTY_BUDGETS,
        "penalty_repetitions": REPEATS,
        "single_psu_bootstrap_replicates": BOOTSTRAP_REPLICATES,
        "paired_bootstrap_draws": 20000,
        "decomposition_scale": ["risk difference", "log odds"],
    }
    (OUT / "revision_analysis_metadata.json").write_text(
        json.dumps(metadata, indent=2), encoding="utf-8"
    )
    report(f"Saved revision analyses to {OUT}")


if __name__ == "__main__":
    main()
```
