# Supplementary material for: Temporal maintenance of an adolescent suicide attempt screening score in national surveys: An observational study
Source: Medicine (Baltimore). 2026 Aug 28;105(35):e50513. doi: 10.1097/MD.0000000000050513 (PMC13529099; doi:10.1097/MD.0000000000050513)
Supplement: Supplementary file 14 [file medi-105-e50513-s014.docx]

**Supplemental Digital Content 9**

**Revision verification code**

Temporal maintenance of an adolescent suicide attempt screening score in national surveys: An observational study

First author: Xiaotian Ma

*Source file: verify_revision_analyses.py*

This Word document contains the complete source code for the identified analysis step.

```
"""Independent numerical and structural checks for the Medicine revision."""

from __future__ import annotations

import json
from pathlib import Path

import numpy as np
import pandas as pd


ROOT = Path(__file__).resolve().parents[1]
PRIMARY = ROOT / "results" / "sci_maintenance"
REVISION = ROOT / "results" / "revision"


def require(condition: bool, message: str) -> None:
    if not condition:
        raise AssertionError(message)


def main() -> None:
    source = pd.read_csv(REVISION / "source_penalty_sensitivity.csv")
    original_points = pd.read_csv(PRIMARY / "frozen_point_estimates.csv")
    base = source[np.isclose(source["c_value"], 0.1)].sort_values("year")
    original = original_points.sort_values("year")
    require(base["year"].tolist() == original["year"].tolist(), "Frozen years differ.")
    for metric in ["auroc", "brier", "calibration_intercept", "calibration_slope"]:
        difference = np.max(np.abs(base[metric].to_numpy() - original[metric].to_numpy()))
        require(difference < 2e-10, f"C=0.1 source sensitivity does not reproduce {metric}: {difference}")

    refit = pd.read_csv(REVISION / "refit_penalty_runs.csv")
    original_runs = pd.read_csv(PRIMARY / "maintenance_learning_curve_runs.csv")
    original_refit = original_runs[
        (original_runs["strategy"].eq("refit"))
        & (original_runs["budget"].isin([250, 1000]))
    ].sort_values(["target_year", "budget", "repeat"])
    base_refit = refit[np.isclose(refit["refit_c_value"], 0.1)].sort_values(
        ["target_year", "budget", "repeat"]
    )
    require(len(base_refit) == len(original_refit), "C=0.1 refit row count differs.")
    for metric in ["auroc", "brier", "calibration_intercept", "calibration_slope"]:
        difference = np.max(
            np.abs(base_refit[metric].to_numpy() - original_refit[metric].to_numpy())
        )
        require(difference < 2e-10, f"C=0.1 refit does not reproduce {metric}: {difference}")

    decomposition = pd.read_csv(REVISION / "drift_decomposition.csv")
    require(
        decomposition["risk_difference_reconstruction_error"].abs().max() < 1e-12,
        "Risk difference decomposition does not reconstruct the total.",
    )
    require(
        decomposition["logodds_reconstruction_error"].abs().max() < 1e-12,
        "Log odds decomposition does not reconstruct the total.",
    )

    single = pd.read_csv(REVISION / "single_psu_variance_sensitivity.csv")
    require(set(single["year"]) == {2017, 2019, 2023}, "Single PSU years are incomplete.")
    require(
        set(single[single["year"].eq(2023)]["scheme"]) == {"certainty"},
        "A single PSU sensitivity was incorrectly applied to 2023.",
    )
    mapping = pd.read_csv(REVISION / "single_psu_collapse_map.csv")
    require(len(mapping) == 4, "Expected four analytically restricted singleton strata.")
    frozen_summary = pd.read_csv(PRIMARY / "frozen_bootstrap_summary.csv")
    frozen_points = pd.read_csv(PRIMARY / "frozen_point_estimates.csv")
    for year in [2017, 2019, 2023]:
        for metric in ["calibration_intercept", "calibration_slope", "auroc"]:
            certain = single[
                (single["year"].eq(year))
                & (single["scheme"].eq("certainty"))
                & (single["metric"].eq(metric))
            ]
            frozen = frozen_summary[
                (frozen_summary["year"].eq(year)) & (frozen_summary["metric"].eq(metric))
            ]
            require(
                len(certain) == 1 and len(frozen) == 1,
                f"Certainty or frozen summary row is missing for {year} {metric}.",
            )
            certain = certain.iloc[0]
            frozen = frozen.iloc[0]
            require(
                int(certain["bootstrap_replicates"]) == int(frozen["bootstrap_replicates"]),
                f"Certainty replicate count does not match frozen for {year} {metric}.",
            )
            for field in ("point_estimate", "ci_lower", "ci_upper", "bootstrap_standard_error"):
                require(
                    abs(float(certain[field]) - float(frozen[field])) < 1e-12,
                    f"Certainty {field} does not reproduce the frozen primary for {year} {metric}.",
                )
            require(
                abs(float(certain["point_estimate"]) - float(frozen_points.loc[
                    frozen_points["year"].eq(year)
                ][metric].iloc[0])) < 1e-12,
                f"Certainty point estimate does not match the frozen points for {year} {metric}.",
            )

    paired = pd.read_csv(REVISION / "paired_nonparametric_summary.csv")
    require(paired["repetitions"].eq(50).all(), "Paired comparisons do not all use 50 repetitions.")
    require(paired["wilcoxon_two_sided_p"].between(0, 1).all(), "Invalid Wilcoxon P value.")

    metadata = json.loads((REVISION / "revision_analysis_metadata.json").read_text())
    require(metadata["analytic_n"] == 68682, "Revision metadata analytic count is wrong.")
    require(metadata["analytic_events"] == 6855, "Revision metadata event count is wrong.")
    print("MEDICINE_REVISION_ANALYSES_VERIFICATION_PASS")


if __name__ == "__main__":
    main()
```
