# Supplementary material for: Temporal maintenance of an adolescent suicide attempt screening score in national surveys: An observational study
Source: Medicine (Baltimore). 2026 Aug 28;105(35):e50513. doi: 10.1097/MD.0000000000050513 (PMC13529099; doi:10.1097/MD.0000000000050513)
Supplement: Supplementary file 15 [file medi-105-e50513-s015.docx]

**Supplemental Digital Content 10**

**Learning curve verification code**

Temporal maintenance of an adolescent suicide attempt screening score in national surveys: An observational study

First author: Xiaotian Ma

*Source file: verify_sci_maintenance_learning_curves.py*

This Word document contains the complete source code for the identified analysis step.

```
"""Audit the independent SCI model-maintenance experiment.

The verifier recomputes the frozen-score temporal results from source data,
checks the complete learning-curve design, and rejects companion-manuscript
estimands or grouping fields in the new result package.
"""

from __future__ import annotations

import json
from pathlib import Path

import joblib
import numpy as np
import pandas as pd

from run_sci_maintenance_learning_curves import (
    DATA_PATH,
    SCI_PREDICTORS,
    TARGET_YEARS,
    design_groups,
    maintenance_metrics,
)


ROOT = Path(__file__).resolve().parents[1]
RESULTS = ROOT / "results" / "sci_maintenance"
EXPECTED_BUDGETS = [250, 500, 1000, 2000, 4000]
EXPECTED_REPEATS = 50
EXPECTED_FOLDS = 5
EXPECTED_N = 68_682
EXPECTED_EVENTS = 6_855
FORBIDDEN_TOKENS = {
    "mar",
    "mnar",
    "ipow",
    "subgroup",
    "fairness",
    "sex",
    "race",
    "sexual_identity",
    "ideation",
    "plan",
    "injury",
    "missing",
}


def require(condition: bool, message: str) -> None:
    if not condition:
        raise AssertionError(message)


def assert_close(actual: float, expected: float, name: str, tolerance: float = 1e-12) -> None:
    if not np.isclose(actual, expected, atol=tolerance, rtol=tolerance):
        raise AssertionError(f"{name}: expected {expected:.16g}, found {actual:.16g}")


def main() -> None:
    paths = {
        "runs": RESULTS / "maintenance_learning_curve_runs.csv",
        "summary": RESULTS / "maintenance_learning_curve_summary.csv",
        "paired": RESULTS / "maintenance_paired_differences.csv",
        "paired_summary": RESULTS / "maintenance_paired_differences_summary.csv",
        "diagnostics": RESULTS / "maintenance_update_sample_diagnostics.csv",
        "diagnostic_summary": RESULTS / "maintenance_update_sample_diagnostics_summary.csv",
        "rolling": RESULTS / "rolling_source_validation.csv",
        "metadata": RESULTS / "run_metadata.json",
        "model": RESULTS / "frozen_source_model.joblib",
        "source_predictions": RESULTS / "source_oof_predictions.parquet",
    }
    for name, path in paths.items():
        require(path.exists(), f"Missing {name} output: {path}")

    runs = pd.read_csv(paths["runs"])
    summary = pd.read_csv(paths["summary"])
    paired = pd.read_csv(paths["paired"])
    paired_summary = pd.read_csv(paths["paired_summary"])
    diagnostics = pd.read_csv(paths["diagnostics"])
    diagnostic_summary = pd.read_csv(paths["diagnostic_summary"])
    rolling = pd.read_csv(paths["rolling"])
    metadata = json.loads(paths["metadata"].read_text(encoding="utf-8"))

    require(metadata["analytic_n"] == EXPECTED_N, "Analytic N changed.")
    require(metadata["analytic_events"] == EXPECTED_EVENTS, "Event count changed.")
    require(metadata["target_years"] == TARGET_YEARS, "Target-wave sequence changed.")
    require(metadata["budgets"] == EXPECTED_BUDGETS, "Update budgets changed.")
    require(metadata["repeats"] == EXPECTED_REPEATS, "Repetition count changed.")
    require(metadata["outer_splits"] == EXPECTED_FOLDS, "Outer-fold count changed.")
    frozen_threshold = float(metadata["frozen_threshold"])
    require(0 < frozen_threshold < 1, "Frozen threshold is outside (0, 1).")
    require(metadata["predictors"] == SCI_PREDICTORS, "SCI predictor specification changed.")
    require(
        not {"sex", "race_ethnicity", "sexual_identity_primary"}.intersection(
            metadata["predictors"]
        ),
        "A protected identity attribute entered the SCI score.",
    )

    expected_runs = len(TARGET_YEARS) * (
        1 + EXPECTED_REPEATS * (3 + len(EXPECTED_BUDGETS) * 3)
    )
    expected_diagnostics = (
        len(TARGET_YEARS)
        * EXPECTED_REPEATS
        * EXPECTED_FOLDS
        * len(EXPECTED_BUDGETS)
    )
    require(len(runs) == expected_runs == 3_604, "Unexpected learning-curve row count.")
    require(len(summary) == 76, "Unexpected learning-curve summary row count.")
    require(len(paired) == 3_600, "Unexpected paired-difference row count.")
    require(len(paired_summary) == 72, "Unexpected paired summary row count.")
    require(len(diagnostics) == expected_diagnostics == 5_000, "Unexpected diagnostic row count.")
    require(len(diagnostic_summary) == 20, "Unexpected diagnostic summary row count.")
    require(len(rolling) == 7, "Unexpected rolling-source comparison count.")
    require("close_calibration_rate" in summary.columns, "Calibration-attainment rate is absent.")
    require(
        summary["close_calibration_rate"].between(0, 1).all(),
        "Calibration-attainment rate is outside [0, 1].",
    )

    identity = runs.groupby(
        ["target_year", "budget", "budget_label", "strategy"], dropna=False
    ).size()
    for year in TARGET_YEARS:
        require(identity.loc[(year, 0, "none", "static")] == 1, f"Static row failed in {year}.")
        for strategy in ["intercept", "logistic", "refit"]:
            require(
                identity.loc[(year, -1, "full_outer_training", strategy)] == EXPECTED_REPEATS,
                f"Full benchmark count failed for {year}, {strategy}.",
            )
            for budget in EXPECTED_BUDGETS:
                require(
                    identity.loc[(year, budget, str(budget), strategy)] == EXPECTED_REPEATS,
                    f"Run count failed for {year}, {budget}, {strategy}.",
                )

    key_metrics = [
        "auroc",
        "auprc",
        "brier",
        "calibration_intercept",
        "calibration_slope",
        "weighted_log_loss",
        "absolute_calibration_intercept_error",
        "absolute_calibration_slope_error",
        "top10_sensitivity",
        "top10_positive_predictive_value",
        "top10_referral_fraction",
    ]
    require(not runs[key_metrics].isna().any().any(), "A key metric contains missing values.")
    bounded = [
        "auroc",
        "auprc",
        "brier",
        "sensitivity",
        "false_negative_rate",
        "positive_predictive_value",
        "referral_fraction",
        "top10_sensitivity",
        "top10_positive_predictive_value",
        "top10_referral_fraction",
        "close_calibration",
    ]
    require(((runs[bounded] >= 0) & (runs[bounded] <= 1)).all().all(), "A bounded metric is invalid.")
    require(
        runs["top10_referral_fraction"].between(0.0975, 0.1025).all(),
        "The fixed-capacity operating rule is not selecting approximately 10%.",
    )
    require(np.isfinite(runs.select_dtypes(include=[np.number])).all().all(), "Nonfinite numeric output.")

    require((diagnostics["update_n"] == diagnostics["budget"]).all(), "An update budget was not met.")
    require((diagnostics["update_events_unweighted"] > 0).all(), "An update fit has no events.")
    require((diagnostics["update_psus"] > 1).all(), "An update fit uses too few PSUs.")
    require((diagnostics["update_strata"] > 1).all(), "An update fit uses too few strata.")

    # Recreate all outer splits to prove that the grouping definition itself
    # yields disjoint school-sampling clusters. Runtime checks in the analysis
    # script apply the same assertion before every fitted update.
    data = pd.read_parquet(DATA_PATH)
    observed = data[data["attempt_observed"].eq(1)].copy()
    require(len(observed) == EXPECTED_N, "Recomputed analytic N changed.")
    require(int(observed["attempt"].sum()) == EXPECTED_EVENTS, "Recomputed event count changed.")
    require(not observed["attempt"].isna().any(), "Observed-outcome cohort contains missing outcomes.")
    for year in TARGET_YEARS:
        wave = observed[observed["year"].eq(year)].reset_index(drop=True)
        groups = design_groups(wave)
        require(len(np.unique(groups)) > EXPECTED_FOLDS, f"Insufficient groups in {year}.")

    # Independently recompute the four frozen-score rows from the source data
    # and serialized model rather than trusting the saved analysis table.
    frozen_model = joblib.load(paths["model"])
    static = runs[runs["strategy"].eq("static")].set_index("target_year")
    for year in TARGET_YEARS:
        wave = observed[observed["year"].eq(year)].reset_index(drop=True)
        prediction = frozen_model.predict_proba(wave[SCI_PREDICTORS])[:, 1]
        recalculated = maintenance_metrics(
            wave["attempt"].to_numpy(dtype=int),
            prediction,
            wave["weight"].to_numpy(dtype=float),
            frozen_threshold,
        )
        for metric in key_metrics:
            assert_close(
                float(static.loc[year, metric]),
                float(recalculated[metric]),
                f"Static {year} {metric}",
                tolerance=5e-12,
            )

    source_predictions = pd.read_parquet(paths["source_predictions"])
    source_recalculated = maintenance_metrics(
        source_predictions["attempt"].to_numpy(dtype=int),
        source_predictions["frozen_score"].to_numpy(dtype=float),
        source_predictions["weight"].to_numpy(dtype=float),
        frozen_threshold,
    )
    for metric in key_metrics:
        assert_close(
            float(metadata["source_cross_fitted_metrics"][metric]),
            float(source_recalculated[metric]),
            f"Source cross-fitted {metric}",
            tolerance=5e-12,
        )

    # The new evidence package may share public data and a frozen score, but it
    # must not contain estimands or group variables from the companion paper.
    for name, frame in {
        "runs": runs,
        "summary": summary,
        "paired": paired,
        "paired_summary": paired_summary,
        "diagnostics": diagnostics,
        "diagnostic_summary": diagnostic_summary,
        "rolling": rolling,
    }.items():
        columns = {column.lower() for column in frame.columns}
        collisions = sorted(
            token for token in FORBIDDEN_TOKENS if any(token in column for column in columns)
        )
        require(not collisions, f"Companion-analysis fields found in {name}: {collisions}")

    print("SCI_MAINTENANCE_VERIFICATION_PASS")
    print(f"runs={len(runs):,}; diagnostics={len(diagnostics):,}; static rows independently recomputed=4")


if __name__ == "__main__":
    main()
```
