# Supplementary material for: Temporal maintenance of an adolescent suicide attempt screening score in national surveys: An observational study
Source: Medicine (Baltimore). 2026 Aug 28;105(35):e50513. doi: 10.1097/MD.0000000000050513 (PMC13529099; doi:10.1097/MD.0000000000050513)
Supplement: Supplementary file 16 [file medi-105-e50513-s016.docx]

**Supplemental Digital Content 11**

**Source verification code**

Temporal maintenance of an adolescent suicide attempt screening score in national surveys: An observational study

First author: Xiaotian Ma

*Source file: verify_source_checksums.py*

This Word document contains the complete source code for the identified analysis step.

```
from __future__ import annotations

import csv
import hashlib
import os
from pathlib import Path


ROOT = Path(__file__).resolve().parents[1]
MANIFEST = ROOT / "raw_data" / "source_checksums.csv"


def candidate_directories() -> list[Path]:
    candidates: list[Path] = []
    if os.environ.get("YRBS_RAW_DIR"):
        candidates.append(Path(os.environ["YRBS_RAW_DIR"]).expanduser())
    candidates.append(ROOT / "raw_data")
    # The public source files are intentionally not redistributed. Search
    # upward for the workspace-level download directory when the package is
    # being verified in its development workspace; external users can set
    # YRBS_RAW_DIR after downloading the same CDC files.
    for ancestor in (ROOT, *ROOT.parents):
        candidates.append(ancestor / "frontiers_psychology_yrbs_topic_audit" / "cdc_yrbs")
    resolved: list[Path] = []
    for path in candidates:
        candidate = path.resolve()
        if candidate not in resolved:
            resolved.append(candidate)
    return resolved


def locate_source_directory(filenames: list[str]) -> Path:
    for candidate in candidate_directories():
        if all((candidate / filename).is_file() for filename in filenames):
            return candidate
    searched = "\n  - ".join(str(path) for path in candidate_directories())
    raise FileNotFoundError(
        "No directory contains every file in the checksum manifest. Searched:\n  - " + searched
    )


def sha256(path: Path) -> str:
    digest = hashlib.sha256()
    with path.open("rb") as handle:
        for block in iter(lambda: handle.read(1024 * 1024), b""):
            digest.update(block)
    return digest.hexdigest()


def main() -> None:
    with MANIFEST.open(newline="", encoding="utf-8") as handle:
        rows = list(csv.DictReader(handle))
    source = locate_source_directory([row["filename"] for row in rows])
    failures: list[str] = []
    for row in rows:
        path = source / row["filename"]
        actual_size = path.stat().st_size
        actual_hash = sha256(path)
        expected_size = int(row["bytes"])
        expected_hash = row["sha256"].lower()
        status = "PASS" if actual_size == expected_size and actual_hash == expected_hash else "FAIL"
        print(f"{status}\t{row['filename']}\t{actual_size}\t{actual_hash}")
        if status == "FAIL":
            failures.append(row["filename"])
    print(f"Verified {len(rows) - len(failures)}/{len(rows)} source files in {source}")
    if failures:
        raise SystemExit("Checksum mismatch: " + ", ".join(failures))


if __name__ == "__main__":
    main()
```
