# Supplementary material for: Temporal maintenance of an adolescent suicide attempt screening score in national surveys: An observational study
Source: Medicine (Baltimore). 2026 Aug 28;105(35):e50513. doi: 10.1097/MD.0000000000050513 (PMC13529099; doi:10.1097/MD.0000000000050513)
Supplement: Supplementary file 17 [file medi-105-e50513-s017.docx]

**Supplemental Digital Content 20**

**Source checksums**

Temporal maintenance of an adolescent suicide attempt screening score in national surveys: An observational study

First author: Xiaotian Ma

*Source file: source_checksums.csv*

This Word document contains aggregate or metadata records only and does not include individual student records.

filename,bytes,sha256

2015.dat,6608952,d557f559b8be4301f1b2e790cab3cea5cc15ca10c119b55f72eadc3692d0332b

2015.sps,27663,e87ffaba6f6a91fa05d1430f7502ae4d15d71a1440116abf3c1faaaa8a6b58f2

2015_guide.pdf,625734,0c8edc98ccad82322b33a75a3d0ab6b31f84a3b1c07e53c32349d29e6266a1bd

2017.dat,6334185,99f7a87f1f936b21674603064118222247e797323377de7c7365e75a26c6f4b9

2017.sps,28041,4c24784ef0efe74d5fa49172d612aee6612bebf5a46ed84fa9c712a233560a4d

2017_guide.pdf,1088143,3ac5a8b3e62cef9007c5c86f4b8a6eaba15ebd82ef77332335e6670e5387370a

2019.dat,5758017,9bfb3e87c519418eb6bd5b491ecfc723c1b21b85dbe796816fcce6b3cea8ca26

2019.sps,27605,c73f8d651e322faa77a3dcd68137cfc86ff198fbc257e89a2121aadc9eb404a6

2019_guide.pdf,929749,b60d9e9571939a47fab597448cda537e12e8c712cd20cd332d2ebe6c0fcd25b1

2021.dat,7289136,0a8605d7843fba9cb50e7c0c4094089b0ce2793a9988506971fdae3d9f2a04ae

2021.sps,28320,cf119914952250d8ca363c19b5a4a2368de655aed6ba55fe9742c2d36c8ca5e9

2021_guide.pdf,2215399,e758264daa61b0e8750401f98c7485ed98eb21983afa87b7fe5f32aa50e2cfe2

2023.dat,8503569,0a83cba11dc8a0cdf0043aacf0b80478f86cf93a91a8ecb81a276518e486241a

2023.sps,30337,e314d685a330946c385fb68c7e521794ccc44ba050f44be190fd3a0392d08288

2023_guide.pdf,1036445,54b081c797873c1a16d7b6d4be728591667fb7781e799ee2bec9fa6ae3f90a61
