# Supplementary material for: Temporal maintenance of an adolescent suicide attempt screening score in national surveys: An observational study
Source: Medicine (Baltimore). 2026 Aug 28;105(35):e50513. doi: 10.1097/MD.0000000000050513 (PMC13529099; doi:10.1097/MD.0000000000050513)
Supplement: Supplementary file 18 [file medi-105-e50513-s018.docx]

**Supplemental Digital Content 17**

**Penalty sensitivity data**

Temporal maintenance of an adolescent suicide attempt screening score in national surveys: An observational study

First author: Xiaotian Ma

*Source file: source_penalty_sensitivity.csv*

This Word document contains aggregate or metadata records only and does not include individual student records.

c_value,year,score_origin,n,effective_n,expected_events,prevalence,auroc,auprc,brier,scaled_brier,calibration_intercept,calibration_slope,observed_expected_ratio,sensitivity,false_negative_rate,positive_predictive_value,referral_fraction,weighted_log_loss,absolute_calibration_intercept_error,absolute_calibration_slope_error,capacity_threshold,top10_sensitivity,top10_positive_predictive_value,top10_referral_fraction,close_calibration

0.01,2015,cross_fitted_source,12567.0,5842.121655132331,1183.3445,0.08564627356459587,0.8617586097125484,0.3975919979507171,0.06311760331573124,0.19401346084230275,-0.008984287790785531,1.202404487073057,0.9931066132149554,0.4725528364732333,0.5274471635267667,0.40463457272115694,0.10002207481712824,0.21859967421190574,0.008984287790785531,0.20240448707305703,0.22179639419858477,0.4725528364732333,0.40463457272115694,0.10002207481712824,0.0

0.01,2017,frozen_source,10686.0,6834.4116150131185,925.1701,0.07371471546889397,0.8599535654628397,0.3839858555382577,0.055426059031248524,0.18826356138234102,-0.23263182563195006,1.1617715689518908,0.8361364528383513,0.4933823520669334,0.5066176479330666,0.34435222492018336,0.10561726356906125,0.19801820084547012,0.23263182563195006,0.16177156895189082,0.23076493759567937,0.4782675099422257,0.35257592359198137,0.09999364974849177,0.0

0.01,2019,frozen_source,10520.0,6240.351246070618,1017.866,0.08905635008953747,0.8556518311153537,0.38488645925394604,0.06601812184385566,0.1862204720780618,-0.0973188419413036,1.1654565771136225,0.9286835721758642,0.4774361261698495,0.5225638738301506,0.36278559700135976,0.11720068037159516,0.22707243874203686,0.0973188419413036,0.1654565771136225,0.24248210047590693,0.438229000673959,0.3901071631002042,0.10004193461421683,0.0

0.01,2021,frozen_source,15573.0,7465.749225286986,1579.6734000000001,0.10170608668544727,0.8542287499585487,0.4427816593540851,0.07209174304456548,0.21092165561911558,0.06895606122973316,1.2035696454599913,1.053245983276966,0.4876121228603329,0.512387877139667,0.42410441717404296,0.11693611013760338,0.2456419335404283,0.06895606122973316,0.20356964545999134,0.24253028048897926,0.4365401101265615,0.4437230274282529,0.1000596848433469,0.0

0.01,2023,frozen_source,19336.0,7383.468311248124,1831.6335,0.09462945164439508,0.8622017193391022,0.4325613426935111,0.06739258216723407,0.21339009538876264,-0.07881287714632827,1.2017569946754767,0.9428834031529164,0.515548716487223,0.484451283512777,0.3911760883803759,0.12471644813248016,0.2307890126745032,0.07881287714632827,0.2017569946754767,0.2582898093628987,0.4509737892433175,0.4245129723293702,0.10052791119179096,0.0

0.1,2015,cross_fitted_source,12567.0,5842.121655132331,1183.3445,0.08564627356459587,0.8591870643533408,0.389724580488319,0.06362473417496849,0.18753760268761233,-0.03305081367247834,0.9666431130334396,0.976907096496851,0.45925865206624106,0.5407413479337589,0.39333894638287975,0.09999973944478144,0.2182616761669894,0.03305081367247834,0.03335688696656036,0.24854573288567855,0.45925865206624106,0.39333894638287975,0.09999973944478144,1.0

0.1,2017,frozen_source,10686.0,6834.4116150131185,925.1701,0.07371471546889397,0.8601100889825326,0.3887092377679062,0.05554088835712368,0.18658184073209216,-0.2662781858554113,0.9956287943903915,0.8253491280078259,0.4938448616097731,0.506155138390227,0.34952867728606185,0.10415063433992919,0.19621229881790847,0.2662781858554113,0.004371205609608464,0.25898733758867104,0.47844672023015006,0.35265953467494576,0.1000074019870152,0.0

0.1,2019,frozen_source,10520.0,6240.351246070618,1017.866,0.08905635008953747,0.856241231794382,0.38783791718232474,0.06605088378196856,0.1858166285001669,-0.11529779595047002,0.9926402389184689,0.9210979291291886,0.4643839169399508,0.5356160830600492,0.38040845811395774,0.10871560766024285,0.22512481971956658,0.11529779595047002,0.007359761081531135,0.2621843106632929,0.4491809334431055,0.39978692301194746,0.10005933701102819,0.0

0.1,2021,frozen_source,15573.0,7465.749225286986,1579.6734000000001,0.10170608668544727,0.8515625000389302,0.44067224828804763,0.07186769785506224,0.2133739365563434,0.02965953518780984,1.0134040898681458,1.0211846163865483,0.4706577954658222,0.5293422045341778,0.421406265896917,0.11359290646271007,0.2448441581407855,0.02965953518780984,0.013404089868145785,0.27126045287083456,0.4314394988229845,0.436868966905357,0.10044206934094847,1.0

0.1,2023,frozen_source,19336.0,7383.468311248124,1831.6335,0.09462945164439508,0.8596242232474846,0.4287899260930158,0.06780345405296319,0.20859437626883315,-0.14762652942101953,1.008825598136005,0.9024759280882273,0.5071069075773074,0.49289309242269264,0.38144260930047724,0.12580463592446775,0.230451655791709,0.14762652942101953,0.00882559813600503,0.29107427049338147,0.44704167072724976,0.42301680641662437,0.10000384741557959,0.0

1.0,2015,cross_fitted_source,12567.0,5842.121655132331,1183.3445,0.08564627356459587,0.8562873307410075,0.3821638714130065,0.06428491014745412,0.1791074196745297,-0.051738807559186396,0.9151378106222865,0.9648055645447515,0.45521840850234235,0.5447815914976577,0.3901060487271734,0.09994144021462803,0.22023665402200854,0.051738807559186396,0.0848621893777135,0.25330050894423684,0.45521840850234235,0.3901060487271734,0.09994144021462803,1.0

1.0,2017,frozen_source,10686.0,6834.4116150131185,925.1701,0.07371471546889397,0.859283936129994,0.3874968888173291,0.05573247196663047,0.1837760234036243,-0.26972085335171436,0.9669754454631972,0.8249727562833736,0.49293324546480694,0.507066754535193,0.3498037849462761,0.10387661740188821,0.1965678913605477,0.26972085335171436,0.03302455453680275,0.2615981772153456,0.47949247387048066,0.3537452715969513,0.09991837098280856,0.0

1.0,2019,frozen_source,10520.0,6240.351246070618,1017.866,0.08905635008953747,0.855669848385636,0.386161257792599,0.0663507581520196,0.18212019460474038,-0.12188746512791841,0.9570520944275289,0.9179484126131461,0.46168031941336085,0.5383196805866391,0.38635145360892204,0.10642010990527889,0.22569057676003076,0.12188746512791841,0.04294790557247108,0.2658606983870302,0.44359640659968996,0.3949168812074517,0.1000339027387654,0.0

1.0,2021,frozen_source,15573.0,7465.749225286986,1579.6734000000001,0.10170608668544727,0.8473467887168831,0.4342143702176262,0.07224152942006382,0.20928217264648197,0.026439041693440926,0.9682297084122673,1.0186337363480582,0.46697342627912836,0.5330265737208717,0.4172841085892253,0.11381703447446283,0.24666978055802724,0.026439041693440926,0.0317702915877327,0.2770552500513167,0.42630666566899206,0.43368791279787233,0.09997507750077005,1.0

1.0,2023,frozen_source,19336.0,7383.468311248124,1831.6335,0.09462945164439508,0.8577217840296752,0.4236180608054384,0.06842870716356754,0.2012963877681122,-0.17029649082281467,0.9611464385208677,0.8903225609245672,0.5003407068062471,0.4996592931937529,0.38060374505884,0.12439963435757717,0.23214404842731512,0.17029649082281467,0.038853561479132326,0.3002117230443702,0.44148968666493604,0.41781161156932567,0.09999225918791076,0.0

10.0,2015,cross_fitted_source,12567.0,5842.121655132331,1183.3445,0.08564627356459587,0.855212078485741,0.3785061791451316,0.06450496433618132,0.17629741573454827,-0.05655733780552027,0.9026627672112976,0.9617474884136415,0.45261772881861545,0.5473822711813845,0.3875656736347334,0.10002181426190968,0.2210093361990481,0.05655733780552027,0.09733723278870243,0.2538131244442644,0.45261772881861545,0.3875656736347334,0.10002181426190968,1.0

10.0,2017,frozen_source,10686.0,6834.4116150131185,925.1701,0.07371471546889397,0.8586534494367964,0.3858178978641344,0.055876411139621406,0.181667977590817,-0.27518388911999553,0.9596024023414774,0.8221331036646115,0.48452938546111685,0.5154706145388832,0.3456211629846689,0.10334131589959557,0.19702347020545422,0.27518388911999553,0.04039759765852258,0.2615177609301892,0.4764220114765922,0.351176901386187,0.10000462126207624,0.0

10.0,2019,frozen_source,10520.0,6240.351246070618,1017.866,0.08905635008953747,0.8553904933930978,0.38426517755880557,0.066467346975643,0.18068304984509975,-0.12230943230782715,0.9483118975993832,0.9179520538649247,0.46035106782228696,0.539648932177713,0.3817233969963018,0.10740024369130086,0.22598012164099016,0.12230943230782715,0.05168810240061683,0.2670723821183726,0.4431378000640556,0.3948157288395993,0.09995608628967521,0.0

10.0,2021,frozen_source,15573.0,7465.749225286986,1579.6734000000001,0.10170608668544727,0.8460623351161262,0.4331983140643902,0.07225832018760629,0.20909838972636152,0.04064313777857603,0.9611434948640961,1.0287758313271733,0.4675009403842592,0.5324990596157408,0.4219554068116684,0.11268416140824941,0.24722964954493956,0.04064313777857603,0.038856505135903885,0.27557545676151585,0.4264339071608093,0.43371306210842764,0.09999911857962114,1.0

10.0,2023,frozen_source,19336.0,7383.468311248124,1831.6335,0.09462945164439508,0.8562729314406902,0.41816926590979103,0.06880825388243508,0.19686629770889696,-0.1730826248864421,0.9445424437330103,0.8890607847598703,0.49487809651876324,0.5051219034812368,0.3720194711610976,0.12588062328628363,0.23334576802073734,0.1730826248864421,0.05545755626698967,0.3037040222587816,0.4343219863580788,0.4111124042228804,0.09997181059000029,0.0
