# Supplementary material for: Temporal maintenance of an adolescent suicide attempt screening score in national surveys: An observational study
Source: Medicine (Baltimore). 2026 Aug 28;105(35):e50513. doi: 10.1097/MD.0000000000050513 (PMC13529099; doi:10.1097/MD.0000000000050513)
Supplement: Supplementary file 19 [file medi-105-e50513-s019.docx]

**Supplemental Digital Content 18**

**Single PSU sensitivity data**

Temporal maintenance of an adolescent suicide attempt screening score in national surveys: An observational study

First author: Xiaotian Ma

*Source file: single_psu_variance_sensitivity.csv*

This Word document contains aggregate or metadata records only and does not include individual student records.

year,scheme,metric,analytic_n,singleton_strata_in_original,bootstrap_replicates,point_estimate,ci_lower,ci_upper,bootstrap_standard_error

2017,certainty,calibration_intercept,10686,1,500,-0.2662781858554113,-0.4152366086791597,-0.1428485037072305,0.0691711183018307

2017,certainty,calibration_slope,10686,1,500,0.9956287943903916,0.9113623676092693,1.0720911400579305,0.0409656879149426

2017,certainty,auroc,10686,1,500,0.8601100889825326,0.8413924605417776,0.8788826329720354,0.009503707414774

2017,nearest_stratum_collapse,calibration_intercept,10686,1,500,-0.2662781858554113,-0.4134640966257198,-0.14066623586930807,0.07000845610329368

2017,nearest_stratum_collapse,calibration_slope,10686,1,500,0.9956287943903915,0.9163324748710998,1.0798818699713248,0.04075120356549334

2017,nearest_stratum_collapse,auroc,10686,1,500,0.8601100889825326,0.8411023346753363,0.8791842884725288,0.009702180516941841

2017,omit_singleton_strata,calibration_intercept,10524,1,500,-0.2639254341417361,-0.4252356078728424,-0.1440701962199378,0.07043253695355103

2017,omit_singleton_strata,calibration_slope,10524,1,500,0.9966833000829974,0.90942502914621,1.074003403028573,0.04348170219310503

2017,omit_singleton_strata,auroc,10524,1,500,0.8606151008982424,0.8381250768803924,0.8799057613453243,0.010386431219820151

2019,certainty,calibration_intercept,10520,3,500,-0.11529779595047,-0.2208969358657754,-0.0154230605082298,0.0546051001090495

2019,certainty,calibration_slope,10520,3,500,0.9926402389184688,0.8780000372229388,1.115340212672255,0.0598597475395673

2019,certainty,auroc,10520,3,500,0.856241231794382,0.8311586201244318,0.8799961073369122,0.0120281515703331

2019,nearest_stratum_collapse,calibration_intercept,10520,3,500,-0.11529779595047002,-0.21947608970466925,-0.018216259132505783,0.055368860552281046

2019,nearest_stratum_collapse,calibration_slope,10520,3,500,0.9926402389184689,0.8759080648839427,1.1288164516219823,0.0654898114336912

2019,nearest_stratum_collapse,auroc,10520,3,500,0.856241231794382,0.8334315890925021,0.8790224885752653,0.01274157991641503

2019,omit_singleton_strata,calibration_intercept,9977,3,500,-0.13372648068305196,-0.24069129473069673,-0.01741384944148578,0.06009241165402136

2019,omit_singleton_strata,calibration_slope,9977,3,500,0.9773327861110973,0.8708529172819175,1.110803669094344,0.0639599025813932

2019,omit_singleton_strata,auroc,9977,3,500,0.853748784072636,0.8305888655803477,0.8767953533222805,0.012885449616211657

2023,certainty,calibration_intercept,19336,0,500,-0.1476265294210195,-0.2849518120855318,-0.019178341763068,0.0667041448498653

2023,certainty,calibration_slope,19336,0,500,1.008825598136005,0.9432915816718704,1.087691634505536,0.0358916458008459

2023,certainty,auroc,19336,0,500,0.8596242232474842,0.8445490144165113,0.8745821166341191,0.0075567854696753
