# Supplementary material for: Temporal maintenance of an adolescent suicide attempt screening score in national surveys: An observational study
Source: Medicine (Baltimore). 2026 Aug 28;105(35):e50513. doi: 10.1097/MD.0000000000050513 (PMC13529099; doi:10.1097/MD.0000000000050513)
Supplement: Supplementary file 20 [file medi-105-e50513-s020.docx]

**Supplemental Digital Content 15**

**Learning curve summary**

Temporal maintenance of an adolescent suicide attempt screening score in national surveys: An observational study

First author: Xiaotian Ma

*Source file: maintenance_learning_curve_summary.csv*

This Word document contains aggregate or metadata records only and does not include individual student records.

target_year,budget,budget_label,strategy,runs,n_median,n_p05,n_p95,effective_n_median,effective_n_p05,effective_n_p95,expected_events_median,expected_events_p05,expected_events_p95,prevalence_median,prevalence_p05,prevalence_p95,auroc_median,auroc_p05,auroc_p95,auprc_median,auprc_p05,auprc_p95,brier_median,brier_p05,brier_p95,scaled_brier_median,scaled_brier_p05,scaled_brier_p95,calibration_intercept_median,calibration_intercept_p05,calibration_intercept_p95,calibration_slope_median,calibration_slope_p05,calibration_slope_p95,observed_expected_ratio_median,observed_expected_ratio_p05,observed_expected_ratio_p95,sensitivity_median,sensitivity_p05,sensitivity_p95,false_negative_rate_median,false_negative_rate_p05,false_negative_rate_p95,positive_predictive_value_median,positive_predictive_value_p05,positive_predictive_value_p95,referral_fraction_median,referral_fraction_p05,referral_fraction_p95,weighted_log_loss_median,weighted_log_loss_p05,weighted_log_loss_p95,absolute_calibration_intercept_error_median,absolute_calibration_intercept_error_p05,absolute_calibration_intercept_error_p95,absolute_calibration_slope_error_median,absolute_calibration_slope_error_p05,absolute_calibration_slope_error_p95,capacity_threshold_median,capacity_threshold_p05,capacity_threshold_p95,top10_sensitivity_median,top10_sensitivity_p05,top10_sensitivity_p95,top10_positive_predictive_value_median,top10_positive_predictive_value_p05,top10_positive_predictive_value_p95,top10_referral_fraction_median,top10_referral_fraction_p05,top10_referral_fraction_p95,close_calibration_median,close_calibration_p05,close_calibration_p95,close_calibration_rate

2017,-1,full_outer_training,intercept,50,10686.0,10686.0,10686.0,6834.4116150131185,6834.4116150131185,6834.4116150131185,925.1701,925.1701,925.1701,0.07371471546889397,0.07371471546889397,0.07371471546889397,0.8598993293055721,0.8594423932463302,0.8603477506529347,0.38768962432157283,0.3870332116491464,0.3885487683651473,0.055002797653780994,0.05496861718279869,0.05504998657949964,0.19446239076251032,0.19377129038166874,0.194962977206331,-0.0021295833266923563,-0.005624109617167823,0.0005983130212491673,0.9934526737213063,0.9913988194450037,0.9946809099830174,0.9984370501865326,0.9958798024567456,1.0004398522599747,0.413617236441169,0.4119786026375042,0.4153611914176647,0.586382763558831,0.5846388085823353,0.5880213973624958,0.3939567877477669,0.39172456244955134,0.3959710280573915,0.07739461450865945,0.07702772494311863,0.07770799623351204,0.19437897167456622,0.19428997013161553,0.19454237151504283,0.0021611816082804315,0.0003800138787146985,0.0056241096171678225,0.006547326278693699,0.005319090016982558,0.008601180554996295,0.21124767609511008,0.2098842005342322,0.21269863163352273,0.4799788168683791,0.4765439350017905,0.4858864818480407,0.35391310458096015,0.3512722856609693,0.3580200036230151,0.09999405211700016,0.09989039043609096,0.10011269784180686,1.0,1.0,1.0,1.0

2017,-1,full_outer_training,logistic,50,10686.0,10686.0,10686.0,6834.4116150131185,6834.4116150131185,6834.4116150131185,925.1701,925.1701,925.1701,0.07371471546889397,0.07371471546889397,0.07371471546889397,0.8598196684221462,0.8592498167327101,0.8602885559986706,0.3862749111743732,0.3851855335357512,0.3880508842343387,0.0550726164013767,0.05498925709042864,0.05511859329697039,0.19343986773791394,0.19276651801505387,0.19466069763953928,-0.002139337710922954,-0.00602504137814394,1.0477223723177897e-05,0.9948517406961429,0.9909911391535949,0.9990494420269489,0.9984279105045895,0.9955851968564259,1.0000078156343692,0.4133746324054355,0.41064850669082364,0.41536902241004114,0.5866253675945645,0.5846309775899589,0.5893514933091762,0.3947248427322486,0.3912411019894981,0.3972616582763842,0.07721220612358656,0.07679886211779374,0.07770784205864796,0.1945955491176567,0.1943561949506176,0.1947961892689252,0.002188917066104624,0.0004781148094969165,0.00602504137814394,0.0051482593038570945,0.0009505579730511527,0.009008860846405108,0.21100586014386002,0.209868206902342,0.21230180226372233,0.4821090197359383,0.47702804597770726,0.48507151819973426,0.35522233666690567,0.3517285169863721,0.35758042543942353,0.1000005497708332,0.09990324352043281,0.10010376167545927,1.0,1.0,1.0,1.0

2017,-1,full_outer_training,refit,50,10686.0,10686.0,10686.0,6834.4116150131185,6834.4116150131185,6834.4116150131185,925.1701,925.1701,925.1701,0.07371471546889397,0.07371471546889397,0.07371471546889397,0.8641687959595158,0.8623393675636294,0.8659921534233226,0.3841519885698407,0.37988783511182844,0.3887427140224491,0.05504914234601828,0.05477262568697673,0.05522069762597117,0.19378365451311685,0.1912711599499277,0.1978333497639067,-0.00849547675782324,-0.014336813257559667,-0.0035500769018750817,1.0081451003408777,1.0000833319572737,1.0157386057747442,0.9938005620975727,0.9895698610710306,0.997397760622953,0.4028372187990079,0.3947662867617533,0.4107175318354971,0.5971627812009921,0.5892824681645029,0.6052337132382467,0.39405670569594975,0.38216865256368593,0.40522410392781394,0.07511994565395796,0.07393443178076986,0.07742100589896136,0.19403548824479785,0.19326676702897788,0.19462931904969386,0.00849547675782324,0.0035500769018750817,0.014336813257559667,0.008145100340877565,0.00198136480946669,0.015738605774744195,0.20033495344478536,0.19791867948439906,0.20363210183185815,0.4937769281562385,0.48794264427698214,0.501065928308751,0.3638501704900321,0.35992854825798987,0.3695428960161098,0.09999953388994574,0.09987307624030689,0.10015030694741417,1.0,1.0,1.0,1.0

2017,0,none,static,1,10686.0,10686.0,10686.0,6834.4116150131185,6834.4116150131185,6834.4116150131185,925.1701,925.1701,925.1701,0.07371471546889397,0.07371471546889397,0.07371471546889397,0.8601100889825326,0.8601100889825326,0.8601100889825326,0.3887092377679062,0.3887092377679062,0.3887092377679062,0.05554088835712368,0.05554088835712368,0.05554088835712368,0.18658184073209216,0.18658184073209216,0.18658184073209216,-0.2662781858554113,-0.2662781858554113,-0.2662781858554113,0.9956287943903915,0.9956287943903915,0.9956287943903915,0.8253491280078259,0.8253491280078259,0.8253491280078259,0.4938448616097731,0.4938448616097731,0.4938448616097731,0.506155138390227,0.506155138390227,0.506155138390227,0.34952867728606185,0.34952867728606185,0.34952867728606185,0.10415063433992919,0.10415063433992919,0.10415063433992919,0.19621229881790847,0.19621229881790847,0.19621229881790847,0.2662781858554113,0.2662781858554113,0.2662781858554113,0.004371205609608464,0.004371205609608464,0.004371205609608464,0.2589873375886714,0.2589873375886714,0.2589873375886714,0.47844672023015006,0.47844672023015006,0.47844672023015006,0.35265953467494576,0.35265953467494576,0.35265953467494576,0.1000074019870152,0.1000074019870152,0.1000074019870152,0.0,0.0,0.0,0.0

2017,250,250,intercept,50,10686.0,10686.0,10686.0,6834.4116150131185,6834.4116150131185,6834.4116150131185,925.1701,925.1701,925.1701,0.07371471546889397,0.07371471546889397,0.07371471546889397,0.8552427844736851,0.8464147152209851,0.8597247407141991,0.37725498726605916,0.35829709103675844,0.3886395075631419,0.05584334537909884,0.05507815731294344,0.05694378789647727,0.18215223866143804,0.16603582509040257,0.1933587189082751,0.03254900205242037,-0.1914875288296733,0.3130666426493744,0.9549694261787837,0.907049475370872,0.991330676279061,1.0239635939359166,0.8715249345372543,1.2613848090239006,0.4047023352786693,0.3250278462306553,0.47521448218008766,0.5952976647213307,0.5247855178199123,0.6749721537693447,0.3866106099060639,0.35343795532949135,0.43427602266639126,0.0762272199666568,0.05565488900843969,0.0958252609977273,0.19762127957835302,0.1949782617275883,0.20278328325726283,0.11429913347274137,0.012180211608101975,0.3130666426493744,0.045030573821216335,0.008669323720938971,0.09295052462912798,0.20482538959397995,0.16220718940280573,0.2424204306486489,0.47814466766705926,0.46087462186683287,0.4920037353131062,0.35241956827813126,0.3396554224337961,0.3626078271139051,0.1000154413894892,0.09992540366724235,0.10011646855263026,0.0,0.0,1.0,0.36

2017,250,250,logistic,50,10686.0,10686.0,10686.0,6834.4116150131185,6834.4116150131185,6834.4116150131185,925.1701,925.1701,925.1701,0.07371471546889397,0.07371471546889397,0.07371471546889397,0.8496540213824462,0.8377234091835187,0.8577114313294115,0.36494594609200615,0.33550946122269476,0.38234769263582485,0.05644718555703242,0.055482643087975625,0.05945024448466866,0.17330876171039383,0.12932778233922718,0.18743486561192194,0.0334125494545902,-0.1793805036536303,0.2808554318010032,0.8868063733592129,0.7217902917362117,1.0619315903999222,1.0232333719949869,0.8762377146102318,1.246648889836197,0.39245053423148885,0.26642914638075743,0.46162326798066644,0.6075494657685112,0.5383767320193336,0.7335708536192426,0.38830806991010147,0.34751294877266237,0.432443769351651,0.07434046622478901,0.046958762167862725,0.09693255801835181,0.2020169193615321,0.19701540241551407,0.2125610781483049,0.11745379687325824,0.014170730294151277,0.28372075280133596,0.11445723503469074,0.020345783695040143,0.2782097082637883,0.20239104851559292,0.15111271181250394,0.24243754278885893,0.4762173464101358,0.4525900804619604,0.4912179392740859,0.35109877483433133,0.33352032490972916,0.36225653110716266,0.10001816634057553,0.09993076074578883,0.10019796291580602,0.0,0.0,1.0,0.14

2017,250,250,refit,50,10686.0,10686.0,10686.0,6834.4116150131185,6834.4116150131185,6834.4116150131185,925.1701,925.1701,925.1701,0.07371471546889397,0.07371471546889397,0.07371471546889397,0.8199143991914186,0.7848900087989762,0.8371608824035125,0.29779952891416495,0.24891498724861025,0.33559836394155307,0.06023458019097541,0.058816100194964006,0.06304428881839437,0.11784087736986681,0.07669158920762319,0.1386150749285193,0.09212676991250426,-0.15284030627762424,0.2529758950243238,1.3315269005824346,1.1156302472087012,1.6331600863630091,1.0824303807206928,0.8782681129122976,1.246632261141426,0.17829526700009002,0.08244733049630548,0.26515598591005046,0.82170473299991,0.7348440140899496,0.9175526695036945,0.3972867900378366,0.3412197120355297,0.5334733946052556,0.031598046767809626,0.01554494599656879,0.05224046713310604,0.2182138274693402,0.2130758985509892,0.2302955055535474,0.11826194184996122,0.022366352342955392,0.25358953899307146,0.3315269005824346,0.11563024720870124,0.633160086363009,0.14482328973828326,0.11318082031639978,0.17580226573863317,0.40694894917161717,0.3479638933424243,0.44704989385195215,0.2996422232453226,0.2564988487848556,0.32915050097834936,0.10000650562152627,0.09992382208013523,0.10015077226069909,0.0,0.0,0.0,0.02

2017,500,500,intercept,50,10686.0,10686.0,10686.0,6834.4116150131185,6834.4116150131185,6834.4116150131185,925.1701,925.1701,925.1701,0.07371471546889397,0.07371471546889397,0.07371471546889397,0.8582491839424683,0.8521958746633166,0.860659473469268,0.3839783636224153,0.3693312558897873,0.3912172098110861,0.0553527643977872,0.05493519652398574,0.056227377924452406,0.18933699012282107,0.17652793095984168,0.19545243590202835,-0.022047883494756094,-0.18899009252746324,0.14746942745474553,0.976881990094479,0.9389854051364754,0.9937672624073344,0.9840037006100679,0.8719742853976363,1.115513070080029,0.41886546052450246,0.3721672911824539,0.4722020145268421,0.5811345394754976,0.5277979854731579,0.627832708817546,0.3875532663137438,0.35561005715724125,0.41595912246319344,0.07979153566451039,0.06652050995786365,0.09812265172161132,0.1957946568993449,0.1943964197114967,0.19874036853738528,0.07933746962526814,0.007892749704919471,0.2142953148848349,0.023118009905521042,0.0062327375926656105,0.06101459486352457,0.2109670080915108,0.1817141585375943,0.24495217463891172,0.4801492179654314,0.4676675186541372,0.4912406864424174,0.35358268011003396,0.3450277734248386,0.36160163349032115,0.10000372091274068,0.09990964038294645,0.10024790242507896,1.0,0.0,1.0,0.68

2017,500,500,logistic,50,10686.0,10686.0,10686.0,6834.4116150131185,6834.4116150131185,6834.4116150131185,925.1701,925.1701,925.1701,0.07371471546889397,0.07371471546889397,0.07371471546889397,0.8554638072186658,0.8484032625544518,0.8596485205176984,0.37679184831931745,0.3494138812014196,0.3891903774670155,0.055749494234778235,0.055065371894087285,0.05698339840951852,0.18352672559019562,0.16545571354449978,0.19354596639709043,-0.01977809469482235,-0.1909017404845327,0.15031587662757373,0.9465487632329822,0.8297322008355247,1.0363625235578546,0.9854979371033028,0.870572519169657,1.1180850902477177,0.4125416504489282,0.36618366179365286,0.47449697628576615,0.5874583495510718,0.5255030237142339,0.6338163382063471,0.3854738139438652,0.3540592771959363,0.4162469073308235,0.07938997189316534,0.06424814496166105,0.09636556143075548,0.19750892368513617,0.19501416734963117,0.20169732895058903,0.08706295787253054,0.005944642706216764,0.22564266895440596,0.06290398324523422,0.008092313253452887,0.17026779916447526,0.20930660134754125,0.18027406605127963,0.23994898801834091,0.47902980219529356,0.4643578083641052,0.49404860252184973,0.3531657842353491,0.34246680028056253,0.36409582055982354,0.10000297593342322,0.09994988480309121,0.10014135524406478,0.0,0.0,1.0,0.44

2017,500,500,refit,50,10686.0,10686.0,10686.0,6834.4116150131185,6834.4116150131185,6834.4116150131185,925.1701,925.1701,925.1701,0.07371471546889397,0.07371471546889397,0.07371471546889397,0.8403460986923897,0.8250750663899109,0.8490211623429075,0.33796377443181935,0.3014125385543051,0.3541307611381145,0.05789406469303326,0.05699063157439965,0.0593871776626286,0.15211864757463406,0.1302514207579152,0.16534978099935466,-0.0037765778677269282,-0.152953553575251,0.14989212336868737,1.205886317345365,1.105298426990052,1.333625174823298,0.9969026534989999,0.8848339696981948,1.1320928270407185,0.2830968056576839,0.2251136953085708,0.34849287174326105,0.7169031943423161,0.6515071282567388,0.7748863046914292,0.4039481099361805,0.3459343752588005,0.4597593308081077,0.05126128982292917,0.03744873108111079,0.07063659996538833,0.20778506069543407,0.20427722642031135,0.21310160363743785,0.07761449185358743,0.009813935090644764,0.19413068229750435,0.20588631734536522,0.10529842699005208,0.333625174823298,0.16971434081332704,0.14388362819024395,0.2023729643617221,0.44563767246693337,0.40959183613910566,0.466688190636511,0.32828549383294825,0.3019313655014421,0.34359740914391224,0.10001429404166337,0.09994402974371755,0.10032494722996771,0.0,0.0,0.0,0.02

2017,1000,1000,intercept,50,10686.0,10686.0,10686.0,6834.4116150131185,6834.4116150131185,6834.4116150131185,925.1701,925.1701,925.1701,0.07371471546889397,0.07371471546889397,0.07371471546889397,0.8593884427048444,0.8556288707442063,0.860871448194844,0.3854352940832053,0.37514521554552205,0.39095457216615753,0.05515459929640085,0.05496074265853209,0.05564396238674987,0.19223919598896383,0.18507228101983542,0.1950783027119813,-0.004747257707078005,-0.13299184305773576,0.08056501789474228,0.9878177279958086,0.9620312515671955,0.9960817675649573,0.996528474186315,0.9079988130857461,1.0612820530903602,0.41733314770981034,0.3885964862029155,0.44838951777624453,0.5826668522901897,0.5516104822237554,0.6114035137970845,0.391954101962412,0.36631636477949114,0.4102313378231745,0.07810635659379202,0.07055664774923821,0.09050495654260754,0.19489582016781704,0.19433225280111208,0.19685332415764786,0.04056792035326189,0.005187311901018184,0.13388582703630925,0.012182272004191419,0.003918232435042723,0.037968748432804456,0.21023677679483405,0.19541066401054927,0.23214876699962264,0.4795059848994255,0.47003284044739446,0.4908257303170519,0.3534680414024932,0.3464095230082239,0.3616190984717705,0.10001280009918184,0.09992195763991826,0.10015539392115998,1.0,0.0,1.0,0.88

2017,1000,1000,logistic,50,10686.0,10686.0,10686.0,6834.4116150131185,6834.4116150131185,6834.4116150131185,925.1701,925.1701,925.1701,0.07371471546889397,0.07371471546889397,0.07371471546889397,0.8574686126993009,0.8537171323847186,0.8604330226520889,0.37995277039651437,0.3684552421628429,0.3891565772214586,0.05539812839977763,0.055003915871592805,0.0559589771976116,0.18867261646788736,0.18045876519081808,0.1944460140227596,-0.010146531433864407,-0.1303931798945989,0.07430309026068987,0.9876066372466956,0.9045919958462485,1.0499888299837334,0.9924834389698549,0.9087519267504055,1.0560573162992672,0.40926165901816325,0.3814111372600563,0.452107309780115,0.5907383409818368,0.547892690219885,0.6185888627399436,0.3905755951794794,0.36428807592768875,0.4108123654909641,0.0777033187991985,0.06945725946091862,0.09136387626363629,0.1958688720989315,0.19467471749779067,0.19737290579801864,0.04158349338677862,0.004916364920445924,0.13137455456630476,0.032948946331476436,0.00200990415759732,0.09540800415375146,0.20713885116412922,0.19465027377155017,0.23099337836260847,0.48171330872020185,0.4691494677573345,0.49134455382853376,0.3549549994203568,0.3458012522544802,0.3618485930798495,0.1000017409409718,0.0999066062853626,0.10018626753729118,1.0,0.0,1.0,0.84

2017,1000,1000,refit,50,10686.0,10686.0,10686.0,6834.4116150131185,6834.4116150131185,6834.4116150131185,925.1701,925.1701,925.1701,0.07371471546889397,0.07371471546889397,0.07371471546889397,0.8471242152396208,0.8414982625302615,0.8543940980224207,0.34811052979915214,0.33456775504138836,0.37135456143588047,0.05700308810509142,0.056054599869310655,0.05777699456716887,0.16516735020708834,0.15383318562223544,0.17905833354670037,-0.008837644207845437,-0.1347456590331527,0.07977854114004636,1.117333154389335,1.0364571188356348,1.168859327084952,0.9931082209586959,0.9002517931689841,1.0639491023575602,0.3389849607115491,0.30117191422420586,0.37582573193837543,0.6610150392884508,0.6241742680616245,0.6988280857757941,0.395826373364894,0.35399513944687094,0.42662168442234444,0.06259552865803968,0.05446677089471777,0.0760622381218426,0.20315914708406127,0.19976409586442928,0.20548430944024562,0.04345969141545991,0.002675275959360499,0.1347456590331527,0.11733315438933489,0.03645711883563477,0.16885932708495205,0.185192837117219,0.16950624540717737,0.20930547932630675,0.4570708672924038,0.43048271879949424,0.47880943190879166,0.3367870609280075,0.3173285776680079,0.3530835464992189,0.10000889592949674,0.09995073455757471,0.1002721485139774,0.0,0.0,1.0,0.22

2017,2000,2000,intercept,50,10686.0,10686.0,10686.0,6834.4116150131185,6834.4116150131185,6834.4116150131185,925.1701,925.1701,925.1701,0.07371471546889397,0.07371471546889397,0.07371471546889397,0.8595792290703529,0.857534657395066,0.8607064482560632,0.3866905605032469,0.3812473984085271,0.39050754883972943,0.055095689406162565,0.05492018407535188,0.05541241071127542,0.19310195450611584,0.18846344639747142,0.19567229911803133,-0.014432054723381797,-0.08948986992460507,0.06219740926054191,0.9889523975825024,0.9740092762866335,0.9962490089554877,0.9894643519087352,0.9368277595328008,1.0469092909481004,0.415568931594309,0.3960449975631508,0.4399985635074025,0.584431068405691,0.5600014364925975,0.6039550024368492,0.3926671205608305,0.37701846209891593,0.40850393401350377,0.07837362489566305,0.07230797500757727,0.08592513005665667,0.1947327399388959,0.19423603754665814,0.19589327710422064,0.03595773888426626,0.006867228303235561,0.08948986992460507,0.011047602417497582,0.003750991044512225,0.025990723713366526,0.210102446171061,0.1988317403746862,0.22374343382958967,0.4821028587067394,0.47493917604989605,0.4908283730743136,0.3555559818510001,0.34998400048416295,0.36189480226911297,0.09999988845229471,0.09990503186917939,0.1000776551301905,1.0,1.0,1.0,1.0

2017,2000,2000,logistic,50,10686.0,10686.0,10686.0,6834.4116150131185,6834.4116150131185,6834.4116150131185,925.1701,925.1701,925.1701,0.07371471546889397,0.07371471546889397,0.07371471546889397,0.8588771305210818,0.8563290354652635,0.8602066183035195,0.38434699394914784,0.37145354091676647,0.3896903914385123,0.05519731857112045,0.054949157701820184,0.05589542053666061,0.191613555405323,0.1813895774930547,0.1952479689604431,-0.012435603971326727,-0.08975970565607837,0.06383635754642586,0.9832686068083782,0.9265849080078449,1.0409467667788614,0.9908489064300632,0.9369879465797559,1.04800999221104,0.4135202812974609,0.39679725922833,0.4411632250112709,0.5864797187025391,0.5588367749887291,0.6032027407716699,0.39242982022748407,0.3768163665260346,0.40963175920362294,0.07747996045474492,0.07182670960403433,0.08585614058962841,0.19508031955688335,0.1944394709343113,0.19721234578846555,0.03504185033365367,0.006956485466228499,0.08975970565607835,0.03324506553623241,0.004398318371132731,0.07341509199215512,0.2085637469159724,0.19870211724939446,0.22291966832560123,0.4816287837231229,0.47480540605451904,0.4896216274174878,0.35497010779374677,0.3499717814240475,0.3610334144915097,0.10001380801237605,0.09990038949271608,0.10016697536166155,1.0,1.0,1.0,1.0

2017,2000,2000,refit,50,10686.0,10686.0,10686.0,6834.4116150131185,6834.4116150131185,6834.4116150131185,925.1701,925.1701,925.1701,0.07371471546889397,0.07371471546889397,0.07371471546889397,0.8543031727327396,0.8503225424421094,0.8590749902968052,0.3659314138580999,0.34891665325022053,0.3749453703271542,0.05597911303763882,0.05561734182200005,0.056941751964744165,0.180163867892364,0.16606564211155095,0.1854621496628833,-0.009014120725087409,-0.08341120699687764,0.04945158912233158,1.0422514951065875,0.9957621327884082,1.1113402741279275,0.993228955787762,0.9394785079001315,1.0379853083330162,0.36754468178338234,0.34422808303035296,0.40356079384753135,0.6324553182166177,0.5964392061524687,0.655771916969647,0.39181299801843184,0.3727426824744267,0.4076088515024031,0.06946292329565464,0.06523911684813354,0.07685176919441203,0.1985868062496127,0.19669562694953363,0.20086306766444123,0.03127883266214271,0.002714029272649158,0.0850810258562412,0.042251495106587544,0.013334594023049018,0.11134027412792752,0.19445777047376492,0.18145737578350124,0.20603235084829585,0.4733665733468905,0.45319418018373053,0.4868342210799938,0.34849572149744235,0.33407890874558727,0.35871104206729804,0.10000837006174323,0.09994658936516926,0.10031439202835478,1.0,0.0,1.0,0.92

2017,4000,4000,intercept,50,10686.0,10686.0,10686.0,6834.4116150131185,6834.4116150131185,6834.4116150131185,925.1701,925.1701,925.1701,0.07371471546889397,0.07371471546889397,0.07371471546889397,0.8599112410081312,0.8590997442776548,0.8606881259757224,0.38760045925393005,0.3843708747141503,0.3897110280742275,0.055018087888059244,0.05492123153057802,0.0551881928991485,0.19423845926646688,0.19174720446130816,0.1956569587241852,0.0021989808857684904,-0.04338959751743275,0.0380984641076953,0.9921760037157571,0.9866430852684623,0.9967288033787357,1.0016174298601732,0.9687391708751038,1.0284455815867453,0.4129679504341959,0.4048937271102903,0.42644155382885807,0.5870320495658041,0.5735584461711419,0.5951062728897096,0.39506278660145044,0.3808262187999361,0.4041820684647229,0.07694504140172759,0.07406907025943765,0.08144925089341744,0.19445016582549926,0.19413970904645772,0.19497422033955272,0.020001846398767717,0.00148800152381845,0.04937725392939108,0.007823996284242907,0.0032711966212642385,0.01335691473153765,0.2095216997534496,0.20303960230560236,0.2170817824447394,0.48091821168885585,0.47644276982146305,0.4864225994765719,0.35451323875570206,0.35126813365917947,0.35834039894203984,0.09999137098822661,0.0998608087814178,0.10009636287552136,1.0,1.0,1.0,1.0

2017,4000,4000,logistic,50,10686.0,10686.0,10686.0,6834.4116150131185,6834.4116150131185,6834.4116150131185,925.1701,925.1701,925.1701,0.07371471546889397,0.07371471546889397,0.07371471546889397,0.8595760390522889,0.8580028430826746,0.86042629546403,0.3859891376938194,0.38108499211235963,0.38944548954000247,0.055084136441100925,0.05494492360417402,0.05531558406613658,0.19327115222427438,0.18988151070624695,0.19530997898612656,0.002746358131322982,-0.045165958020486556,0.03631794234125317,0.9940790366665448,0.9685107402522578,1.0232180370532118,1.0020357976408134,0.9672587438377,1.0270938140029944,0.4114624435009303,0.4000622588213778,0.42148888080148716,0.5885375564990697,0.5785111191985128,0.5999377411786222,0.3958476146975469,0.3820981069838606,0.4059316362814743,0.07590581118925471,0.07289968020866432,0.08097443573593278,0.19473694238459321,0.19429355727574177,0.1952585161769933,0.021229185306648406,0.0019910155245363844,0.04972734697368982,0.013183524392161194,0.001885808969534825,0.034216448524359316,0.2081695047733827,0.20100379772756857,0.2148585003445408,0.48179929290840673,0.4760757454223823,0.4888001892841111,0.35529928042919234,0.35044404556817443,0.3601253105529449,0.1000148716994229,0.09991686110494055,0.10015106228473283,1.0,1.0,1.0,1.0

2017,4000,4000,refit,50,10686.0,10686.0,10686.0,6834.4116150131185,6834.4116150131185,6834.4116150131185,925.1701,925.1701,925.1701,0.07371471546889397,0.07371471546889397,0.07371471546889397,0.8593823826196882,0.8564148516044318,0.8631042961278902,0.37546301155402323,0.3668615520110084,0.38651975489546453,0.05553869867137804,0.0550057511489969,0.05603095877508551,0.1866139095412394,0.17940456667181806,0.19441913566319538,-0.0014307489444171306,-0.04609850975597334,0.042333782238673755,1.022898645434567,0.9869879901317867,1.05737625947022,0.9989443047223641,0.9665306490358565,1.03203536970157,0.3868287572198885,0.36759733156097457,0.41407087194019776,0.6131712427801115,0.5859291280598022,0.6324026684390254,0.39457979792347464,0.3829575106484571,0.4148762402998702,0.07216563256632069,0.06781123323637181,0.07637200968489047,0.1962212381416668,0.19466887580992737,0.19762062796501703,0.01834214129458165,0.0014136945632420013,0.04763214341533109,0.02289864543456699,0.0019601012443224543,0.057376259470220015,0.19613638710089504,0.18872869939956669,0.2044960415305086,0.48602484018884745,0.4673301482613846,0.49842658123084604,0.3579718197017002,0.3443769520051631,0.36743439467078887,0.10001134201131986,0.09992555824049111,0.10018484092181748,1.0,1.0,1.0,1.0

2019,-1,full_outer_training,intercept,50,10520.0,10520.0,10520.0,6240.351246070618,6240.351246070618,6240.351246070618,1017.866,1017.866,1017.866,0.08905635008953747,0.08905635008953747,0.08905635008953747,0.8555808367599687,0.8552646821510729,0.8558092849244703,0.3872500122787898,0.38583446381188075,0.3880617406704364,0.0660049732896397,0.06597487147618816,0.06605506856299138,0.18638254915544405,0.18576504434372476,0.18675360241863936,0.0023809277271333116,0.0015753623305542024,0.004330804080830625,0.9898720418676983,0.9878197079445623,0.990925277985398,1.0017131875211516,1.001133102880077,1.0031175430839039,0.4295520235473039,0.42535579339520135,0.4340342343687676,0.5704479764526962,0.5659657656312325,0.5746442066047986,0.4059338785408366,0.40348616231883905,0.4079818190229704,0.09423551861386698,0.09359610958351391,0.09497268991734788,0.2249378031347258,0.2248884424787512,0.22510074409758807,0.0023809277271333116,0.0015753623305542024,0.004330804080830625,0.010127958132301718,0.009074722014602021,0.012180292055437652,0.23961031696096624,0.23883870267841942,0.240895177922938,0.4446959619439101,0.44040283298587435,0.44854385547802955,0.39532163487319577,0.3920937917230501,0.39895149845492744,0.10003059549593249,0.09996779025458953,0.10043996741368387,1.0,1.0,1.0,1.0

2019,-1,full_outer_training,logistic,50,10520.0,10520.0,10520.0,6240.351246070618,6240.351246070618,6240.351246070618,1017.866,1017.866,1017.866,0.08905635008953747,0.08905635008953747,0.08905635008953747,0.854752839637696,0.8541549602033153,0.8552703508216961,0.3861287930237489,0.3814059560576547,0.38803286168572726,0.0660753515769115,0.06601101263508091,0.06624421024050597,0.18551502357621819,0.18343356897394325,0.18630810450987045,0.0025866637367880346,0.00120181533146474,0.004949650245105494,0.9950686591209107,0.9906951790837331,0.9978097768826721,1.001866862211993,1.0008674669357567,1.0035794274941214,0.4275935633963606,0.4208250398382498,0.4305428219431634,0.5724064366036393,0.5694571780568366,0.5791749601617502,0.40599547737873415,0.402252767715006,0.4082585117888028,0.0938231719274299,0.09292004965308905,0.09430217705558987,0.22532415920164817,0.22518149344184388,0.22570731587236664,0.0025866637367880346,0.00120181533146474,0.004949650245105494,0.004931340879089308,0.002190223117327883,0.009304820916266953,0.23870172147515595,0.237598614536208,0.24010805612430391,0.44307821461764124,0.4376637985746651,0.4474090597387082,0.3945473518719962,0.3903935702062058,0.39829798040149983,0.10001154385236447,0.09989085704931941,0.10018199372339556,1.0,1.0,1.0,1.0

2019,-1,full_outer_training,refit,50,10520.0,10520.0,10520.0,6240.351246070618,6240.351246070618,6240.351246070618,1017.866,1017.866,1017.866,0.08905635008953747,0.08905635008953747,0.08905635008953747,0.8527693884412197,0.8508680056808702,0.8544137982695359,0.3763445232626056,0.3717772639616104,0.3810203769888482,0.06649632634181812,0.06622997164067064,0.0667185407448948,0.18032583254980533,0.17758668264698174,0.18360908261669232,-0.008096541392664967,-0.017981070738270336,-0.0021281886444863727,0.9832643734430838,0.9715563818451707,0.9957299212305973,0.9941840219988302,0.9871567251646503,0.9984633413894016,0.4212631132192254,0.4147583670149116,0.42588867296874044,0.5787368867807747,0.5741113270312596,0.5852416329850884,0.39955994445456644,0.3928739387375938,0.4037084820659068,0.09407512608578947,0.09176516570542122,0.09502776950942765,0.22636656681530787,0.22537019763945684,0.22719708953504802,0.008096541392664967,0.0021281886444863727,0.017981070738270336,0.016735626556916183,0.004270078769402635,0.02844361815482932,0.23912432822505728,0.2351778511412448,0.24088514982859294,0.43724272153701965,0.43355284487349016,0.44388685249335375,0.38939224722962845,0.3860951275403786,0.39514398092866465,0.10000283827929896,0.09991122677789478,0.10008598393899638,1.0,1.0,1.0,1.0

2019,0,none,static,1,10520.0,10520.0,10520.0,6240.351246070618,6240.351246070618,6240.351246070618,1017.866,1017.866,1017.866,0.08905635008953747,0.08905635008953747,0.08905635008953747,0.856241231794382,0.856241231794382,0.856241231794382,0.38783791718232474,0.38783791718232474,0.38783791718232474,0.06605088378196856,0.06605088378196856,0.06605088378196856,0.1858166285001669,0.1858166285001669,0.1858166285001669,-0.11529779595047002,-0.11529779595047002,-0.11529779595047002,0.9926402389184689,0.9926402389184689,0.9926402389184689,0.9210979291291886,0.9210979291291886,0.9210979291291886,0.4643839169399508,0.4643839169399508,0.4643839169399508,0.5356160830600492,0.5356160830600492,0.5356160830600492,0.38040845811395774,0.38040845811395774,0.38040845811395774,0.10871560766024285,0.10871560766024285,0.10871560766024285,0.22512481971956658,0.22512481971956658,0.22512481971956658,0.11529779595047002,0.11529779595047002,0.11529779595047002,0.007359761081531135,0.007359761081531135,0.007359761081531135,0.2621843106632928,0.2621843106632928,0.2621843106632928,0.4491809334431055,0.4491809334431055,0.4491809334431055,0.39978692301194746,0.39978692301194746,0.39978692301194746,0.10005933701102819,0.10005933701102819,0.10005933701102819,0.0,0.0,0.0,0.0

2019,250,250,intercept,50,10520.0,10520.0,10520.0,6240.351246070618,6240.351246070618,6240.351246070618,1017.866,1017.866,1017.866,0.08905635008953747,0.08905635008953747,0.08905635008953747,0.8519814292673349,0.8402458403814945,0.8561412786792261,0.3801331984409875,0.36434223276071165,0.39018171912415495,0.06674207811526017,0.06596642637799259,0.06784788551542494,0.17729654670234274,0.16366569203781534,0.18685770183605552,0.007387085679122831,-0.21301763698076706,0.2735845712274144,0.9575575652561822,0.8894289747465955,0.9919451808923533,1.0052524327023669,0.8604234343522487,1.220796833504569,0.4271740091524818,0.35221860244865233,0.49744017876616375,0.5728259908475182,0.5025598212338362,0.6477813975513476,0.3896174867451153,0.3536491656134731,0.4430343313954472,0.09879709016176538,0.0705978373379159,0.12424456602285176,0.22804155729575243,0.2249624821637631,0.23330829988091983,0.08419974605053057,0.010543907267325249,0.28457743321125434,0.042442434743817814,0.008054819107646749,0.11057102525340434,0.24526957369044447,0.1968631668133802,0.28535377605601525,0.4357415416174624,0.41780642540373686,0.447888474514327,0.38816356530989093,0.3719539123221503,0.3990006652982872,0.10000175773832046,0.09991795762951218,0.1002173326629838,1.0,0.0,1.0,0.56

2019,250,250,logistic,50,10520.0,10520.0,10520.0,6240.351246070618,6240.351246070618,6240.351246070618,1017.866,1017.866,1017.866,0.08905635008953747,0.08905635008953747,0.08905635008953747,0.847741035961441,0.8300951913155783,0.8540501390282457,0.371764497622483,0.35160951284187075,0.3880834237409735,0.06761209796781972,0.06615063817482704,0.06922059067230056,0.1665721527764874,0.14674489327319207,0.1845869951743278,0.00787034793836022,-0.22368396470013535,0.2590733552210652,0.9102853686827471,0.800935247986008,1.0137550189736169,1.0058407601425954,0.8609220223254703,1.207033622089949,0.41760845730184526,0.3392927998380927,0.5033148272955379,0.5823915426981547,0.4966851727044621,0.6607072001619073,0.39317867621528435,0.35219851782920913,0.433795316063134,0.09417263725344296,0.07177105198521144,0.12495524788611842,0.23240221991261095,0.22654786865185314,0.23696510428416548,0.09222727661944832,0.011799312088318912,0.27785075909220264,0.09266239231194495,0.010557483871357704,0.19906475201399187,0.23942356864939757,0.20268147668214523,0.2901488291024208,0.43394400638197955,0.4079884778546488,0.4487501350865438,0.38653758505490754,0.3631025778250917,0.39961875947021386,0.10000889280850633,0.09991156625149773,0.10013199267391466,0.0,0.0,1.0,0.32

2019,250,250,refit,50,10520.0,10520.0,10520.0,6240.351246070618,6240.351246070618,6240.351246070618,1017.866,1017.866,1017.866,0.08905635008953747,0.08905635008953747,0.08905635008953747,0.8130489608697598,0.7855430929962949,0.833541897594497,0.31364415158614756,0.2704120906079506,0.33643136397519185,0.07123641336499976,0.069840790261192,0.07351592063543386,0.12189663656090627,0.09379804334712309,0.13909993588014227,0.03871286211863892,-0.18430272075978327,0.2814778873847355,1.3588203104496863,1.1195598507622024,1.5327957466160205,1.0329623424532977,0.8612485921141134,1.2747088540808262,0.22599502292050233,0.12972148102009498,0.3325535925161072,0.7740049770794977,0.6674464074838927,0.8702785189799049,0.39982986621893757,0.3427425103629872,0.4778522569855635,0.05047598880020588,0.024174266114284783,0.08270636116923842,0.24967290467995645,0.24433747868866182,0.25875865118185903,0.08510644648248718,0.006991717559217216,0.28419178384649074,0.3588203104496863,0.11955985076220237,0.5327957466160205,0.18428201247624137,0.1429209660985805,0.22696202234816662,0.37621106314583647,0.3387453849524397,0.4022923204036681,0.33481954763487237,0.3015999305845398,0.3581437107260246,0.10000416380122802,0.09994737677941584,0.10024408108306007,0.0,0.0,0.0,0.04

2019,500,500,intercept,50,10520.0,10520.0,10520.0,6240.351246070618,6240.351246070618,6240.351246070618,1017.866,1017.866,1017.866,0.08905635008953747,0.08905635008953747,0.08905635008953747,0.8530096232651709,0.8470593645365001,0.8561289508615187,0.3833833472092373,0.3730125282866539,0.3902649906797084,0.06637811890925382,0.06591078799258418,0.0671292121656566,0.18178292926783307,0.17252449690790556,0.18754353441879826,0.04467859639532531,-0.09099552859360666,0.23016733544180376,0.9703573490811861,0.9363856575259972,0.9880461638755476,1.0327016276932603,0.9372082986600265,1.1818763108275678,0.418135147455559,0.3617325758007439,0.4565303586130198,0.581864852544441,0.5434696413869802,0.638267424199256,0.4052753356915512,0.3754148260921607,0.43481839686395196,0.09120431244166993,0.074710212689749,0.10788609516209781,0.22664374346946542,0.22522206701774988,0.23003787398505726,0.0713339590863683,0.012378220350360583,0.23016733544180376,0.02964265091881385,0.011953836124452394,0.06361434247400277,0.23457683127725348,0.2029306847433808,0.2601587213250986,0.4406877231384092,0.43027239341917306,0.4500420978792886,0.39221238793044466,0.3832886975136144,0.40077555332631076,0.10000996460016515,0.09992442425167572,0.10025719631323618,1.0,0.0,1.0,0.66

2019,500,500,logistic,50,10520.0,10520.0,10520.0,6240.351246070618,6240.351246070618,6240.351246070618,1017.866,1017.866,1017.866,0.08905635008953747,0.08905635008953747,0.08905635008953747,0.8503801850140221,0.8441100149597667,0.8546570339471632,0.37856785532483456,0.35989097148431676,0.38753965885730396,0.06677846018825145,0.06609363112881116,0.0681896750695694,0.17684807913985012,0.15945258608672225,0.18528969870025894,0.048262752158226896,-0.09532132824587666,0.2268291485068256,0.9444215567477884,0.8363538330412208,1.0649073302662526,1.0351170650632249,0.9324713418649088,1.1784082409286556,0.41394265060430346,0.3469656123694082,0.46154959002462015,0.5860573493956965,0.5384504099753797,0.6530343876305917,0.4068720113495745,0.37541712696633345,0.44049467544851406,0.09131464573729077,0.06997219098739406,0.10964626185623585,0.22870624140694756,0.2258679476922465,0.2325921578086621,0.0714855526579464,0.01926540758451179,0.2268291485068256,0.06253142824848568,0.006190877708772592,0.1636461669587791,0.23647721828961865,0.1978743468423916,0.26536371960365096,0.4375913921871838,0.4252583051207134,0.44787727461178584,0.389724299654684,0.37858981072730796,0.398949857412752,0.1000112901220942,0.09995029205272785,0.10020606003952891,0.5,0.0,1.0,0.5

2019,500,500,refit,50,10520.0,10520.0,10520.0,6240.351246070618,6240.351246070618,6240.351246070618,1017.866,1017.866,1017.866,0.08905635008953747,0.08905635008953747,0.08905635008953747,0.8314525452908017,0.8180269094874245,0.8414173971611284,0.33407267608261904,0.30882353379437977,0.3566398994283995,0.06948343423617012,0.06812108956246442,0.07081947490040869,0.14350492362017775,0.12703607369439393,0.16029801276708416,0.07112581807697542,-0.06811668054800396,0.20817632334157374,1.2027151495552875,1.088832331663245,1.2705810374058142,1.0584470756011752,0.9474556505807878,1.1850580010235214,0.2843928375640801,0.2256172128747792,0.34947961224758467,0.7156071624359199,0.6505203877524153,0.7743827871252208,0.4002173625457395,0.36999541109643413,0.4403073394043794,0.06193313103214735,0.048025778575490195,0.08149743573626894,0.24092329188754377,0.23690594313104837,0.2465394099517486,0.08193422826450468,0.008981428399715469,0.20817632334157374,0.20271514955528747,0.08883233166324492,0.27058103740581424,0.19623872976918133,0.17477272730560145,0.2218119183613033,0.3976146663706225,0.3667544942065066,0.4274387591293942,0.3541988984957037,0.3264357712683192,0.37989626500369905,0.09999928605551543,0.09993917779195888,0.10015683986682801,0.0,0.0,0.5499999999999972,0.06

2019,1000,1000,intercept,50,10520.0,10520.0,10520.0,6240.351246070618,6240.351246070618,6240.351246070618,1017.866,1017.866,1017.866,0.08905635008953747,0.08905635008953747,0.08905635008953747,0.8549792051685869,0.8513026852580632,0.8568832424826986,0.3854760796820157,0.37779809610925635,0.39032220891533154,0.06617547951181477,0.06589173990830266,0.06661214698725254,0.1842807857439136,0.1788981568217962,0.1877783326923858,0.005107734679466189,-0.12109062318116448,0.14333432237082258,0.9815917002104044,0.9582958320397555,0.9907684017541442,1.0036721226507161,0.9174861406031929,1.1092883897111072,0.42656101097786947,0.38610781772846325,0.46467066391843326,0.5734389890221305,0.5353293360815667,0.6138921822715366,0.3993958484363159,0.3724889156439603,0.42659065355011117,0.09513682977903742,0.08215228731582869,0.11122399823592717,0.22557315294276564,0.22471816108692866,0.227473663840677,0.049994846187868744,0.00551634291991812,0.15424279768389698,0.018408299789595628,0.009231598245855788,0.04170416796024448,0.24119173086194273,0.2183152990604214,0.26465459340456,0.4412450656569725,0.4336521064658806,0.44781943792208406,0.39310960359310787,0.386202496856902,0.39902668903566985,0.10000817973895373,0.0999179178201077,0.10007700057503152,1.0,0.0,1.0,0.8

2019,1000,1000,logistic,50,10520.0,10520.0,10520.0,6240.351246070618,6240.351246070618,6240.351246070618,1017.866,1017.866,1017.866,0.08905635008953747,0.08905635008953747,0.08905635008953747,0.8535149574444187,0.8491341534276972,0.8566405208646023,0.38299710504568946,0.3684287704258574,0.38908087632669763,0.06639657835739718,0.0659551269779092,0.06733286360416221,0.18155538688134532,0.1700141653980256,0.18699698511478016,-0.0013196874200566287,-0.12474894008020793,0.1384956336131642,0.9693525008417333,0.9193557315508036,1.06438214492523,0.999047194207471,0.9162413176961285,1.106218453080139,0.42263908019326707,0.39392800231071673,0.4624231283882161,0.5773609198067329,0.5375768716117839,0.6060719976892832,0.3999322378500343,0.37127954374884525,0.42867488569663426,0.09408962808313229,0.08217917747488351,0.11094713913914445,0.2266219073009212,0.22507170164242757,0.22925293006115297,0.050371894251042784,0.0015823624901530999,0.15234780862401787,0.03539222013189508,0.004947010758778586,0.09356928707603714,0.23766210066445287,0.2166625117515499,0.26690575974937747,0.43958630114376546,0.43026590926507025,0.4481593697009233,0.39138512018523464,0.3834357664760559,0.3994848717379494,0.10001339433347337,0.09990398365361107,0.10015469453364642,1.0,0.0,1.0,0.76

2019,1000,1000,refit,50,10520.0,10520.0,10520.0,6240.351246070618,6240.351246070618,6240.351246070618,1017.866,1017.866,1017.866,0.08905635008953747,0.08905635008953747,0.08905635008953747,0.8416335802870917,0.8340462298367685,0.849189580716158,0.3513263398671723,0.32962449583049286,0.3753002248211632,0.06818639441205361,0.06691120248029124,0.06930575681302324,0.15949302546684863,0.14569508361704708,0.17521181690253734,0.006306106169968887,-0.10078981743027497,0.13926220185395177,1.0799719205341392,1.036922815492612,1.1514100463258243,1.0048478739620437,0.9259020420582533,1.1147480147231046,0.35779567251484967,0.3154462669938872,0.39771472374556177,0.6422043274851503,0.6022852762544382,0.6845537330061128,0.39220977933627343,0.36573937693221226,0.4216808103955994,0.08173054036034476,0.06667671380208064,0.09288351818123063,0.23420198152125693,0.23045356803352168,0.2378225881468293,0.04622421074590267,0.005216919432028803,0.1415836424186346,0.07997192053413926,0.036922815492612074,0.1514100463258244,0.21875232525676913,0.19630594599063378,0.23814560520401046,0.41625552872382027,0.38468009541531,0.4327317299133678,0.3700489572764706,0.3427586160113988,0.3853844400724,0.1000122744205564,0.0999125278017288,0.10023446470581755,1.0,0.0,1.0,0.54

2019,2000,2000,intercept,50,10520.0,10520.0,10520.0,6240.351246070618,6240.351246070618,6240.351246070618,1017.866,1017.866,1017.866,0.08905635008953747,0.08905635008953747,0.08905635008953747,0.8553162951027119,0.8534883677389515,0.8565637999967474,0.3862983898380157,0.3826297833313738,0.3891760129202246,0.06605343582200254,0.06592027109837686,0.06635387676729657,0.18578517050233107,0.18208175265581825,0.18742663988833183,0.0017559707112903549,-0.06211874940670886,0.058186575990258636,0.9864518192368011,0.972760351580597,0.9928623558139433,1.0012630027293576,0.9566051376896553,1.0428648519010373,0.43139951624280604,0.4114271623180261,0.45025042097879286,0.568600483757194,0.5497495790212071,0.588572837681974,0.40435174062200807,0.38992751706106377,0.4135408352437029,0.09487998037632592,0.0889332174141804,0.10243586527766083,0.22516368033734818,0.22471768017564048,0.2264038283497848,0.02291412594121179,0.0031467992176787206,0.0866451318601092,0.013548180763198858,0.00713764418605663,0.02723964841940292,0.23978330640388323,0.22951694463188033,0.2525896265138206,0.4430473166408938,0.4378183424930197,0.44886098464827395,0.3944541471900075,0.39007449437919983,0.399186564215814,0.09999600943530132,0.09991775508276196,0.1001630313228881,1.0,1.0,1.0,0.98

2019,2000,2000,logistic,50,10520.0,10520.0,10520.0,6240.351246070618,6240.351246070618,6240.351246070618,1017.866,1017.866,1017.866,0.08905635008953747,0.08905635008953747,0.08905635008953747,0.8542632662944345,0.8517875683003954,0.8554649084693063,0.3853803626868698,0.37821031092632074,0.38945577323990144,0.06617995621574735,0.06590104295821853,0.06669785533024297,0.18422560316811248,0.1778416636506833,0.18766365764018308,0.002435358851953555,-0.06264010658663671,0.061310872265033595,0.9835650887388863,0.9202080589130507,1.0413841651046296,1.0017482452968611,0.9565909098107248,1.0458241945530542,0.42681404035501724,0.40497260936115365,0.4511794283333956,0.5731859596449828,0.5488205716666044,0.5950273906388465,0.40377774971187896,0.3882783778597204,0.4174084692446127,0.09497997197610411,0.0859979405326416,0.1042889882436354,0.22583914920469406,0.22489476335350236,0.2271809162910095,0.021207530319618438,0.0033513565667653844,0.08630698267085479,0.025724146840889084,0.0027994103536148566,0.07979194108694926,0.23926278775011262,0.22629338777419497,0.2551157585134116,0.4432302483824,0.4359199197143829,0.4493851892095816,0.394759457033722,0.3881433461402712,0.40009625218781875,0.0999986517298398,0.09992055530252036,0.10017030813205254,1.0,1.0,1.0,0.98

2019,2000,2000,refit,50,10520.0,10520.0,10520.0,6240.351246070618,6240.351246070618,6240.351246070618,1017.866,1017.866,1017.866,0.08905635008953747,0.08905635008953747,0.08905635008953747,0.846362003562892,0.841859934302686,0.852418862320838,0.3650379750441244,0.35623432015146,0.37850871551885196,0.06719474191001856,0.06657331240604702,0.0678994680010173,0.17171673741789978,0.16302985494336295,0.17937685549237053,0.007930417336164276,-0.05726848689890293,0.06684139628765032,1.0302651493287378,0.9836965837017828,1.0809462174775633,1.0059593232272526,0.9587808589060078,1.0518971336414056,0.38891101579186255,0.36738133015544283,0.4185167202755569,0.6110889842081374,0.581483279724443,0.6326186698445572,0.39329175108632475,0.37571399185177456,0.41344666902772204,0.08720281984273255,0.08012281437510771,0.09666823676124472,0.2304113099722906,0.2275929231201723,0.23234529144628865,0.023370470690918136,0.0038910498853040702,0.08610168584113004,0.030943172599436353,0.004736159962171782,0.08094621747756323,0.22811418823427154,0.21901007322362248,0.24307399226034473,0.4278379963570843,0.41251189252809306,0.44371990517415855,0.3810191140832685,0.36756364589707025,0.3951451829392521,0.10000869157415407,0.09992873504147409,0.10011009837638787,1.0,1.0,1.0,0.96

2019,4000,4000,intercept,50,10520.0,10520.0,10520.0,6240.351246070618,6240.351246070618,6240.351246070618,1017.866,1017.866,1017.866,0.08905635008953747,0.08905635008953747,0.08905635008953747,0.8554635951978327,0.8544157738605512,0.8565508486247204,0.38691465154739235,0.384355524069794,0.3887956494307802,0.06601274414850292,0.06589449939228918,0.06617651610098223,0.18628676082217072,0.18426800811529,0.18774431761420515,0.011651519963284871,-0.022295017517984288,0.041518211492456214,0.9898437740627541,0.9831912779636068,0.9928330719641021,1.008420063173961,0.9841265693168921,1.0303893030062734,0.4267770512032036,0.41643904993388126,0.4406212703833314,0.5732229487967964,0.5593787296166687,0.5835609500661186,0.406747469530367,0.3993509518602159,0.41351854391760384,0.09327399463077,0.09021960494879369,0.09744110310162418,0.22503371275901224,0.2246947182796532,0.22560930079096467,0.019720841634577005,0.003633624180792107,0.04211979138942995,0.0101562259372458,0.007166928035897901,0.016808722036393203,0.23885904245797218,0.2319877703757826,0.24490000198120013,0.44468255153428843,0.43839635570890473,0.4480680806707366,0.39598633844930436,0.39003593372629325,0.3991379513043023,0.09999942166997022,0.09994025395827753,0.10010835288711493,1.0,1.0,1.0,1.0

2019,4000,4000,logistic,50,10520.0,10520.0,10520.0,6240.351246070618,6240.351246070618,6240.351246070618,1017.866,1017.866,1017.866,0.08905635008953747,0.08905635008953747,0.08905635008953747,0.8545180077556523,0.8531009252801187,0.8561114263323597,0.3852592089056258,0.380775880434437,0.3886567538639122,0.06616258798097038,0.06596478600280888,0.06638917739531539,0.18443969459487336,0.18164661564188664,0.18687792209839144,0.0127010775835076,-0.023236096301436096,0.04231183435329743,0.9874735851149474,0.9653772429930317,1.0132279670077162,1.0091267868525842,0.9833738533279338,1.0308759474077023,0.4260766151929625,0.41361041139010435,0.43778908029151187,0.5739233848070375,0.5622109197084881,0.5863895886098957,0.40786024708133606,0.39762649184174587,0.4146849218746725,0.09354291809461762,0.08922194540055307,0.09722259802980543,0.22560605109646564,0.22481833969463144,0.2263288403768036,0.020761265625330528,0.0035193066617882534,0.042540328599326487,0.013638754260985875,0.002399816955961304,0.03789862709121779,0.23810590656205888,0.2301519020741527,0.24281247041312368,0.4439016530663172,0.43830609333645093,0.4466256019947616,0.39508681157027314,0.3903148029485183,0.39767456787268185,0.10000017411146131,0.09992627604518256,0.1001383131824398,1.0,1.0,1.0,1.0

2019,4000,4000,refit,50,10520.0,10520.0,10520.0,6240.351246070618,6240.351246070618,6240.351246070618,1017.866,1017.866,1017.866,0.08905635008953747,0.08905635008953747,0.08905635008953747,0.850757184925763,0.8480349538550255,0.8548655765724602,0.3722042719199718,0.3660142361113855,0.38462688662801636,0.06680335967551929,0.06612473348216974,0.06723970774035794,0.17654115291371103,0.17116246124093218,0.18490631217355227,0.010277545692188153,-0.025569489174443356,0.0570068690602941,1.0001209360608057,0.9742452598641739,1.0314168337502134,1.0075188814883997,0.9814644648624739,1.042664572441294,0.40507262252595133,0.3853207494896185,0.4223808241949333,0.5949273774740487,0.5776191758050667,0.6146792505103815,0.39949745241787216,0.38882626620321215,0.4139663055180297,0.08999796937039922,0.08463507169511883,0.09503850054999535,0.2276995321451145,0.22571396143970837,0.22938274225262037,0.019825262872970758,0.002926635796915065,0.06059830650452493,0.014822424403788392,0.003895185099347698,0.03663860743057188,0.2313445417395512,0.22297074851162044,0.24086569426542856,0.4341248258611644,0.42375014982325765,0.4426649529505849,0.38607114738548587,0.3774403781191883,0.39426458808018977,0.09999292967477966,0.0999139145688955,0.10012602826269983,1.0,1.0,1.0,1.0

2021,-1,full_outer_training,intercept,50,15573.0,15573.0,15573.0,7465.749225286986,7465.749225286986,7465.749225286986,1579.6734000000001,1579.6734000000001,1579.6734000000001,0.10170608668544727,0.10170608668544727,0.10170608668544727,0.851185851734324,0.8511193797512514,0.8512675139330695,0.4400473517083068,0.43952184712772924,0.44019521257568717,0.07192907159324657,0.0719153013381625,0.07194653049966888,0.21270217186222273,0.2125110758449451,0.21285289383986616,0.0006777235913858606,0.00027494066442871647,0.003286392928683074,1.0116361856311582,1.0114601969036008,1.012227657366296,1.000478031567583,1.0001939116366263,1.0023209243936522,0.48410278985516875,0.48229039939521673,0.48825152085234835,0.5158972101448313,0.5117484791476516,0.5177096006047832,0.42100773516266715,0.4190315802633736,0.42280524029893496,0.11701726648439444,0.11681656146269531,0.11744922942966197,0.24502871691010983,0.24496911272388847,0.24504734660179583,0.0006777235913858606,0.00027494066442871647,0.003286392928683074,0.011636185631158091,0.011460196903600705,0.012227657366296052,0.27784897526688546,0.2768697817650996,0.27899062568169447,0.42947884037295303,0.4280057510622133,0.42947884037295303,0.43674599924667923,0.4351840814285737,0.43720733035793924,0.09998656365092279,0.09990823377269512,0.1000556801430134,1.0,1.0,1.0,1.0

2021,-1,full_outer_training,logistic,50,15573.0,15573.0,15573.0,7465.749225286986,7465.749225286986,7465.749225286986,1579.6734000000001,1579.6734000000001,1579.6734000000001,0.10170608668544727,0.10170608668544727,0.10170608668544727,0.8506005794541037,0.850302604505016,0.8509723731387733,0.43835523297794976,0.4381118392271862,0.43957152336873545,0.07200169495741951,0.07195370610922928,0.07201667240033305,0.21190727467226955,0.21174333945086743,0.21243253539256146,0.0007598432554354124,0.00040724322624619854,0.003639385475375366,0.9975216776496837,0.9922121800969161,0.9996702665847832,1.0005325122609048,1.000285393875513,1.0025580118660515,0.4897303455258536,0.48474317539309064,0.49456273682901786,0.5102696544741464,0.5054372631709821,0.5152568246069094,0.42273309926059116,0.41976378005847687,0.42506672275841695,0.11782506991212045,0.11738734715187162,0.11839340899980684,0.24531145790554554,0.2451132097165592,0.24535869376514174,0.0007598432554354124,0.00040724322624619854,0.003639385475375366,0.0024783223503163088,0.0003297334152168041,0.007787819903083859,0.2787870664905985,0.27810154587272273,0.2796556972580392,0.4299820456557665,0.42522967089273,0.4318215398195601,0.43631037895014746,0.43279615565492047,0.4385429053122724,0.09999745746597796,0.09993573872418192,0.1002309211939423,1.0,1.0,1.0,1.0

2021,-1,full_outer_training,refit,50,15573.0,15573.0,15573.0,7465.749225286986,7465.749225286986,7465.749225286986,1579.6734000000001,1579.6734000000001,1579.6734000000001,0.10170608668544727,0.10170608668544727,0.10170608668544727,0.8558216473243871,0.855627632758185,0.8561859828443307,0.44971290717718904,0.44793965790887186,0.4514606529254199,0.0712473924053258,0.0711607122197697,0.07142889220565682,0.22016347411802917,0.21817687265852315,0.22111222988978163,-0.01790446707150138,-0.020224865044283478,-0.014306746544853128,0.9846345669404513,0.9832108713040397,0.9897195265501798,0.9878947030406997,0.986343143903989,0.9903212277948725,0.47554045032346554,0.4731162150353357,0.47835248412741516,0.5244595496765345,0.5216475158725848,0.5268837849646644,0.41867808222725744,0.4149752655056691,0.4235332744144641,0.11541346770034377,0.11395312990155282,0.11654997847666726,0.24213006697570125,0.24192320003289297,0.24248252907646456,0.01790446707150138,0.014306746544853127,0.020224865044283478,0.015365433059548794,0.010280473449820193,0.016789128695960276,0.2766737310857525,0.2736521698349082,0.2799516869653263,0.44460924644296723,0.44080877730801826,0.44648849565992565,0.4520504186698531,0.44825812591648495,0.454079661492383,0.10001589067667711,0.09991191655177997,0.10010465724178685,1.0,1.0,1.0,1.0

2021,0,none,static,1,15573.0,15573.0,15573.0,7465.749225286986,7465.749225286986,7465.749225286986,1579.6734000000001,1579.6734000000001,1579.6734000000001,0.10170608668544727,0.10170608668544727,0.10170608668544727,0.8515625000389302,0.8515625000389302,0.8515625000389302,0.44067224828804763,0.44067224828804763,0.44067224828804763,0.07186769785506224,0.07186769785506224,0.07186769785506224,0.2133739365563434,0.2133739365563434,0.2133739365563434,0.02965953518780984,0.02965953518780984,0.02965953518780984,1.0134040898681458,1.0134040898681458,1.0134040898681458,1.0211846163865483,1.0211846163865483,1.0211846163865483,0.4706577954658222,0.4706577954658222,0.4706577954658222,0.5293422045341778,0.5293422045341778,0.5293422045341778,0.421406265896917,0.421406265896917,0.421406265896917,0.11359290646271007,0.11359290646271007,0.11359290646271007,0.2448441581407855,0.2448441581407855,0.2448441581407855,0.02965953518780984,0.02965953518780984,0.02965953518780984,0.013404089868145785,0.013404089868145785,0.013404089868145785,0.27126045287083456,0.27126045287083456,0.27126045287083456,0.4314394988229845,0.4314394988229845,0.4314394988229845,0.436868966905357,0.436868966905357,0.436868966905357,0.10044206934094847,0.10044206934094847,0.10044206934094847,1.0,1.0,1.0,1.0

2021,250,250,intercept,50,15573.0,15573.0,15573.0,7465.749225286986,7465.749225286986,7465.749225286986,1579.6734000000001,1579.6734000000001,1579.6734000000001,0.10170608668544727,0.10170608668544727,0.10170608668544727,0.8470943433077718,0.8359138589815974,0.8506634544686984,0.431895631534308,0.4085089383513924,0.440530776814713,0.07258460203534431,0.0719897357279902,0.07454427316370087,0.20552708004037584,0.18407754942577134,0.21203817411455822,-0.003862633239871565,-0.18253150055826425,0.24304810344986624,0.9850959766270926,0.9059785334750219,1.0066492966853777,0.9973019148051602,0.8816431652296508,1.1892466309858307,0.47575543147083443,0.3987147976284211,0.5501103899071795,0.5242445685291656,0.44988961009282047,0.6012852023715788,0.4129041535859279,0.3892643612928069,0.44140154962935757,0.1177074141341546,0.09187765970128452,0.14225815387731383,0.24742724374858782,0.24528180446382739,0.2546076357031379,0.09398487638327291,0.009778805606951286,0.26546536293590167,0.015196180516733104,0.00128903057704296,0.09402146652497805,0.27957820043392834,0.23160073957298913,0.31423636784520953,0.42943468567616566,0.4160065112193444,0.44037363989290434,0.4366609745400065,0.4229835409001777,0.4478980200360985,0.10000113380663783,0.09992691389699199,0.100069388837467,0.5,0.0,1.0,0.5

2021,250,250,logistic,50,15573.0,15573.0,15573.0,7465.749225286986,7465.749225286986,7465.749225286986,1579.6734000000001,1579.6734000000001,1579.6734000000001,0.10170608668544727,0.10170608668544727,0.10170608668544727,0.8423306561802261,0.8289233104955253,0.848755173688116,0.42137605558361435,0.3977773227410902,0.4332116597575869,0.07350197122592295,0.07234998052499075,0.07623288185005547,0.19548603884041021,0.16559492589269628,0.20809512382912815,0.0007495615576935933,-0.18416244391879458,0.2451014087282244,0.9003848256185467,0.7539895082872523,1.0735567398780754,1.0005805543680886,0.884329902506663,1.1931605919961679,0.4744321832601599,0.3711165865045267,0.5487823970448574,0.5255678167398401,0.45121760295514257,0.6288834134954733,0.4171348445870068,0.3887357732117155,0.44389693654255036,0.11603162410126269,0.08433495824800516,0.1428065241899449,0.25151110772050583,0.248197675655216,0.2621116147332169,0.09092127994361865,0.008607943416714173,0.2778604957318346,0.10981289268608563,0.00950560582508574,0.24601049171274772,0.2766080319148994,0.22184841164947913,0.3317901628760571,0.42638452986547726,0.41007970065204613,0.4381560707422179,0.43317497472722477,0.41709821228589,0.44558813912291173,0.10000007146651721,0.0999276671927139,0.1001871917334435,0.0,0.0,1.0,0.24

2021,250,250,refit,50,15573.0,15573.0,15573.0,7465.749225286986,7465.749225286986,7465.749225286986,1579.6734000000001,1579.6734000000001,1579.6734000000001,0.10170608668544727,0.10170608668544727,0.10170608668544727,0.8160577204075904,0.7930484043613643,0.834177279370446,0.3759348129424426,0.3322459435140646,0.40097793215408184,0.07721920731202117,0.07527200175102765,0.08004563482387698,0.15479912557382375,0.12386253495499153,0.1761122146371329,0.05321805281730674,-0.17027693079625705,0.23410576817200912,1.258576051369237,1.144297761050182,1.45150310848901,1.0434889107186294,0.8757132085513094,1.209838972773824,0.32512119277313906,0.21335048434695425,0.4165303631750714,0.674878807226861,0.5834696368249285,0.7866495156530457,0.4398497517150299,0.3798716471127998,0.49268824028333924,0.07620361413388546,0.045576962038061275,0.10716718160438456,0.26748452787007115,0.25998170504264667,0.27736489730284086,0.09572587110901723,0.01119734082609875,0.25445766116430724,0.258576051369237,0.14429776105018205,0.45150310848901004,0.21361505954025542,0.17622808564635373,0.2587310263555507,0.396370192724648,0.36022384753709213,0.42142144382503366,0.40311102338646765,0.36649139719141527,0.42792325044198043,0.10000380253378935,0.09992710447437121,0.10015719607725568,0.0,0.0,0.0,0.0

2021,500,500,intercept,50,15573.0,15573.0,15573.0,7465.749225286986,7465.749225286986,7465.749225286986,1579.6734000000001,1579.6734000000001,1579.6734000000001,0.10170608668544727,0.10170608668544727,0.10170608668544727,0.8494863269773987,0.8457472069804307,0.8515512778880417,0.437267128433278,0.4276532424051616,0.4418246496422255,0.07220077751231631,0.07178056372334096,0.07298163958366453,0.2097282216187739,0.2011813156288828,0.21432766098432937,0.015421267046690241,-0.13484882721742905,0.17840765394443758,0.9982136199927806,0.9738301713536843,1.0126604113223177,1.0109273030425177,0.9103781811349084,1.1350656874313922,0.4729701721887575,0.4185245696990276,0.5203970770160464,0.5270298278112425,0.4796029229839536,0.5814754303009723,0.42218584179548324,0.3976487900637457,0.4452154327006896,0.11436634801635251,0.09739794048563327,0.1312379106901477,0.2461566002963512,0.2446830696196297,0.24856682261865684,0.06756614225988813,0.00553403061515587,0.17840765394443758,0.007059904619842072,0.0008139553136108813,0.02616982864631565,0.27284946494364226,0.24435747924946427,0.3043501818823115,0.4308917273659225,0.42344405811986197,0.44318687964233616,0.43825280570482583,0.4306919795455094,0.4506341333517389,0.10000488096997243,0.09994630514532721,0.10022211729163354,1.0,0.0,1.0,0.62

2021,500,500,logistic,50,15573.0,15573.0,15573.0,7465.749225286986,7465.749225286986,7465.749225286986,1579.6734000000001,1579.6734000000001,1579.6734000000001,0.10170608668544727,0.10170608668544727,0.10170608668544727,0.8467961375104285,0.8429687654523325,0.8502178039796991,0.42926571743096387,0.4146974902048894,0.4374268042241443,0.07270151635741873,0.07202620178834211,0.07401905468421452,0.20424739729446312,0.18982631496713345,0.21163903577619406,0.014281672930702234,-0.13578225728830565,0.18883069504766062,0.9516745235992277,0.825979558186984,1.0997341976418566,1.0099466309693594,0.9097830223242306,1.138994028187102,0.47327691280995166,0.4121908364096021,0.5160379386017387,0.5267230871900483,0.48396206139826126,0.5878091635903979,0.4202289134173751,0.3981902857472165,0.44674106205394587,0.11556622255205273,0.09240011921645219,0.13172587376814984,0.2480432394818367,0.24577808209576726,0.2508932018059655,0.07126123975139301,0.007719757608652508,0.18883069504766062,0.0671197907262675,0.009026767095487304,0.1745608350154201,0.27627703216512534,0.23461833298729218,0.3108079033325273,0.4314094926204366,0.42000244797437236,0.44156546536771457,0.4387039934571779,0.4264202561264279,0.4484523734713349,0.10000232491525794,0.09993959405305607,0.10023727044672992,0.0,0.0,1.0,0.48

2021,500,500,refit,50,15573.0,15573.0,15573.0,7465.749225286986,7465.749225286986,7465.749225286986,1579.6734000000001,1579.6734000000001,1579.6734000000001,0.10170608668544727,0.10170608668544727,0.10170608668544727,0.8350519287551279,0.8226971182844129,0.8445811868143456,0.3988802958226953,0.38371508335548693,0.4177725650285661,0.07480463104876454,0.07352191584021035,0.0765268492592013,0.18122780880061318,0.1623773130744388,0.19526773557076244,0.04439015974882933,-0.10696734511295279,0.17694742241552544,1.126127032903587,1.0367307303046722,1.2087511220909062,1.0341177217099073,0.9223311844675839,1.1432618057888624,0.3873280071690768,0.3333740031325462,0.4484347460683961,0.6126719928309232,0.5515652539316039,0.6666259968674537,0.42916650984332394,0.39290728947954956,0.47132559519966893,0.09165936232718777,0.0742995664834628,0.11177969694063236,0.25672295078941965,0.2526207548321159,0.2633736132565551,0.06931493196992326,0.012654895381866815,0.17694742241552544,0.12612703290358707,0.04003359512852972,0.2087511220909063,0.23564334722418934,0.2079063079618621,0.26554240637157145,0.4118922620334051,0.39209822739308003,0.4322782449840581,0.41870168674355046,0.39870940773404,0.43960919995114195,0.09999775363352675,0.09992987525055856,0.10016996991240311,0.0,0.0,1.0,0.16

2021,1000,1000,intercept,50,15573.0,15573.0,15573.0,7465.749225286986,7465.749225286986,7465.749225286986,1579.6734000000001,1579.6734000000001,1579.6734000000001,0.10170608668544727,0.10170608668544727,0.10170608668544727,0.8503113374168494,0.8479238727549416,0.8517151507682155,0.4382198452800344,0.431173608943455,0.44135873631350153,0.07207090507109758,0.07177869276822926,0.07267351062270294,0.21114973712900692,0.20455393335323263,0.21434813947603454,-0.009075350617058438,-0.11079106476855921,0.11730358335108836,1.0054371854420676,0.98119611809759,1.0139649688151477,0.993634497234406,0.9255486929495108,1.086861135500638,0.48595535634138043,0.4424291090803959,0.5125914603613633,0.5140446436586197,0.48740853963863673,0.5575708909196041,0.4194086352034627,0.4042815443531932,0.4311178523219969,0.11801754984591595,0.10453225232092184,0.12958546155739098,0.245476134316577,0.24470852388458691,0.24729334728001412,0.05469421108440983,0.006366815340771729,0.1259965043557774,0.007606554312398317,0.0011168199520233514,0.018803881902410006,0.2795376408392244,0.25628179209411045,0.3017228715568024,0.43160054477083676,0.42518442419806524,0.4369902759646392,0.4389845023232621,0.43215910385114154,0.44429478874369444,0.10001026349325028,0.09993741142699006,0.10010563652622533,1.0,0.0,1.0,0.84

2021,1000,1000,logistic,50,15573.0,15573.0,15573.0,7465.749225286986,7465.749225286986,7465.749225286986,1579.6734000000001,1579.6734000000001,1579.6734000000001,0.10170608668544727,0.10170608668544727,0.10170608668544727,0.8483907806538258,0.8440020646305211,0.8513604275971883,0.43525587812258487,0.42315658802449363,0.4405469844338762,0.07227521915930735,0.07183687240821449,0.07339562291880626,0.20891342246036881,0.19665007153768388,0.21371133570271159,-0.010416015427529781,-0.11126046708224135,0.1221580116949662,0.9711240735365467,0.8843171002313808,1.0764445999654526,0.9926999171708757,0.9267141027819314,1.0906553421926617,0.4828563613212705,0.4422588017244577,0.5208029773749434,0.5171436386787295,0.4791970226250566,0.5577411982755422,0.41914699352181783,0.40233731565958375,0.43299649167045356,0.11782448401544789,0.10474193862353777,0.13071384671947667,0.24650092348513564,0.24508735806154527,0.24970313874647906,0.05322511134731582,0.006728695204385856,0.12730900618711072,0.04854961555434356,0.006433324011121788,0.11725414994451161,0.2783762031248881,0.2561338437716686,0.3068233200006711,0.43068462759453946,0.4249383765023833,0.4394638853828899,0.4378708820994985,0.4323822029879368,0.44738369388196025,0.09999812706217522,0.09991401901945506,0.10009706022223937,1.0,0.0,1.0,0.72

2021,1000,1000,refit,50,15573.0,15573.0,15573.0,7465.749225286986,7465.749225286986,7465.749225286986,1579.6734000000001,1579.6734000000001,1579.6734000000001,0.10170608668544727,0.10170608668544727,0.10170608668544727,0.84315075186826,0.8384878122107098,0.8483140142721467,0.41710104504447465,0.4022381411135987,0.4301783829604557,0.07358834355244026,0.07257900249379455,0.07451201259428329,0.19454065273195548,0.1844306566698154,0.205588369680337,0.0029420336411282843,-0.10412509063481595,0.12096321927829697,1.0529938134497503,0.9643213261149725,1.14948224273794,1.0021464494616261,0.9288943017011535,1.0911918399739702,0.44221992343480615,0.3999491192293293,0.48093141594965133,0.5577800765651939,0.5190685840503486,0.6000508807706707,0.4209644285161815,0.4005843592230737,0.4431724768396558,0.10678652228275468,0.09433376076534435,0.12065342750937728,0.25136145463140214,0.24871135923927046,0.2541043475650602,0.051381167841914335,0.0036326214363257368,0.12820435080138268,0.05299381344975029,0.0060858308224580274,0.14948224273794003,0.2602949870255753,0.23690836753410946,0.28770680894956135,0.4243212552670697,0.41100766778753123,0.4409846459401038,0.43127379163671165,0.4180558966049819,0.44886666743108955,0.09999816247351256,0.09991091247940534,0.10004185265961905,1.0,0.0,1.0,0.68

2021,2000,2000,intercept,50,15573.0,15573.0,15573.0,7465.749225286986,7465.749225286986,7465.749225286986,1579.6734000000001,1579.6734000000001,1579.6734000000001,0.10170608668544727,0.10170608668544727,0.10170608668544727,0.8506151741407887,0.8488143936351354,0.8517192153606957,0.4384581892453704,0.43411927864663136,0.44083061144395885,0.07204611544889598,0.07181752749218534,0.07239266852569766,0.21142107130985155,0.2076278834004717,0.2139230749902888,-0.01413276950276909,-0.07347804955991619,0.07425506869595697,1.0074030937799732,0.9975620241061374,1.0130348549167738,0.99010463099513,0.9498311260675205,1.0539972247102556,0.48393636304820975,0.45666947674120484,0.510577870083778,0.5160636369517901,0.48942212991622197,0.5433305232587952,0.4185999878403487,0.4111466070586876,0.4304025559267329,0.11705843699288751,0.10843929559020878,0.12500177716625333,0.2454484347669596,0.24485310311707198,0.2464215763743587,0.040134018241001115,0.006244339227794795,0.09103239251598207,0.00740309377997328,0.0011689918696403013,0.013034854916773775,0.2793151219679766,0.26387972148374794,0.29444729606566966,0.42933903299251597,0.42502542614188477,0.4344132495995691,0.4364880711685537,0.43211866271081356,0.4419531252946805,0.10000547974349497,0.09994015805908374,0.10009303652855217,1.0,1.0,1.0,0.96

2021,2000,2000,logistic,50,15573.0,15573.0,15573.0,7465.749225286986,7465.749225286986,7465.749225286986,1579.6734000000001,1579.6734000000001,1579.6734000000001,0.10170608668544727,0.10170608668544727,0.10170608668544727,0.849509884847497,0.8467362476304644,0.8512966778459025,0.43591447263078886,0.42924445829134755,0.440281908084557,0.07220008282556326,0.07190418812781749,0.07268300583946934,0.20973582529500379,0.20445000369902888,0.21297453320173287,-0.01394636379058137,-0.07530587992590429,0.07522591538513686,0.978468487111092,0.9206127065311412,1.0269893038262985,0.9904137071653158,0.9493689329508391,1.0546611480278854,0.48534627474261444,0.4530920632074959,0.5129582450397657,0.5146537252573855,0.4870417549602342,0.546907936792504,0.4183353304668307,0.4069601805576336,0.4312354166237043,0.11853980268764198,0.10712326478571903,0.12605363971600134,0.24597119774515944,0.24513589223973675,0.2473941965079607,0.04098889033205051,0.008306981108327179,0.08733306486872244,0.026971274362963427,0.006967749921088273,0.0793872934688587,0.28093904418359783,0.26244030376581484,0.2961839025236096,0.42850227774931193,0.4252038522646516,0.435454243263196,0.4358888694970045,0.4325688206806589,0.4426642789085744,0.1000010436686882,0.0998880203374908,0.10010799298976564,1.0,1.0,1.0,0.96

2021,2000,2000,refit,50,15573.0,15573.0,15573.0,7465.749225286986,7465.749225286986,7465.749225286986,1579.6734000000001,1579.6734000000001,1579.6734000000001,0.10170608668544727,0.10170608668544727,0.10170608668544727,0.8483998621524184,0.8437930447283621,0.8521329493690095,0.430195975999332,0.418865831605095,0.441803569189517,0.07269232389514027,0.07185920072476652,0.07375029680132286,0.2043480131571006,0.19276799755539267,0.21346694168040575,-0.010430515297947158,-0.07773715288166225,0.06952357141998124,0.9981216660800909,0.9397671198187473,1.030894438754051,0.9926723149238619,0.9475379180576797,1.0504807242741092,0.46372905310680035,0.4349685542593804,0.49207620701848864,0.5362709468931997,0.5079237929815114,0.5650314457406196,0.4230203835260037,0.4019809735721686,0.4414843367845953,0.11201905279321123,0.10240299114029587,0.12142966301753694,0.24781939382657162,0.24514053858040016,0.250463936286127,0.04072709032387463,0.005611326984332406,0.08710604713140263,0.019040941842796144,0.0015739887551733279,0.06188219855919199,0.27147495360631674,0.2530993094924048,0.2900396672974154,0.43235050992186097,0.41788695688615124,0.44682153285609544,0.43974656831617165,0.4251728897262301,0.45441157615016686,0.10001045986521198,0.09995709272637031,0.10018021698766968,1.0,1.0,1.0,0.96

2021,4000,4000,intercept,50,15573.0,15573.0,15573.0,7465.749225286986,7465.749225286986,7465.749225286986,1579.6734000000001,1579.6734000000001,1579.6734000000001,0.10170608668544727,0.10170608668544727,0.10170608668544727,0.8511188674993628,0.8504404376022884,0.8517081701422726,0.43965184748187924,0.4375148852897226,0.441645452110255,0.07194318083964363,0.07178046553809032,0.07209965621693544,0.21254773946376398,0.21083504219171337,0.2143287356684991,-0.004272621570618551,-0.04897287640112913,0.04733032310238397,1.0110423763481629,1.0069456521698366,1.0141922363333473,0.9969940327163884,0.9661864430706746,1.0340654201327164,0.4847943252067167,0.46349210222822007,0.5005494711754974,0.5152056747932833,0.49945052882450264,0.53650789777178,0.422260890908105,0.41553900085265955,0.4256867734378622,0.11727858927721915,0.11147404301649508,0.12226517195368772,0.24511518257505574,0.24467855118132628,0.24551789786170178,0.021231779133388532,0.00375599107339127,0.05357327875889939,0.011042376348162852,0.0069456521698366466,0.01419223633334733,0.2783740270214953,0.2676586836729527,0.28862821272771555,0.42943658480290925,0.4250710969748556,0.43323916196854356,0.4365618149909285,0.43241740051058997,0.44077847643111423,0.10002314678162227,0.09995426110706697,0.10011768410703575,1.0,1.0,1.0,1.0

2021,4000,4000,logistic,50,15573.0,15573.0,15573.0,7465.749225286986,7465.749225286986,7465.749225286986,1579.6734000000001,1579.6734000000001,1579.6734000000001,0.10170608668544727,0.10170608668544727,0.10170608668544727,0.8503217168113224,0.8484709164720304,0.8513477392676148,0.4383121297175401,0.4343747545437542,0.4407570057358554,0.07205765067991811,0.0718318156456572,0.07231579453507288,0.21129481273137102,0.2084693057144066,0.2137666843689819,-0.004139795162209558,-0.0490381566888701,0.046765154975567706,0.9867927546237844,0.9458665006679573,1.0252852155491108,0.9970919114423684,0.9665875477245678,1.0336852010158137,0.48939701713024986,0.46432927844451893,0.5036468424422414,0.51060298286975,0.4963531575577585,0.5356707215554811,0.4204112443666519,0.41345328469138065,0.4255130720936992,0.11819356350784166,0.11125079320590872,0.12383724893981515,0.24546553164635537,0.24482505172608654,0.24620269601887196,0.02188963466521992,0.00412834553583561,0.05381418436696271,0.01846708999836355,0.0017756394576313784,0.05413349933204269,0.28049186745600835,0.2677632255492734,0.2936248002792865,0.4300377217214647,0.4253582037907329,0.4344774970573031,0.4371462168710606,0.4325299516728263,0.4415351158274967,0.10002237738983792,0.09995655737133376,0.10024930997950914,1.0,1.0,1.0,1.0

2021,4000,4000,refit,50,15573.0,15573.0,15573.0,7465.749225286986,7465.749225286986,7465.749225286986,1579.6734000000001,1579.6734000000001,1579.6734000000001,0.10170608668544727,0.10170608668544727,0.10170608668544727,0.852576403308405,0.8495563858487198,0.8547998136295143,0.44083831111667976,0.4321876202168644,0.44830116173630474,0.07192803874398368,0.07144600829491483,0.07273777357102176,0.21271347688762415,0.20385054488289012,0.21798952893781937,-0.010565436318897217,-0.07770921824207447,0.03683094204046729,0.9855257260705712,0.9532592777619937,1.0213384700547472,0.9927897874197966,0.9489348579081064,1.0260648688754581,0.47312865431550594,0.4553050681235753,0.49941658826438423,0.526871345684494,0.5005834117356157,0.5446949318764247,0.4247839499408719,0.4067886434921962,0.43965744806334445,0.11348229432166523,0.10732939933119831,0.12455214460748848,0.24472130007552953,0.24315555602912164,0.24697941500141068,0.02587543389077115,0.0037858699687104923,0.07770921824207445,0.02207761356820953,0.0016526851363893203,0.0467407222380063,0.2759357543903844,0.2621712391124468,0.29269029620870934,0.4413546496383366,0.428831481874671,0.449202249654897,0.4488315097867106,0.43626822284972994,0.4566168459117077,0.10000197724030938,0.09993526678763137,0.100139128569095,1.0,1.0,1.0,0.98

2023,-1,full_outer_training,intercept,50,19336.0,19336.0,19336.0,7383.468311248124,7383.468311248124,7383.468311248124,1831.6335,1831.6335,1831.6335,0.09462945164439508,0.09462945164439508,0.09462945164439508,0.8591681318440444,0.8586279175207695,0.8594994573486718,0.4276378691420633,0.42611988706451864,0.4284512054278514,0.0676675052186195,0.06759871173911054,0.06776063651433724,0.21018117849804174,0.20909414492127423,0.21098413975303773,-0.004061743872476433,-0.010876638398500602,-0.00010570613582787749,1.0051063364794537,1.0010409014428716,1.0078498057102956,0.997150241261577,0.9923985827140904,0.9999258023795138,0.46878600440535734,0.466061761263921,0.47234442643683905,0.5312139955946427,0.527655573563161,0.5339382387360789,0.4098794414423248,0.4042047127247775,0.41394704717130265,0.10837908784611752,0.1072190233358025,0.10961857898849324,0.23003875187046155,0.22980014156087236,0.23034402652310332,0.004084537513120384,0.0004163530374122652,0.010876638398500596,0.005106336479453577,0.0010409014428715115,0.007849805710295676,0.26334018336213966,0.26182156135784235,0.26565866976192276,0.44676724355609354,0.4425926010853154,0.4508228310958497,0.4222499983020701,0.4185284325329365,0.4266679852072393,0.10002246710907865,0.0999652541743413,0.10020074473297617,1.0,1.0,1.0,1.0

2023,-1,full_outer_training,logistic,50,19336.0,19336.0,19336.0,7383.468311248124,7383.468311248124,7383.468311248124,1831.6335,1831.6335,1831.6335,0.09462945164439508,0.09462945164439508,0.09462945164439508,0.8588984310955989,0.858496367943457,0.8593190517552005,0.42709376679155775,0.4247997126798855,0.4283280493494097,0.06771205524008808,0.06762789864100657,0.06783324275334474,0.20966118821111585,0.20824668092889978,0.2106434686379385,-0.0050016489756584594,-0.01230158955886085,0.00020325632341445236,0.9950117768238411,0.9869063498253249,1.0014182818658686,0.9965077145652386,0.9914591713325731,1.0001426709265087,0.46963349927810344,0.4663946826698682,0.4727206206918579,0.5303665007218966,0.5272793793081421,0.5336053173301317,0.4088338473979777,0.4024295565833399,0.41250372774463856,0.10883763138011046,0.10764601697709969,0.110305139630058,0.23017418685337399,0.229897238306923,0.2304876719467905,0.0050016489756584594,0.000618552595274876,0.012301589558860848,0.004988223176158879,0.0009641535578161664,0.013093650174675146,0.264073830595825,0.2621550617671941,0.26601534673999255,0.4464874932676215,0.442479630886856,0.4515939487894276,0.4222431730527738,0.4180740854739762,0.4270225498128221,0.10001307718318114,0.09995509548862341,0.10032108896812454,1.0,1.0,1.0,1.0

2023,-1,full_outer_training,refit,50,19336.0,19336.0,19336.0,7383.468311248124,7383.468311248124,7383.468311248124,1831.6335,1831.6335,1831.6335,0.09462945164439508,0.09462945164439508,0.09462945164439508,0.8642245797481831,0.8627577858240557,0.8653908248351525,0.4323861479722085,0.42852213430347286,0.4358855675735138,0.06706182870936772,0.06688599803030974,0.06724742680906491,0.21725066783717334,0.2150843566675472,0.2193029731389396,-0.012363233667647258,-0.028486518022182543,0.0020210979185904263,0.9838908690509467,0.9741611387466047,0.9942539891531728,0.9915615580407748,0.98073569271793,1.0013923007518268,0.4841497220923291,0.47726514338157716,0.49386707821188025,0.5158502779076709,0.5061329217881197,0.5227348566184228,0.4293233236672992,0.42068424096214163,0.43638564371553396,0.10645499515978382,0.10439531452668863,0.10998861465672734,0.22750871317833593,0.2267933064699533,0.22817303102634912,0.012363233667647258,0.0008297916848585699,0.028486518022182543,0.016109130949053307,0.005746010846827249,0.025838861253395334,0.2615377829281211,0.25721729283060685,0.26764290059511553,0.4676688049219454,0.46193265464952465,0.4736142929248674,0.4423719413475137,0.4372059429719953,0.4481884811970053,0.1000008250735438,0.09992462873098373,0.10013241784578175,1.0,1.0,1.0,1.0

2023,0,none,static,1,19336.0,19336.0,19336.0,7383.468311248124,7383.468311248124,7383.468311248124,1831.6335,1831.6335,1831.6335,0.09462945164439508,0.09462945164439508,0.09462945164439508,0.8596242232474842,0.8596242232474842,0.8596242232474842,0.4287899260930158,0.4287899260930158,0.4287899260930158,0.06780345405296319,0.06780345405296319,0.06780345405296319,0.20859437626883315,0.20859437626883315,0.20859437626883315,-0.14762652942101953,-0.14762652942101953,-0.14762652942101953,1.008825598136005,1.008825598136005,1.008825598136005,0.9024759280882273,0.9024759280882273,0.9024759280882273,0.5071069075773074,0.5071069075773074,0.5071069075773074,0.49289309242269264,0.49289309242269264,0.49289309242269264,0.38144260930047724,0.38144260930047724,0.38144260930047724,0.12580463592446775,0.12580463592446775,0.12580463592446775,0.230451655791709,0.230451655791709,0.230451655791709,0.14762652942101953,0.14762652942101953,0.14762652942101953,0.00882559813600503,0.00882559813600503,0.00882559813600503,0.29107427049338286,0.29107427049338286,0.29107427049338286,0.44704167072724976,0.44704167072724976,0.44704167072724976,0.42301680641662437,0.42301680641662437,0.42301680641662437,0.10000384741557959,0.10000384741557959,0.10000384741557959,0.0,0.0,0.0,0.0

2023,250,250,intercept,50,19336.0,19336.0,19336.0,7383.468311248124,7383.468311248124,7383.468311248124,1831.6335,1831.6335,1831.6335,0.09462945164439508,0.09462945164439508,0.09462945164439508,0.854006543689197,0.8408840538736689,0.8587720029880306,0.416681978429176,0.39345491103233093,0.4275556225948651,0.06899341055669936,0.06766084186468772,0.07143738778307523,0.19470513888106433,0.16617890303895022,0.21025895352953797,-0.02097961142251207,-0.35101445419264143,0.41465258984785053,0.9570991998215856,0.8614819804975156,1.0031179859360684,0.9854432590081159,0.7901062371429876,1.3475568568949654,0.4731916619782288,0.3411074786522522,0.5496238357728225,0.5268083380217712,0.4503761642271775,0.6588925213477478,0.3981805849963045,0.34127303238800893,0.47159652177468897,0.11014021688316117,0.06921197230999458,0.1524017952856352,0.2350859764777301,0.23012492354871314,0.24392820377082794,0.11360261817752401,0.008903995098420138,0.41465258984785053,0.04290080017841441,0.0043252280842593915,0.13851801950248435,0.2669378228156429,0.19413234565124507,0.3406770534142406,0.43706696781861654,0.4114118681493869,0.45141179717448937,0.41357758282887,0.38905616753163014,0.4268708055768028,0.10000728048614843,0.09996156278394032,0.10026715953677325,0.0,0.0,1.0,0.42

2023,250,250,logistic,50,19336.0,19336.0,19336.0,7383.468311248124,7383.468311248124,7383.468311248124,1831.6335,1831.6335,1831.6335,0.09462945164439508,0.09462945164439508,0.09462945164439508,0.847786687270267,0.8321603961957863,0.8557927413659234,0.4036174415714878,0.3804337886243541,0.4192083457856155,0.07028581403939656,0.06840893363324348,0.07253083360087985,0.17962013475225758,0.15341614365533637,0.20152718549542645,-0.0208581485212591,-0.33964122340760583,0.43463422605509394,0.8585045621919563,0.6915669449712795,1.042354566738983,0.9859479210312888,0.7892026882157375,1.3405566347397706,0.4565730262085729,0.3350039486611268,0.5463783666328443,0.5434269737914271,0.4536216333671556,0.6649960513388731,0.400116485086639,0.3487197645180376,0.46689197896270823,0.10838156771650587,0.06779261281019518,0.14582925891920132,0.2411302327831033,0.23349813308797326,0.25170269099624937,0.129538545412698,0.009116093013493221,0.43463422605509394,0.1414954378080437,0.015330206155619964,0.3084330550287205,0.26336921632382637,0.19035088221547286,0.34128986993311805,0.43602794991465277,0.4052369892776039,0.45359127794943693,0.4124793356096256,0.38343570239453767,0.4289384314121979,0.10001973150205652,0.09993066489050084,0.10013891381460002,0.0,0.0,1.0,0.18

2023,250,250,refit,50,19336.0,19336.0,19336.0,7383.468311248124,7383.468311248124,7383.468311248124,1831.6335,1831.6335,1831.6335,0.09462945164439508,0.09462945164439508,0.09462945164439508,0.8183230779252739,0.7789959862928378,0.8335733896873171,0.33970654951061374,0.2935997726748308,0.3790309779230434,0.07382426799301639,0.07166143369323008,0.07718019998659079,0.1383191066951754,0.09914848493747662,0.1635638269230343,0.027720334048960883,-0.2601712301068212,0.4179126825584465,1.168698774542176,0.9679129604249115,1.3575990111399838,1.022544492067337,0.817431597973963,1.4148756351092289,0.30141772357843427,0.16863094882245822,0.43458536874325565,0.6985822764215657,0.5654146312567443,0.8313690511775418,0.40211396407432154,0.3387850411263052,0.4830249066300521,0.07078725443541997,0.030995793297698738,0.11628828287641797,0.25652864930769476,0.24955030108142312,0.27188785169025714,0.11274998113588927,0.010032348800931845,0.4179126825584465,0.16869877454217597,0.038977742305732734,0.35759901113998377,0.20861174655958686,0.14041588054169962,0.2723425870228147,0.3847177942530534,0.3436881969018365,0.4152856671381038,0.3639911368780735,0.32539215516456493,0.39297204844247324,0.10000685942482207,0.09993764598396383,0.10023906622175108,0.0,0.0,1.0,0.12

2023,500,500,intercept,50,19336.0,19336.0,19336.0,7383.468311248124,7383.468311248124,7383.468311248124,1831.6335,1831.6335,1831.6335,0.09462945164439508,0.09462945164439508,0.09462945164439508,0.8572624269128724,0.8514376341831423,0.8593383883747389,0.4213217738818814,0.4045945083316988,0.42805613777652224,0.06831800229915744,0.06777899302053435,0.0692550778389792,0.2025885409997178,0.1916509440522734,0.20887988677706387,0.02665019531860721,-0.18916808786976527,0.2353836612845576,0.985544656035008,0.9442940461819304,1.006442944187685,1.0188547098895804,0.8778111604959221,1.1832494257337107,0.46043441550943465,0.40561229088679585,0.5202312416758047,0.5395655844905654,0.4797687583241953,0.5943877091132042,0.40849249822616773,0.37523410835555454,0.4581576422353937,0.10671930284561046,0.08356373137479783,0.13072723780055276,0.2323274028481798,0.23051482497529774,0.23549213789003753,0.1038298950059075,0.017582633136722932,0.25835833782033224,0.01445534396499204,0.0018890459012076424,0.055705953818069635,0.2584729728538774,0.22069495591158375,0.3042238323580961,0.4418497750778199,0.4291337677543024,0.45394085115826943,0.4178648586706998,0.4042996739687638,0.42941333468330894,0.1000101917506564,0.09992415988048844,0.10028683627502534,0.0,0.0,1.0,0.46

2023,500,500,logistic,50,19336.0,19336.0,19336.0,7383.468311248124,7383.468311248124,7383.468311248124,1831.6335,1831.6335,1831.6335,0.09462945164439508,0.09462945164439508,0.09462945164439508,0.8553579959368597,0.846266733745459,0.8586025138616261,0.41597627436175844,0.39225414829107585,0.42558514181267326,0.0686554662659069,0.0679100865618177,0.07041167069969426,0.1986496431246399,0.17815112893043294,0.2073497557939809,0.015330221833355393,-0.17437053419931586,0.23479803277112138,0.9557221058170824,0.8382830684331628,1.0561371522898768,1.0110540475611558,0.8862207474845154,1.186115763001621,0.44840258708961156,0.3883788323373645,0.508679012477114,0.5515974129103884,0.49132098752288594,0.6116211676626355,0.4097622430264787,0.3774371090498286,0.4520644225540621,0.10291022967872407,0.08345472712195962,0.12570215011427321,0.23370768004712392,0.23153580105979668,0.23919554995147152,0.09692804066759672,0.011364821524651342,0.26510260932609314,0.05611876150584216,0.004204079821612689,0.16171693156683717,0.25432927373262587,0.21708334895479292,0.29923566039322264,0.4428142147432879,0.42409256273157264,0.455362560250181,0.4190050804364902,0.4015970164705056,0.4309342981576944,0.1000044596335817,0.09992470286877556,0.1001066522507668,0.0,0.0,1.0,0.4

2023,500,500,refit,50,19336.0,19336.0,19336.0,7383.468311248124,7383.468311248124,7383.468311248124,1831.6335,1831.6335,1831.6335,0.09462945164439508,0.09462945164439508,0.09462945164439508,0.8350359354450816,0.8206831770619873,0.8449072135130477,0.36568038109411277,0.34151186599528705,0.3916290224652901,0.07186558170294094,0.070130729312437,0.07345623333865411,0.1611810001892739,0.14261482730791508,0.1814302921665795,0.05925191707714388,-0.15681632327037315,0.25873266263270683,1.0987369515535697,0.9863326426578832,1.2022099017750187,1.046042535671718,0.8907720014268585,1.2268040427156999,0.3643168515972218,0.271568165792993,0.43815180547855237,0.6356831484027782,0.5618481945214476,0.7284318342070071,0.40620887434494357,0.3634932807565958,0.45781117959226825,0.08425832627978116,0.059012097236565225,0.11134486894641876,0.2473470596218445,0.2412054600311842,0.2529689731255276,0.09718701160240384,0.006748235128844238,0.25873266263270683,0.09873695155356965,0.007529708724241147,0.20220990177501874,0.2220279570256437,0.18572615494668265,0.26832429150168463,0.4080527026831514,0.37736711247091737,0.4314822615987314,0.3860892563489239,0.35708775448752755,0.408277605348205,0.10000057708650495,0.0999241684050429,0.10009527610368016,0.0,0.0,1.0,0.3

2023,1000,1000,intercept,50,19336.0,19336.0,19336.0,7383.468311248124,7383.468311248124,7383.468311248124,1831.6335,1831.6335,1831.6335,0.09462945164439508,0.09462945164439508,0.09462945164439508,0.8579178570470174,0.8544024379747216,0.8599589258424092,0.42388020501648105,0.4138816472778836,0.4301536185871951,0.0680330551682499,0.06751145136284482,0.06874218251566508,0.20591445949481574,0.1976374863151373,0.21200264763689347,-0.01296290600342884,-0.14644815215600762,0.1629275201246449,0.9919215491612674,0.9632215852541639,1.013725788968864,0.9909911109530928,0.9033773603250397,1.122094397819411,0.467801582576427,0.42216960161517025,0.5070540831449087,0.532198417423573,0.49294591685509126,0.5778303983848297,0.40475269106220907,0.3793539053221508,0.440823136876607,0.10973748851529402,0.09170577099369281,0.12534619132697053,0.23132403901531867,0.22963616705545617,0.2341143731955562,0.054694476108769215,0.005537407049138185,0.1817836097856174,0.011457036864676307,0.0019410111798287611,0.03677841474583614,0.2664914892009328,0.23300332538957594,0.2951233767364912,0.44286990273982213,0.4341708480435633,0.45388095653415383,0.4193438485849591,0.41084101854551025,0.42938334262708966,0.10000455779511791,0.09992689936230807,0.1001299338422761,1.0,0.0,1.0,0.76

2023,1000,1000,logistic,50,19336.0,19336.0,19336.0,7383.468311248124,7383.468311248124,7383.468311248124,1831.6335,1831.6335,1831.6335,0.09462945164439508,0.09462945164439508,0.09462945164439508,0.8563732795014973,0.8523344908393258,0.8592583840196475,0.42054222442793043,0.4064319864519273,0.42720923032532354,0.0683454275987232,0.06775390798840746,0.06924920991119242,0.2022684314033556,0.19171943482630513,0.20917268064389524,-0.011557349405113743,-0.14576202728247167,0.16811614060040653,0.9589859991620635,0.8829917013430569,1.043824971772223,0.9919869940303457,0.9039929288196451,1.1219105899122537,0.46580331163412325,0.4203525486949218,0.5112271832765671,0.5341966883658766,0.48877281672343287,0.5796474513050782,0.4036124844994638,0.38195977336648956,0.43845005552236793,0.11132700535506829,0.09055861911682589,0.12711735841437047,0.23273229167700143,0.23045646166015415,0.2354738198216112,0.059910549526113015,0.008343338512665978,0.17877165503424974,0.048209514572732315,0.007951951801968615,0.11976228904747269,0.2687556574491344,0.2316244823286366,0.3003943915798209,0.44373418590564107,0.4329787209067753,0.4516266463787652,0.4199869432628325,0.40965489807278743,0.4266465856232308,0.10001557513595774,0.09993898201413555,0.10010888465075596,1.0,0.0,1.0,0.66

2023,1000,1000,refit,50,19336.0,19336.0,19336.0,7383.468311248124,7383.468311248124,7383.468311248124,1831.6335,1831.6335,1831.6335,0.09462945164439508,0.09462945164439508,0.09462945164439508,0.8443120191472719,0.8342233902757051,0.8514419269632522,0.3863258706762948,0.3646625610993788,0.4067784836865267,0.07024154969085981,0.06915040011544328,0.07149516909462442,0.18013679064910038,0.16550447641061278,0.1928727481659842,0.006277196584871316,-0.1075889005569248,0.18786812039033576,1.004631625441557,0.9210327742561804,1.0845405446488297,1.004678226947359,0.9261412302957893,1.1453714183182606,0.41994495623715117,0.36195317731412974,0.46048047821794047,0.5800550437628489,0.5395195217820595,0.6380468226858702,0.3997997501792746,0.3677517458500464,0.45079542430335795,0.0999752539933624,0.07934053767712804,0.1116117471728948,0.2405635502494341,0.23648722766395366,0.24547907268574304,0.06158867059814124,0.01128114292101352,0.18786812039033576,0.026106028807583725,0.005022718235209051,0.09380330061860367,0.24843703129387595,0.21075198759840422,0.2694335483855436,0.42243748544673376,0.40227926602128644,0.4429142893488245,0.40002768966442737,0.3803538175307965,0.4188784911805599,0.10001847348447411,0.09991872043978355,0.100248967879236,1.0,0.0,1.0,0.7

2023,2000,2000,intercept,50,19336.0,19336.0,19336.0,7383.468311248124,7383.468311248124,7383.468311248124,1831.6335,1831.6335,1831.6335,0.09462945164439508,0.09462945164439508,0.09462945164439508,0.8587247703354155,0.8569712547169512,0.859832055388357,0.4263181297062246,0.42123166221271513,0.42971004957206965,0.0677708781398299,0.06755132718876362,0.06813334861033912,0.2089746041084315,0.20474382895391788,0.21153721481604604,0.0014074813610627946,-0.07915315407184732,0.09674652288081424,1.0022563732676064,0.985753034230805,1.0091507871138279,1.0009947249841131,0.9462943955608586,1.0707318653935782,0.46500462565245726,0.4397134989068501,0.49180106991928246,0.5349953743475427,0.5081989300807175,0.5602865010931499,0.40809544243418017,0.39475781084779177,0.4288590345277194,0.10820738782010524,0.09813281207284395,0.11706551765385373,0.23042471970939427,0.2297144712829347,0.23168299307091567,0.0402174236601473,0.0043570780877594155,0.15140955059198322,0.005221913296389102,0.0011221304180842773,0.014490600713622327,0.2631751347596966,0.24502486710635518,0.28216436385445,0.4469678568338044,0.4407265045108643,0.4528186424849731,0.4228869494906391,0.41724235507608215,0.4280616046934922,0.1000044725495733,0.09993145560750695,0.100143596119877,1.0,0.0,1.0,0.92

2023,2000,2000,logistic,50,19336.0,19336.0,19336.0,7383.468311248124,7383.468311248124,7383.468311248124,1831.6335,1831.6335,1831.6335,0.09462945164439508,0.09462945164439508,0.09462945164439508,0.8578723316578402,0.8558759495122633,0.8596033424073369,0.42498439721777004,0.4132188960390116,0.4286211156597044,0.06787116208452959,0.06756864378011092,0.06859761933064298,0.2078040844215835,0.19932483571656467,0.21133509461483335,0.005109811710867796,-0.08019063775712727,0.09234754136566628,1.0010238885386777,0.9185125972441843,1.0738865955307189,1.0035928675645822,0.9466473732970648,1.0678934790923256,0.4630982672024726,0.44033533182265994,0.4992632286972257,0.5369017327975274,0.5007367713027743,0.5596646681773401,0.4103650118463016,0.39053159407140126,0.43057064715755905,0.10648971592841883,0.09713510364357385,0.11992373980077746,0.23111998658828403,0.22990375670205548,0.23279740822413902,0.039683146374132755,0.005549653014064823,0.15354666959247226,0.034847554984726026,0.0031097016422037666,0.09402617105265465,0.2610139390913706,0.24315934774320305,0.2902467860319238,0.4467715566460212,0.44121920406020093,0.4517209228811332,0.42278777867364553,0.4170453409434572,0.4274593044590004,0.10000075532718912,0.09995043927364948,0.10011361629512087,1.0,0.0,1.0,0.86

2023,2000,2000,refit,50,19336.0,19336.0,19336.0,7383.468311248124,7383.468311248124,7383.468311248124,1831.6335,1831.6335,1831.6335,0.09462945164439508,0.09462945164439508,0.09462945164439508,0.853019788312942,0.8464651497786003,0.8587998031152898,0.40600846046811256,0.3886879639374591,0.4286851294932172,0.06911498127464277,0.06801293282075077,0.07013624239211814,0.19328615881278793,0.18136594319904265,0.20614932863527347,0.013464074519292754,-0.1411997175138233,0.11967786051039037,0.9778978268502878,0.9154245736112661,1.0561887765365823,1.0095139527840773,0.9073834110031969,1.088638971609844,0.4416095523476722,0.4097858960321483,0.49099524004119816,0.5583904476523278,0.5090047599588018,0.5902141039678516,0.41076630419045146,0.3783185343480774,0.43094067160435817,0.1019878832189563,0.09219523024519363,0.11636883655790621,0.2355293441883763,0.2318313690582882,0.23930786742533652,0.03825059830655525,0.004588680337991631,0.16028940215880436,0.04078680830409198,0.006160268495390725,0.08457542638873394,0.2521820670406657,0.23524151644960534,0.2786148398585014,0.43659028948749845,0.41993358114491797,0.4549221610109228,0.41316830219821027,0.39725787477881197,0.43050269397456026,0.10000784362338244,0.09995644985950321,0.10016724891698506,1.0,0.0,1.0,0.84

2023,4000,4000,intercept,50,19336.0,19336.0,19336.0,7383.468311248124,7383.468311248124,7383.468311248124,1831.6335,1831.6335,1831.6335,0.09462945164439508,0.09462945164439508,0.09462945164439508,0.8588743742575384,0.8577620387367171,0.8596873558565296,0.4268050421710547,0.4237202895766952,0.42883696501675006,0.06772731430607132,0.06756250097318096,0.06794818701626444,0.209483083558474,0.20690504520604663,0.2114067937932567,0.0013213735211602623,-0.06528509867869499,0.083125516988547,1.0029295272368632,0.993833205979688,1.0097481674656676,1.000930647325092,0.9554123615384038,1.0606291916515478,0.4650568740962644,0.4432588069611088,0.48393130776435356,0.5349431259037356,0.5160686922356464,0.5567411930388911,0.4091846405457435,0.39454573797032294,0.42138019257232684,0.1076306319645384,0.09871494821387412,0.11600808206292028,0.23026709751714336,0.22969820070535246,0.2309153026510748,0.03510907120440655,0.004581353692234898,0.08483979562113825,0.004663721387996089,0.00047652526438555224,0.01024724147448318,0.2620551481600425,0.2461435398377689,0.2762596474464849,0.44676970037947006,0.4406752333368002,0.4526796354183301,0.4229418696347296,0.4168987871302801,0.42837325090472356,0.10000267722674008,0.0999337869439918,0.10013798722136222,1.0,1.0,1.0,0.98

2023,4000,4000,logistic,50,19336.0,19336.0,19336.0,7383.468311248124,7383.468311248124,7383.468311248124,1831.6335,1831.6335,1831.6335,0.09462945164439508,0.09462945164439508,0.09462945164439508,0.858422168357027,0.8571011353456347,0.8595846386274613,0.4246665982288626,0.4204889530935081,0.4285918586991019,0.06782120034431173,0.06762191219859552,0.06812215903632451,0.2083872405857065,0.20487443427375007,0.21071334272114595,0.003582687541662141,-0.06638475371299939,0.07760153813022085,0.9848629409418996,0.9562453957908978,1.043252353688211,1.002486686506011,0.9551298292652372,1.056315858812038,0.467666511886794,0.44376778160041297,0.48573185902092314,0.532333488113206,0.5142681409790769,0.556232218399587,0.4066274240493868,0.3925323833205244,0.42409150598373885,0.10764372878002679,0.09916145301041102,0.11652134497031112,0.23077138858477558,0.22991589431560508,0.2315632348901849,0.03203342851819753,0.0048926122744098926,0.07854189453392771,0.022637708761629072,0.004268284178247306,0.048557576391897964,0.2632766992214601,0.24676601765994044,0.2786157286980727,0.4464785668093535,0.4405963010613204,0.4529341077240617,0.42196348509006754,0.41697261251205653,0.4286136950520962,0.10001045265368683,0.09992046745680813,0.10015691069898387,1.0,1.0,1.0,0.98

2023,4000,4000,refit,50,19336.0,19336.0,19336.0,7383.468311248124,7383.468311248124,7383.468311248124,1831.6335,1831.6335,1831.6335,0.09462945164439508,0.09462945164439508,0.09462945164439508,0.8579914934775167,0.853329708322323,0.8630187671253882,0.41885014370734885,0.4067464084769306,0.42898606922805643,0.06821970761254173,0.06749234920815363,0.06908990344262962,0.20373584195742117,0.19357886863949794,0.21222560903110477,-0.01170228201223274,-0.07018021831771463,0.07412852875182875,0.9717664016441314,0.9287881074573375,1.0200421256994159,0.9918150452761492,0.9532182754230644,1.0522431460965016,0.46692782699158975,0.4420334936001116,0.4927278464823886,0.5330721730084103,0.5072721535176113,0.5579665063998883,0.4172619571505087,0.39143125667309664,0.4349064229889467,0.1067562348320084,0.09681159990165869,0.11512386025221533,0.2317464686873063,0.22966791808247028,0.2347623678790243,0.03670811541601175,0.006802982052935587,0.08833688592280119,0.030753317600737407,0.007719870184672795,0.0712118925426625,0.2610906983555028,0.24258042336457827,0.2777964725250412,0.45109458305932937,0.439080148949012,0.46595418515767484,0.42674881775599455,0.41538859349400953,0.44114625812365665,0.10000686717441704,0.09992554964118523,0.1001128338443494,1.0,1.0,1.0,0.98
